# Data related to the microstructural identification and analyzing the mechanical properties of maraging stainless steel 13Cr10Ni1.7Mo2Al0.4Mn0.4Si (commercially known as CX) processed by laser powder bed fusion method

**DOI:** 10.1016/j.dib.2022.107856

**Published:** 2022-01-24

**Authors:** Shahriar Afkhami, Vahid Javaheri, Edris Dabiri, Heidi Piili, Timo Björk

**Affiliations:** a Laboratory of Steel Structures, LUT University, Lappeenranta, 53850, Finland; b Materials and Mechanical Engineering, University of Oulu, 90014, Finland; c Laboratory of Laser Materials Processing and Additive Manufacturing, LUT University, Lappeenranta, 53850, Finland; d Department of Mechanical and Materials Engineering, University of Turku, Turku, 20520, Finland

**Keywords:** Additive manufacturing, CX, Scanning electron microscopy, Electron backscatter diffraction, Quasi-static tensile test, Digital image correlation

## Abstract

The data available in this article presents the microstructural information achieved via scanning electron microscopy and electron backscatter diffraction to evaluate the microstructure of maraging stainless steel 13Cr10Ni1.7Mo2Al0.4Mn0.4Si, in its as-built and heat-treated conditions, fabricated by laser powder bed fusion. In addition, the statistical analysis of the defects is included to indicate the quality of the additively manufactured metal. Furthermore, true stress-logarithmic strain diagrams of the material with different types of post-processing are available, indicating the strain hardening behavior of the material. These diagrams were achieved via quasi-static tensile tests performed in conjunction with the digital image correlation technique. Finally, the sample designs, additive manufacturing parameters, and the heat treatment procedure carried out on the material are also available in this paper to guide future research and ensure the repeatability of the data in this data article and its linked research paper. The research paper investigates the effects of processing and post-processing parameters on the microstructure, surface quality, residual stress, and mechanical properties of 13Cr10Ni1.7Mo2Al0.4Mn0.4Si (conventionally known as CX developed by EOS GmbH) processed by laser powder bed fusion [1].

## Specifications Table

**Table:** 

Subject	Materials science
Specific subject area	Additive manufacturing
Type of data	Table Images Graphs Figures
How the data were acquired	The schematic illustrations of the specimens and their dimensions in Figs. 1 and 2 are based on ASTM E8[2] and E23 [3] standards for the tensile and Charpy specimens, respectively. The heat treatment process (Fig. 3) was plotted based on data available in [1] and [4]. Statistical analysis and distribution of the defects in Fig. 4 are carried out by analyzing the numerical data obtained by ImageJ software from the surface of the specimens, available in “Table A.docx” in the Supplementary material. Microstructural images (Figs. 5 and 6) were obtained by scanning electron microscopy (SEM) using a Hi-Tech Instruments SU3500 equipped with an energy dispersive X-ray spectroscopy probe (EDS) for elemental analysis. Overall results available in “EDS.docx” in the supplementary material were used for Fig. 9. The EDS parameters are also available in “EDS.docx”. Electron backscatter diffraction (EBSD) data were collected using a ZEISS Sigma field emission scanning electron microscope (FE-SEM). The acquired data were analyzed using TSL software to generate the maps of inverse pole figures (IPF), grain boundaries, image qualities (IQ), and orientation diffraction functions (ODF) in Figs. 7 and 8. The raw diffraction data for the horizontal (as-built), vertical (as-built), and vertical (heat-treated) specimens are available in files ``Horizontal (as-built).txt'', ``Vertical (as-built).txt'', and ``Vertical (heat-traeted).txt'', respectively, in the Supplementary material. Local true stress-logarithmic strain values in Fig. 10 are obtained using an ARAMIS Digital image correlation (DIC) system in conjunction with a Galdabini Quasar 600 universal testing machine. The raw data from the DIC system (turned into numerical values via GOM Correlate software (version 2020)) are available in"DIC.xlsx" (stage 844 for the last moment with stable stress flow prior to the final fracture) in the Supplementary material. True stress-logarithmic strain curves were generated using the Galdabini Quasar 600 universal testing machine in conjunction with DIC system. DIC data were turned into numerical values using GOM Correlate software (version 2020). Force-strain data used to plot the curves in Fig. 11, including the strain hardening curves, are available in ``Table B.docx'' in the Supplementary material. Shear fracture areas (SFA) in Fig. 12 were determined by topological analysis of the fracture surfaces using a KEYENCE VE-3200 3D measuring microscope. The data obtained were analyzed using VR-3000 G2 software (version 2.5.0.116). The raw data for horizontal (as-built), horizontal (heat-treated), vertical (as-built), and vertical (heat-treated) speciemens are available in “Horizontal (as-built).zon”,
	“Horizontal (heat-treated).zon”, “Vertical (as-built).zon”, and “vertical (heat-treated).zon”, respectively, in the Supplementary material. >The schematic representation of the microstructural features in a solidified laser passage (Fig. 13) was based on the concept from the literature and discussed in the linked research paper [1].
Data format	Raw Analyzed
Description of data collection	Images from SEM were acquired by mounting 10 mm × 10 mm × 10 mm cuboidal specimens in epoxy resin. A scanning voltage of 15 kV was used for taking the images through the secondary electron detector of the SEM instrument. EBSD data were acquired with an accelerating voltage, working distance, and scan step of 15 kV, 15 mm, and 200 µm, respectively. Quasi-static tensile tests and Charpy tests were performed according to ASTM E08 and ASTM E23, respectively.
Data source location	• Institution: LUT University • City/Town/Region: Lappeenranta, South Karelia • Country: Finland
Data accessibility	With the article
Related research article	S. Afkhami, V. Javaheri, E. Dabiri, H. Piili, T. Björk, Effects of manufacturing parameters, heat treatment, and machining on the physical and mechanical properties of 13Cr10Ni1• 7Mo2Al0• 4Mn0• 4Si steel processed by laser powder bed fusion, Mater. Sci. Eng. 832 (2022 January 14) 142402, doi:10.1016/j.msea.2021.142402

## Value of the Data

• The images from the SEM and the EBSD data in this article provide insight into the nature and properties of the microstructural features of maraging steel CX processed with L-PBF.
• It is possible to identify the microstructural changes associated with the heat treatment performed on L- PBF CX.
• True stress-logarithmic strain data can be used for calibrating finite element models to simulate material behavior and failure under mechanical loading.
• The data can be useful to researchers studying the correlations between microstructure and mechanical properties of additively manufactured metals.
• The data can be reused to estimate the mechanical performance of industrial components made from additively manufactured metals.

## 1 Data Description

**Fig. 1:**
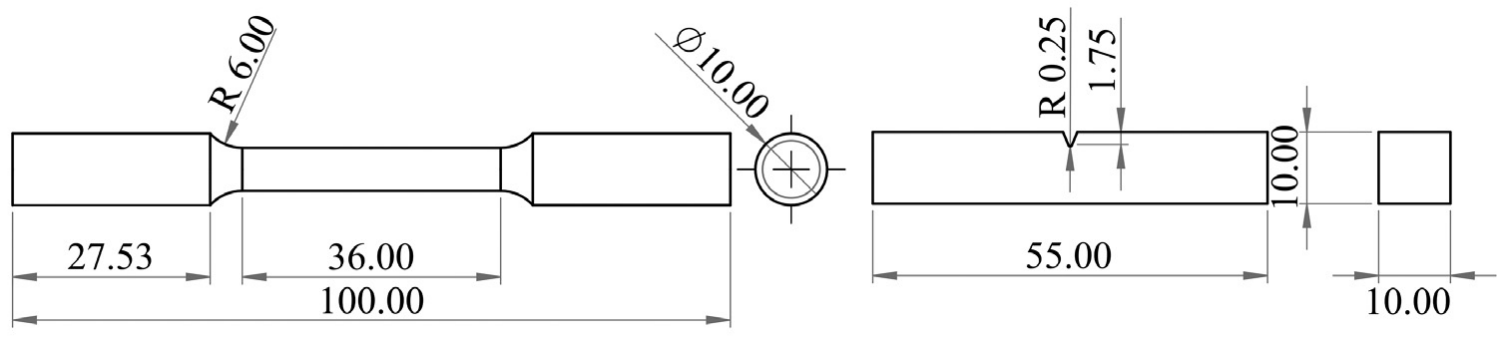
Schematic view and dimensions of the specimens used for the mechanical tests: (left) tensile and (right) Charpy samples (dimensions are in mm).

**Fig. 2:**
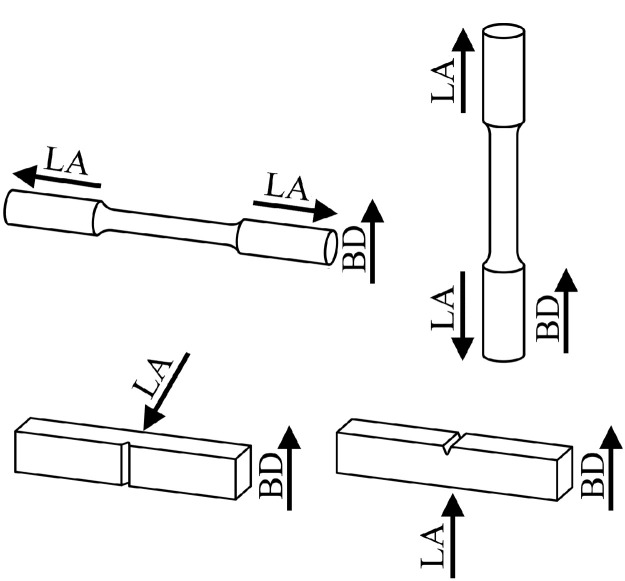
Building direction (BD) and loading axis (LA) in the (left) horizontal and (right) vertical samples manufactured using L-PBF for the (top) tensile and (bottom) Charpy tests.

**Fig. 3:**
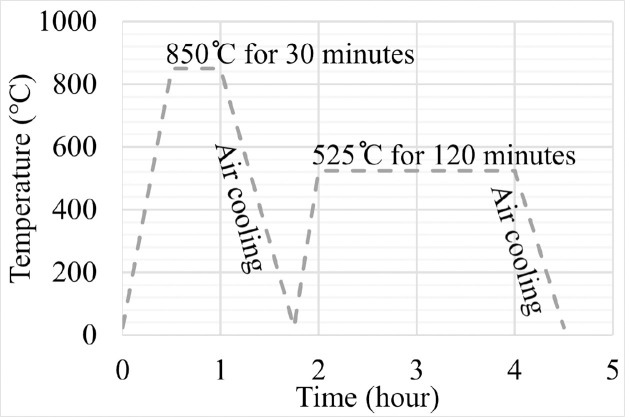
Heat treatment procedure.

**Fig. 4:**
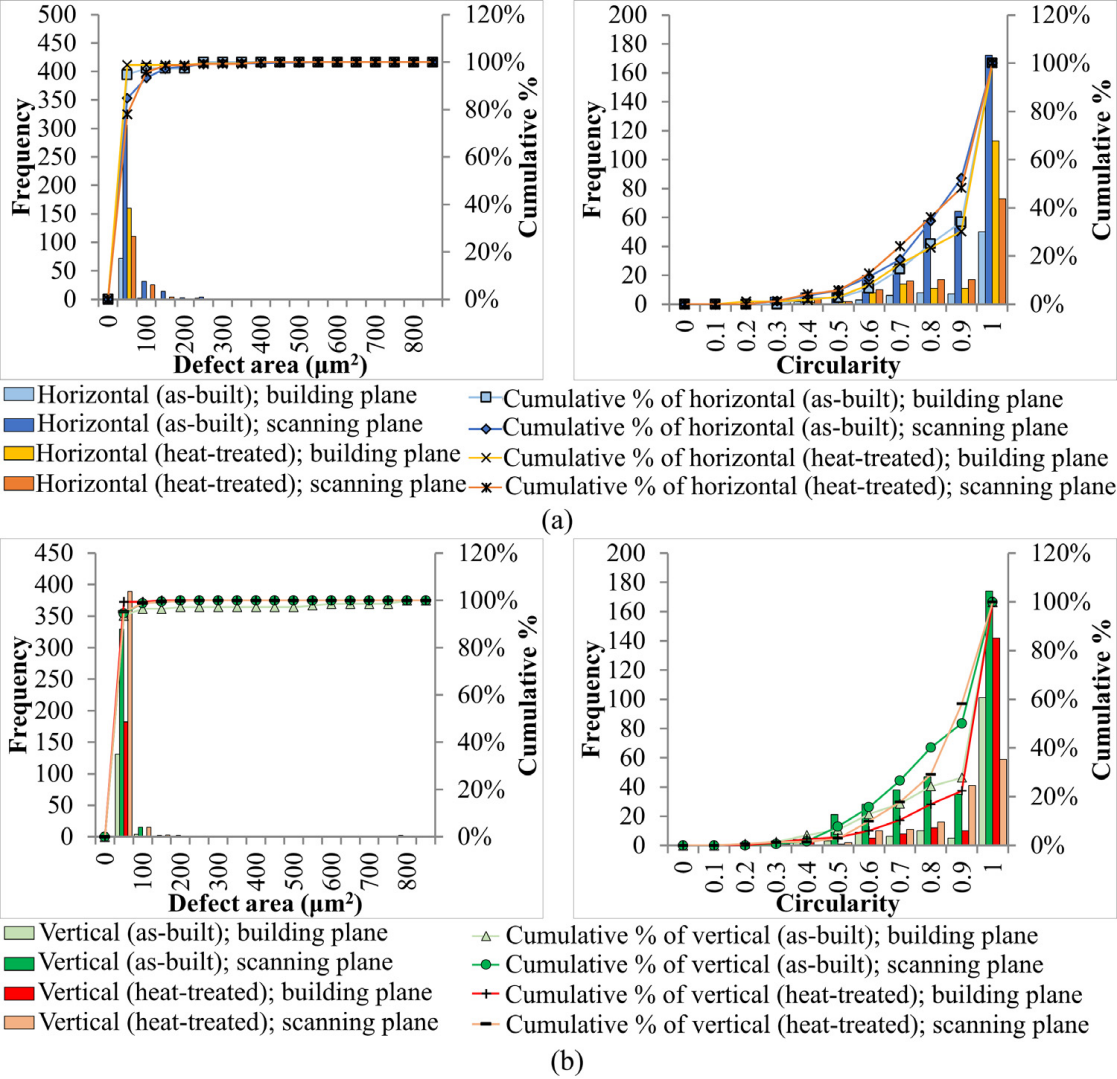
(left) Defect size and (right) shape distribution data from (a) horizontal and (b) vertical specimens.

**Fig. 5:**
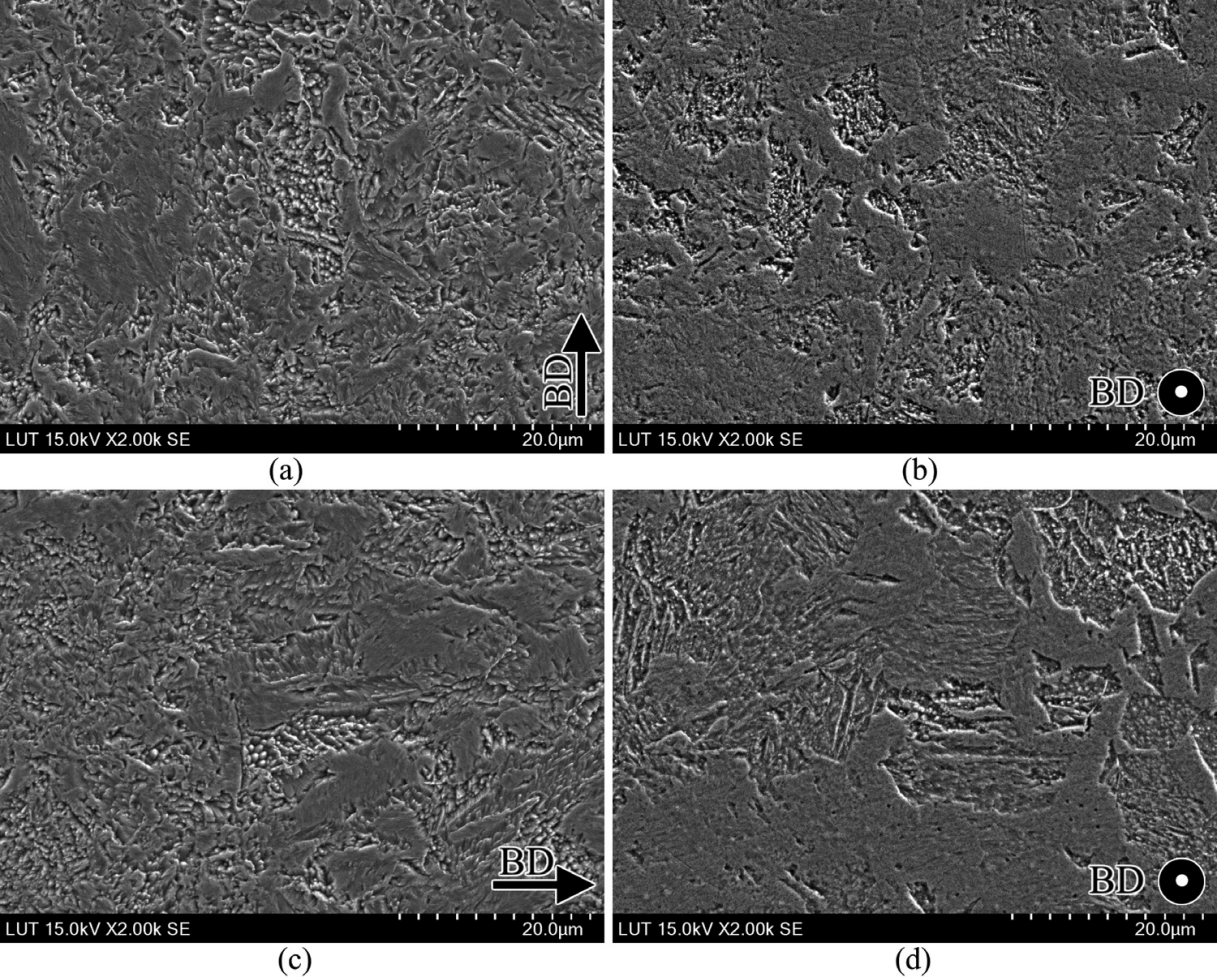
SEM micrographs of the as-built samples in their (left) building and (right) scanning planes: (a, b) horizontal specimens and (c, d) vertical specimens.

Metal samples of maraging stainless steel 13Cr10Ni1.7Mo2Al0.4Mn0.4Si, as shown in Figs. 1 and 2, were fabricated via L-PBF using the AM parameters listed in Table 1. Subsequently, the specimens were divided into two groups: as-built and heat-treated. The latter group was subjected to a heat treatment process proposed by the powder manufacturer (Fig. 3) [4]. After heat treatment, the polished and unetched surfaces of the samples were examined to determine the size and distribution of the defects detected in the samples (Fig. 4) based on the data available in “Table A.docx” in the Supplementary material. Next, microstructural samples were etched and subjected to SEM and EBSD analysis, as shown in Fig. 5 through Fig. 9. Raw EBSD data used in these figures are available in “Horizontal (as-built).txt”, “Vertical (as-built).txt”, and “Vertical (heat-treated).txt”. Also, EDS parameters are available in “EDS.docx” in the Supplementary material.

**Table 1:** Parameters of the additive manufacturing process using an EOS M290 system.

Laser power (W)	Scanning speed (mm/s)	Hatching distance (mm)	Layer thickness (mm)	Scanning strategy	interlayer scanning rotation (degrees)
260	1000	0.1	0.03	Stripe	67

**Fig. 6:**
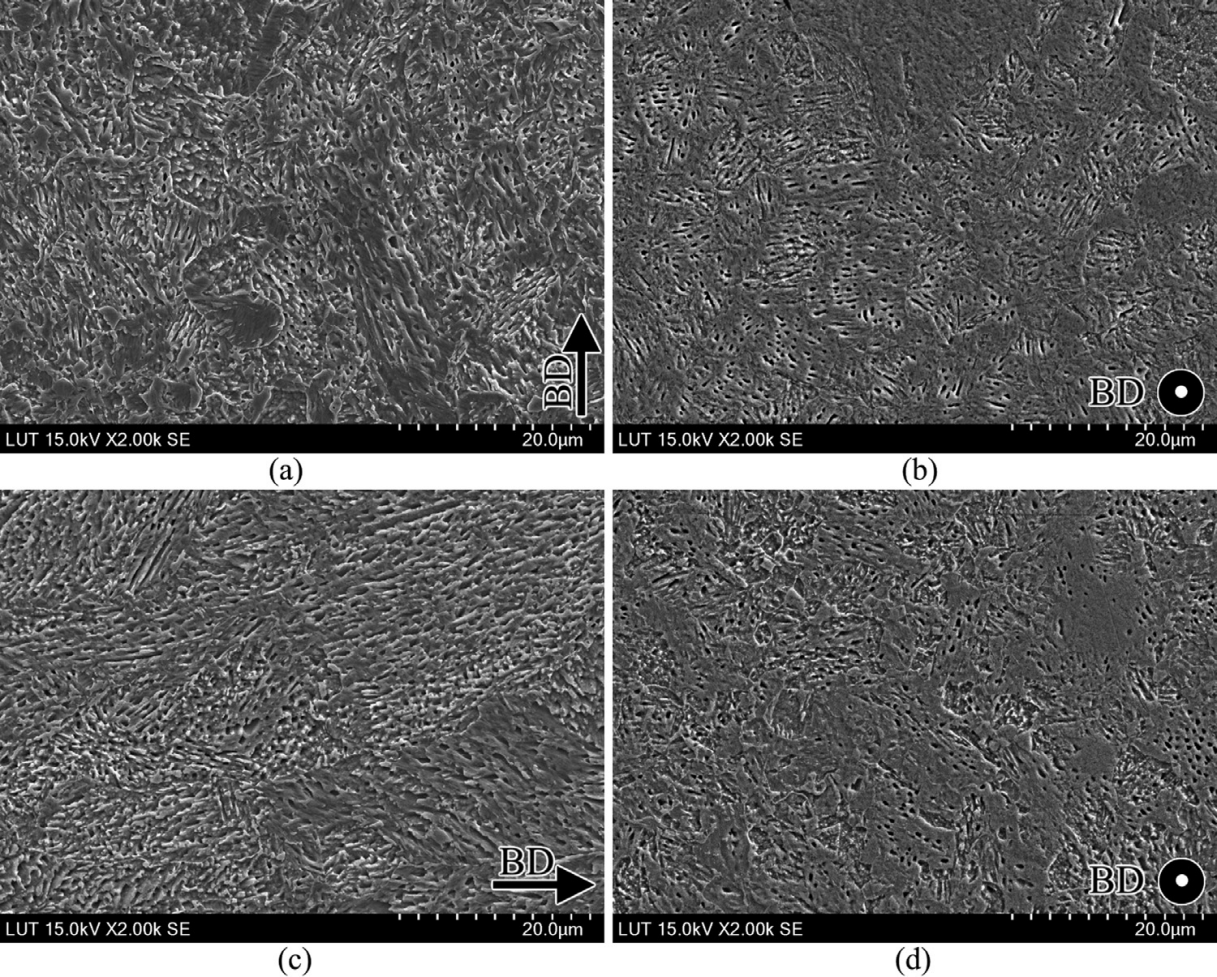
SEM micrographs of the heat-treated samples in their (left) building and (right) scanning planes: (a, b) horizontal specimens and (c, d) vertical specimens.

**Fig. 7:**
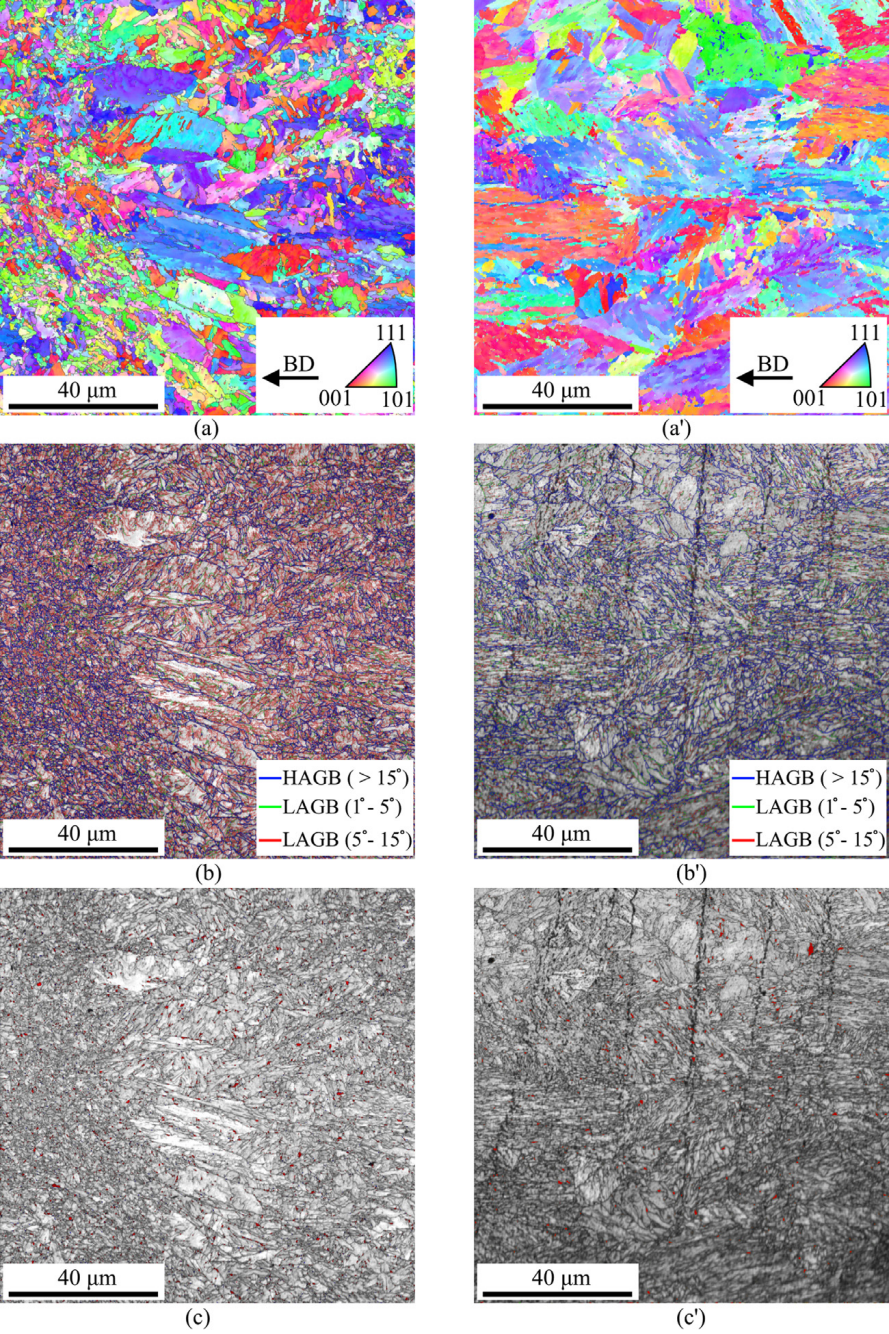
Results of the EBSD analysis for the vertical specimens from their building plane: (a, a') IPF maps, (b, b') grain boundary maps, and (c, c') image quality (IQ) maps of the (left) as-built and (right) heat-treated samples (retained austenites are superimposed on the IQ maps as red spots, and the remaining areas are martensitic). More details on the phase fractions and grain boundaries are available in [1].

**Fig. 8:**
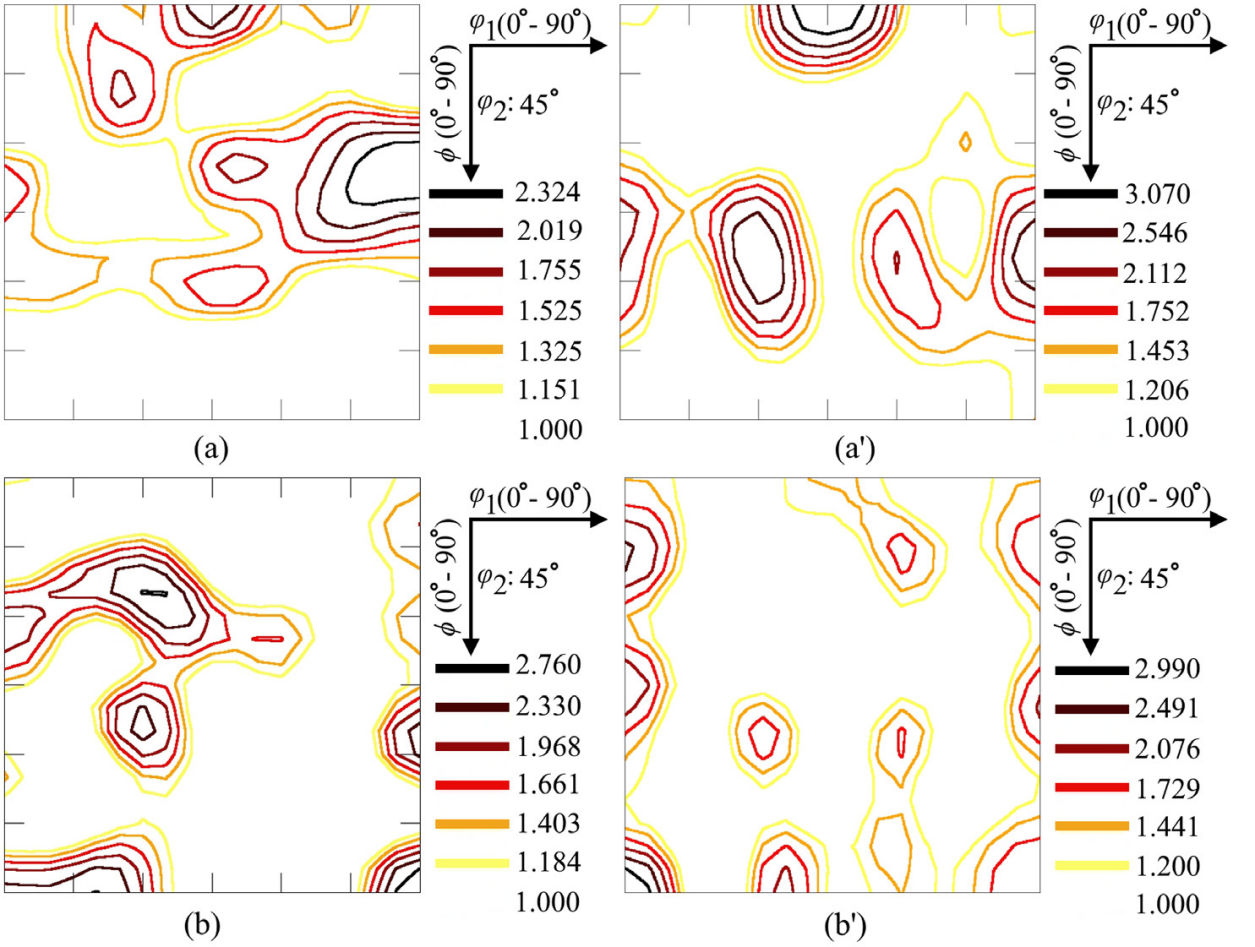
ODF maps of (a, a') martensitic backgrounds and (b, b') austenitic features from the as-built (left) horizontal and (right) vertical samples.

**Fig. 9:**
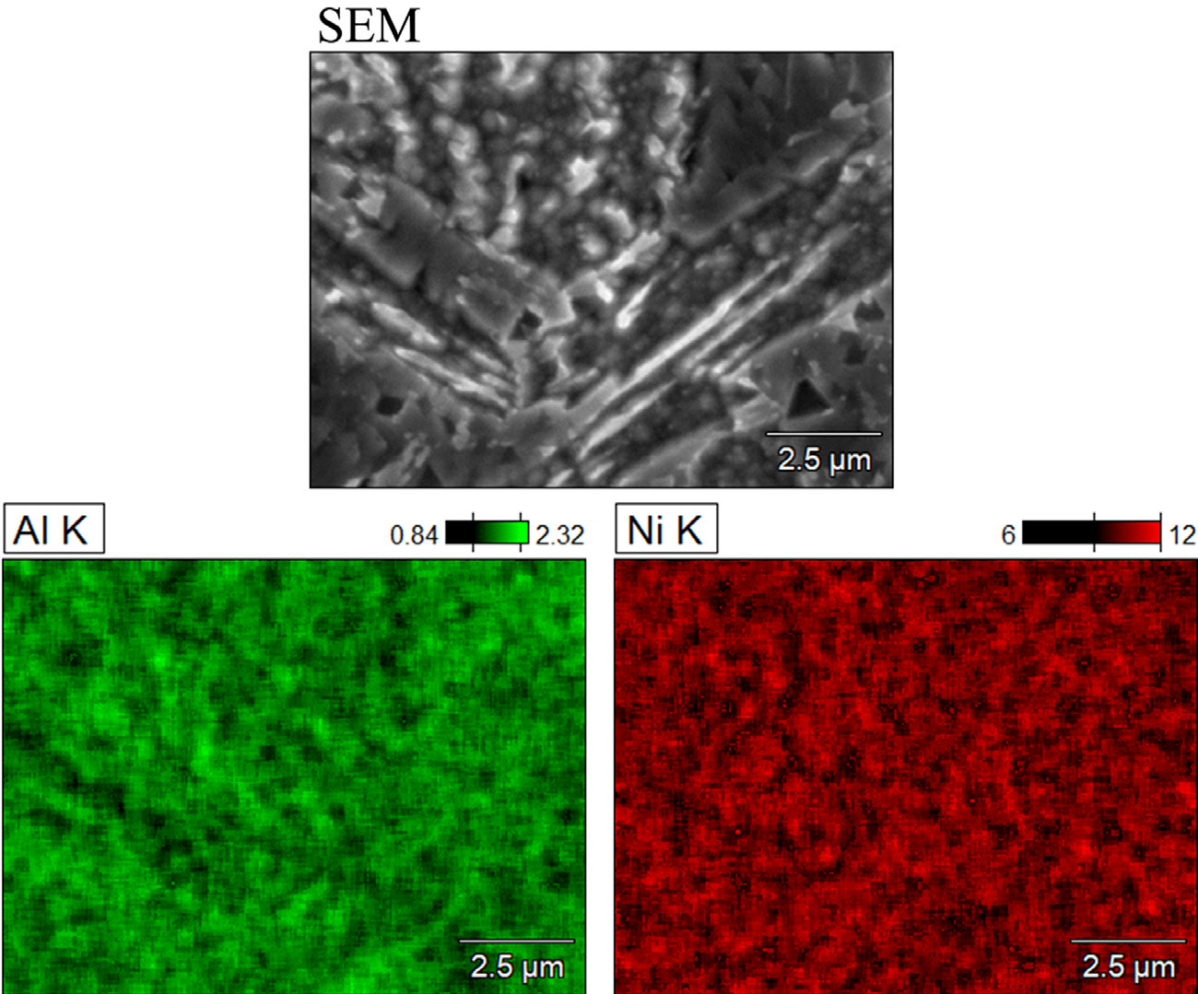
Microscale EDS mapping of a horizontal (heat-treated) specimen showing Ni and Al distributions throughout the microstructure.

**Fig. 10:**
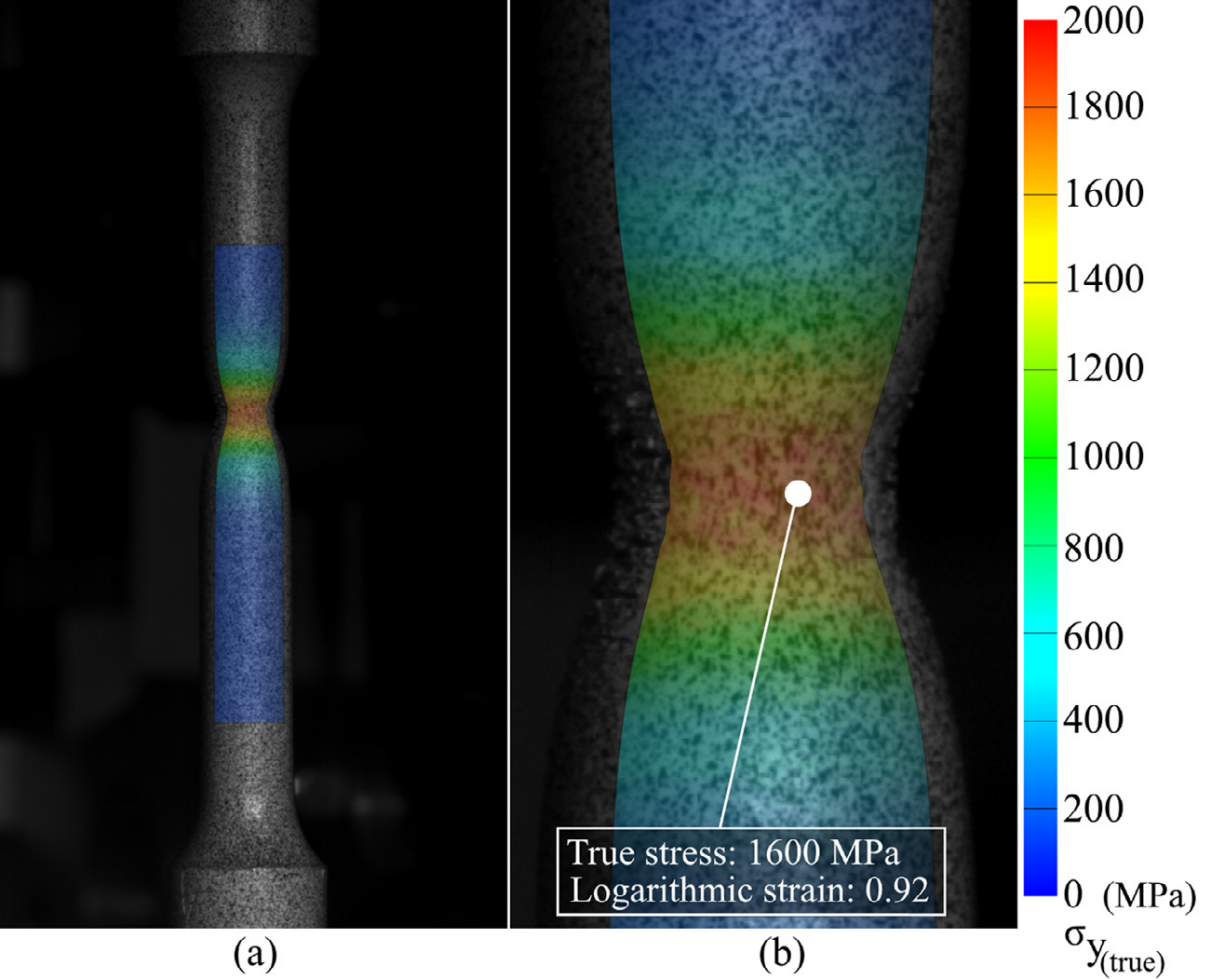
(a) DIC data of a heat-treated horizontal sample prior to its final fracture; (b) magnified view of its necking region showing its local stress and strain values.

**Fig. 11:**
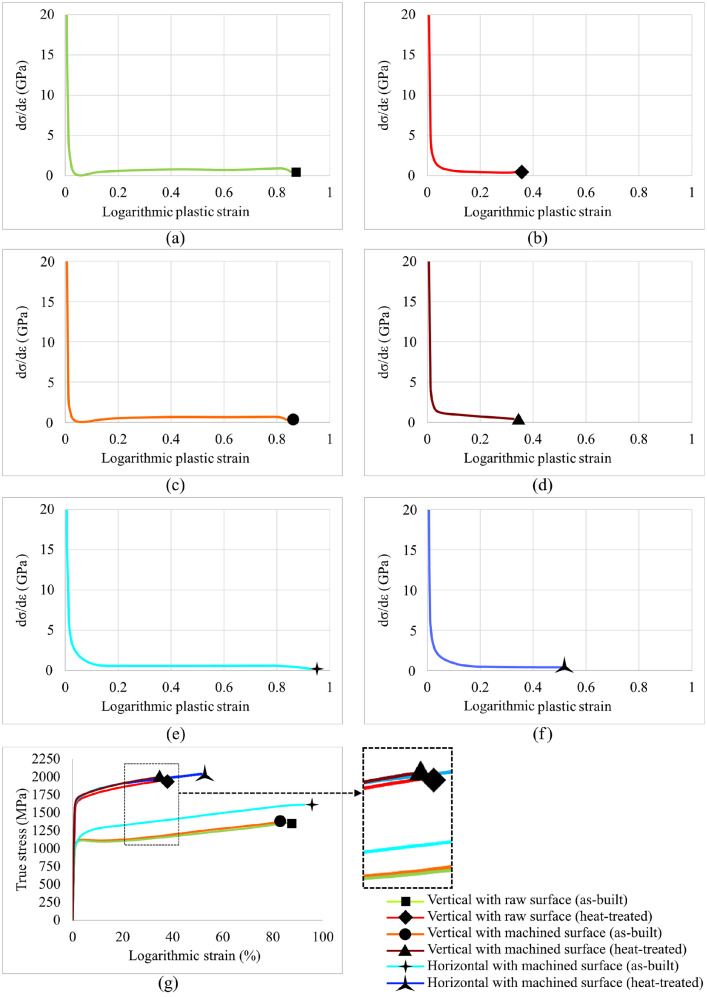
Strain hardening and true stress-logarithmic strain curves of L-PBF CX according to its BDs and post-processing conditions.

**Fig. 12:**
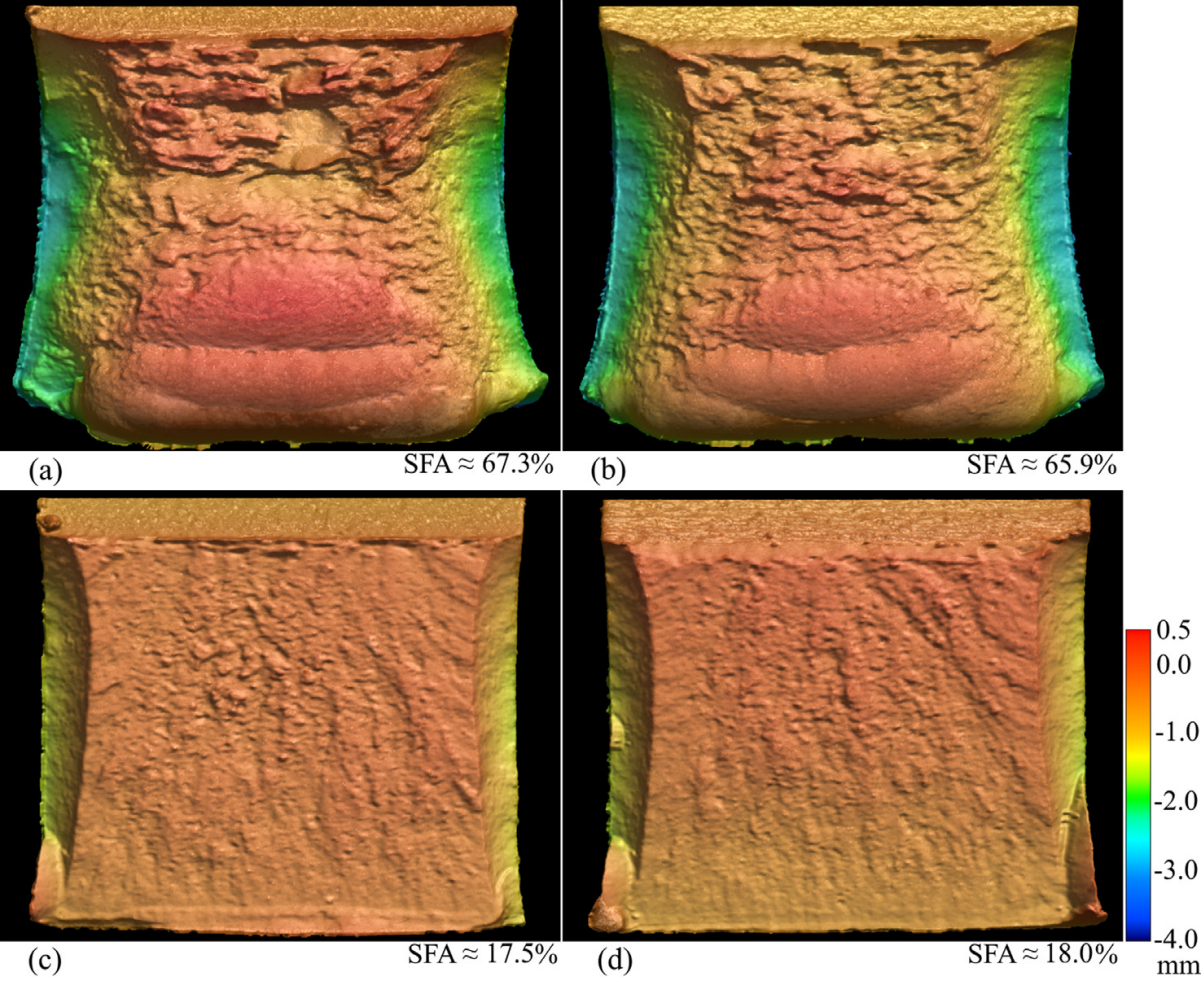
Topological analysis of the fracture surface from (a) horizontal (as-built), (b) vertical (as-built), (c) horizontal (heat-treated), and (d) vertical (heat-treated) specimens.

**Fig. 13:**
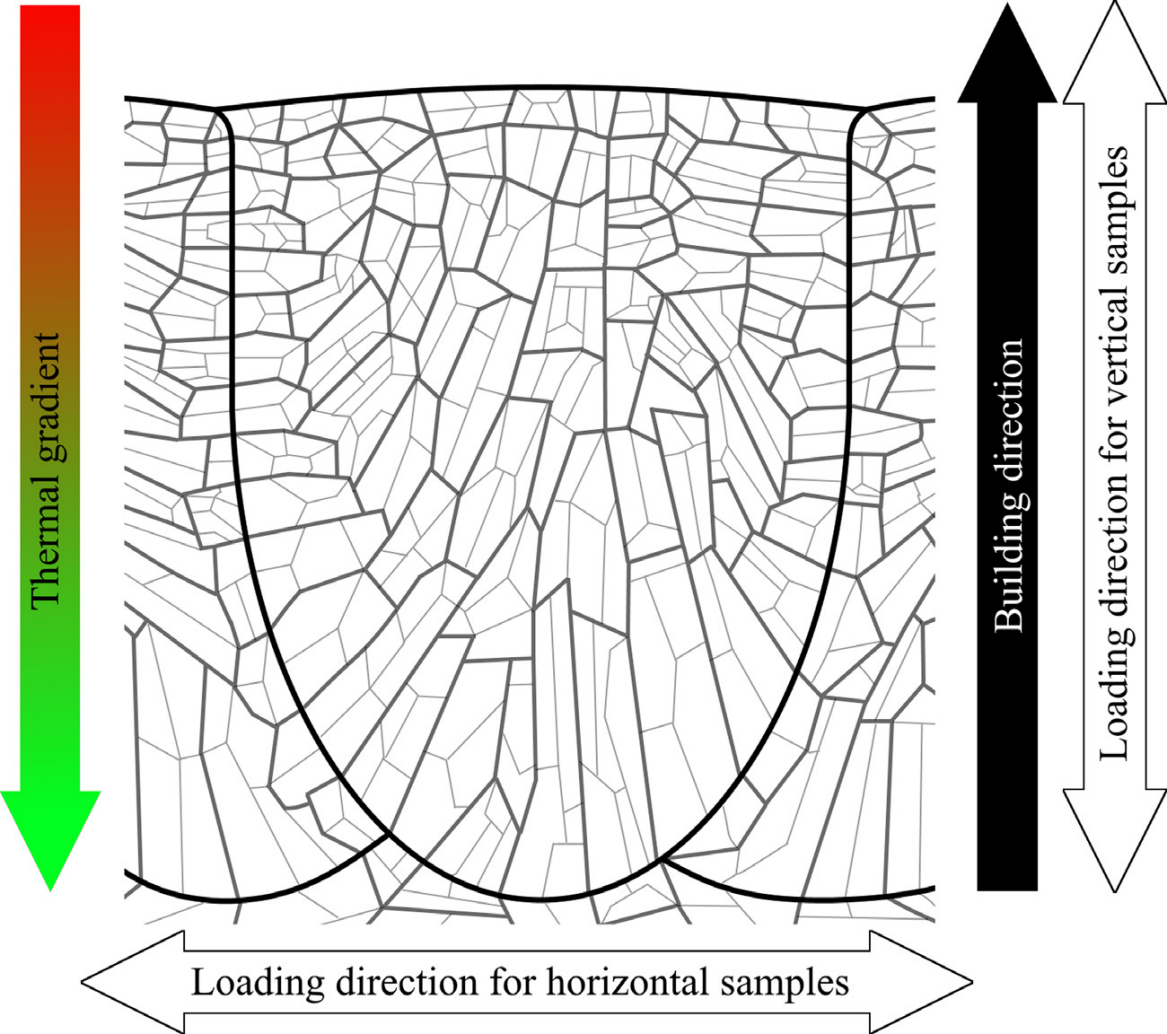
Schematic view of the prior austenites and martensitic packets of a solidified laser pass in L-PBF.

Quasi-static tensile tests were then performed on standard specimens in conjunction with the DIC technique to determine local stress-strain values (Fig. 10) and plot true stress-logarithmic strain curves (Fig. 11). Data related to Fig. 10 and Fig. 11 are available in “DIC.xlsx” and “Table B.docx”, respectively, in the Supplementary material. Next, the Charpy test was carried out to estimate the notch toughness of the material (results can be found in [1]), and the shear fracture area of each sample type was calculated by analyzing the surface of the fractured specimens (Fig. 12). Data used for Fig. 12 are available in “Horizontal (as-built).zon”, “Horizontal (heat-treated).zon”, “Vertical (as-built).zon”, and “Vertical (heat-treated).zon” in the Supplementary material. Finally, the crystallographic growth pattern of the material during the L-PBF process was realized based on discussions in the literature (more details can be found in [1]) and schematically shown in Fig. 13.

## 2 Experimental Design, Materials and Methods

An EOS M290 metal additive manufacturing (AM) system was used to fabricate the samples. The AM system was equipped with a Yb-fiber laser with a maximum output of 400 W. The specimens were manufactured in a sealed chamber of 250 mm × 250 mm × 325 mm, under an argon atmosphere with a purity of 99.9%, and on a substrate made of stainless steel 316L. The raw metal powder used in the AM process was purchased from EOS GmbH. The powder had a general particle size distribution of 20 - 65 µm. After AM, the samples were separated from the substrate by mechanical sawing. After separation, the samples were divided into two groups: as-built and heat-treated. The second group was heat-treated in the sealed chamber of an electric furnace and under an argon atmosphere with 99.9% purity. Next, cuboids of 10 mm × 10 mm × 10 mm from both groups were mounted in epoxy resin, ground sequentially with different grinding pads (up to 2000 grit), and polished with 1-µm colloidal silica solution. Optical images were taken from the building and scanning planes of the unetched polished specimens using a Nikon Eclipse ME600 to analyze the defect distributions. The visual data were investigated using the open-source ImageJ software (version 1.52v).

For the microstructural analyses by SEM and EBSD, the polished samples were etched by immersion in Kalling's reagent (1.5 g CuCl_2_ + 33 ml HCl + 33 ml C_2_H_5_OH + 33 ml distilled water) for 15 s, according to the instructions in [5]. After etching, the samples were washed with ethanol, dried under a stream of hot air, and stored in a desiccator filled with silica gel. For SEM, the prepared specimens were cleaned with a jet stream of carbon dioxide and then placed in the chamber of a SU3500 scanning electron microscope manufactured by Hi-Tech Instruments. A scanning voltage of 15 kV and aperture size of 30 µm were used to take the SEM images through the device's secondary electron (SE) detector. A magnification of 2000 × was used for SEM images. Following SEM, EBSD analysis was performed on the samples using a Zeiss Sigma FE-SEM. The detector and camera used to collect the EDS and EBSD data were Apollo X EDAX and Hikari XP EDAX, respectively. The aperture size, scanning step, and acceleration voltage used to collect the data were 120 µm, 0.2 µm, and 15 kV, respectively. The acquired data were analyzed using the TSL software.

For the tensile test, samples were divided into six groups based on their manufacturing and post-processing conditions: horizontal with machined surface, horizontal with machined surface and heat-treated, vertical with machined surface, vertical with machined surface and heat-treated, vertical with raw surface, and vertical with raw surface and heat-treated. The direction of the applied external load in the mechanical tests was perpendicular to the building direction for the horizontal samples, while these directions were parallel for the vertical specimens. In addition, the machined specimens were mechanically processed by removing 0.5 mm of material from the surface in 0.1 mm increments. The machining was performed using a FANUC 18i-TB precision system and a Sandvik Coromant VNMG 331-MF 1115 tool. Finally, the heat-treated samples were post-processed via the same heat treatment procedure introduced earlier. The quasi-static tensile tests were performed with a Galdabini Quasar 600 machine using a strain rate of 0.001 s^−1^ at room temperature (≈ 25 °C). An ARAMIS DIC system was used to record the stress-strain data during the tensile test. A deformation 12M (FG) sensor was calibrated for the DIC technique. Regarding the calibration parameters, the working distance, camera angle, and distance between cameras were 410 mm, 25˚, 142 mm, respectively, resulting in a calibration deviation of 0.037 pixels.

Finally, Charpy tests were performed using a standardized hammer set up at room temperature following ASTM E23. Then, the fracture surface analysis was carried out using a KEYENCE VE-3200 3D measurement microscope. The images were taken using the low-mag camera under 25 × magnification and the Oneshot 3D set up. Subsequently, the visual data were analyzed using VR-3000 G2 software (version 2.5.0.116) and Foxit Reader (version 10.1.3.37598) to calculate the shear fracture areas.

## CRediT Author Statement

**Shahriar Afkhami:** Conceptualization, Methodology, Software, Investigation, Writing - original draft, Writing - review & editing, Visualization; **Vahid Javaheri:** Conceptualization, Methodology, Validation, Writing - original draft, Writing - review & editing; **Edris Dabiri:** Conceptualization, Methodology, Validation, Writing - original draft, Writing - review & editing; **Heidi Piili:** Writing - review & editing, Supervision, Funding acquisition; **Timo Björk:** Conceptualization, Writing - review & editing, Supervision, Funding acquisition.

## Declaration of Competing Interest

The authors declare that they have no known competing financial interests or personal relationships that could have appeared to influence the work reported in this paper.

## Appendix Supplementary materials

Image, application 1
