# Supplementary material for: Data related to the microstructural identification and analyzing the mechanical properties of maraging stainless steel 13Cr10Ni1.7Mo2Al0.4Mn0.4Si (commercially known as CX) processed by laser powder bed fusion method
Source: Data Brief. 2022 Jan 24;41:107856. doi: 10.1016/j.dib.2022.107856 (PMC8814306; doi:10.1016/j.dib.2022.107856)

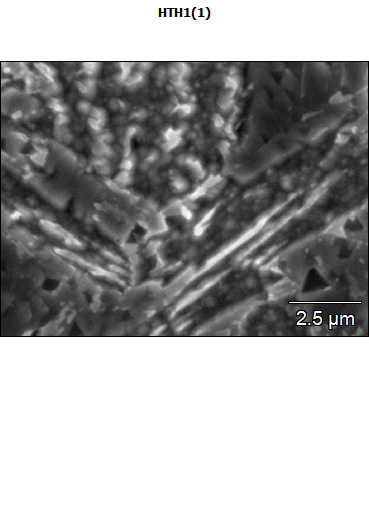


Data Type: Weight %

Image Resolution: 1024 by 768

Image Pixel Size: 0.01 µm

Map Resolution: 256 by 192

Map Pixel Size: 0.05 µm

Acc. Voltage: 15.0 kV

Magnification: 10000


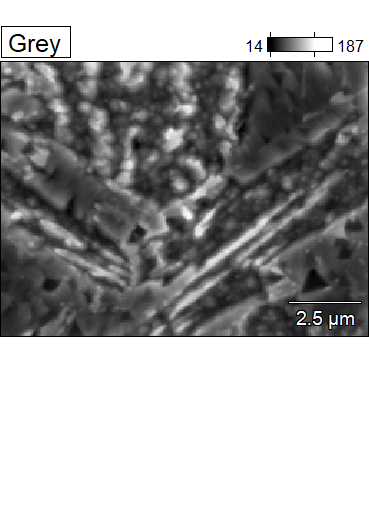

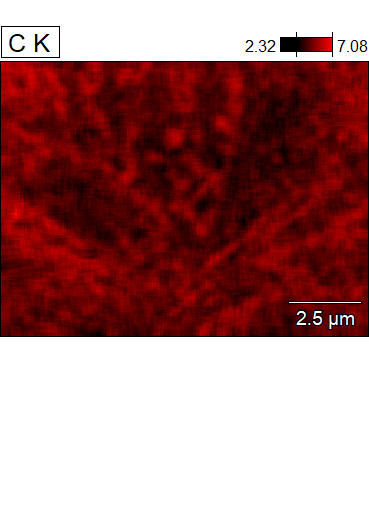


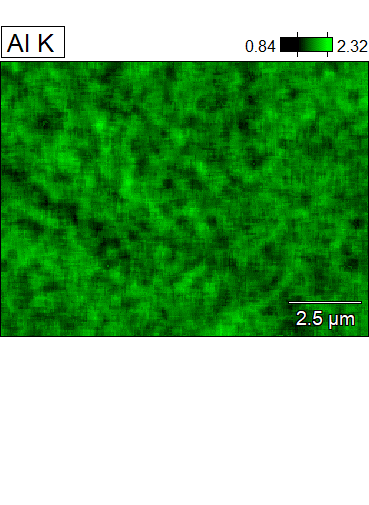

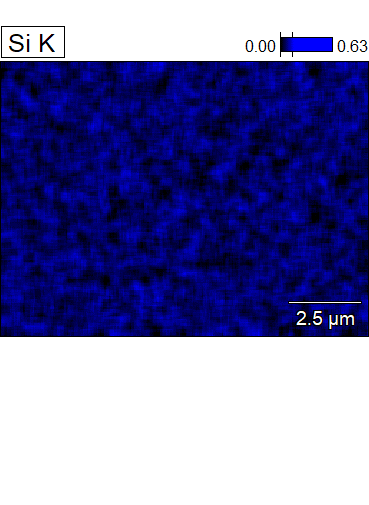


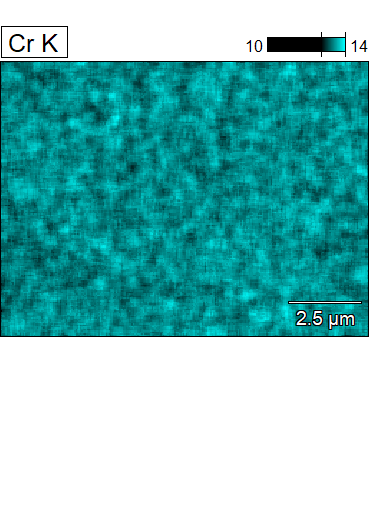

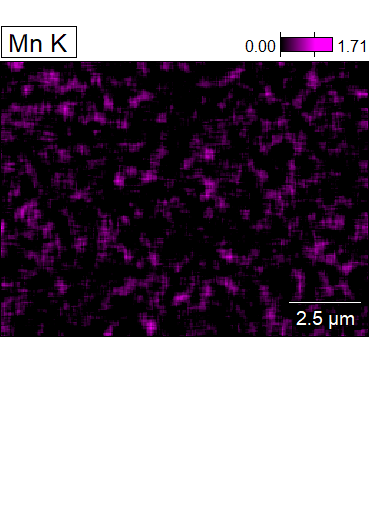


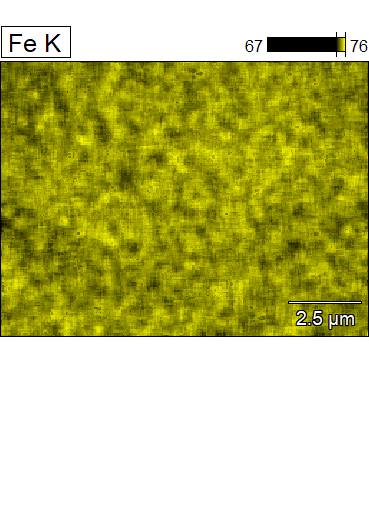

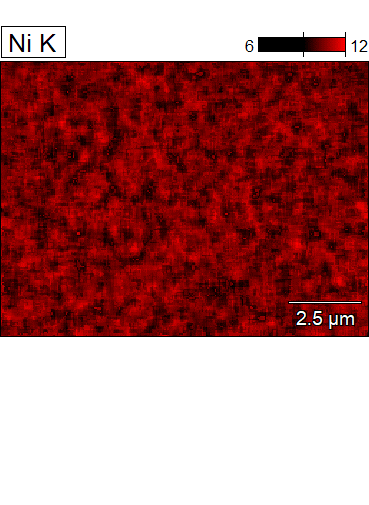


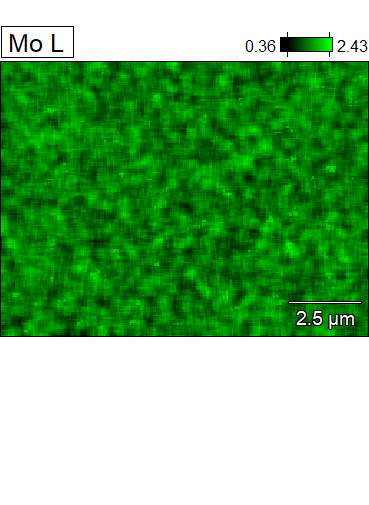

Supplement: Supplementary file 1 [file mmc1.zip › Supplementary material/EDS.docx]
