# Supplementary material for: Data related to the microstructural identification and analyzing the mechanical properties of maraging stainless steel 13Cr10Ni1.7Mo2Al0.4Mn0.4Si (commercially known as CX) processed by laser powder bed fusion method
Source: Data Brief. 2022 Jan 24;41:107856. doi: 10.1016/j.dib.2022.107856 (PMC8814306; doi:10.1016/j.dib.2022.107856)
Supplement: Supplementary file 1 [file mmc1.zip › Supplementary material/Table A.docx]

# **Table A.** Size and shape distribution data associated with the defects in horizontal and vertical specimens with different post-processing conditions.

| **Sample type** | **Defect number** | **Defect area (µm^2^)** | **Defect circularity** |
| --- | --- | --- | --- |
| Horizontal (as-built); building plane | 1 | 12.601 | 0.961 |
| Horizontal (as-built); building plane | 2 | 0.548 | 0.967 |
| Horizontal (as-built); building plane | 3 | 0.183 | 1.000 |
| Horizontal (as-built); building plane | 4 | 0.365 | 1.000 |
| Horizontal (as-built); building plane | 5 | 0.183 | 1.000 |
| Horizontal (as-built); building plane | 6 | 30.864 | 0.924 |
| Horizontal (as-built); building plane | 7 | 0.731 | 1.000 |
| Horizontal (as-built); building plane | 8 | 32.690 | 0.621 |
| Horizontal (as-built); building plane | 9 | 1.644 | 0.923 |
| Horizontal (as-built); building plane | 10 | 248.375 | 0.601 |
| Horizontal (as-built); building plane | 11 | 0.365 | 1.000 |
| Horizontal (as-built); building plane | 12 | 0.183 | 1.000 |
| Horizontal (as-built); building plane | 13 | 3.287 | 0.895 |
| Horizontal (as-built); building plane | 14 | 1.096 | 0.916 |
| Horizontal (as-built); building plane | 15 | 96.793 | 0.326 |
| Horizontal (as-built); building plane | 16 | 0.183 | 1.000 |
| Horizontal (as-built); building plane | 17 | 0.183 | 1.000 |
| Horizontal (as-built); building plane | 18 | 0.365 | 1.000 |
| Horizontal (as-built); building plane | 19 | 0.183 | 1.000 |
| Horizontal (as-built); building plane | 20 | 1.461 | 0.914 |
| Horizontal (as-built); building plane | 21 | 0.183 | 1.000 |
| Horizontal (as-built); building plane | 22 | 6.209 | 0.754 |
| Horizontal (as-built); building plane | 23 | 1.278 | 0.800 |
| Horizontal (as-built); building plane | 24 | 45.840 | 0.760 |
| Horizontal (as-built); building plane | 25 | 69.216 | 0.770 |
| Horizontal (as-built); building plane | 26 | 0.183 | 1.000 |
| Horizontal (as-built); building plane | 27 | 1.644 | 0.799 |
| Horizontal (as-built); building plane | 28 | 0.548 | 0.967 |
| Horizontal (as-built); building plane | 29 | 23.742 | 0.833 |
| Horizontal (as-built); building plane | 30 | 3.287 | 0.808 |
| Horizontal (as-built); building plane | 31 | 0.183 | 1.000 |
| Horizontal (as-built); building plane | 32 | 1.096 | 1.000 |
| Horizontal (as-built); building plane | 33 | 2.739 | 0.674 |
| Horizontal (as-built); building plane | 34 | 0.913 | 1.000 |
| Horizontal (as-built); building plane | 35 | 0.548 | 0.754 |
| Horizontal (as-built); building plane | 36 | 0.365 | 1.000 |
| Horizontal (as-built); building plane | 37 | 1.096 | 0.809 |
| Horizontal (as-built); building plane | 38 | 6.757 | 0.668 |
| Horizontal (as-built); building plane | 39 | 0.183 | 1.000 |
| Horizontal (as-built); building plane | 40 | 0.548 | 1.000 |
| Horizontal (as-built); building plane | 41 | 0.183 | 1.000 |
| Horizontal (as-built); building plane | 42 | 6.027 | 0.527 |
| Horizontal (as-built); building plane | 43 | 8.584 | 0.375 |
| Horizontal (as-built); building plane | 44 | 0.183 | 1.000 |
| Horizontal (as-built); building plane | 45 | 0.183 | 1.000 |
| Horizontal (as-built); building plane | 46 | 0.183 | 1.000 |
| Horizontal (as-built); building plane | 47 | 0.183 | 1.000 |
| Horizontal (as-built); building plane | 48 | 0.183 | 1.000 |
| Horizontal (as-built); building plane | 49 | 0.913 | 1.000 |
| Horizontal (as-built); building plane | 50 | 0.365 | 1.000 |
| Horizontal (as-built); building plane | 51 | 0.183 | 1.000 |
| Horizontal (as-built); building plane | 52 | 0.183 | 1.000 |
| Horizontal (as-built); building plane | 53 | 0.365 | 1.000 |
| Horizontal (as-built); building plane | 54 | 0.183 | 1.000 |
| Horizontal (as-built); building plane | 55 | 0.365 | 1.000 |
| Horizontal (as-built); building plane | 56 | 9.862 | 0.863 |
| Horizontal (as-built); building plane | 57 | 3.105 | 0.609 |
| Horizontal (as-built); building plane | 58 | 0.731 | 1.000 |
| Horizontal (as-built); building plane | 59 | 0.731 | 1.000 |
| Horizontal (as-built); building plane | 60 | 0.365 | 0.785 |
| Horizontal (as-built); building plane | 61 | 204.909 | 0.535 |
| Horizontal (as-built); building plane | 62 | 0.731 | 0.857 |
| Horizontal (as-built); building plane | 63 | 0.183 | 1.000 |
| Horizontal (as-built); building plane | 64 | 4.931 | 0.730 |
| Horizontal (as-built); building plane | 65 | 5.479 | 0.634 |
| Horizontal (as-built); building plane | 66 | 0.731 | 1.000 |
| Horizontal (as-built); building plane | 67 | 0.365 | 1.000 |
| Horizontal (as-built); building plane | 68 | 0.183 | 1.000 |
| Horizontal (as-built); building plane | 69 | 0.183 | 1.000 |
| Horizontal (as-built); building plane | 70 | 0.183 | 1.000 |
| Horizontal (as-built); building plane | 71 | 0.183 | 1.000 |
| Horizontal (as-built); building plane | 72 | 0.731 | 0.857 |
| Horizontal (as-built); building plane | 73 | 3.653 | 0.529 |
| Horizontal (as-built); building plane | 74 | 0.183 | 1.000 |
| Horizontal (as-built); building plane | 75 | 0.365 | 1.000 |
| Horizontal (as-built); building plane | 76 | 0.183 | 1.000 |
| Horizontal (as-built); scanning plane | 1 | 0.177 | 1.000 |
| Horizontal (as-built); scanning plane | 2 | 0.353 | 0.785 |
| Horizontal (as-built); scanning plane | 3 | 0.177 | 1.000 |
| Horizontal (as-built); scanning plane | 4 | 0.177 | 1.000 |
| Horizontal (as-built); scanning plane | 5 | 81.385 | 0.548 |
| Horizontal (as-built); scanning plane | 6 | 0.530 | 1.000 |
| Horizontal (as-built); scanning plane | 7 | 0.177 | 1.000 |
| Horizontal (as-built); scanning plane | 8 | 9.886 | 0.734 |
| Horizontal (as-built); scanning plane | 9 | 0.353 | 1.000 |
| Horizontal (as-built); scanning plane | 10 | 144.587 | 0.687 |
| Horizontal (as-built); scanning plane | 11 | 4.590 | 0.619 |
| Horizontal (as-built); scanning plane | 12 | 0.706 | 1.000 |
| Horizontal (as-built); scanning plane | 13 | 13.594 | 0.629 |
| Horizontal (as-built); scanning plane | 14 | 273.639 | 0.502 |
| Horizontal (as-built); scanning plane | 15 | 1.942 | 0.780 |
| Horizontal (as-built); scanning plane | 16 | 18.360 | 0.782 |
| Horizontal (as-built); scanning plane | 17 | 0.883 | 0.462 |
| Horizontal (as-built); scanning plane | 18 | 4.237 | 0.860 |
| Horizontal (as-built); scanning plane | 19 | 64.438 | 0.696 |
| Horizontal (as-built); scanning plane | 20 | 0.706 | 0.740 |
| Horizontal (as-built); scanning plane | 21 | 0.530 | 0.524 |
| Horizontal (as-built); scanning plane | 22 | 34.602 | 0.885 |
| Horizontal (as-built); scanning plane | 23 | 1.589 | 0.884 |
| Horizontal (as-built); scanning plane | 24 | 58.435 | 0.853 |
| Horizontal (as-built); scanning plane | 25 | 0.530 | 0.967 |
| Horizontal (as-built); scanning plane | 26 | 107.337 | 0.601 |
| Horizontal (as-built); scanning plane | 27 | 0.353 | 1.000 |
| Horizontal (as-built); scanning plane | 28 | 3.178 | 0.868 |
| Horizontal (as-built); scanning plane | 29 | 4.060 | 0.696 |
| Horizontal (as-built); scanning plane | 30 | 6.355 | 0.726 |
| Horizontal (as-built); scanning plane | 31 | 71.852 | 0.777 |
| Horizontal (as-built); scanning plane | 32 | 9.710 | 0.894 |
| Horizontal (as-built); scanning plane | 33 | 1.236 | 1.000 |
| Horizontal (as-built); scanning plane | 34 | 0.530 | 1.000 |
| Horizontal (as-built); scanning plane | 35 | 0.177 | 1.000 |
| Horizontal (as-built); scanning plane | 36 | 1.589 | 0.799 |
| Horizontal (as-built); scanning plane | 37 | 82.092 | 0.683 |
| Horizontal (as-built); scanning plane | 38 | 138.055 | 0.772 |
| Horizontal (as-built); scanning plane | 39 | 5.473 | 0.738 |
| Horizontal (as-built); scanning plane | 40 | 27.540 | 0.832 |
| Horizontal (as-built); scanning plane | 41 | 0.177 | 1.000 |
| Horizontal (as-built); scanning plane | 42 | 13.064 | 0.874 |
| Horizontal (as-built); scanning plane | 43 | 0.353 | 0.785 |
| Horizontal (as-built); scanning plane | 44 | 213.438 | 0.383 |
| Horizontal (as-built); scanning plane | 45 | 88.447 | 0.398 |
| Horizontal (as-built); scanning plane | 46 | 124.815 | 0.805 |
| Horizontal (as-built); scanning plane | 47 | 0.883 | 0.873 |
| Horizontal (as-built); scanning plane | 48 | 70.440 | 0.845 |
| Horizontal (as-built); scanning plane | 49 | 11.299 | 0.857 |
| Horizontal (as-built); scanning plane | 50 | 17.478 | 0.849 |
| Horizontal (as-built); scanning plane | 51 | 18.537 | 0.941 |
| Horizontal (as-built); scanning plane | 52 | 1.589 | 1.000 |
| Horizontal (as-built); scanning plane | 53 | 42.193 | 0.836 |
| Horizontal (as-built); scanning plane | 54 | 8.474 | 0.781 |
| Horizontal (as-built); scanning plane | 55 | 2.295 | 0.846 |
| Horizontal (as-built); scanning plane | 56 | 3.531 | 0.764 |
| Horizontal (as-built); scanning plane | 57 | 5.296 | 0.929 |
| Horizontal (as-built); scanning plane | 58 | 0.177 | 1.000 |
| Horizontal (as-built); scanning plane | 59 | 10.946 | 0.831 |
| Horizontal (as-built); scanning plane | 60 | 16.242 | 0.789 |
| Horizontal (as-built); scanning plane | 61 | 0.177 | 1.000 |
| Horizontal (as-built); scanning plane | 62 | 1.059 | 0.916 |
| Horizontal (as-built); scanning plane | 63 | 9.886 | 0.750 |
| Horizontal (as-built); scanning plane | 64 | 2.825 | 1.000 |
| Horizontal (as-built); scanning plane | 65 | 1.059 | 1.000 |
| Horizontal (as-built); scanning plane | 66 | 0.883 | 1.000 |
| Horizontal (as-built); scanning plane | 67 | 3.178 | 0.629 |
| Horizontal (as-built); scanning plane | 68 | 2.825 | 0.857 |
| Horizontal (as-built); scanning plane | 69 | 103.630 | 0.516 |
| Horizontal (as-built); scanning plane | 70 | 0.353 | 1.000 |
| Horizontal (as-built); scanning plane | 71 | 51.020 | 0.859 |
| Horizontal (as-built); scanning plane | 72 | 0.353 | 1.000 |
| Horizontal (as-built); scanning plane | 73 | 162.771 | 0.748 |
| Horizontal (as-built); scanning plane | 74 | 0.177 | 1.000 |
| Horizontal (as-built); scanning plane | 75 | 90.742 | 0.850 |
| Horizontal (as-built); scanning plane | 76 | 107.514 | 0.775 |
| Horizontal (as-built); scanning plane | 77 | 1.589 | 0.565 |
| Horizontal (as-built); scanning plane | 78 | 7.415 | 0.793 |
| Horizontal (as-built); scanning plane | 79 | 0.177 | 1.000 |
| Horizontal (as-built); scanning plane | 80 | 37.427 | 0.869 |
| Horizontal (as-built); scanning plane | 81 | 13.947 | 0.900 |
| Horizontal (as-built); scanning plane | 82 | 55.257 | 0.820 |
| Horizontal (as-built); scanning plane | 83 | 54.728 | 0.826 |
| Horizontal (as-built); scanning plane | 84 | 0.883 | 1.000 |
| Horizontal (as-built); scanning plane | 85 | 21.361 | 0.796 |
| Horizontal (as-built); scanning plane | 86 | 0.177 | 1.000 |
| Horizontal (as-built); scanning plane | 87 | 2.825 | 0.857 |
| Horizontal (as-built); scanning plane | 88 | 4.414 | 0.583 |
| Horizontal (as-built); scanning plane | 89 | 12.005 | 0.763 |
| Horizontal (as-built); scanning plane | 90 | 90.389 | 0.294 |
| Horizontal (as-built); scanning plane | 91 | 2.118 | 0.851 |
| Horizontal (as-built); scanning plane | 92 | 0.706 | 1.000 |
| Horizontal (as-built); scanning plane | 93 | 9.357 | 0.780 |
| Horizontal (as-built); scanning plane | 94 | 62.672 | 0.879 |
| Horizontal (as-built); scanning plane | 95 | 5.826 | 0.892 |
| Horizontal (as-built); scanning plane | 96 | 0.883 | 0.873 |
| Horizontal (as-built); scanning plane | 97 | 4.943 | 0.867 |
| Horizontal (as-built); scanning plane | 98 | 82.445 | 0.800 |
| Horizontal (as-built); scanning plane | 99 | 3.354 | 1.000 |
| Horizontal (as-built); scanning plane | 100 | 3.178 | 1.000 |
| Horizontal (as-built); scanning plane | 101 | 4.237 | 1.000 |
| Horizontal (as-built); scanning plane | 102 | 10.063 | 0.889 |
| Horizontal (as-built); scanning plane | 103 | 0.177 | 1.000 |
| Horizontal (as-built); scanning plane | 104 | 26.305 | 0.751 |
| Horizontal (as-built); scanning plane | 105 | 23.657 | 0.759 |
| Horizontal (as-built); scanning plane | 106 | 66.026 | 0.472 |
| Horizontal (as-built); scanning plane | 107 | 0.530 | 0.809 |
| Horizontal (as-built); scanning plane | 108 | 0.177 | 1.000 |
| Horizontal (as-built); scanning plane | 109 | 1.059 | 0.686 |
| Horizontal (as-built); scanning plane | 110 | 0.177 | 1.000 |
| Horizontal (as-built); scanning plane | 111 | 0.177 | 1.000 |
| Horizontal (as-built); scanning plane | 112 | 0.706 | 1.000 |
| Horizontal (as-built); scanning plane | 113 | 0.353 | 1.000 |
| Horizontal (as-built); scanning plane | 114 | 76.442 | 0.810 |
| Horizontal (as-built); scanning plane | 115 | 81.915 | 0.825 |
| Horizontal (as-built); scanning plane | 116 | 0.177 | 1.000 |
| Horizontal (as-built); scanning plane | 117 | 0.706 | 1.000 |
| Horizontal (as-built); scanning plane | 118 | 103.277 | 0.792 |
| Horizontal (as-built); scanning plane | 119 | 48.019 | 0.908 |
| Horizontal (as-built); scanning plane | 120 | 6.179 | 0.968 |
| Horizontal (as-built); scanning plane | 121 | 211.496 | 0.699 |
| Horizontal (as-built); scanning plane | 122 | 5.649 | 1.000 |
| Horizontal (as-built); scanning plane | 123 | 81.562 | 0.702 |
| Horizontal (as-built); scanning plane | 124 | 16.771 | 0.919 |
| Horizontal (as-built); scanning plane | 125 | 40.075 | 0.912 |
| Horizontal (as-built); scanning plane | 126 | 65.673 | 0.885 |
| Horizontal (as-built); scanning plane | 127 | 3.531 | 0.764 |
| Horizontal (as-built); scanning plane | 128 | 0.177 | 1.000 |
| Horizontal (as-built); scanning plane | 129 | 0.177 | 1.000 |
| Horizontal (as-built); scanning plane | 130 | 19.949 | 0.690 |
| Horizontal (as-built); scanning plane | 131 | 5.120 | 0.898 |
| Horizontal (as-built); scanning plane | 132 | 46.783 | 0.796 |
| Horizontal (as-built); scanning plane | 133 | 4.060 | 0.964 |
| Horizontal (as-built); scanning plane | 134 | 43.782 | 0.863 |
| Horizontal (as-built); scanning plane | 135 | 13.594 | 0.877 |
| Horizontal (as-built); scanning plane | 136 | 124.108 | 0.718 |
| Horizontal (as-built); scanning plane | 137 | 1.412 | 1.000 |
| Horizontal (as-built); scanning plane | 138 | 13.947 | 0.900 |
| Horizontal (as-built); scanning plane | 139 | 0.706 | 1.000 |
| Horizontal (as-built); scanning plane | 140 | 64.614 | 0.871 |
| Horizontal (as-built); scanning plane | 141 | 136.113 | 0.618 |
| Horizontal (as-built); scanning plane | 142 | 41.487 | 0.664 |
| Horizontal (as-built); scanning plane | 143 | 0.530 | 1.000 |
| Horizontal (as-built); scanning plane | 144 | 11.475 | 0.730 |
| Horizontal (as-built); scanning plane | 145 | 17.478 | 0.927 |
| Horizontal (as-built); scanning plane | 146 | 213.615 | 0.281 |
| Horizontal (as-built); scanning plane | 147 | 0.177 | 1.000 |
| Horizontal (as-built); scanning plane | 148 | 71.146 | 0.732 |
| Horizontal (as-built); scanning plane | 149 | 9.357 | 0.862 |
| Horizontal (as-built); scanning plane | 150 | 21.715 | 0.732 |
| Horizontal (as-built); scanning plane | 151 | 120.578 | 0.790 |
| Horizontal (as-built); scanning plane | 152 | 0.353 | 1.000 |
| Horizontal (as-built); scanning plane | 153 | 1.765 | 0.709 |
| Horizontal (as-built); scanning plane | 154 | 0.177 | 1.000 |
| Horizontal (as-built); scanning plane | 155 | 0.177 | 1.000 |
| Horizontal (as-built); scanning plane | 156 | 57.729 | 0.857 |
| Horizontal (as-built); scanning plane | 157 | 0.177 | 1.000 |
| Horizontal (as-built); scanning plane | 158 | 0.177 | 1.000 |
| Horizontal (as-built); scanning plane | 159 | 0.177 | 1.000 |
| Horizontal (as-built); scanning plane | 160 | 0.353 | 1.000 |
| Horizontal (as-built); scanning plane | 161 | 64.614 | 0.812 |
| Horizontal (as-built); scanning plane | 162 | 0.353 | 1.000 |
| Horizontal (as-built); scanning plane | 163 | 1.059 | 0.555 |
| Horizontal (as-built); scanning plane | 164 | 1.589 | 0.799 |
| Horizontal (as-built); scanning plane | 165 | 73.971 | 0.838 |
| Horizontal (as-built); scanning plane | 166 | 5.826 | 0.892 |
| Horizontal (as-built); scanning plane | 167 | 100.805 | 0.886 |
| Horizontal (as-built); scanning plane | 168 | 0.177 | 1.000 |
| Horizontal (as-built); scanning plane | 169 | 10.416 | 0.749 |
| Horizontal (as-built); scanning plane | 170 | 155.709 | 0.226 |
| Horizontal (as-built); scanning plane | 171 | 0.177 | 1.000 |
| Horizontal (as-built); scanning plane | 172 | 0.530 | 0.643 |
| Horizontal (as-built); scanning plane | 173 | 2.118 | 1.000 |
| Horizontal (as-built); scanning plane | 174 | 0.883 | 0.641 |
| Horizontal (as-built); scanning plane | 175 | 152.002 | 0.762 |
| Horizontal (as-built); scanning plane | 176 | 0.353 | 1.000 |
| Horizontal (as-built); scanning plane | 177 | 88.624 | 0.858 |
| Horizontal (as-built); scanning plane | 178 | 37.074 | 0.743 |
| Horizontal (as-built); scanning plane | 179 | 0.883 | 1.000 |
| Horizontal (as-built); scanning plane | 180 | 4.767 | 0.910 |
| Horizontal (as-built); scanning plane | 181 | 0.177 | 1.000 |
| Horizontal (as-built); scanning plane | 182 | 6.709 | 0.766 |
| Horizontal (as-built); scanning plane | 183 | 57.199 | 0.694 |
| Horizontal (as-built); scanning plane | 184 | 40.781 | 0.857 |
| Horizontal (as-built); scanning plane | 185 | 28.600 | 0.790 |
| Horizontal (as-built); scanning plane | 186 | 14.829 | 0.707 |
| Horizontal (as-built); scanning plane | 187 | 0.177 | 1.000 |
| Horizontal (as-built); scanning plane | 188 | 17.831 | 0.905 |
| Horizontal (as-built); scanning plane | 189 | 0.177 | 1.000 |
| Horizontal (as-built); scanning plane | 190 | 81.915 | 0.827 |
| Horizontal (as-built); scanning plane | 191 | 1.059 | 0.532 |
| Horizontal (as-built); scanning plane | 192 | 6.532 | 0.470 |
| Horizontal (as-built); scanning plane | 193 | 94.096 | 0.738 |
| Horizontal (as-built); scanning plane | 194 | 0.353 | 1.000 |
| Horizontal (as-built); scanning plane | 195 | 1.589 | 1.000 |
| Horizontal (as-built); scanning plane | 196 | 13.241 | 0.825 |
| Horizontal (as-built); scanning plane | 197 | 2.295 | 0.922 |
| Horizontal (as-built); scanning plane | 198 | 6.179 | 0.834 |
| Horizontal (as-built); scanning plane | 199 | 0.177 | 1.000 |
| Horizontal (as-built); scanning plane | 200 | 0.177 | 1.000 |
| Horizontal (as-built); scanning plane | 201 | 0.530 | 0.967 |
| Horizontal (as-built); scanning plane | 202 | 28.423 | 0.889 |
| Horizontal (as-built); scanning plane | 203 | 8.297 | 0.704 |
| Horizontal (as-built); scanning plane | 204 | 0.353 | 1.000 |
| Horizontal (as-built); scanning plane | 205 | 1.059 | 0.484 |
| Horizontal (as-built); scanning plane | 206 | 16.948 | 0.899 |
| Horizontal (as-built); scanning plane | 207 | 20.302 | 0.876 |
| Horizontal (as-built); scanning plane | 208 | 16.771 | 0.745 |
| Horizontal (as-built); scanning plane | 209 | 1.059 | 1.000 |
| Horizontal (as-built); scanning plane | 210 | 0.177 | 1.000 |
| Horizontal (as-built); scanning plane | 211 | 7.415 | 0.793 |
| Horizontal (as-built); scanning plane | 212 | 0.177 | 1.000 |
| Horizontal (as-built); scanning plane | 213 | 0.177 | 1.000 |
| Horizontal (as-built); scanning plane | 214 | 2.295 | 0.646 |
| Horizontal (as-built); scanning plane | 215 | 2.118 | 0.967 |
| Horizontal (as-built); scanning plane | 216 | 2.472 | 0.880 |
| Horizontal (as-built); scanning plane | 217 | 0.530 | 1.000 |
| Horizontal (as-built); scanning plane | 218 | 2.648 | 0.537 |
| Horizontal (as-built); scanning plane | 219 | 48.019 | 0.750 |
| Horizontal (as-built); scanning plane | 220 | 65.497 | 0.793 |
| Horizontal (as-built); scanning plane | 221 | 0.353 | 1.000 |
| Horizontal (as-built); scanning plane | 222 | 0.177 | 1.000 |
| Horizontal (as-built); scanning plane | 223 | 4.237 | 0.348 |
| Horizontal (as-built); scanning plane | 224 | 2.472 | 0.750 |
| Horizontal (as-built); scanning plane | 225 | 45.901 | 0.885 |
| Horizontal (as-built); scanning plane | 226 | 0.177 | 1.000 |
| Horizontal (as-built); scanning plane | 227 | 0.177 | 1.000 |
| Horizontal (as-built); scanning plane | 228 | 0.177 | 1.000 |
| Horizontal (as-built); scanning plane | 229 | 0.177 | 1.000 |
| Horizontal (as-built); scanning plane | 230 | 7.768 | 0.747 |
| Horizontal (as-built); scanning plane | 231 | 0.353 | 1.000 |
| Horizontal (as-built); scanning plane | 232 | 0.177 | 1.000 |
| Horizontal (as-built); scanning plane | 233 | 0.177 | 1.000 |
| Horizontal (as-built); scanning plane | 234 | 0.177 | 1.000 |
| Horizontal (as-built); scanning plane | 235 | 0.353 | 1.000 |
| Horizontal (as-built); scanning plane | 236 | 2.295 | 0.956 |
| Horizontal (as-built); scanning plane | 237 | 26.305 | 0.490 |
| Horizontal (as-built); scanning plane | 238 | 0.177 | 1.000 |
| Horizontal (as-built); scanning plane | 239 | 0.177 | 1.000 |
| Horizontal (as-built); scanning plane | 240 | 0.177 | 1.000 |
| Horizontal (as-built); scanning plane | 241 | 5.649 | 0.520 |
| Horizontal (as-built); scanning plane | 242 | 0.353 | 1.000 |
| Horizontal (as-built); scanning plane | 243 | 0.530 | 0.967 |
| Horizontal (as-built); scanning plane | 244 | 0.177 | 1.000 |
| Horizontal (as-built); scanning plane | 245 | 29.129 | 0.275 |
| Horizontal (as-built); scanning plane | 246 | 3.178 | 0.544 |
| Horizontal (as-built); scanning plane | 247 | 1.765 | 0.887 |
| Horizontal (as-built); scanning plane | 248 | 0.353 | 1.000 |
| Horizontal (as-built); scanning plane | 249 | 13.594 | 0.507 |
| Horizontal (as-built); scanning plane | 250 | 0.177 | 1.000 |
| Horizontal (as-built); scanning plane | 251 | 1.236 | 0.647 |
| Horizontal (as-built); scanning plane | 252 | 1.059 | 0.686 |
| Horizontal (as-built); scanning plane | 253 | 0.883 | 0.572 |
| Horizontal (as-built); scanning plane | 254 | 0.177 | 1.000 |
| Horizontal (as-built); scanning plane | 255 | 2.118 | 0.781 |
| Horizontal (as-built); scanning plane | 256 | 0.177 | 1.000 |
| Horizontal (as-built); scanning plane | 257 | 20.655 | 0.750 |
| Horizontal (as-built); scanning plane | 258 | 5.473 | 0.574 |
| Horizontal (as-built); scanning plane | 259 | 0.353 | 1.000 |
| Horizontal (as-built); scanning plane | 260 | 0.177 | 1.000 |
| Horizontal (as-built); scanning plane | 261 | 1.059 | 0.484 |
| Horizontal (as-built); scanning plane | 262 | 0.883 | 1.000 |
| Horizontal (as-built); scanning plane | 263 | 0.353 | 1.000 |
| Horizontal (as-built); scanning plane | 264 | 140.880 | 0.767 |
| Horizontal (as-built); scanning plane | 265 | 0.177 | 1.000 |
| Horizontal (as-built); scanning plane | 266 | 0.353 | 1.000 |
| Horizontal (as-built); scanning plane | 267 | 0.177 | 1.000 |
| Horizontal (as-built); scanning plane | 268 | 0.177 | 1.000 |
| Horizontal (as-built); scanning plane | 269 | 8.827 | 0.767 |
| Horizontal (as-built); scanning plane | 270 | 0.353 | 1.000 |
| Horizontal (as-built); scanning plane | 271 | 0.177 | 1.000 |
| Horizontal (as-built); scanning plane | 272 | 1.412 | 0.503 |
| Horizontal (as-built); scanning plane | 273 | 0.177 | 1.000 |
| Horizontal (as-built); scanning plane | 274 | 10.946 | 0.414 |
| Horizontal (as-built); scanning plane | 275 | 1.236 | 1.000 |
| Horizontal (as-built); scanning plane | 276 | 0.177 | 1.000 |
| Horizontal (as-built); scanning plane | 277 | 15.712 | 0.393 |
| Horizontal (as-built); scanning plane | 278 | 82.621 | 0.789 |
| Horizontal (as-built); scanning plane | 279 | 0.177 | 1.000 |
| Horizontal (as-built); scanning plane | 280 | 0.353 | 1.000 |
| Horizontal (as-built); scanning plane | 281 | 11.122 | 0.630 |
| Horizontal (as-built); scanning plane | 282 | 0.177 | 1.000 |
| Horizontal (as-built); scanning plane | 283 | 0.353 | 1.000 |
| Horizontal (as-built); scanning plane | 284 | 0.177 | 1.000 |
| Horizontal (as-built); scanning plane | 285 | 0.530 | 1.000 |
| Horizontal (as-built); scanning plane | 286 | 16.595 | 0.247 |
| Horizontal (as-built); scanning plane | 287 | 0.353 | 1.000 |
| Horizontal (as-built); scanning plane | 288 | 0.530 | 1.000 |
| Horizontal (as-built); scanning plane | 289 | 0.530 | 0.754 |
| Horizontal (as-built); scanning plane | 290 | 0.177 | 1.000 |
| Horizontal (as-built); scanning plane | 291 | 1.942 | 0.420 |
| Horizontal (as-built); scanning plane | 292 | 9.357 | 0.649 |
| Horizontal (as-built); scanning plane | 293 | 0.177 | 1.000 |
| Horizontal (as-built); scanning plane | 294 | 0.883 | 1.000 |
| Horizontal (as-built); scanning plane | 295 | 0.177 | 1.000 |
| Horizontal (as-built); scanning plane | 296 | 0.177 | 1.000 |
| Horizontal (as-built); scanning plane | 297 | 0.177 | 1.000 |
| Horizontal (as-built); scanning plane | 298 | 0.883 | 0.873 |
| Horizontal (as-built); scanning plane | 299 | 513.911 | 0.347 |
| Horizontal (as-built); scanning plane | 300 | 1.942 | 0.384 |
| Horizontal (as-built); scanning plane | 301 | 0.177 | 1.000 |
| Horizontal (as-built); scanning plane | 302 | 0.177 | 1.000 |
| Horizontal (as-built); scanning plane | 303 | 0.353 | 1.000 |
| Horizontal (as-built); scanning plane | 304 | 231.269 | 0.330 |
| Horizontal (as-built); scanning plane | 305 | 0.177 | 1.000 |
| Horizontal (as-built); scanning plane | 306 | 1.236 | 0.687 |
| Horizontal (as-built); scanning plane | 307 | 0.706 | 0.857 |
| Horizontal (as-built); scanning plane | 308 | 0.177 | 1.000 |
| Horizontal (as-built); scanning plane | 309 | 56.670 | 0.855 |
| Horizontal (as-built); scanning plane | 310 | 0.353 | 1.000 |
| Horizontal (as-built); scanning plane | 311 | 0.353 | 1.000 |
| Horizontal (as-built); scanning plane | 312 | 0.177 | 1.000 |
| Horizontal (as-built); scanning plane | 313 | 2.295 | 0.846 |
| Horizontal (as-built); scanning plane | 314 | 0.706 | 1.000 |
| Horizontal (as-built); scanning plane | 315 | 0.177 | 1.000 |
| Horizontal (as-built); scanning plane | 316 | 0.177 | 1.000 |
| Horizontal (as-built); scanning plane | 317 | 0.177 | 1.000 |
| Horizontal (as-built); scanning plane | 318 | 0.177 | 1.000 |
| Horizontal (as-built); scanning plane | 319 | 0.177 | 1.000 |
| Horizontal (as-built); scanning plane | 320 | 0.706 | 0.857 |
| Horizontal (as-built); scanning plane | 321 | 430.761 | 0.718 |
| Horizontal (as-built); scanning plane | 322 | 3.001 | 0.820 |
| Horizontal (as-built); scanning plane | 323 | 0.353 | 1.000 |
| Horizontal (as-built); scanning plane | 324 | 0.353 | 1.000 |
| Horizontal (as-built); scanning plane | 325 | 0.353 | 1.000 |
| Horizontal (as-built); scanning plane | 326 | 0.706 | 1.000 |
| Horizontal (as-built); scanning plane | 327 | 0.530 | 0.643 |
| Horizontal (as-built); scanning plane | 328 | 12.181 | 0.638 |
| Horizontal (as-built); scanning plane | 329 | 0.706 | 1.000 |
| Horizontal (as-built); scanning plane | 330 | 2.825 | 0.671 |
| Horizontal (as-built); scanning plane | 331 | 6.355 | 0.815 |
| Horizontal (as-built); scanning plane | 332 | 0.353 | 1.000 |
| Horizontal (as-built); scanning plane | 333 | 0.883 | 0.572 |
| Horizontal (as-built); scanning plane | 334 | 121.990 | 0.583 |
| Horizontal (as-built); scanning plane | 335 | 0.706 | 1.000 |
| Horizontal (as-built); scanning plane | 336 | 119.165 | 0.513 |
| Horizontal (as-built); scanning plane | 337 | 0.177 | 1.000 |
| Horizontal (as-built); scanning plane | 338 | 0.177 | 1.000 |
| Horizontal (as-built); scanning plane | 339 | 0.706 | 0.539 |
| Horizontal (as-built); scanning plane | 340 | 3.707 | 0.943 |
| Horizontal (as-built); scanning plane | 341 | 0.530 | 1.000 |
| Horizontal (as-built); scanning plane | 342 | 0.353 | 1.000 |
| Horizontal (as-built); scanning plane | 343 | 1.059 | 0.916 |
| Horizontal (as-built); scanning plane | 344 | 0.530 | 1.000 |
| Horizontal (as-built); scanning plane | 345 | 0.706 | 0.645 |
| Horizontal (as-built); scanning plane | 346 | 0.177 | 1.000 |
| Horizontal (as-built); scanning plane | 347 | 0.177 | 1.000 |
| Horizontal (as-built); scanning plane | 348 | 16.418 | 0.793 |
| Horizontal (as-built); scanning plane | 349 | 5.473 | 0.738 |
| Horizontal (as-built); scanning plane | 350 | 0.530 | 1.000 |
| Horizontal (as-built); scanning plane | 351 | 0.177 | 1.000 |
| Horizontal (as-built); scanning plane | 352 | 0.530 | 1.000 |
| Horizontal (as-built); scanning plane | 353 | 1.059 | 1.000 |
| Horizontal (as-built); scanning plane | 354 | 0.353 | 1.000 |
| Horizontal (as-built); scanning plane | 355 | 1.942 | 0.547 |
| Horizontal (as-built); scanning plane | 356 | 0.177 | 1.000 |
| Horizontal (as-built); scanning plane | 357 | 0.177 | 1.000 |
| Horizontal (as-built); scanning plane | 358 | 2.118 | 0.343 |
| Horizontal (as-built); scanning plane | 359 | 1.589 | 1.000 |
| Horizontal (as-built); scanning plane | 360 | 0.706 | 0.857 |
| Horizontal (as-built); scanning plane | 361 | 1.942 | 1.000 |
| Horizontal (heat-treated); building plane | 1 | 1.278 | 0.515 |
| Horizontal (heat-treated); building plane | 2 | 5.114 | 0.740 |
| Horizontal (heat-treated); building plane | 3 | 0.183 | 1.000 |
| Horizontal (heat-treated); building plane | 4 | 0.183 | 1.000 |
| Horizontal (heat-treated); building plane | 5 | 206.735 | 0.176 |
| Horizontal (heat-treated); building plane | 6 | 0.183 | 1.000 |
| Horizontal (heat-treated); building plane | 7 | 0.183 | 1.000 |
| Horizontal (heat-treated); building plane | 8 | 0.183 | 1.000 |
| Horizontal (heat-treated); building plane | 9 | 0.183 | 1.000 |
| Horizontal (heat-treated); building plane | 10 | 0.913 | 0.764 |
| Horizontal (heat-treated); building plane | 11 | 6.027 | 0.763 |
| Horizontal (heat-treated); building plane | 12 | 0.183 | 1.000 |
| Horizontal (heat-treated); building plane | 13 | 0.548 | 1.000 |
| Horizontal (heat-treated); building plane | 14 | 9.497 | 0.831 |
| Horizontal (heat-treated); building plane | 15 | 4.018 | 1.000 |
| Horizontal (heat-treated); building plane | 16 | 2.922 | 0.573 |
| Horizontal (heat-treated); building plane | 17 | 20.089 | 0.862 |
| Horizontal (heat-treated); building plane | 18 | 0.365 | 1.000 |
| Horizontal (heat-treated); building plane | 19 | 22.829 | 0.658 |
| Horizontal (heat-treated); building plane | 20 | 18.445 | 0.665 |
| Horizontal (heat-treated); building plane | 21 | 6.392 | 0.816 |
| Horizontal (heat-treated); building plane | 22 | 0.365 | 1.000 |
| Horizontal (heat-treated); building plane | 23 | 2.374 | 1.000 |
| Horizontal (heat-treated); building plane | 24 | 3.653 | 0.815 |
| Horizontal (heat-treated); building plane | 25 | 0.365 | 1.000 |
| Horizontal (heat-treated); building plane | 26 | 0.183 | 1.000 |
| Horizontal (heat-treated); building plane | 27 | 0.731 | 1.000 |
| Horizontal (heat-treated); building plane | 28 | 11.140 | 0.898 |
| Horizontal (heat-treated); building plane | 29 | 0.548 | 1.000 |
| Horizontal (heat-treated); building plane | 30 | 10.775 | 0.883 |
| Horizontal (heat-treated); building plane | 31 | 1.461 | 0.820 |
| Horizontal (heat-treated); building plane | 32 | 1.461 | 1.000 |
| Horizontal (heat-treated); building plane | 33 | 0.183 | 1.000 |
| Horizontal (heat-treated); building plane | 34 | 367.083 | 0.165 |
| Horizontal (heat-treated); building plane | 35 | 0.183 | 1.000 |
| Horizontal (heat-treated); building plane | 36 | 0.365 | 1.000 |
| Horizontal (heat-treated); building plane | 37 | 0.365 | 1.000 |
| Horizontal (heat-treated); building plane | 38 | 0.183 | 1.000 |
| Horizontal (heat-treated); building plane | 39 | 2.739 | 0.629 |
| Horizontal (heat-treated); building plane | 40 | 0.183 | 1.000 |
| Horizontal (heat-treated); building plane | 41 | 1.644 | 0.884 |
| Horizontal (heat-treated); building plane | 42 | 0.913 | 0.674 |
| Horizontal (heat-treated); building plane | 43 | 2.009 | 0.976 |
| Horizontal (heat-treated); building plane | 44 | 7.670 | 0.713 |
| Horizontal (heat-treated); building plane | 45 | 0.183 | 1.000 |
| Horizontal (heat-treated); building plane | 46 | 0.365 | 1.000 |
| Horizontal (heat-treated); building plane | 47 | 0.548 | 1.000 |
| Horizontal (heat-treated); building plane | 48 | 2.922 | 0.719 |
| Horizontal (heat-treated); building plane | 49 | 0.183 | 1.000 |
| Horizontal (heat-treated); building plane | 50 | 0.548 | 1.000 |
| Horizontal (heat-treated); building plane | 51 | 4.748 | 0.666 |
| Horizontal (heat-treated); building plane | 52 | 0.913 | 1.000 |
| Horizontal (heat-treated); building plane | 53 | 0.365 | 1.000 |
| Horizontal (heat-treated); building plane | 54 | 1.826 | 1.000 |
| Horizontal (heat-treated); building plane | 55 | 0.365 | 1.000 |
| Horizontal (heat-treated); building plane | 56 | 4.200 | 0.486 |
| Horizontal (heat-treated); building plane | 57 | 1.644 | 0.923 |
| Horizontal (heat-treated); building plane | 58 | 8.401 | 0.616 |
| Horizontal (heat-treated); building plane | 59 | 0.183 | 1.000 |
| Horizontal (heat-treated); building plane | 60 | 0.731 | 0.698 |
| Horizontal (heat-treated); building plane | 61 | 2.557 | 0.629 |
| Horizontal (heat-treated); building plane | 62 | 0.183 | 1.000 |
| Horizontal (heat-treated); building plane | 63 | 0.913 | 0.513 |
| Horizontal (heat-treated); building plane | 64 | 0.731 | 0.857 |
| Horizontal (heat-treated); building plane | 65 | 0.183 | 1.000 |
| Horizontal (heat-treated); building plane | 66 | 0.183 | 1.000 |
| Horizontal (heat-treated); building plane | 67 | 19.541 | 0.565 |
| Horizontal (heat-treated); building plane | 68 | 0.183 | 1.000 |
| Horizontal (heat-treated); building plane | 69 | 0.183 | 1.000 |
| Horizontal (heat-treated); building plane | 70 | 8.401 | 0.641 |
| Horizontal (heat-treated); building plane | 71 | 1.278 | 0.943 |
| Horizontal (heat-treated); building plane | 72 | 0.183 | 1.000 |
| Horizontal (heat-treated); building plane | 73 | 0.183 | 1.000 |
| Horizontal (heat-treated); building plane | 74 | 0.365 | 1.000 |
| Horizontal (heat-treated); building plane | 75 | 0.183 | 1.000 |
| Horizontal (heat-treated); building plane | 76 | 0.183 | 1.000 |
| Horizontal (heat-treated); building plane | 77 | 0.183 | 1.000 |
| Horizontal (heat-treated); building plane | 78 | 1.644 | 0.521 |
| Horizontal (heat-treated); building plane | 79 | 0.183 | 1.000 |
| Horizontal (heat-treated); building plane | 80 | 0.183 | 1.000 |
| Horizontal (heat-treated); building plane | 81 | 0.183 | 1.000 |
| Horizontal (heat-treated); building plane | 82 | 0.183 | 1.000 |
| Horizontal (heat-treated); building plane | 83 | 0.731 | 1.000 |
| Horizontal (heat-treated); building plane | 84 | 0.365 | 1.000 |
| Horizontal (heat-treated); building plane | 85 | 0.913 | 0.674 |
| Horizontal (heat-treated); building plane | 86 | 4.566 | 0.451 |
| Horizontal (heat-treated); building plane | 87 | 0.183 | 1.000 |
| Horizontal (heat-treated); building plane | 88 | 0.183 | 1.000 |
| Horizontal (heat-treated); building plane | 89 | 0.183 | 1.000 |
| Horizontal (heat-treated); building plane | 90 | 0.183 | 1.000 |
| Horizontal (heat-treated); building plane | 91 | 0.913 | 0.873 |
| Horizontal (heat-treated); building plane | 92 | 0.183 | 1.000 |
| Horizontal (heat-treated); building plane | 93 | 0.365 | 1.000 |
| Horizontal (heat-treated); building plane | 94 | 0.913 | 0.764 |
| Horizontal (heat-treated); building plane | 95 | 0.183 | 1.000 |
| Horizontal (heat-treated); building plane | 96 | 0.183 | 1.000 |
| Horizontal (heat-treated); building plane | 97 | 0.365 | 1.000 |
| Horizontal (heat-treated); building plane | 98 | 0.183 | 1.000 |
| Horizontal (heat-treated); building plane | 99 | 0.183 | 1.000 |
| Horizontal (heat-treated); building plane | 100 | 0.183 | 1.000 |
| Horizontal (heat-treated); building plane | 101 | 0.365 | 1.000 |
| Horizontal (heat-treated); building plane | 102 | 1.278 | 0.943 |
| Horizontal (heat-treated); building plane | 103 | 0.365 | 1.000 |
| Horizontal (heat-treated); building plane | 104 | 1.096 | 0.686 |
| Horizontal (heat-treated); building plane | 105 | 0.183 | 1.000 |
| Horizontal (heat-treated); building plane | 106 | 0.548 | 1.000 |
| Horizontal (heat-treated); building plane | 107 | 0.183 | 1.000 |
| Horizontal (heat-treated); building plane | 108 | 0.183 | 1.000 |
| Horizontal (heat-treated); building plane | 109 | 0.365 | 0.785 |
| Horizontal (heat-treated); building plane | 110 | 29.586 | 0.543 |
| Horizontal (heat-treated); building plane | 111 | 0.183 | 1.000 |
| Horizontal (heat-treated); building plane | 112 | 0.548 | 1.000 |
| Horizontal (heat-treated); building plane | 113 | 4.566 | 0.627 |
| Horizontal (heat-treated); building plane | 114 | 0.731 | 1.000 |
| Horizontal (heat-treated); building plane | 115 | 3.835 | 0.943 |
| Horizontal (heat-treated); building plane | 116 | 0.183 | 1.000 |
| Horizontal (heat-treated); building plane | 117 | 0.183 | 1.000 |
| Horizontal (heat-treated); building plane | 118 | 0.183 | 1.000 |
| Horizontal (heat-treated); building plane | 119 | 0.183 | 1.000 |
| Horizontal (heat-treated); building plane | 120 | 0.183 | 1.000 |
| Horizontal (heat-treated); building plane | 121 | 0.183 | 1.000 |
| Horizontal (heat-treated); building plane | 122 | 0.183 | 1.000 |
| Horizontal (heat-treated); building plane | 123 | 0.183 | 1.000 |
| Horizontal (heat-treated); building plane | 124 | 1.096 | 0.769 |
| Horizontal (heat-treated); building plane | 125 | 25.933 | 0.316 |
| Horizontal (heat-treated); building plane | 126 | 1.461 | 0.914 |
| Horizontal (heat-treated); building plane | 127 | 0.183 | 1.000 |
| Horizontal (heat-treated); building plane | 128 | 0.365 | 0.785 |
| Horizontal (heat-treated); building plane | 129 | 2.009 | 0.715 |
| Horizontal (heat-treated); building plane | 130 | 0.183 | 1.000 |
| Horizontal (heat-treated); building plane | 131 | 0.183 | 1.000 |
| Horizontal (heat-treated); building plane | 132 | 0.183 | 1.000 |
| Horizontal (heat-treated); building plane | 133 | 0.183 | 1.000 |
| Horizontal (heat-treated); building plane | 134 | 0.183 | 1.000 |
| Horizontal (heat-treated); building plane | 135 | 0.365 | 1.000 |
| Horizontal (heat-treated); building plane | 136 | 0.183 | 1.000 |
| Horizontal (heat-treated); building plane | 137 | 0.183 | 1.000 |
| Horizontal (heat-treated); building plane | 138 | 0.183 | 1.000 |
| Horizontal (heat-treated); building plane | 139 | 0.365 | 1.000 |
| Horizontal (heat-treated); building plane | 140 | 0.913 | 1.000 |
| Horizontal (heat-treated); building plane | 141 | 0.183 | 1.000 |
| Horizontal (heat-treated); building plane | 142 | 4.566 | 0.555 |
| Horizontal (heat-treated); building plane | 143 | 1.096 | 0.809 |
| Horizontal (heat-treated); building plane | 144 | 4.931 | 0.657 |
| Horizontal (heat-treated); building plane | 145 | 0.183 | 1.000 |
| Horizontal (heat-treated); building plane | 146 | 0.183 | 1.000 |
| Horizontal (heat-treated); building plane | 147 | 1.278 | 0.687 |
| Horizontal (heat-treated); building plane | 148 | 0.183 | 1.000 |
| Horizontal (heat-treated); building plane | 149 | 0.183 | 1.000 |
| Horizontal (heat-treated); building plane | 150 | 3.105 | 0.594 |
| Horizontal (heat-treated); building plane | 151 | 0.183 | 1.000 |
| Horizontal (heat-treated); building plane | 152 | 0.365 | 1.000 |
| Horizontal (heat-treated); building plane | 153 | 0.365 | 1.000 |
| Horizontal (heat-treated); building plane | 154 | 0.365 | 0.785 |
| Horizontal (heat-treated); building plane | 155 | 0.365 | 1.000 |
| Horizontal (heat-treated); building plane | 156 | 0.365 | 1.000 |
| Horizontal (heat-treated); building plane | 157 | 0.183 | 1.000 |
| Horizontal (heat-treated); building plane | 158 | 0.183 | 1.000 |
| Horizontal (heat-treated); building plane | 159 | 0.183 | 1.000 |
| Horizontal (heat-treated); building plane | 160 | 0.183 | 1.000 |
| Horizontal (heat-treated); building plane | 161 | 0.183 | 1.000 |
| Horizontal (heat-treated); building plane | 162 | 0.183 | 1.000 |
| Horizontal (heat-treated); scanning plane | 1 | 0.183 | 1.000 |
| Horizontal (heat-treated); scanning plane | 2 | 25.568 | 0.921 |
| Horizontal (heat-treated); scanning plane | 3 | 131.492 | 0.529 |
| Horizontal (heat-treated); scanning plane | 4 | 0.365 | 1.000 |
| Horizontal (heat-treated); scanning plane | 5 | 6.575 | 0.974 |
| Horizontal (heat-treated); scanning plane | 6 | 144.459 | 0.394 |
| Horizontal (heat-treated); scanning plane | 7 | 0.183 | 1.000 |
| Horizontal (heat-treated); scanning plane | 8 | 3.653 | 1.000 |
| Horizontal (heat-treated); scanning plane | 9 | 1.644 | 1.000 |
| Horizontal (heat-treated); scanning plane | 10 | 65.381 | 0.761 |
| Horizontal (heat-treated); scanning plane | 11 | 62.642 | 0.549 |
| Horizontal (heat-treated); scanning plane | 12 | 4.200 | 0.642 |
| Horizontal (heat-treated); scanning plane | 13 | 0.183 | 1.000 |
| Horizontal (heat-treated); scanning plane | 14 | 55.336 | 0.623 |
| Horizontal (heat-treated); scanning plane | 15 | 31.412 | 0.581 |
| Horizontal (heat-treated); scanning plane | 16 | 0.183 | 1.000 |
| Horizontal (heat-treated); scanning plane | 17 | 0.183 | 1.000 |
| Horizontal (heat-treated); scanning plane | 18 | 51.501 | 0.691 |
| Horizontal (heat-treated); scanning plane | 19 | 14.610 | 0.979 |
| Horizontal (heat-treated); scanning plane | 20 | 0.365 | 1.000 |
| Horizontal (heat-treated); scanning plane | 21 | 27.212 | 0.795 |
| Horizontal (heat-treated); scanning plane | 22 | 71.408 | 0.602 |
| Horizontal (heat-treated); scanning plane | 23 | 0.183 | 1.000 |
| Horizontal (heat-treated); scanning plane | 24 | 0.183 | 1.000 |
| Horizontal (heat-treated); scanning plane | 25 | 0.365 | 1.000 |
| Horizontal (heat-treated); scanning plane | 26 | 0.183 | 1.000 |
| Horizontal (heat-treated); scanning plane | 27 | 36.526 | 0.681 |
| Horizontal (heat-treated); scanning plane | 28 | 3.105 | 1.000 |
| Horizontal (heat-treated); scanning plane | 29 | 53.327 | 0.853 |
| Horizontal (heat-treated); scanning plane | 30 | 3.835 | 1.000 |
| Horizontal (heat-treated); scanning plane | 31 | 6.027 | 0.747 |
| Horizontal (heat-treated); scanning plane | 32 | 51.501 | 0.783 |
| Horizontal (heat-treated); scanning plane | 33 | 1.826 | 1.000 |
| Horizontal (heat-treated); scanning plane | 34 | 74.695 | 0.592 |
| Horizontal (heat-treated); scanning plane | 35 | 65.746 | 0.806 |
| Horizontal (heat-treated); scanning plane | 36 | 59.172 | 0.654 |
| Horizontal (heat-treated); scanning plane | 37 | 18.628 | 0.926 |
| Horizontal (heat-treated); scanning plane | 38 | 226.642 | 0.482 |
| Horizontal (heat-treated); scanning plane | 39 | 18.993 | 0.692 |
| Horizontal (heat-treated); scanning plane | 40 | 1.096 | 1.000 |
| Horizontal (heat-treated); scanning plane | 41 | 1.826 | 1.000 |
| Horizontal (heat-treated); scanning plane | 42 | 37.256 | 0.855 |
| Horizontal (heat-treated); scanning plane | 43 | 29.768 | 0.795 |
| Horizontal (heat-treated); scanning plane | 44 | 43.466 | 0.819 |
| Horizontal (heat-treated); scanning plane | 45 | 109.029 | 0.315 |
| Horizontal (heat-treated); scanning plane | 46 | 81.087 | 0.592 |
| Horizontal (heat-treated); scanning plane | 47 | 36.343 | 0.898 |
| Horizontal (heat-treated); scanning plane | 48 | 1.278 | 1.000 |
| Horizontal (heat-treated); scanning plane | 49 | 0.183 | 1.000 |
| Horizontal (heat-treated); scanning plane | 50 | 0.183 | 1.000 |
| Horizontal (heat-treated); scanning plane | 51 | 11.871 | 1.000 |
| Horizontal (heat-treated); scanning plane | 52 | 9.314 | 0.865 |
| Horizontal (heat-treated); scanning plane | 53 | 5.479 | 0.877 |
| Horizontal (heat-treated); scanning plane | 54 | 4.931 | 0.730 |
| Horizontal (heat-treated); scanning plane | 55 | 26.481 | 0.929 |
| Horizontal (heat-treated); scanning plane | 56 | 17.532 | 0.860 |
| Horizontal (heat-treated); scanning plane | 57 | 13.149 | 0.943 |
| Horizontal (heat-treated); scanning plane | 58 | 88.027 | 0.344 |
| Horizontal (heat-treated); scanning plane | 59 | 55.884 | 0.747 |
| Horizontal (heat-treated); scanning plane | 60 | 66.659 | 0.741 |
| Horizontal (heat-treated); scanning plane | 61 | 27.577 | 0.834 |
| Horizontal (heat-treated); scanning plane | 62 | 20.089 | 0.662 |
| Horizontal (heat-treated); scanning plane | 63 | 0.183 | 1.000 |
| Horizontal (heat-treated); scanning plane | 64 | 35.978 | 0.926 |
| Horizontal (heat-treated); scanning plane | 65 | 73.782 | 0.845 |
| Horizontal (heat-treated); scanning plane | 66 | 92.958 | 0.372 |
| Horizontal (heat-treated); scanning plane | 67 | 20.089 | 0.818 |
| Horizontal (heat-treated); scanning plane | 68 | 0.183 | 1.000 |
| Horizontal (heat-treated); scanning plane | 69 | 1.461 | 0.415 |
| Horizontal (heat-treated); scanning plane | 70 | 59.537 | 0.807 |
| Horizontal (heat-treated); scanning plane | 71 | 27.942 | 0.857 |
| Horizontal (heat-treated); scanning plane | 72 | 50.223 | 0.839 |
| Horizontal (heat-treated); scanning plane | 73 | 9.497 | 0.736 |
| Horizontal (heat-treated); scanning plane | 74 | 11.323 | 0.686 |
| Horizontal (heat-treated); scanning plane | 75 | 100.446 | 0.671 |
| Horizontal (heat-treated); scanning plane | 76 | 0.183 | 1.000 |
| Horizontal (heat-treated); scanning plane | 77 | 0.183 | 1.000 |
| Horizontal (heat-treated); scanning plane | 78 | 0.183 | 1.000 |
| Horizontal (heat-treated); scanning plane | 79 | 94.784 | 0.742 |
| Horizontal (heat-treated); scanning plane | 80 | 1.461 | 0.914 |
| Horizontal (heat-treated); scanning plane | 81 | 0.183 | 1.000 |
| Horizontal (heat-treated); scanning plane | 82 | 0.183 | 1.000 |
| Horizontal (heat-treated); scanning plane | 83 | 69.216 | 0.631 |
| Horizontal (heat-treated); scanning plane | 84 | 0.183 | 1.000 |
| Horizontal (heat-treated); scanning plane | 85 | 53.145 | 0.536 |
| Horizontal (heat-treated); scanning plane | 86 | 0.365 | 1.000 |
| Horizontal (heat-treated); scanning plane | 87 | 0.183 | 1.000 |
| Horizontal (heat-treated); scanning plane | 88 | 46.570 | 0.575 |
| Horizontal (heat-treated); scanning plane | 89 | 31.777 | 0.768 |
| Horizontal (heat-treated); scanning plane | 90 | 0.183 | 1.000 |
| Horizontal (heat-treated); scanning plane | 91 | 10.410 | 0.874 |
| Horizontal (heat-treated); scanning plane | 92 | 75.791 | 0.668 |
| Horizontal (heat-treated); scanning plane | 93 | 77.617 | 0.564 |
| Horizontal (heat-treated); scanning plane | 94 | 81.270 | 0.616 |
| Horizontal (heat-treated); scanning plane | 95 | 3.105 | 0.713 |
| Horizontal (heat-treated); scanning plane | 96 | 0.365 | 1.000 |
| Horizontal (heat-treated); scanning plane | 97 | 20.454 | 0.904 |
| Horizontal (heat-treated); scanning plane | 98 | 0.365 | 1.000 |
| Horizontal (heat-treated); scanning plane | 99 | 25.751 | 0.790 |
| Horizontal (heat-treated); scanning plane | 100 | 4.566 | 0.627 |
| Horizontal (heat-treated); scanning plane | 101 | 7.488 | 0.655 |
| Horizontal (heat-treated); scanning plane | 102 | 0.183 | 1.000 |
| Horizontal (heat-treated); scanning plane | 103 | 42.370 | 0.851 |
| Horizontal (heat-treated); scanning plane | 104 | 16.254 | 0.710 |
| Horizontal (heat-treated); scanning plane | 105 | 3.287 | 1.000 |
| Horizontal (heat-treated); scanning plane | 106 | 0.913 | 0.764 |
| Horizontal (heat-treated); scanning plane | 107 | 0.183 | 1.000 |
| Horizontal (heat-treated); scanning plane | 108 | 9.862 | 0.247 |
| Horizontal (heat-treated); scanning plane | 109 | 0.731 | 0.698 |
| Horizontal (heat-treated); scanning plane | 110 | 367.448 | 0.246 |
| Horizontal (heat-treated); scanning plane | 111 | 0.183 | 1.000 |
| Horizontal (heat-treated); scanning plane | 112 | 0.183 | 1.000 |
| Horizontal (heat-treated); scanning plane | 113 | 0.183 | 1.000 |
| Horizontal (heat-treated); scanning plane | 114 | 1.096 | 1.000 |
| Horizontal (heat-treated); scanning plane | 115 | 0.365 | 1.000 |
| Horizontal (heat-treated); scanning plane | 116 | 0.731 | 0.857 |
| Horizontal (heat-treated); scanning plane | 117 | 2.009 | 0.976 |
| Horizontal (heat-treated); scanning plane | 118 | 0.183 | 1.000 |
| Horizontal (heat-treated); scanning plane | 119 | 0.183 | 1.000 |
| Horizontal (heat-treated); scanning plane | 120 | 0.183 | 1.000 |
| Horizontal (heat-treated); scanning plane | 121 | 0.183 | 1.000 |
| Horizontal (heat-treated); scanning plane | 122 | 0.183 | 1.000 |
| Horizontal (heat-treated); scanning plane | 123 | 0.365 | 1.000 |
| Horizontal (heat-treated); scanning plane | 124 | 0.548 | 0.967 |
| Horizontal (heat-treated); scanning plane | 125 | 27.212 | 0.542 |
| Horizontal (heat-treated); scanning plane | 126 | 0.183 | 1.000 |
| Horizontal (heat-treated); scanning plane | 127 | 51.319 | 0.760 |
| Horizontal (heat-treated); scanning plane | 128 | 0.365 | 1.000 |
| Horizontal (heat-treated); scanning plane | 129 | 0.183 | 1.000 |
| Horizontal (heat-treated); scanning plane | 130 | 0.365 | 1.000 |
| Horizontal (heat-treated); scanning plane | 131 | 2.009 | 0.530 |
| Horizontal (heat-treated); scanning plane | 132 | 0.183 | 1.000 |
| Horizontal (heat-treated); scanning plane | 133 | 0.183 | 1.000 |
| Horizontal (heat-treated); scanning plane | 134 | 4.931 | 0.714 |
| Horizontal (heat-treated); scanning plane | 135 | 0.183 | 1.000 |
| Horizontal (heat-treated); scanning plane | 136 | 0.365 | 1.000 |
| Horizontal (heat-treated); scanning plane | 137 | 0.183 | 1.000 |
| Horizontal (heat-treated); scanning plane | 138 | 0.183 | 1.000 |
| Horizontal (heat-treated); scanning plane | 139 | 0.183 | 1.000 |
| Horizontal (heat-treated); scanning plane | 140 | 0.183 | 1.000 |
| Horizontal (heat-treated); scanning plane | 141 | 0.183 | 1.000 |
| Vertical (as-built); building plane | 1 | 0.683 | 1.000 |
| Vertical (as-built); building plane | 2 | 509.357 | 0.319 |
| Vertical (as-built); building plane | 3 | 0.171 | 1.000 |
| Vertical (as-built); building plane | 4 | 0.171 | 1.000 |
| Vertical (as-built); building plane | 5 | 0.171 | 1.000 |
| Vertical (as-built); building plane | 6 | 0.683 | 1.000 |
| Vertical (as-built); building plane | 7 | 0.171 | 1.000 |
| Vertical (as-built); building plane | 8 | 0.683 | 1.000 |
| Vertical (as-built); building plane | 9 | 1.537 | 1.000 |
| Vertical (as-built); building plane | 10 | 0.342 | 1.000 |
| Vertical (as-built); building plane | 11 | 3.586 | 0.880 |
| Vertical (as-built); building plane | 12 | 19.807 | 0.482 |
| Vertical (as-built); building plane | 13 | 199.440 | 0.394 |
| Vertical (as-built); building plane | 14 | 1.366 | 0.645 |
| Vertical (as-built); building plane | 15 | 0.854 | 0.513 |
| Vertical (as-built); building plane | 16 | 6.318 | 0.798 |
| Vertical (as-built); building plane | 17 | 798.443 | 0.238 |
| Vertical (as-built); building plane | 18 | 0.854 | 1.000 |
| Vertical (as-built); building plane | 19 | 0.171 | 1.000 |
| Vertical (as-built); building plane | 20 | 2.732 | 0.795 |
| Vertical (as-built); building plane | 21 | 0.342 | 1.000 |
| Vertical (as-built); building plane | 22 | 0.342 | 1.000 |
| Vertical (as-built); building plane | 23 | 0.171 | 1.000 |
| Vertical (as-built); building plane | 24 | 0.342 | 1.000 |
| Vertical (as-built); building plane | 25 | 1.195 | 0.800 |
| Vertical (as-built); building plane | 26 | 0.171 | 1.000 |
| Vertical (as-built); building plane | 27 | 0.342 | 1.000 |
| Vertical (as-built); building plane | 28 | 0.171 | 1.000 |
| Vertical (as-built); building plane | 29 | 70.863 | 0.690 |
| Vertical (as-built); building plane | 30 | 1.708 | 0.579 |
| Vertical (as-built); building plane | 31 | 61.471 | 0.596 |
| Vertical (as-built); building plane | 32 | 0.171 | 1.000 |
| Vertical (as-built); building plane | 33 | 2.903 | 0.985 |
| Vertical (as-built); building plane | 34 | 0.512 | 0.809 |
| Vertical (as-built); building plane | 35 | 0.342 | 0.785 |
| Vertical (as-built); building plane | 36 | 560.071 | 0.376 |
| Vertical (as-built); building plane | 37 | 1.195 | 0.800 |
| Vertical (as-built); building plane | 38 | 0.171 | 1.000 |
| Vertical (as-built); building plane | 39 | 87.255 | 0.361 |
| Vertical (as-built); building plane | 40 | 0.171 | 1.000 |
| Vertical (as-built); building plane | 41 | 0.171 | 1.000 |
| Vertical (as-built); building plane | 42 | 19.637 | 0.524 |
| Vertical (as-built); building plane | 43 | 0.342 | 1.000 |
| Vertical (as-built); building plane | 44 | 0.512 | 1.000 |
| Vertical (as-built); building plane | 45 | 0.171 | 1.000 |
| Vertical (as-built); building plane | 46 | 1.025 | 1.000 |
| Vertical (as-built); building plane | 47 | 3.074 | 0.687 |
| Vertical (as-built); building plane | 48 | 0.512 | 1.000 |
| Vertical (as-built); building plane | 49 | 1.195 | 1.000 |
| Vertical (as-built); building plane | 50 | 0.512 | 1.000 |
| Vertical (as-built); building plane | 51 | 0.171 | 1.000 |
| Vertical (as-built); building plane | 52 | 4.440 | 0.760 |
| Vertical (as-built); building plane | 53 | 0.171 | 1.000 |
| Vertical (as-built); building plane | 54 | 0.342 | 1.000 |
| Vertical (as-built); building plane | 55 | 0.171 | 1.000 |
| Vertical (as-built); building plane | 56 | 0.342 | 1.000 |
| Vertical (as-built); building plane | 57 | 0.171 | 1.000 |
| Vertical (as-built); building plane | 58 | 3.074 | 0.645 |
| Vertical (as-built); building plane | 59 | 0.171 | 1.000 |
| Vertical (as-built); building plane | 60 | 0.854 | 1.000 |
| Vertical (as-built); building plane | 61 | 2.903 | 0.763 |
| Vertical (as-built); building plane | 62 | 13.490 | 0.919 |
| Vertical (as-built); building plane | 63 | 0.854 | 0.764 |
| Vertical (as-built); building plane | 64 | 2.391 | 0.535 |
| Vertical (as-built); building plane | 65 | 0.342 | 1.000 |
| Vertical (as-built); building plane | 66 | 0.171 | 1.000 |
| Vertical (as-built); building plane | 67 | 0.171 | 1.000 |
| Vertical (as-built); building plane | 68 | 20.490 | 0.520 |
| Vertical (as-built); building plane | 69 | 0.171 | 1.000 |
| Vertical (as-built); building plane | 70 | 769.244 | 0.128 |
| Vertical (as-built); building plane | 71 | 0.342 | 0.785 |
| Vertical (as-built); building plane | 72 | 0.854 | 0.513 |
| Vertical (as-built); building plane | 73 | 1.708 | 0.579 |
| Vertical (as-built); building plane | 74 | 1.195 | 0.943 |
| Vertical (as-built); building plane | 75 | 2.391 | 0.750 |
| Vertical (as-built); building plane | 76 | 0.171 | 1.000 |
| Vertical (as-built); building plane | 77 | 0.342 | 1.000 |
| Vertical (as-built); building plane | 78 | 0.683 | 0.857 |
| Vertical (as-built); building plane | 79 | 2.732 | 0.927 |
| Vertical (as-built); building plane | 80 | 0.171 | 1.000 |
| Vertical (as-built); building plane | 81 | 0.171 | 1.000 |
| Vertical (as-built); building plane | 82 | 0.171 | 1.000 |
| Vertical (as-built); building plane | 83 | 0.171 | 1.000 |
| Vertical (as-built); building plane | 84 | 0.512 | 1.000 |
| Vertical (as-built); building plane | 85 | 0.171 | 1.000 |
| Vertical (as-built); building plane | 86 | 0.171 | 1.000 |
| Vertical (as-built); building plane | 87 | 0.171 | 1.000 |
| Vertical (as-built); building plane | 88 | 0.171 | 1.000 |
| Vertical (as-built); building plane | 89 | 0.171 | 1.000 |
| Vertical (as-built); building plane | 90 | 0.342 | 1.000 |
| Vertical (as-built); building plane | 91 | 0.171 | 1.000 |
| Vertical (as-built); building plane | 92 | 0.512 | 0.967 |
| Vertical (as-built); building plane | 93 | 0.171 | 1.000 |
| Vertical (as-built); building plane | 94 | 0.342 | 1.000 |
| Vertical (as-built); building plane | 95 | 0.512 | 0.967 |
| Vertical (as-built); building plane | 96 | 0.171 | 1.000 |
| Vertical (as-built); building plane | 97 | 0.171 | 1.000 |
| Vertical (as-built); building plane | 98 | 0.512 | 1.000 |
| Vertical (as-built); building plane | 99 | 1.195 | 0.687 |
| Vertical (as-built); building plane | 100 | 0.512 | 0.967 |
| Vertical (as-built); building plane | 101 | 0.683 | 0.857 |
| Vertical (as-built); building plane | 102 | 0.683 | 0.645 |
| Vertical (as-built); building plane | 103 | 0.171 | 1.000 |
| Vertical (as-built); building plane | 104 | 0.171 | 1.000 |
| Vertical (as-built); building plane | 105 | 1.025 | 1.000 |
| Vertical (as-built); building plane | 106 | 0.854 | 1.000 |
| Vertical (as-built); building plane | 107 | 0.171 | 1.000 |
| Vertical (as-built); building plane | 108 | 0.171 | 1.000 |
| Vertical (as-built); building plane | 109 | 2.391 | 0.472 |
| Vertical (as-built); building plane | 110 | 0.342 | 1.000 |
| Vertical (as-built); building plane | 111 | 0.171 | 1.000 |
| Vertical (as-built); building plane | 112 | 0.342 | 1.000 |
| Vertical (as-built); building plane | 113 | 0.171 | 1.000 |
| Vertical (as-built); building plane | 114 | 0.342 | 1.000 |
| Vertical (as-built); building plane | 115 | 1.025 | 0.809 |
| Vertical (as-built); building plane | 116 | 0.854 | 1.000 |
| Vertical (as-built); building plane | 117 | 0.171 | 1.000 |
| Vertical (as-built); building plane | 118 | 0.171 | 1.000 |
| Vertical (as-built); building plane | 119 | 0.171 | 1.000 |
| Vertical (as-built); building plane | 120 | 0.171 | 1.000 |
| Vertical (as-built); building plane | 121 | 0.171 | 1.000 |
| Vertical (as-built); building plane | 122 | 70.521 | 0.550 |
| Vertical (as-built); building plane | 123 | 0.171 | 1.000 |
| Vertical (as-built); building plane | 124 | 0.342 | 1.000 |
| Vertical (as-built); building plane | 125 | 0.512 | 1.000 |
| Vertical (as-built); building plane | 126 | 0.512 | 0.967 |
| Vertical (as-built); building plane | 127 | 0.512 | 1.000 |
| Vertical (as-built); building plane | 128 | 0.171 | 1.000 |
| Vertical (as-built); building plane | 129 | 0.342 | 1.000 |
| Vertical (as-built); building plane | 130 | 0.171 | 1.000 |
| Vertical (as-built); building plane | 131 | 0.171 | 1.000 |
| Vertical (as-built); building plane | 132 | 0.171 | 1.000 |
| Vertical (as-built); building plane | 133 | 0.512 | 1.000 |
| Vertical (as-built); building plane | 134 | 0.171 | 1.000 |
| Vertical (as-built); building plane | 135 | 0.171 | 1.000 |
| Vertical (as-built); building plane | 136 | 0.171 | 1.000 |
| Vertical (as-built); building plane | 137 | 0.171 | 1.000 |
| Vertical (as-built); building plane | 138 | 0.342 | 1.000 |
| Vertical (as-built); building plane | 139 | 0.171 | 1.000 |
| Vertical (as-built); building plane | 140 | 2.391 | 0.409 |
| Vertical (as-built); scanning plane | 1 | 0.539 | 1.000 |
| Vertical (as-built); scanning plane | 2 | 21.725 | 0.710 |
| Vertical (as-built); scanning plane | 3 | 1.616 | 0.799 |
| Vertical (as-built); scanning plane | 4 | 0.180 | 1.000 |
| Vertical (as-built); scanning plane | 5 | 4.309 | 0.941 |
| Vertical (as-built); scanning plane | 6 | 5.745 | 0.762 |
| Vertical (as-built); scanning plane | 7 | 0.180 | 1.000 |
| Vertical (as-built); scanning plane | 8 | 1.257 | 1.000 |
| Vertical (as-built); scanning plane | 9 | 0.180 | 1.000 |
| Vertical (as-built); scanning plane | 10 | 21.725 | 0.885 |
| Vertical (as-built); scanning plane | 11 | 33.755 | 0.698 |
| Vertical (as-built); scanning plane | 12 | 0.180 | 1.000 |
| Vertical (as-built); scanning plane | 13 | 18.673 | 0.427 |
| Vertical (as-built); scanning plane | 14 | 0.180 | 1.000 |
| Vertical (as-built); scanning plane | 15 | 28.548 | 0.765 |
| Vertical (as-built); scanning plane | 16 | 2.873 | 0.927 |
| Vertical (as-built); scanning plane | 17 | 150.101 | 0.523 |
| Vertical (as-built); scanning plane | 18 | 1.975 | 1.000 |
| Vertical (as-built); scanning plane | 19 | 0.180 | 1.000 |
| Vertical (as-built); scanning plane | 20 | 0.180 | 1.000 |
| Vertical (as-built); scanning plane | 21 | 0.359 | 0.785 |
| Vertical (as-built); scanning plane | 22 | 0.359 | 0.785 |
| Vertical (as-built); scanning plane | 23 | 12.568 | 0.542 |
| Vertical (as-built); scanning plane | 24 | 40.577 | 0.658 |
| Vertical (as-built); scanning plane | 25 | 0.898 | 1.000 |
| Vertical (as-built); scanning plane | 26 | 86.541 | 0.370 |
| Vertical (as-built); scanning plane | 27 | 0.180 | 1.000 |
| Vertical (as-built); scanning plane | 28 | 0.718 | 1.000 |
| Vertical (as-built); scanning plane | 29 | 18.493 | 0.455 |
| Vertical (as-built); scanning plane | 30 | 22.623 | 0.778 |
| Vertical (as-built); scanning plane | 31 | 14.902 | 0.803 |
| Vertical (as-built); scanning plane | 32 | 0.180 | 1.000 |
| Vertical (as-built); scanning plane | 33 | 1.257 | 0.943 |
| Vertical (as-built); scanning plane | 34 | 59.430 | 0.697 |
| Vertical (as-built); scanning plane | 35 | 3.411 | 1.000 |
| Vertical (as-built); scanning plane | 36 | 66.073 | 0.741 |
| Vertical (as-built); scanning plane | 37 | 33.216 | 0.824 |
| Vertical (as-built); scanning plane | 38 | 0.180 | 1.000 |
| Vertical (as-built); scanning plane | 39 | 3.591 | 0.925 |
| Vertical (as-built); scanning plane | 40 | 12.927 | 0.832 |
| Vertical (as-built); scanning plane | 41 | 0.718 | 1.000 |
| Vertical (as-built); scanning plane | 42 | 1.616 | 0.923 |
| Vertical (as-built); scanning plane | 43 | 0.180 | 1.000 |
| Vertical (as-built); scanning plane | 44 | 0.898 | 0.572 |
| Vertical (as-built); scanning plane | 45 | 10.234 | 0.524 |
| Vertical (as-built); scanning plane | 46 | 0.180 | 1.000 |
| Vertical (as-built); scanning plane | 47 | 1.257 | 1.000 |
| Vertical (as-built); scanning plane | 48 | 0.180 | 1.000 |
| Vertical (as-built); scanning plane | 49 | 10.593 | 0.733 |
| Vertical (as-built); scanning plane | 50 | 77.384 | 0.690 |
| Vertical (as-built); scanning plane | 51 | 56.916 | 0.731 |
| Vertical (as-built); scanning plane | 52 | 21.725 | 0.728 |
| Vertical (as-built); scanning plane | 53 | 1.257 | 1.000 |
| Vertical (as-built); scanning plane | 54 | 11.850 | 0.791 |
| Vertical (as-built); scanning plane | 55 | 3.052 | 0.820 |
| Vertical (as-built); scanning plane | 56 | 1.975 | 0.976 |
| Vertical (as-built); scanning plane | 57 | 9.157 | 0.722 |
| Vertical (as-built); scanning plane | 58 | 179.187 | 0.516 |
| Vertical (as-built); scanning plane | 59 | 4.489 | 0.774 |
| Vertical (as-built); scanning plane | 60 | 0.180 | 1.000 |
| Vertical (as-built); scanning plane | 61 | 0.180 | 1.000 |
| Vertical (as-built); scanning plane | 62 | 64.278 | 0.706 |
| Vertical (as-built); scanning plane | 63 | 0.180 | 1.000 |
| Vertical (as-built); scanning plane | 64 | 110.062 | 0.680 |
| Vertical (as-built); scanning plane | 65 | 6.284 | 0.451 |
| Vertical (as-built); scanning plane | 66 | 0.718 | 0.857 |
| Vertical (as-built); scanning plane | 67 | 0.180 | 1.000 |
| Vertical (as-built); scanning plane | 68 | 9.336 | 0.357 |
| Vertical (as-built); scanning plane | 69 | 14.723 | 0.902 |
| Vertical (as-built); scanning plane | 70 | 0.359 | 1.000 |
| Vertical (as-built); scanning plane | 71 | 1.616 | 1.000 |
| Vertical (as-built); scanning plane | 72 | 1.616 | 0.638 |
| Vertical (as-built); scanning plane | 73 | 4.130 | 0.878 |
| Vertical (as-built); scanning plane | 74 | 47.041 | 0.464 |
| Vertical (as-built); scanning plane | 75 | 0.359 | 1.000 |
| Vertical (as-built); scanning plane | 76 | 1.616 | 0.884 |
| Vertical (as-built); scanning plane | 77 | 0.539 | 1.000 |
| Vertical (as-built); scanning plane | 78 | 0.180 | 1.000 |
| Vertical (as-built); scanning plane | 79 | 0.898 | 1.000 |
| Vertical (as-built); scanning plane | 80 | 29.984 | 0.445 |
| Vertical (as-built); scanning plane | 81 | 0.180 | 1.000 |
| Vertical (as-built); scanning plane | 82 | 10.414 | 0.661 |
| Vertical (as-built); scanning plane | 83 | 1.975 | 0.659 |
| Vertical (as-built); scanning plane | 84 | 0.180 | 1.000 |
| Vertical (as-built); scanning plane | 85 | 0.359 | 1.000 |
| Vertical (as-built); scanning plane | 86 | 1.436 | 0.785 |
| Vertical (as-built); scanning plane | 87 | 8.259 | 0.499 |
| Vertical (as-built); scanning plane | 88 | 1.795 | 0.650 |
| Vertical (as-built); scanning plane | 89 | 0.180 | 1.000 |
| Vertical (as-built); scanning plane | 90 | 5.386 | 0.857 |
| Vertical (as-built); scanning plane | 91 | 1.436 | 1.000 |
| Vertical (as-built); scanning plane | 92 | 0.718 | 1.000 |
| Vertical (as-built); scanning plane | 93 | 0.898 | 0.873 |
| Vertical (as-built); scanning plane | 94 | 0.180 | 1.000 |
| Vertical (as-built); scanning plane | 95 | 0.359 | 0.785 |
| Vertical (as-built); scanning plane | 96 | 0.359 | 1.000 |
| Vertical (as-built); scanning plane | 97 | 8.439 | 0.721 |
| Vertical (as-built); scanning plane | 98 | 0.359 | 1.000 |
| Vertical (as-built); scanning plane | 99 | 1.077 | 0.916 |
| Vertical (as-built); scanning plane | 100 | 0.180 | 1.000 |
| Vertical (as-built); scanning plane | 101 | 69.484 | 0.712 |
| Vertical (as-built); scanning plane | 102 | 0.359 | 1.000 |
| Vertical (as-built); scanning plane | 103 | 0.180 | 1.000 |
| Vertical (as-built); scanning plane | 104 | 0.180 | 1.000 |
| Vertical (as-built); scanning plane | 105 | 0.180 | 1.000 |
| Vertical (as-built); scanning plane | 106 | 61.405 | 0.638 |
| Vertical (as-built); scanning plane | 107 | 3.591 | 0.838 |
| Vertical (as-built); scanning plane | 108 | 1.077 | 0.686 |
| Vertical (as-built); scanning plane | 109 | 11.491 | 0.517 |
| Vertical (as-built); scanning plane | 110 | 2.693 | 1.000 |
| Vertical (as-built); scanning plane | 111 | 0.180 | 1.000 |
| Vertical (as-built); scanning plane | 112 | 7.541 | 0.608 |
| Vertical (as-built); scanning plane | 113 | 4.848 | 0.431 |
| Vertical (as-built); scanning plane | 114 | 1.257 | 1.000 |
| Vertical (as-built); scanning plane | 115 | 0.898 | 0.572 |
| Vertical (as-built); scanning plane | 116 | 99.828 | 0.692 |
| Vertical (as-built); scanning plane | 117 | 0.180 | 1.000 |
| Vertical (as-built); scanning plane | 118 | 0.718 | 0.857 |
| Vertical (as-built); scanning plane | 119 | 0.180 | 1.000 |
| Vertical (as-built); scanning plane | 120 | 9.695 | 0.586 |
| Vertical (as-built); scanning plane | 121 | 3.411 | 0.796 |
| Vertical (as-built); scanning plane | 122 | 3.052 | 0.845 |
| Vertical (as-built); scanning plane | 123 | 0.539 | 1.000 |
| Vertical (as-built); scanning plane | 124 | 0.539 | 1.000 |
| Vertical (as-built); scanning plane | 125 | 0.180 | 1.000 |
| Vertical (as-built); scanning plane | 126 | 6.464 | 0.611 |
| Vertical (as-built); scanning plane | 127 | 2.155 | 0.967 |
| Vertical (as-built); scanning plane | 128 | 4.309 | 0.497 |
| Vertical (as-built); scanning plane | 129 | 2.873 | 0.772 |
| Vertical (as-built); scanning plane | 130 | 0.539 | 1.000 |
| Vertical (as-built); scanning plane | 131 | 115.089 | 0.674 |
| Vertical (as-built); scanning plane | 132 | 1.975 | 0.853 |
| Vertical (as-built); scanning plane | 133 | 0.180 | 1.000 |
| Vertical (as-built); scanning plane | 134 | 1.436 | 1.000 |
| Vertical (as-built); scanning plane | 135 | 0.898 | 1.000 |
| Vertical (as-built); scanning plane | 136 | 73.614 | 0.448 |
| Vertical (as-built); scanning plane | 137 | 1.077 | 0.916 |
| Vertical (as-built); scanning plane | 138 | 19.032 | 0.788 |
| Vertical (as-built); scanning plane | 139 | 0.539 | 0.809 |
| Vertical (as-built); scanning plane | 140 | 0.180 | 1.000 |
| Vertical (as-built); scanning plane | 141 | 1.616 | 0.884 |
| Vertical (as-built); scanning plane | 142 | 5.745 | 0.803 |
| Vertical (as-built); scanning plane | 143 | 0.180 | 1.000 |
| Vertical (as-built); scanning plane | 144 | 5.207 | 0.501 |
| Vertical (as-built); scanning plane | 145 | 95.698 | 0.439 |
| Vertical (as-built); scanning plane | 146 | 0.718 | 0.698 |
| Vertical (as-built); scanning plane | 147 | 0.180 | 1.000 |
| Vertical (as-built); scanning plane | 148 | 8.977 | 0.429 |
| Vertical (as-built); scanning plane | 149 | 5.207 | 0.953 |
| Vertical (as-built); scanning plane | 150 | 17.775 | 0.715 |
| Vertical (as-built); scanning plane | 151 | 0.539 | 1.000 |
| Vertical (as-built); scanning plane | 152 | 21.007 | 0.846 |
| Vertical (as-built); scanning plane | 153 | 1.436 | 0.567 |
| Vertical (as-built); scanning plane | 154 | 1.077 | 0.809 |
| Vertical (as-built); scanning plane | 155 | 0.539 | 1.000 |
| Vertical (as-built); scanning plane | 156 | 0.180 | 1.000 |
| Vertical (as-built); scanning plane | 157 | 2.334 | 1.000 |
| Vertical (as-built); scanning plane | 158 | 0.359 | 1.000 |
| Vertical (as-built); scanning plane | 159 | 2.155 | 1.000 |
| Vertical (as-built); scanning plane | 160 | 0.539 | 1.000 |
| Vertical (as-built); scanning plane | 161 | 43.450 | 0.780 |
| Vertical (as-built); scanning plane | 162 | 0.359 | 1.000 |
| Vertical (as-built); scanning plane | 163 | 0.180 | 1.000 |
| Vertical (as-built); scanning plane | 164 | 0.359 | 1.000 |
| Vertical (as-built); scanning plane | 165 | 16.518 | 0.622 |
| Vertical (as-built); scanning plane | 166 | 2.334 | 0.922 |
| Vertical (as-built); scanning plane | 167 | 0.359 | 1.000 |
| Vertical (as-built); scanning plane | 168 | 0.898 | 0.873 |
| Vertical (as-built); scanning plane | 169 | 0.898 | 1.000 |
| Vertical (as-built); scanning plane | 170 | 0.180 | 1.000 |
| Vertical (as-built); scanning plane | 171 | 1.616 | 0.884 |
| Vertical (as-built); scanning plane | 172 | 8.080 | 0.593 |
| Vertical (as-built); scanning plane | 173 | 26.393 | 0.717 |
| Vertical (as-built); scanning plane | 174 | 14.723 | 0.460 |
| Vertical (as-built); scanning plane | 175 | 4.848 | 0.772 |
| Vertical (as-built); scanning plane | 176 | 61.225 | 0.512 |
| Vertical (as-built); scanning plane | 177 | 0.180 | 1.000 |
| Vertical (as-built); scanning plane | 178 | 0.180 | 1.000 |
| Vertical (as-built); scanning plane | 179 | 0.180 | 1.000 |
| Vertical (as-built); scanning plane | 180 | 0.898 | 1.000 |
| Vertical (as-built); scanning plane | 181 | 0.180 | 1.000 |
| Vertical (as-built); scanning plane | 182 | 0.180 | 1.000 |
| Vertical (as-built); scanning plane | 183 | 1.257 | 0.687 |
| Vertical (as-built); scanning plane | 184 | 0.180 | 1.000 |
| Vertical (as-built); scanning plane | 185 | 3.232 | 0.965 |
| Vertical (as-built); scanning plane | 186 | 4.130 | 0.730 |
| Vertical (as-built); scanning plane | 187 | 0.359 | 1.000 |
| Vertical (as-built); scanning plane | 188 | 6.643 | 0.470 |
| Vertical (as-built); scanning plane | 189 | 31.959 | 0.709 |
| Vertical (as-built); scanning plane | 190 | 0.359 | 1.000 |
| Vertical (as-built); scanning plane | 191 | 3.052 | 0.820 |
| Vertical (as-built); scanning plane | 192 | 1.975 | 0.780 |
| Vertical (as-built); scanning plane | 193 | 2.514 | 0.549 |
| Vertical (as-built); scanning plane | 194 | 0.180 | 1.000 |
| Vertical (as-built); scanning plane | 195 | 4.130 | 0.486 |
| Vertical (as-built); scanning plane | 196 | 0.718 | 1.000 |
| Vertical (as-built); scanning plane | 197 | 0.539 | 0.643 |
| Vertical (as-built); scanning plane | 198 | 4.848 | 0.348 |
| Vertical (as-built); scanning plane | 199 | 29.984 | 0.691 |
| Vertical (as-built); scanning plane | 200 | 0.180 | 1.000 |
| Vertical (as-built); scanning plane | 201 | 6.284 | 0.673 |
| Vertical (as-built); scanning plane | 202 | 2.873 | 0.484 |
| Vertical (as-built); scanning plane | 203 | 0.180 | 1.000 |
| Vertical (as-built); scanning plane | 204 | 0.180 | 1.000 |
| Vertical (as-built); scanning plane | 205 | 8.977 | 0.736 |
| Vertical (as-built); scanning plane | 206 | 3.232 | 0.487 |
| Vertical (as-built); scanning plane | 207 | 0.359 | 0.785 |
| Vertical (as-built); scanning plane | 208 | 0.718 | 1.000 |
| Vertical (as-built); scanning plane | 209 | 0.180 | 1.000 |
| Vertical (as-built); scanning plane | 210 | 1.257 | 0.800 |
| Vertical (as-built); scanning plane | 211 | 0.718 | 1.000 |
| Vertical (as-built); scanning plane | 212 | 38.602 | 0.653 |
| Vertical (as-built); scanning plane | 213 | 2.693 | 0.629 |
| Vertical (as-built); scanning plane | 214 | 8.080 | 0.523 |
| Vertical (as-built); scanning plane | 215 | 1.975 | 0.780 |
| Vertical (as-built); scanning plane | 216 | 0.180 | 1.000 |
| Vertical (as-built); scanning plane | 217 | 3.052 | 0.911 |
| Vertical (as-built); scanning plane | 218 | 7.361 | 0.884 |
| Vertical (as-built); scanning plane | 219 | 0.539 | 1.000 |
| Vertical (as-built); scanning plane | 220 | 0.180 | 1.000 |
| Vertical (as-built); scanning plane | 221 | 1.257 | 0.621 |
| Vertical (as-built); scanning plane | 222 | 0.180 | 1.000 |
| Vertical (as-built); scanning plane | 223 | 13.286 | 0.208 |
| Vertical (as-built); scanning plane | 224 | 0.180 | 1.000 |
| Vertical (as-built); scanning plane | 225 | 9.516 | 0.862 |
| Vertical (as-built); scanning plane | 226 | 1.077 | 1.000 |
| Vertical (as-built); scanning plane | 227 | 1.795 | 0.709 |
| Vertical (as-built); scanning plane | 228 | 0.359 | 1.000 |
| Vertical (as-built); scanning plane | 229 | 0.359 | 1.000 |
| Vertical (as-built); scanning plane | 230 | 1.436 | 0.914 |
| Vertical (as-built); scanning plane | 231 | 3.052 | 0.594 |
| Vertical (as-built); scanning plane | 232 | 1.975 | 0.637 |
| Vertical (as-built); scanning plane | 233 | 0.180 | 1.000 |
| Vertical (as-built); scanning plane | 234 | 0.898 | 0.873 |
| Vertical (as-built); scanning plane | 235 | 3.950 | 0.595 |
| Vertical (as-built); scanning plane | 236 | 16.518 | 0.560 |
| Vertical (as-built); scanning plane | 237 | 0.898 | 0.764 |
| Vertical (as-built); scanning plane | 238 | 0.180 | 1.000 |
| Vertical (as-built); scanning plane | 239 | 0.539 | 1.000 |
| Vertical (as-built); scanning plane | 240 | 0.898 | 0.674 |
| Vertical (as-built); scanning plane | 241 | 0.180 | 1.000 |
| Vertical (as-built); scanning plane | 242 | 0.180 | 1.000 |
| Vertical (as-built); scanning plane | 243 | 4.130 | 0.673 |
| Vertical (as-built); scanning plane | 244 | 1.257 | 0.800 |
| Vertical (as-built); scanning plane | 245 | 0.718 | 0.857 |
| Vertical (as-built); scanning plane | 246 | 0.180 | 1.000 |
| Vertical (as-built); scanning plane | 247 | 0.180 | 1.000 |
| Vertical (as-built); scanning plane | 248 | 1.077 | 0.686 |
| Vertical (as-built); scanning plane | 249 | 0.539 | 1.000 |
| Vertical (as-built); scanning plane | 250 | 0.359 | 1.000 |
| Vertical (as-built); scanning plane | 251 | 0.180 | 1.000 |
| Vertical (as-built); scanning plane | 252 | 4.130 | 0.696 |
| Vertical (as-built); scanning plane | 253 | 0.180 | 1.000 |
| Vertical (as-built); scanning plane | 254 | 0.539 | 0.967 |
| Vertical (as-built); scanning plane | 255 | 0.539 | 1.000 |
| Vertical (as-built); scanning plane | 256 | 3.770 | 0.527 |
| Vertical (as-built); scanning plane | 257 | 3.770 | 1.000 |
| Vertical (as-built); scanning plane | 258 | 24.777 | 0.357 |
| Vertical (as-built); scanning plane | 259 | 0.180 | 1.000 |
| Vertical (as-built); scanning plane | 260 | 0.539 | 0.809 |
| Vertical (as-built); scanning plane | 261 | 2.873 | 0.611 |
| Vertical (as-built); scanning plane | 262 | 0.180 | 1.000 |
| Vertical (as-built); scanning plane | 263 | 0.539 | 0.967 |
| Vertical (as-built); scanning plane | 264 | 0.718 | 1.000 |
| Vertical (as-built); scanning plane | 265 | 0.898 | 0.873 |
| Vertical (as-built); scanning plane | 266 | 0.180 | 1.000 |
| Vertical (as-built); scanning plane | 267 | 2.155 | 0.579 |
| Vertical (as-built); scanning plane | 268 | 3.950 | 0.524 |
| Vertical (as-built); scanning plane | 269 | 0.180 | 1.000 |
| Vertical (as-built); scanning plane | 270 | 0.539 | 1.000 |
| Vertical (as-built); scanning plane | 271 | 28.907 | 0.429 |
| Vertical (as-built); scanning plane | 272 | 3.232 | 0.429 |
| Vertical (as-built); scanning plane | 273 | 0.359 | 1.000 |
| Vertical (as-built); scanning plane | 274 | 0.180 | 1.000 |
| Vertical (as-built); scanning plane | 275 | 19.571 | 0.227 |
| Vertical (as-built); scanning plane | 276 | 0.539 | 1.000 |
| Vertical (as-built); scanning plane | 277 | 0.359 | 1.000 |
| Vertical (as-built); scanning plane | 278 | 4.848 | 0.730 |
| Vertical (as-built); scanning plane | 279 | 0.359 | 1.000 |
| Vertical (as-built); scanning plane | 280 | 1.436 | 0.520 |
| Vertical (as-built); scanning plane | 281 | 0.359 | 1.000 |
| Vertical (as-built); scanning plane | 282 | 0.359 | 0.785 |
| Vertical (as-built); scanning plane | 283 | 0.359 | 0.785 |
| Vertical (as-built); scanning plane | 284 | 0.898 | 0.873 |
| Vertical (as-built); scanning plane | 285 | 42.732 | 0.581 |
| Vertical (as-built); scanning plane | 286 | 0.180 | 1.000 |
| Vertical (as-built); scanning plane | 287 | 0.180 | 1.000 |
| Vertical (as-built); scanning plane | 288 | 1.257 | 0.800 |
| Vertical (as-built); scanning plane | 289 | 1.436 | 0.645 |
| Vertical (as-built); scanning plane | 290 | 0.180 | 1.000 |
| Vertical (as-built); scanning plane | 291 | 0.180 | 1.000 |
| Vertical (as-built); scanning plane | 292 | 0.180 | 1.000 |
| Vertical (as-built); scanning plane | 293 | 9.516 | 0.513 |
| Vertical (as-built); scanning plane | 294 | 0.539 | 1.000 |
| Vertical (as-built); scanning plane | 295 | 0.898 | 0.873 |
| Vertical (as-built); scanning plane | 296 | 0.180 | 1.000 |
| Vertical (as-built); scanning plane | 297 | 16.877 | 0.668 |
| Vertical (as-built); scanning plane | 298 | 0.180 | 1.000 |
| Vertical (as-built); scanning plane | 299 | 9.336 | 0.513 |
| Vertical (as-built); scanning plane | 300 | 6.643 | 0.554 |
| Vertical (as-built); scanning plane | 301 | 0.180 | 1.000 |
| Vertical (as-built); scanning plane | 302 | 0.180 | 1.000 |
| Vertical (as-built); scanning plane | 303 | 1.436 | 0.914 |
| Vertical (as-built); scanning plane | 304 | 0.180 | 1.000 |
| Vertical (as-built); scanning plane | 305 | 1.795 | 0.887 |
| Vertical (as-built); scanning plane | 306 | 0.180 | 1.000 |
| Vertical (as-built); scanning plane | 307 | 2.873 | 0.559 |
| Vertical (as-built); scanning plane | 308 | 93.005 | 0.720 |
| Vertical (as-built); scanning plane | 309 | 0.180 | 1.000 |
| Vertical (as-built); scanning plane | 310 | 0.180 | 1.000 |
| Vertical (as-built); scanning plane | 311 | 0.718 | 1.000 |
| Vertical (as-built); scanning plane | 312 | 0.180 | 1.000 |
| Vertical (as-built); scanning plane | 313 | 1.975 | 0.637 |
| Vertical (as-built); scanning plane | 314 | 0.359 | 1.000 |
| Vertical (as-built); scanning plane | 315 | 0.180 | 1.000 |
| Vertical (as-built); scanning plane | 316 | 0.180 | 1.000 |
| Vertical (as-built); scanning plane | 317 | 4.309 | 0.726 |
| Vertical (as-built); scanning plane | 318 | 6.105 | 0.614 |
| Vertical (as-built); scanning plane | 319 | 0.539 | 1.000 |
| Vertical (as-built); scanning plane | 320 | 5.566 | 0.475 |
| Vertical (as-built); scanning plane | 321 | 13.107 | 0.654 |
| Vertical (as-built); scanning plane | 322 | 1.077 | 1.000 |
| Vertical (as-built); scanning plane | 323 | 2.693 | 0.869 |
| Vertical (as-built); scanning plane | 324 | 0.180 | 1.000 |
| Vertical (as-built); scanning plane | 325 | 78.282 | 0.720 |
| Vertical (as-built); scanning plane | 326 | 0.180 | 1.000 |
| Vertical (as-built); scanning plane | 327 | 0.718 | 0.857 |
| Vertical (as-built); scanning plane | 328 | 0.539 | 0.643 |
| Vertical (as-built); scanning plane | 329 | 0.180 | 1.000 |
| Vertical (as-built); scanning plane | 330 | 0.359 | 1.000 |
| Vertical (as-built); scanning plane | 331 | 0.180 | 1.000 |
| Vertical (as-built); scanning plane | 332 | 5.745 | 0.690 |
| Vertical (as-built); scanning plane | 333 | 0.180 | 1.000 |
| Vertical (as-built); scanning plane | 334 | 64.457 | 0.689 |
| Vertical (as-built); scanning plane | 335 | 0.359 | 1.000 |
| Vertical (as-built); scanning plane | 336 | 40.937 | 0.703 |
| Vertical (as-built); scanning plane | 337 | 0.180 | 1.000 |
| Vertical (as-built); scanning plane | 338 | 0.359 | 0.785 |
| Vertical (as-built); scanning plane | 339 | 0.898 | 0.873 |
| Vertical (as-built); scanning plane | 340 | 3.950 | 0.723 |
| Vertical (as-built); scanning plane | 341 | 0.718 | 0.857 |
| Vertical (as-built); scanning plane | 342 | 0.359 | 1.000 |
| Vertical (as-built); scanning plane | 343 | 30.164 | 0.594 |
| Vertical (as-built); scanning plane | 344 | 0.180 | 1.000 |
| Vertical (as-built); scanning plane | 345 | 1.616 | 0.799 |
| Vertical (as-built); scanning plane | 346 | 0.180 | 1.000 |
| Vertical (as-built); scanning plane | 347 | 8.439 | 0.455 |
| Vertical (as-built); scanning plane | 348 | 9.875 | 0.596 |
| Vertical (as-built); scanning plane | 349 | 14.364 | 0.489 |
| Vertical (heat-treated); building plane | 1 | 0.180 | 1.000 |
| Vertical (heat-treated); building plane | 2 | 0.718 | 0.698 |
| Vertical (heat-treated); building plane | 3 | 0.539 | 1.000 |
| Vertical (heat-treated); building plane | 4 | 0.180 | 1.000 |
| Vertical (heat-treated); building plane | 5 | 0.718 | 1.000 |
| Vertical (heat-treated); building plane | 6 | 0.359 | 1.000 |
| Vertical (heat-treated); building plane | 7 | 0.180 | 1.000 |
| Vertical (heat-treated); building plane | 8 | 0.180 | 1.000 |
| Vertical (heat-treated); building plane | 9 | 0.180 | 1.000 |
| Vertical (heat-treated); building plane | 10 | 0.180 | 1.000 |
| Vertical (heat-treated); building plane | 11 | 1.795 | 0.736 |
| Vertical (heat-treated); building plane | 12 | 5.027 | 0.920 |
| Vertical (heat-treated); building plane | 13 | 0.539 | 0.967 |
| Vertical (heat-treated); building plane | 14 | 1.616 | 1.000 |
| Vertical (heat-treated); building plane | 15 | 0.180 | 1.000 |
| Vertical (heat-treated); building plane | 16 | 2.873 | 0.468 |
| Vertical (heat-treated); building plane | 17 | 6.105 | 0.220 |
| Vertical (heat-treated); building plane | 18 | 0.539 | 1.000 |
| Vertical (heat-treated); building plane | 19 | 0.180 | 1.000 |
| Vertical (heat-treated); building plane | 20 | 1.077 | 0.916 |
| Vertical (heat-treated); building plane | 21 | 0.718 | 1.000 |
| Vertical (heat-treated); building plane | 22 | 0.180 | 1.000 |
| Vertical (heat-treated); building plane | 23 | 1.077 | 1.000 |
| Vertical (heat-treated); building plane | 24 | 0.539 | 0.754 |
| Vertical (heat-treated); building plane | 25 | 0.539 | 1.000 |
| Vertical (heat-treated); building plane | 26 | 0.359 | 1.000 |
| Vertical (heat-treated); building plane | 27 | 0.180 | 1.000 |
| Vertical (heat-treated); building plane | 28 | 4.489 | 0.311 |
| Vertical (heat-treated); building plane | 29 | 0.180 | 1.000 |
| Vertical (heat-treated); building plane | 30 | 0.180 | 1.000 |
| Vertical (heat-treated); building plane | 31 | 0.359 | 1.000 |
| Vertical (heat-treated); building plane | 32 | 0.539 | 0.967 |
| Vertical (heat-treated); building plane | 33 | 0.180 | 1.000 |
| Vertical (heat-treated); building plane | 34 | 1.436 | 0.820 |
| Vertical (heat-treated); building plane | 35 | 0.539 | 1.000 |
| Vertical (heat-treated); building plane | 36 | 0.539 | 1.000 |
| Vertical (heat-treated); building plane | 37 | 1.795 | 0.776 |
| Vertical (heat-treated); building plane | 38 | 1.975 | 1.000 |
| Vertical (heat-treated); building plane | 39 | 3.591 | 0.838 |
| Vertical (heat-treated); building plane | 40 | 0.539 | 1.000 |
| Vertical (heat-treated); building plane | 41 | 49.914 | 0.311 |
| Vertical (heat-treated); building plane | 42 | 0.180 | 1.000 |
| Vertical (heat-treated); building plane | 43 | 0.539 | 0.967 |
| Vertical (heat-treated); building plane | 44 | 0.180 | 1.000 |
| Vertical (heat-treated); building plane | 45 | 1.436 | 0.914 |
| Vertical (heat-treated); building plane | 46 | 0.180 | 1.000 |
| Vertical (heat-treated); building plane | 47 | 1.257 | 0.898 |
| Vertical (heat-treated); building plane | 48 | 110.780 | 0.368 |
| Vertical (heat-treated); building plane | 49 | 1.257 | 1.000 |
| Vertical (heat-treated); building plane | 50 | 0.359 | 0.785 |
| Vertical (heat-treated); building plane | 51 | 0.718 | 1.000 |
| Vertical (heat-treated); building plane | 52 | 1.436 | 1.000 |
| Vertical (heat-treated); building plane | 53 | 0.359 | 1.000 |
| Vertical (heat-treated); building plane | 54 | 0.180 | 1.000 |
| Vertical (heat-treated); building plane | 55 | 0.718 | 1.000 |
| Vertical (heat-treated); building plane | 56 | 0.180 | 1.000 |
| Vertical (heat-treated); building plane | 57 | 0.718 | 0.857 |
| Vertical (heat-treated); building plane | 58 | 0.718 | 0.857 |
| Vertical (heat-treated); building plane | 59 | 0.180 | 1.000 |
| Vertical (heat-treated); building plane | 60 | 0.359 | 1.000 |
| Vertical (heat-treated); building plane | 61 | 0.180 | 1.000 |
| Vertical (heat-treated); building plane | 62 | 0.359 | 1.000 |
| Vertical (heat-treated); building plane | 63 | 1.616 | 0.521 |
| Vertical (heat-treated); building plane | 64 | 0.359 | 1.000 |
| Vertical (heat-treated); building plane | 65 | 0.180 | 1.000 |
| Vertical (heat-treated); building plane | 66 | 0.359 | 1.000 |
| Vertical (heat-treated); building plane | 67 | 1.436 | 0.710 |
| Vertical (heat-treated); building plane | 68 | 0.718 | 1.000 |
| Vertical (heat-treated); building plane | 69 | 5.207 | 0.676 |
| Vertical (heat-treated); building plane | 70 | 1.616 | 0.638 |
| Vertical (heat-treated); building plane | 71 | 0.359 | 0.785 |
| Vertical (heat-treated); building plane | 72 | 4.309 | 0.726 |
| Vertical (heat-treated); building plane | 73 | 0.539 | 0.967 |
| Vertical (heat-treated); building plane | 74 | 3.411 | 0.775 |
| Vertical (heat-treated); building plane | 75 | 1.795 | 0.806 |
| Vertical (heat-treated); building plane | 76 | 0.718 | 1.000 |
| Vertical (heat-treated); building plane | 77 | 0.898 | 0.873 |
| Vertical (heat-treated); building plane | 78 | 0.180 | 1.000 |
| Vertical (heat-treated); building plane | 79 | 0.539 | 1.000 |
| Vertical (heat-treated); building plane | 80 | 0.180 | 1.000 |
| Vertical (heat-treated); building plane | 81 | 0.359 | 0.785 |
| Vertical (heat-treated); building plane | 82 | 0.180 | 1.000 |
| Vertical (heat-treated); building plane | 83 | 0.180 | 1.000 |
| Vertical (heat-treated); building plane | 84 | 0.359 | 1.000 |
| Vertical (heat-treated); building plane | 85 | 0.180 | 1.000 |
| Vertical (heat-treated); building plane | 86 | 0.718 | 0.698 |
| Vertical (heat-treated); building plane | 87 | 0.180 | 1.000 |
| Vertical (heat-treated); building plane | 88 | 2.514 | 0.727 |
| Vertical (heat-treated); building plane | 89 | 0.180 | 1.000 |
| Vertical (heat-treated); building plane | 90 | 0.359 | 1.000 |
| Vertical (heat-treated); building plane | 91 | 0.180 | 1.000 |
| Vertical (heat-treated); building plane | 92 | 0.180 | 1.000 |
| Vertical (heat-treated); building plane | 93 | 0.180 | 1.000 |
| Vertical (heat-treated); building plane | 94 | 0.180 | 1.000 |
| Vertical (heat-treated); building plane | 95 | 0.180 | 1.000 |
| Vertical (heat-treated); building plane | 96 | 0.359 | 1.000 |
| Vertical (heat-treated); building plane | 97 | 0.180 | 1.000 |
| Vertical (heat-treated); building plane | 98 | 0.539 | 0.967 |
| Vertical (heat-treated); building plane | 99 | 0.359 | 1.000 |
| Vertical (heat-treated); building plane | 100 | 0.898 | 1.000 |
| Vertical (heat-treated); building plane | 101 | 0.180 | 1.000 |
| Vertical (heat-treated); building plane | 102 | 0.180 | 1.000 |
| Vertical (heat-treated); building plane | 103 | 0.359 | 1.000 |
| Vertical (heat-treated); building plane | 104 | 0.180 | 1.000 |
| Vertical (heat-treated); building plane | 105 | 0.180 | 1.000 |
| Vertical (heat-treated); building plane | 106 | 0.539 | 1.000 |
| Vertical (heat-treated); building plane | 107 | 0.180 | 1.000 |
| Vertical (heat-treated); building plane | 108 | 15.800 | 0.211 |
| Vertical (heat-treated); building plane | 109 | 0.180 | 1.000 |
| Vertical (heat-treated); building plane | 110 | 0.180 | 1.000 |
| Vertical (heat-treated); building plane | 111 | 1.257 | 0.621 |
| Vertical (heat-treated); building plane | 112 | 0.180 | 1.000 |
| Vertical (heat-treated); building plane | 113 | 1.077 | 0.555 |
| Vertical (heat-treated); building plane | 114 | 0.180 | 1.000 |
| Vertical (heat-treated); building plane | 115 | 1.077 | 1.000 |
| Vertical (heat-treated); building plane | 116 | 0.359 | 1.000 |
| Vertical (heat-treated); building plane | 117 | 0.180 | 1.000 |
| Vertical (heat-treated); building plane | 118 | 0.359 | 1.000 |
| Vertical (heat-treated); building plane | 119 | 2.334 | 0.584 |
| Vertical (heat-treated); building plane | 120 | 0.359 | 1.000 |
| Vertical (heat-treated); building plane | 121 | 0.180 | 1.000 |
| Vertical (heat-treated); building plane | 122 | 0.180 | 1.000 |
| Vertical (heat-treated); building plane | 123 | 0.180 | 1.000 |
| Vertical (heat-treated); building plane | 124 | 0.539 | 1.000 |
| Vertical (heat-treated); building plane | 125 | 0.180 | 1.000 |
| Vertical (heat-treated); building plane | 126 | 5.027 | 0.800 |
| Vertical (heat-treated); building plane | 127 | 0.180 | 1.000 |
| Vertical (heat-treated); building plane | 128 | 0.359 | 1.000 |
| Vertical (heat-treated); building plane | 129 | 0.359 | 1.000 |
| Vertical (heat-treated); building plane | 130 | 0.359 | 1.000 |
| Vertical (heat-treated); building plane | 131 | 0.180 | 1.000 |
| Vertical (heat-treated); building plane | 132 | 0.539 | 1.000 |
| Vertical (heat-treated); building plane | 133 | 0.359 | 1.000 |
| Vertical (heat-treated); building plane | 134 | 0.539 | 1.000 |
| Vertical (heat-treated); building plane | 135 | 0.718 | 0.611 |
| Vertical (heat-treated); building plane | 136 | 0.180 | 1.000 |
| Vertical (heat-treated); building plane | 137 | 0.180 | 1.000 |
| Vertical (heat-treated); building plane | 138 | 0.180 | 1.000 |
| Vertical (heat-treated); building plane | 139 | 2.334 | 0.697 |
| Vertical (heat-treated); building plane | 140 | 0.180 | 1.000 |
| Vertical (heat-treated); building plane | 141 | 0.359 | 1.000 |
| Vertical (heat-treated); building plane | 142 | 0.180 | 1.000 |
| Vertical (heat-treated); building plane | 143 | 0.180 | 1.000 |
| Vertical (heat-treated); building plane | 144 | 0.180 | 1.000 |
| Vertical (heat-treated); building plane | 145 | 0.180 | 1.000 |
| Vertical (heat-treated); building plane | 146 | 0.180 | 1.000 |
| Vertical (heat-treated); building plane | 147 | 0.359 | 1.000 |
| Vertical (heat-treated); building plane | 148 | 0.718 | 1.000 |
| Vertical (heat-treated); building plane | 149 | 0.359 | 1.000 |
| Vertical (heat-treated); building plane | 150 | 0.180 | 1.000 |
| Vertical (heat-treated); building plane | 151 | 0.180 | 1.000 |
| Vertical (heat-treated); building plane | 152 | 0.180 | 1.000 |
| Vertical (heat-treated); building plane | 153 | 0.180 | 1.000 |
| Vertical (heat-treated); building plane | 154 | 0.180 | 1.000 |
| Vertical (heat-treated); building plane | 155 | 0.180 | 1.000 |
| Vertical (heat-treated); building plane | 156 | 0.180 | 1.000 |
| Vertical (heat-treated); building plane | 157 | 0.898 | 1.000 |
| Vertical (heat-treated); building plane | 158 | 0.180 | 1.000 |
| Vertical (heat-treated); building plane | 159 | 0.180 | 1.000 |
| Vertical (heat-treated); building plane | 160 | 0.898 | 0.873 |
| Vertical (heat-treated); building plane | 161 | 0.718 | 1.000 |
| Vertical (heat-treated); building plane | 162 | 5.027 | 0.539 |
| Vertical (heat-treated); building plane | 163 | 0.180 | 1.000 |
| Vertical (heat-treated); building plane | 164 | 1.616 | 0.799 |
| Vertical (heat-treated); building plane | 165 | 0.539 | 0.967 |
| Vertical (heat-treated); building plane | 166 | 1.077 | 0.532 |
| Vertical (heat-treated); building plane | 167 | 0.180 | 1.000 |
| Vertical (heat-treated); building plane | 168 | 0.718 | 0.857 |
| Vertical (heat-treated); building plane | 169 | 0.180 | 1.000 |
| Vertical (heat-treated); building plane | 170 | 0.359 | 1.000 |
| Vertical (heat-treated); building plane | 171 | 1.436 | 0.914 |
| Vertical (heat-treated); building plane | 172 | 0.180 | 1.000 |
| Vertical (heat-treated); building plane | 173 | 0.180 | 1.000 |
| Vertical (heat-treated); building plane | 174 | 0.180 | 1.000 |
| Vertical (heat-treated); building plane | 175 | 0.539 | 0.809 |
| Vertical (heat-treated); building plane | 176 | 0.718 | 0.645 |
| Vertical (heat-treated); building plane | 177 | 0.180 | 1.000 |
| Vertical (heat-treated); building plane | 178 | 0.180 | 1.000 |
| Vertical (heat-treated); building plane | 179 | 0.180 | 1.000 |
| Vertical (heat-treated); building plane | 180 | 0.180 | 1.000 |
| Vertical (heat-treated); building plane | 181 | 0.180 | 1.000 |
| Vertical (heat-treated); building plane | 182 | 0.180 | 1.000 |
| Vertical (heat-treated); building plane | 183 | 0.180 | 1.000 |
| Vertical (heat-treated); scanning plane | 1 | 0.365 | 1.000 |
| Vertical (heat-treated); scanning plane | 2 | 0.183 | 1.000 |
| Vertical (heat-treated); scanning plane | 3 | 0.913 | 0.873 |
| Vertical (heat-treated); scanning plane | 4 | 0.365 | 1.000 |
| Vertical (heat-treated); scanning plane | 5 | 60.085 | 0.495 |
| Vertical (heat-treated); scanning plane | 6 | 0.731 | 1.000 |
| Vertical (heat-treated); scanning plane | 7 | 22.098 | 0.728 |
| Vertical (heat-treated); scanning plane | 8 | 21.368 | 0.832 |
| Vertical (heat-treated); scanning plane | 9 | 0.548 | 1.000 |
| Vertical (heat-treated); scanning plane | 10 | 2.009 | 0.637 |
| Vertical (heat-treated); scanning plane | 11 | 11.506 | 0.943 |
| Vertical (heat-treated); scanning plane | 12 | 0.183 | 1.000 |
| Vertical (heat-treated); scanning plane | 13 | 0.548 | 1.000 |
| Vertical (heat-treated); scanning plane | 14 | 39.630 | 0.806 |
| Vertical (heat-treated); scanning plane | 15 | 0.183 | 1.000 |
| Vertical (heat-treated); scanning plane | 16 | 3.105 | 1.000 |
| Vertical (heat-treated); scanning plane | 17 | 0.731 | 1.000 |
| Vertical (heat-treated); scanning plane | 18 | 5.296 | 0.501 |
| Vertical (heat-treated); scanning plane | 19 | 46.570 | 0.199 |
| Vertical (heat-treated); scanning plane | 20 | 0.548 | 1.000 |
| Vertical (heat-treated); scanning plane | 21 | 43.466 | 0.883 |
| Vertical (heat-treated); scanning plane | 22 | 1.461 | 0.914 |
| Vertical (heat-treated); scanning plane | 23 | 6.209 | 0.754 |
| Vertical (heat-treated); scanning plane | 24 | 0.731 | 0.857 |
| Vertical (heat-treated); scanning plane | 25 | 4.566 | 0.896 |
| Vertical (heat-treated); scanning plane | 26 | 1.644 | 0.585 |
| Vertical (heat-treated); scanning plane | 27 | 0.183 | 1.000 |
| Vertical (heat-treated); scanning plane | 28 | 0.548 | 1.000 |
| Vertical (heat-treated); scanning plane | 29 | 2.557 | 1.000 |
| Vertical (heat-treated); scanning plane | 30 | 2.192 | 0.643 |
| Vertical (heat-treated); scanning plane | 31 | 30.499 | 0.778 |
| Vertical (heat-treated); scanning plane | 32 | 3.470 | 0.853 |
| Vertical (heat-treated); scanning plane | 33 | 9.131 | 0.919 |
| Vertical (heat-treated); scanning plane | 34 | 2.192 | 0.781 |
| Vertical (heat-treated); scanning plane | 35 | 8.766 | 0.707 |
| Vertical (heat-treated); scanning plane | 36 | 0.183 | 1.000 |
| Vertical (heat-treated); scanning plane | 37 | 8.584 | 0.547 |
| Vertical (heat-treated); scanning plane | 38 | 0.183 | 1.000 |
| Vertical (heat-treated); scanning plane | 39 | 3.287 | 0.571 |
| Vertical (heat-treated); scanning plane | 40 | 237.234 | 0.292 |
| Vertical (heat-treated); scanning plane | 41 | 8.036 | 0.831 |
| Vertical (heat-treated); scanning plane | 42 | 0.183 | 1.000 |
| Vertical (heat-treated); scanning plane | 43 | 0.183 | 1.000 |
| Vertical (heat-treated); scanning plane | 44 | 4.748 | 0.601 |
| Vertical (heat-treated); scanning plane | 45 | 7.488 | 0.655 |
| Vertical (heat-treated); scanning plane | 46 | 0.913 | 0.873 |
| Vertical (heat-treated); scanning plane | 47 | 2.009 | 0.780 |
| Vertical (heat-treated); scanning plane | 48 | 1.096 | 0.809 |
| Vertical (heat-treated); scanning plane | 49 | 11.688 | 0.871 |
| Vertical (heat-treated); scanning plane | 50 | 2.557 | 0.571 |
| Vertical (heat-treated); scanning plane | 51 | 0.913 | 0.873 |
| Vertical (heat-treated); scanning plane | 52 | 0.913 | 1.000 |
| Vertical (heat-treated); scanning plane | 53 | 1.644 | 1.000 |
| Vertical (heat-treated); scanning plane | 54 | 0.548 | 1.000 |
| Vertical (heat-treated); scanning plane | 55 | 1.461 | 0.820 |
| Vertical (heat-treated); scanning plane | 56 | 1.096 | 0.809 |
| Vertical (heat-treated); scanning plane | 57 | 5.844 | 0.530 |
| Vertical (heat-treated); scanning plane | 58 | 0.548 | 1.000 |
| Vertical (heat-treated); scanning plane | 59 | 1.826 | 0.806 |
| Vertical (heat-treated); scanning plane | 60 | 1.826 | 0.887 |
| Vertical (heat-treated); scanning plane | 61 | 0.913 | 0.599 |
| Vertical (heat-treated); scanning plane | 62 | 5.661 | 0.937 |
| Vertical (heat-treated); scanning plane | 63 | 69.764 | 0.787 |
| Vertical (heat-treated); scanning plane | 64 | 7.670 | 0.846 |
| Vertical (heat-treated); scanning plane | 65 | 9.862 | 0.795 |
| Vertical (heat-treated); scanning plane | 66 | 0.183 | 1.000 |
| Vertical (heat-treated); scanning plane | 67 | 6.757 | 0.838 |
| Vertical (heat-treated); scanning plane | 68 | 32.143 | 0.760 |
| Vertical (heat-treated); scanning plane | 69 | 6.209 | 0.941 |
| Vertical (heat-treated); scanning plane | 70 | 2.922 | 0.958 |
| Vertical (heat-treated); scanning plane | 71 | 13.149 | 0.662 |
| Vertical (heat-treated); scanning plane | 72 | 0.183 | 1.000 |
| Vertical (heat-treated); scanning plane | 73 | 33.056 | 0.567 |
| Vertical (heat-treated); scanning plane | 74 | 19.176 | 0.848 |
| Vertical (heat-treated); scanning plane | 75 | 5.661 | 0.838 |
| Vertical (heat-treated); scanning plane | 76 | 11.871 | 0.905 |
| Vertical (heat-treated); scanning plane | 77 | 83.461 | 0.810 |
| Vertical (heat-treated); scanning plane | 78 | 1.461 | 1.000 |
| Vertical (heat-treated); scanning plane | 79 | 10.227 | 0.750 |
| Vertical (heat-treated); scanning plane | 80 | 0.731 | 0.857 |
| Vertical (heat-treated); scanning plane | 81 | 3.287 | 0.808 |
| Vertical (heat-treated); scanning plane | 82 | 4.566 | 0.896 |
| Vertical (heat-treated); scanning plane | 83 | 6.027 | 0.828 |
| Vertical (heat-treated); scanning plane | 84 | 4.931 | 0.836 |
| Vertical (heat-treated); scanning plane | 85 | 2.922 | 1.000 |
| Vertical (heat-treated); scanning plane | 86 | 3.835 | 0.856 |
| Vertical (heat-treated); scanning plane | 87 | 0.548 | 0.754 |
| Vertical (heat-treated); scanning plane | 88 | 18.993 | 0.839 |
| Vertical (heat-treated); scanning plane | 89 | 0.913 | 0.641 |
| Vertical (heat-treated); scanning plane | 90 | 4.383 | 0.433 |
| Vertical (heat-treated); scanning plane | 91 | 6.940 | 0.618 |
| Vertical (heat-treated); scanning plane | 92 | 1.826 | 0.806 |
| Vertical (heat-treated); scanning plane | 93 | 4.566 | 0.954 |
| Vertical (heat-treated); scanning plane | 94 | 77.252 | 0.786 |
| Vertical (heat-treated); scanning plane | 95 | 43.831 | 0.856 |
| Vertical (heat-treated); scanning plane | 96 | 24.107 | 0.837 |
| Vertical (heat-treated); scanning plane | 97 | 16.437 | 0.871 |
| Vertical (heat-treated); scanning plane | 98 | 1.096 | 1.000 |
| Vertical (heat-treated); scanning plane | 99 | 0.365 | 0.785 |
| Vertical (heat-treated); scanning plane | 100 | 0.183 | 1.000 |
| Vertical (heat-treated); scanning plane | 101 | 7.488 | 0.866 |
| Vertical (heat-treated); scanning plane | 102 | 5.296 | 0.898 |
| Vertical (heat-treated); scanning plane | 103 | 0.731 | 1.000 |
| Vertical (heat-treated); scanning plane | 104 | 6.575 | 0.876 |
| Vertical (heat-treated); scanning plane | 105 | 0.183 | 1.000 |
| Vertical (heat-treated); scanning plane | 106 | 1.461 | 0.914 |
| Vertical (heat-treated); scanning plane | 107 | 2.557 | 0.811 |
| Vertical (heat-treated); scanning plane | 108 | 0.183 | 1.000 |
| Vertical (heat-treated); scanning plane | 109 | 2.739 | 0.537 |
| Vertical (heat-treated); scanning plane | 110 | 23.376 | 0.875 |
| Vertical (heat-treated); scanning plane | 111 | 8.949 | 0.693 |
| Vertical (heat-treated); scanning plane | 112 | 72.138 | 0.763 |
| Vertical (heat-treated); scanning plane | 113 | 3.470 | 1.000 |
| Vertical (heat-treated); scanning plane | 114 | 5.844 | 0.676 |
| Vertical (heat-treated); scanning plane | 115 | 1.278 | 0.898 |
| Vertical (heat-treated); scanning plane | 116 | 27.942 | 0.897 |
| Vertical (heat-treated); scanning plane | 117 | 0.548 | 1.000 |
| Vertical (heat-treated); scanning plane | 118 | 81.087 | 0.769 |
| Vertical (heat-treated); scanning plane | 119 | 0.913 | 1.000 |
| Vertical (heat-treated); scanning plane | 120 | 0.183 | 1.000 |
| Vertical (heat-treated); scanning plane | 121 | 0.731 | 1.000 |
| Vertical (heat-treated); scanning plane | 122 | 9.314 | 0.938 |
| Vertical (heat-treated); scanning plane | 123 | 104.281 | 0.683 |
| Vertical (heat-treated); scanning plane | 124 | 6.575 | 0.680 |
| Vertical (heat-treated); scanning plane | 125 | 39.265 | 0.856 |
| Vertical (heat-treated); scanning plane | 126 | 34.152 | 0.929 |
| Vertical (heat-treated); scanning plane | 127 | 3.835 | 0.856 |
| Vertical (heat-treated); scanning plane | 128 | 2.192 | 1.000 |
| Vertical (heat-treated); scanning plane | 129 | 2.739 | 1.000 |
| Vertical (heat-treated); scanning plane | 130 | 0.548 | 1.000 |
| Vertical (heat-treated); scanning plane | 131 | 5.844 | 0.991 |
| Vertical (heat-treated); scanning plane | 132 | 0.183 | 1.000 |
| Vertical (heat-treated); scanning plane | 133 | 0.548 | 1.000 |
| Vertical (heat-treated); scanning plane | 134 | 4.383 | 0.978 |
| Vertical (heat-treated); scanning plane | 135 | 0.183 | 1.000 |
| Vertical (heat-treated); scanning plane | 136 | 6.392 | 0.546 |
| Vertical (heat-treated); scanning plane | 137 | 15.341 | 0.787 |
| Vertical (heat-treated); scanning plane | 138 | 0.731 | 1.000 |
| Vertical (heat-treated); scanning plane | 139 | 0.365 | 1.000 |
| Vertical (heat-treated); scanning plane | 140 | 3.470 | 0.916 |
| Vertical (heat-treated); scanning plane | 141 | 1.461 | 0.914 |
| Vertical (heat-treated); scanning plane | 142 | 0.183 | 1.000 |
| Vertical (heat-treated); scanning plane | 143 | 0.731 | 0.857 |
| Vertical (heat-treated); scanning plane | 144 | 47.483 | 0.836 |
| Vertical (heat-treated); scanning plane | 145 | 0.183 | 1.000 |
| Vertical (heat-treated); scanning plane | 146 | 0.365 | 1.000 |
| Vertical (heat-treated); scanning plane | 147 | 0.731 | 1.000 |
| Vertical (heat-treated); scanning plane | 148 | 1.461 | 1.000 |
| Vertical (heat-treated); scanning plane | 149 | 0.365 | 1.000 |
| Vertical (heat-treated); scanning plane | 150 | 2.922 | 0.772 |
| Vertical (heat-treated); scanning plane | 151 | 0.913 | 1.000 |
| Vertical (heat-treated); scanning plane | 152 | 1.096 | 0.686 |
| Vertical (heat-treated); scanning plane | 153 | 10.410 | 0.673 |
| Vertical (heat-treated); scanning plane | 154 | 10.045 | 0.627 |
| Vertical (heat-treated); scanning plane | 155 | 4.383 | 0.941 |
| Vertical (heat-treated); scanning plane | 156 | 0.183 | 1.000 |
| Vertical (heat-treated); scanning plane | 157 | 10.410 | 0.586 |
| Vertical (heat-treated); scanning plane | 158 | 3.105 | 0.763 |
| Vertical (heat-treated); scanning plane | 159 | 0.183 | 1.000 |
| Vertical (heat-treated); scanning plane | 160 | 92.045 | 0.845 |
| Vertical (heat-treated); scanning plane | 161 | 0.548 | 1.000 |
| Vertical (heat-treated); scanning plane | 162 | 0.183 | 1.000 |
| Vertical (heat-treated); scanning plane | 163 | 0.183 | 1.000 |
| Vertical (heat-treated); scanning plane | 164 | 3.470 | 0.853 |
| Vertical (heat-treated); scanning plane | 165 | 0.183 | 1.000 |
| Vertical (heat-treated); scanning plane | 166 | 0.183 | 1.000 |
| Vertical (heat-treated); scanning plane | 167 | 1.826 | 0.887 |
| Vertical (heat-treated); scanning plane | 168 | 18.628 | 0.577 |
| Vertical (heat-treated); scanning plane | 169 | 24.655 | 0.852 |
| Vertical (heat-treated); scanning plane | 170 | 5.844 | 0.710 |
| Vertical (heat-treated); scanning plane | 171 | 0.548 | 1.000 |
| Vertical (heat-treated); scanning plane | 172 | 0.365 | 1.000 |
| Vertical (heat-treated); scanning plane | 173 | 58.989 | 0.838 |
| Vertical (heat-treated); scanning plane | 174 | 91.497 | 0.540 |
| Vertical (heat-treated); scanning plane | 175 | 0.183 | 1.000 |
| Vertical (heat-treated); scanning plane | 176 | 3.653 | 0.466 |
| Vertical (heat-treated); scanning plane | 177 | 0.365 | 1.000 |
| Vertical (heat-treated); scanning plane | 178 | 25.203 | 0.944 |
| Vertical (heat-treated); scanning plane | 179 | 7.853 | 0.659 |
| Vertical (heat-treated); scanning plane | 180 | 4.931 | 0.887 |
| Vertical (heat-treated); scanning plane | 181 | 0.548 | 0.967 |
| Vertical (heat-treated); scanning plane | 182 | 2.739 | 0.804 |
| Vertical (heat-treated); scanning plane | 183 | 0.365 | 1.000 |
| Vertical (heat-treated); scanning plane | 184 | 2.374 | 1.000 |
| Vertical (heat-treated); scanning plane | 185 | 2.009 | 0.589 |
| Vertical (heat-treated); scanning plane | 186 | 7.123 | 0.929 |
| Vertical (heat-treated); scanning plane | 187 | 2.922 | 0.652 |
| Vertical (heat-treated); scanning plane | 188 | 0.913 | 0.873 |
| Vertical (heat-treated); scanning plane | 189 | 31.412 | 0.855 |
| Vertical (heat-treated); scanning plane | 190 | 1.461 | 1.000 |
| Vertical (heat-treated); scanning plane | 191 | 0.913 | 1.000 |
| Vertical (heat-treated); scanning plane | 192 | 89.305 | 0.713 |
| Vertical (heat-treated); scanning plane | 193 | 2.192 | 0.851 |
| Vertical (heat-treated); scanning plane | 194 | 21.002 | 0.940 |
| Vertical (heat-treated); scanning plane | 195 | 0.183 | 1.000 |
| Vertical (heat-treated); scanning plane | 196 | 0.548 | 0.643 |
| Vertical (heat-treated); scanning plane | 197 | 1.644 | 1.000 |
| Vertical (heat-treated); scanning plane | 198 | 8.584 | 0.798 |
| Vertical (heat-treated); scanning plane | 199 | 27.760 | 0.593 |
| Vertical (heat-treated); scanning plane | 200 | 19.359 | 0.845 |
| Vertical (heat-treated); scanning plane | 201 | 1.826 | 0.709 |
| Vertical (heat-treated); scanning plane | 202 | 0.183 | 1.000 |
| Vertical (heat-treated); scanning plane | 203 | 1.278 | 1.000 |
| Vertical (heat-treated); scanning plane | 204 | 1.826 | 0.776 |
| Vertical (heat-treated); scanning plane | 205 | 3.835 | 0.444 |
| Vertical (heat-treated); scanning plane | 206 | 2.739 | 0.869 |
| Vertical (heat-treated); scanning plane | 207 | 0.365 | 0.785 |
| Vertical (heat-treated); scanning plane | 208 | 0.183 | 1.000 |
| Vertical (heat-treated); scanning plane | 209 | 2.739 | 0.493 |
| Vertical (heat-treated); scanning plane | 210 | 0.183 | 1.000 |
| Vertical (heat-treated); scanning plane | 211 | 5.296 | 0.319 |
| Vertical (heat-treated); scanning plane | 212 | 2.922 | 0.508 |
| Vertical (heat-treated); scanning plane | 213 | 0.183 | 1.000 |
| Vertical (heat-treated); scanning plane | 214 | 1.278 | 1.000 |
| Vertical (heat-treated); scanning plane | 215 | 0.183 | 1.000 |
| Vertical (heat-treated); scanning plane | 216 | 0.731 | 0.740 |
| Vertical (heat-treated); scanning plane | 217 | 2.192 | 0.967 |
| Vertical (heat-treated); scanning plane | 218 | 0.365 | 1.000 |
| Vertical (heat-treated); scanning plane | 219 | 2.374 | 0.922 |
| Vertical (heat-treated); scanning plane | 220 | 45.109 | 0.840 |
| Vertical (heat-treated); scanning plane | 221 | 1.278 | 1.000 |
| Vertical (heat-treated); scanning plane | 222 | 111.586 | 0.633 |
| Vertical (heat-treated); scanning plane | 223 | 20.637 | 0.826 |
| Vertical (heat-treated); scanning plane | 224 | 1.644 | 1.000 |
| Vertical (heat-treated); scanning plane | 225 | 0.183 | 1.000 |
| Vertical (heat-treated); scanning plane | 226 | 0.731 | 1.000 |
| Vertical (heat-treated); scanning plane | 227 | 0.731 | 1.000 |
| Vertical (heat-treated); scanning plane | 228 | 22.829 | 0.889 |
| Vertical (heat-treated); scanning plane | 229 | 0.365 | 1.000 |
| Vertical (heat-treated); scanning plane | 230 | 46.570 | 0.892 |
| Vertical (heat-treated); scanning plane | 231 | 1.278 | 0.898 |
| Vertical (heat-treated); scanning plane | 232 | 4.931 | 0.943 |
| Vertical (heat-treated); scanning plane | 233 | 111.951 | 0.763 |
| Vertical (heat-treated); scanning plane | 234 | 10.775 | 0.920 |
| Vertical (heat-treated); scanning plane | 235 | 0.913 | 0.873 |
| Vertical (heat-treated); scanning plane | 236 | 0.548 | 1.000 |
| Vertical (heat-treated); scanning plane | 237 | 86.748 | 0.709 |
| Vertical (heat-treated); scanning plane | 238 | 1.278 | 0.943 |
| Vertical (heat-treated); scanning plane | 239 | 7.123 | 0.841 |
| Vertical (heat-treated); scanning plane | 240 | 12.967 | 0.930 |
| Vertical (heat-treated); scanning plane | 241 | 5.296 | 0.877 |
| Vertical (heat-treated); scanning plane | 242 | 0.183 | 1.000 |
| Vertical (heat-treated); scanning plane | 243 | 2.009 | 0.887 |
| Vertical (heat-treated); scanning plane | 244 | 51.319 | 0.637 |
| Vertical (heat-treated); scanning plane | 245 | 0.731 | 1.000 |
| Vertical (heat-treated); scanning plane | 246 | 2.739 | 0.804 |
| Vertical (heat-treated); scanning plane | 247 | 0.548 | 1.000 |
| Vertical (heat-treated); scanning plane | 248 | 7.670 | 0.846 |
| Vertical (heat-treated); scanning plane | 249 | 33.604 | 0.656 |
| Vertical (heat-treated); scanning plane | 250 | 0.365 | 1.000 |
| Vertical (heat-treated); scanning plane | 251 | 1.461 | 1.000 |
| Vertical (heat-treated); scanning plane | 252 | 0.365 | 1.000 |
| Vertical (heat-treated); scanning plane | 253 | 0.183 | 1.000 |
| Vertical (heat-treated); scanning plane | 254 | 14.793 | 0.866 |
| Vertical (heat-treated); scanning plane | 255 | 0.913 | 1.000 |
| Vertical (heat-treated); scanning plane | 256 | 58.258 | 0.830 |
| Vertical (heat-treated); scanning plane | 257 | 3.105 | 0.693 |
| Vertical (heat-treated); scanning plane | 258 | 1.644 | 0.585 |
| Vertical (heat-treated); scanning plane | 259 | 2.739 | 0.723 |
| Vertical (heat-treated); scanning plane | 260 | 0.183 | 1.000 |
| Vertical (heat-treated); scanning plane | 261 | 21.368 | 0.742 |
| Vertical (heat-treated); scanning plane | 262 | 0.183 | 1.000 |
| Vertical (heat-treated); scanning plane | 263 | 2.922 | 0.857 |
| Vertical (heat-treated); scanning plane | 264 | 0.365 | 1.000 |
| Vertical (heat-treated); scanning plane | 265 | 2.922 | 0.652 |
| Vertical (heat-treated); scanning plane | 266 | 22.098 | 0.937 |
| Vertical (heat-treated); scanning plane | 267 | 0.365 | 1.000 |
| Vertical (heat-treated); scanning plane | 268 | 0.731 | 0.857 |
| Vertical (heat-treated); scanning plane | 269 | 0.913 | 1.000 |
| Vertical (heat-treated); scanning plane | 270 | 5.296 | 0.898 |
| Vertical (heat-treated); scanning plane | 271 | 1.461 | 1.000 |
| Vertical (heat-treated); scanning plane | 272 | 2.374 | 0.697 |
| Vertical (heat-treated); scanning plane | 273 | 38.900 | 0.360 |
| Vertical (heat-treated); scanning plane | 274 | 0.183 | 1.000 |
| Vertical (heat-treated); scanning plane | 275 | 0.548 | 1.000 |
| Vertical (heat-treated); scanning plane | 276 | 5.661 | 0.655 |
| Vertical (heat-treated); scanning plane | 277 | 1.644 | 1.000 |
| Vertical (heat-treated); scanning plane | 278 | 32.143 | 0.847 |
| Vertical (heat-treated); scanning plane | 279 | 2.739 | 0.804 |
| Vertical (heat-treated); scanning plane | 280 | 12.784 | 0.593 |
| Vertical (heat-treated); scanning plane | 281 | 0.365 | 1.000 |
| Vertical (heat-treated); scanning plane | 282 | 0.183 | 1.000 |
| Vertical (heat-treated); scanning plane | 283 | 0.548 | 1.000 |
| Vertical (heat-treated); scanning plane | 284 | 0.548 | 1.000 |
| Vertical (heat-treated); scanning plane | 285 | 9.497 | 0.454 |
| Vertical (heat-treated); scanning plane | 286 | 6.392 | 0.445 |
| Vertical (heat-treated); scanning plane | 287 | 10.592 | 0.392 |
| Vertical (heat-treated); scanning plane | 288 | 0.183 | 1.000 |
| Vertical (heat-treated); scanning plane | 289 | 2.009 | 0.780 |
| Vertical (heat-treated); scanning plane | 290 | 0.183 | 1.000 |
| Vertical (heat-treated); scanning plane | 291 | 0.548 | 0.809 |
| Vertical (heat-treated); scanning plane | 292 | 1.461 | 0.820 |
| Vertical (heat-treated); scanning plane | 293 | 22.281 | 0.468 |
| Vertical (heat-treated); scanning plane | 294 | 0.365 | 1.000 |
| Vertical (heat-treated); scanning plane | 295 | 0.731 | 0.740 |
| Vertical (heat-treated); scanning plane | 296 | 30.499 | 0.858 |
| Vertical (heat-treated); scanning plane | 297 | 0.365 | 1.000 |
| Vertical (heat-treated); scanning plane | 298 | 0.183 | 1.000 |
| Vertical (heat-treated); scanning plane | 299 | 0.913 | 1.000 |
| Vertical (heat-treated); scanning plane | 300 | 1.096 | 0.686 |
| Vertical (heat-treated); scanning plane | 301 | 0.548 | 0.967 |
| Vertical (heat-treated); scanning plane | 302 | 1.826 | 0.392 |
| Vertical (heat-treated); scanning plane | 303 | 0.731 | 0.740 |
| Vertical (heat-treated); scanning plane | 304 | 0.365 | 1.000 |
| Vertical (heat-treated); scanning plane | 305 | 168.383 | 0.408 |
| Vertical (heat-treated); scanning plane | 306 | 0.183 | 1.000 |
| Vertical (heat-treated); scanning plane | 307 | 0.731 | 1.000 |
| Vertical (heat-treated); scanning plane | 308 | 0.548 | 1.000 |
| Vertical (heat-treated); scanning plane | 309 | 0.183 | 1.000 |
| Vertical (heat-treated); scanning plane | 310 | 0.183 | 1.000 |
| Vertical (heat-treated); scanning plane | 311 | 6.940 | 0.375 |
| Vertical (heat-treated); scanning plane | 312 | 0.548 | 0.967 |
| Vertical (heat-treated); scanning plane | 313 | 1.096 | 1.000 |
| Vertical (heat-treated); scanning plane | 314 | 6.940 | 0.803 |
| Vertical (heat-treated); scanning plane | 315 | 0.548 | 1.000 |
| Vertical (heat-treated); scanning plane | 316 | 2.374 | 0.846 |
| Vertical (heat-treated); scanning plane | 317 | 0.365 | 1.000 |
| Vertical (heat-treated); scanning plane | 318 | 0.183 | 1.000 |
| Vertical (heat-treated); scanning plane | 319 | 1.644 | 0.799 |
| Vertical (heat-treated); scanning plane | 320 | 37.439 | 0.783 |
| Vertical (heat-treated); scanning plane | 321 | 4.931 | 0.887 |
| Vertical (heat-treated); scanning plane | 322 | 0.183 | 1.000 |
| Vertical (heat-treated); scanning plane | 323 | 0.183 | 1.000 |
| Vertical (heat-treated); scanning plane | 324 | 0.183 | 1.000 |
| Vertical (heat-treated); scanning plane | 325 | 37.804 | 0.807 |
| Vertical (heat-treated); scanning plane | 326 | 0.913 | 0.764 |
| Vertical (heat-treated); scanning plane | 327 | 5.296 | 0.676 |
| Vertical (heat-treated); scanning plane | 328 | 0.183 | 1.000 |
| Vertical (heat-treated); scanning plane | 329 | 0.183 | 1.000 |
| Vertical (heat-treated); scanning plane | 330 | 1.644 | 0.799 |
| Vertical (heat-treated); scanning plane | 331 | 89.671 | 0.686 |
| Vertical (heat-treated); scanning plane | 332 | 7.488 | 0.655 |
| Vertical (heat-treated); scanning plane | 333 | 0.365 | 1.000 |
| Vertical (heat-treated); scanning plane | 334 | 45.109 | 0.788 |
| Vertical (heat-treated); scanning plane | 335 | 0.365 | 1.000 |
| Vertical (heat-treated); scanning plane | 336 | 0.183 | 1.000 |
| Vertical (heat-treated); scanning plane | 337 | 3.105 | 0.820 |
| Vertical (heat-treated); scanning plane | 338 | 48.031 | 0.871 |
| Vertical (heat-treated); scanning plane | 339 | 1.278 | 0.943 |
| Vertical (heat-treated); scanning plane | 340 | 0.183 | 1.000 |
| Vertical (heat-treated); scanning plane | 341 | 1.461 | 1.000 |
| Vertical (heat-treated); scanning plane | 342 | 1.278 | 1.000 |
| Vertical (heat-treated); scanning plane | 343 | 0.183 | 1.000 |
| Vertical (heat-treated); scanning plane | 344 | 8.584 | 0.692 |
| Vertical (heat-treated); scanning plane | 345 | 0.548 | 1.000 |
| Vertical (heat-treated); scanning plane | 346 | 2.739 | 0.694 |
| Vertical (heat-treated); scanning plane | 347 | 2.739 | 0.723 |
| Vertical (heat-treated); scanning plane | 348 | 0.731 | 0.857 |
| Vertical (heat-treated); scanning plane | 349 | 1.826 | 0.536 |
| Vertical (heat-treated); scanning plane | 350 | 0.365 | 1.000 |
| Vertical (heat-treated); scanning plane | 351 | 1.461 | 1.000 |
| Vertical (heat-treated); scanning plane | 352 | 5.114 | 0.653 |
| Vertical (heat-treated); scanning plane | 353 | 4.383 | 0.789 |
| Vertical (heat-treated); scanning plane | 354 | 37.256 | 0.403 |
| Vertical (heat-treated); scanning plane | 355 | 9.314 | 0.602 |
| Vertical (heat-treated); scanning plane | 356 | 12.784 | 0.826 |
| Vertical (heat-treated); scanning plane | 357 | 1.461 | 0.710 |
| Vertical (heat-treated); scanning plane | 358 | 2.192 | 0.539 |
| Vertical (heat-treated); scanning plane | 359 | 0.365 | 1.000 |
| Vertical (heat-treated); scanning plane | 360 | 25.203 | 0.629 |
| Vertical (heat-treated); scanning plane | 361 | 2.009 | 0.976 |
| Vertical (heat-treated); scanning plane | 362 | 7.488 | 0.639 |
| Vertical (heat-treated); scanning plane | 363 | 0.365 | 0.785 |
| Vertical (heat-treated); scanning plane | 364 | 0.183 | 1.000 |
| Vertical (heat-treated); scanning plane | 365 | 4.200 | 0.696 |
| Vertical (heat-treated); scanning plane | 366 | 0.183 | 1.000 |
| Vertical (heat-treated); scanning plane | 367 | 0.183 | 1.000 |
| Vertical (heat-treated); scanning plane | 368 | 0.183 | 1.000 |
| Vertical (heat-treated); scanning plane | 369 | 12.419 | 0.947 |
| Vertical (heat-treated); scanning plane | 370 | 1.461 | 0.621 |
| Vertical (heat-treated); scanning plane | 371 | 0.183 | 1.000 |
| Vertical (heat-treated); scanning plane | 372 | 0.183 | 1.000 |
| Vertical (heat-treated); scanning plane | 373 | 0.731 | 1.000 |
| Vertical (heat-treated); scanning plane | 374 | 0.183 | 1.000 |
| Vertical (heat-treated); scanning plane | 375 | 0.548 | 0.809 |
| Vertical (heat-treated); scanning plane | 376 | 1.461 | 0.914 |
| Vertical (heat-treated); scanning plane | 377 | 0.183 | 1.000 |
| Vertical (heat-treated); scanning plane | 378 | 0.731 | 0.698 |
| Vertical (heat-treated); scanning plane | 379 | 1.096 | 1.000 |
| Vertical (heat-treated); scanning plane | 380 | 0.183 | 1.000 |
| Vertical (heat-treated); scanning plane | 381 | 4.018 | 0.768 |
| Vertical (heat-treated); scanning plane | 382 | 2.557 | 0.993 |
| Vertical (heat-treated); scanning plane | 383 | 2.374 | 0.697 |
| Vertical (heat-treated); scanning plane | 384 | 0.183 | 1.000 |
| Vertical (heat-treated); scanning plane | 385 | 0.548 | 1.000 |
| Vertical (heat-treated); scanning plane | 386 | 8.584 | 0.528 |
| Vertical (heat-treated); scanning plane | 387 | 18.263 | 0.857 |
| Vertical (heat-treated); scanning plane | 388 | 8.036 | 0.501 |
| Vertical (heat-treated); scanning plane | 389 | 36.891 | 0.378 |
| Vertical (heat-treated); scanning plane | 390 | 0.183 | 1.000 |
| Vertical (heat-treated); scanning plane | 391 | 1.278 | 0.898 |
| Vertical (heat-treated); scanning plane | 392 | 0.183 | 1.000 |
| Vertical (heat-treated); scanning plane | 393 | 79.078 | 0.728 |
| Vertical (heat-treated); scanning plane | 394 | 2.009 | 1.000 |
| Vertical (heat-treated); scanning plane | 395 | 0.183 | 1.000 |
| Vertical (heat-treated); scanning plane | 396 | 0.731 | 0.698 |
| Vertical (heat-treated); scanning plane | 397 | 3.105 | 0.911 |
| Vertical (heat-treated); scanning plane | 398 | 5.114 | 0.621 |
| Vertical (heat-treated); scanning plane | 399 | 1.826 | 0.358 |
| Vertical (heat-treated); scanning plane | 400 | 0.183 | 1.000 |
| Vertical (heat-treated); scanning plane | 401 | 5.479 | 0.811 |
| Vertical (heat-treated); scanning plane | 402 | 6.940 | 0.645 |
| Vertical (heat-treated); scanning plane | 403 | 0.731 | 0.698 |
| Vertical (heat-treated); scanning plane | 404 | 0.183 | 1.000 |
| Vertical (heat-treated); scanning plane | 405 | 0.365 | 1.000 |
| Vertical (heat-treated); scanning plane | 406 | 0.183 | 1.000 |
| Vertical (heat-treated); scanning plane | 407 | 0.183 | 1.000 |
| Vertical (heat-treated); scanning plane | 408 | 0.548 | 0.809 |
| Vertical (heat-treated); scanning plane | 409 | 0.548 | 0.754 |
