# Supplementary material for: Data related to the microstructural identification and analyzing the mechanical properties of maraging stainless steel 13Cr10Ni1.7Mo2Al0.4Mn0.4Si (commercially known as CX) processed by laser powder bed fusion method
Source: Data Brief. 2022 Jan 24;41:107856. doi: 10.1016/j.dib.2022.107856 (PMC8814306; doi:10.1016/j.dib.2022.107856)
Supplement: Supplementary file 1 [file mmc1.zip › Supplementary material/Table B.docx]

# **Table B.** True stress-logarithmic strain data of the specimens (Force signal in volts × 20000 = Force value in kN).

| **Sample** | **point** | **Force**  **signal**  **(V)** | **Gauge**  **diameter**  **(mm)** | **stress**  **(MPa)** | **Strain**  **(%)** |
| --- | --- | --- | --- | --- | --- |
| Vertical with raw surface (as-built) | 1 | 0 | 8 | 0 | 0 |
| Vertical with raw surface (as-built) | 2 | 0.028386 | 8 | 11.3 | 0.0051 |
| Vertical with raw surface (as-built) | 3 | 0.05679 | 8 | 22.62 | 0.0102 |
| Vertical with raw surface (as-built) | 4 | 0.085238 | 8 | 33.96 | 0.0153 |
| Vertical with raw surface (as-built) | 5 | 0.113765 | 8 | 45.32 | 0.0205 |
| Vertical with raw surface (as-built) | 6 | 0.142414 | 8 | 56.74 | 0.0256 |
| Vertical with raw surface (as-built) | 7 | 0.171226 | 8 | 68.22 | 0.0308 |
| Vertical with raw surface (as-built) | 8 | 0.200248 | 8 | 79.78 | 0.0361 |
| Vertical with raw surface (as-built) | 9 | 0.229528 | 8 | 91.44 | 0.0414 |
| Vertical with raw surface (as-built) | 10 | 0.259112 | 8 | 103.22 | 0.0468 |
| Vertical with raw surface (as-built) | 11 | 0.289041 | 8 | 115.16 | 0.0524 |
| Vertical with raw surface (as-built) | 12 | 0.319351 | 8 | 127.22 | 0.058 |
| Vertical with raw surface (as-built) | 13 | 0.35007 | 8 | 139.46 | 0.0637 |
| Vertical with raw surface (as-built) | 14 | 0.381217 | 8 | 151.88 | 0.0696 |
| Vertical with raw surface (as-built) | 15 | 0.412801 | 8 | 164.46 | 0.0756 |
| Vertical with raw surface (as-built) | 16 | 0.444823 | 8 | 177.24 | 0.0818 |
| Vertical with raw surface (as-built) | 17 | 0.477275 | 8 | 190.16 | 0.0881 |
| Vertical with raw surface (as-built) | 18 | 0.510143 | 8 | 203.26 | 0.0946 |
| Vertical with raw surface (as-built) | 19 | 0.543404 | 8 | 216.52 | 0.1012 |
| Vertical with raw surface (as-built) | 20 | 0.577034 | 8 | 229.94 | 0.108 |
| Vertical with raw surface (as-built) | 21 | 0.611 | 8 | 243.48 | 0.115 |
| Vertical with raw surface (as-built) | 22 | 0.645271 | 8 | 257.16 | 0.122 |
| Vertical with raw surface (as-built) | 23 | 0.679808 | 8 | 270.92 | 0.1293 |
| Vertical with raw surface (as-built) | 24 | 0.714573 | 8 | 284.8 | 0.1366 |
| Vertical with raw surface (as-built) | 25 | 0.749524 | 8 | 298.74 | 0.1441 |
| Vertical with raw surface (as-built) | 26 | 0.784619 | 8 | 312.74 | 0.1517 |
| Vertical with raw surface (as-built) | 27 | 0.819816 | 8 | 326.8 | 0.1595 |
| Vertical with raw surface (as-built) | 28 | 0.85507 | 8 | 340.86 | 0.1674 |
| Vertical with raw surface (as-built) | 29 | 0.890342 | 8 | 354.94 | 0.1753 |
| Vertical with raw surface (as-built) | 30 | 0.925588 | 8 | 369.02 | 0.1834 |
| Vertical with raw surface (as-built) | 31 | 0.960769 | 8 | 383.06 | 0.1916 |
| Vertical with raw surface (as-built) | 32 | 0.995847 | 8 | 397.08 | 0.1998 |
| Vertical with raw surface (as-built) | 33 | 1.030784 | 8 | 411.04 | 0.2081 |
| Vertical with raw surface (as-built) | 34 | 1.065545 | 8 | 424.92 | 0.2165 |
| Vertical with raw surface (as-built) | 35 | 1.100097 | 8 | 438.74 | 0.225 |
| Vertical with raw surface (as-built) | 36 | 1.134409 | 8 | 452.44 | 0.2335 |
| Vertical with raw surface (as-built) | 37 | 1.168451 | 8 | 466.06 | 0.242 |
| Vertical with raw surface (as-built) | 38 | 1.202194 | 8 | 479.56 | 0.2507 |
| Vertical with raw surface (as-built) | 39 | 1.235612 | 8 | 492.92 | 0.2593 |
| Vertical with raw surface (as-built) | 40 | 1.268679 | 8 | 506.14 | 0.268 |
| Vertical with raw surface (as-built) | 41 | 1.301369 | 8 | 519.22 | 0.2767 |
| Vertical with raw surface (as-built) | 42 | 1.333661 | 8 | 532.14 | 0.2855 |
| Vertical with raw surface (as-built) | 43 | 1.365534 | 8 | 544.9 | 0.2943 |
| Vertical with raw surface (as-built) | 44 | 1.396969 | 8 | 557.5 | 0.3031 |
| Vertical with raw surface (as-built) | 45 | 1.427947 | 8 | 569.9 | 0.312 |
| Vertical with raw surface (as-built) | 46 | 1.458452 | 8 | 582.12 | 0.3208 |
| Vertical with raw surface (as-built) | 47 | 1.488469 | 8 | 594.14 | 0.3297 |
| Vertical with raw surface (as-built) | 48 | 1.517982 | 8 | 605.98 | 0.3386 |
| Vertical with raw surface (as-built) | 49 | 1.54698 | 8 | 617.6 | 0.3476 |
| Vertical with raw surface (as-built) | 50 | 1.575451 | 8 | 629.02 | 0.3565 |
| Vertical with raw surface (as-built) | 51 | 1.603387 | 8 | 640.22 | 0.3655 |
| Vertical with raw surface (as-built) | 52 | 1.630779 | 8 | 651.22 | 0.3745 |
| Vertical with raw surface (as-built) | 53 | 1.657623 | 8 | 661.98 | 0.3835 |
| Vertical with raw surface (as-built) | 54 | 1.683917 | 8 | 672.54 | 0.3926 |
| Vertical with raw surface (as-built) | 55 | 1.709656 | 8 | 682.88 | 0.4016 |
| Vertical with raw surface (as-built) | 56 | 1.73484 | 8 | 693 | 0.4108 |
| Vertical with raw surface (as-built) | 57 | 1.759469 | 8 | 702.88 | 0.4199 |
| Vertical with raw surface (as-built) | 58 | 1.783544 | 8 | 712.56 | 0.4291 |
| Vertical with raw surface (as-built) | 59 | 1.807066 | 8 | 722.02 | 0.4384 |
| Vertical with raw surface (as-built) | 60 | 1.830038 | 8 | 731.26 | 0.4477 |
| Vertical with raw surface (as-built) | 61 | 1.852463 | 8 | 740.3 | 0.457 |
| Vertical with raw surface (as-built) | 62 | 1.874348 | 8 | 749.1 | 0.4665 |
| Vertical with raw surface (as-built) | 63 | 1.895699 | 8 | 757.7 | 0.4759 |
| Vertical with raw surface (as-built) | 64 | 1.916521 | 8 | 766.1 | 0.4855 |
| Vertical with raw surface (as-built) | 65 | 1.936823 | 8 | 774.28 | 0.495 |
| Vertical with raw surface (as-built) | 66 | 1.956612 | 8 | 782.26 | 0.5046 |
| Vertical with raw surface (as-built) | 67 | 1.975895 | 8 | 790.04 | 0.5143 |
| Vertical with raw surface (as-built) | 68 | 1.994682 | 8 | 797.62 | 0.524 |
| Vertical with raw surface (as-built) | 69 | 2.012981 | 8 | 805.02 | 0.5337 |
| Vertical with raw surface (as-built) | 70 | 2.030803 | 8 | 812.22 | 0.5434 |
| Vertical with raw surface (as-built) | 71 | 2.048157 | 8 | 819.24 | 0.5531 |
| Vertical with raw surface (as-built) | 72 | 2.065056 | 8 | 826.08 | 0.5628 |
| Vertical with raw surface (as-built) | 73 | 2.081512 | 8 | 832.74 | 0.5726 |
| Vertical with raw surface (as-built) | 74 | 2.097535 | 8 | 839.22 | 0.5823 |
| Vertical with raw surface (as-built) | 75 | 2.113137 | 8 | 845.54 | 0.592 |
| Vertical with raw surface (as-built) | 76 | 2.128329 | 8 | 851.7 | 0.6016 |
| Vertical with raw surface (as-built) | 77 | 2.143122 | 8 | 857.7 | 0.6113 |
| Vertical with raw surface (as-built) | 78 | 2.157526 | 8 | 863.54 | 0.6209 |
| Vertical with raw surface (as-built) | 79 | 2.171552 | 8 | 869.24 | 0.6305 |
| Vertical with raw surface (as-built) | 80 | 2.185212 | 8 | 874.78 | 0.64 |
| Vertical with raw surface (as-built) | 81 | 2.198516 | 8 | 880.2 | 0.6496 |
| Vertical with raw surface (as-built) | 82 | 2.211475 | 8 | 885.46 | 0.6591 |
| Vertical with raw surface (as-built) | 83 | 2.2241 | 8 | 890.6 | 0.6686 |
| Vertical with raw surface (as-built) | 84 | 2.236402 | 8 | 895.6 | 0.678 |
| Vertical with raw surface (as-built) | 85 | 2.248388 | 8 | 900.5 | 0.6875 |
| Vertical with raw surface (as-built) | 86 | **2.260069** | 8 | **905.26** | **0.6969** |
| Vertical with raw surface (as-built) | 87 | 2.271451 | 8 | 909.9 | 0.7063 |
| Vertical with raw surface (as-built) | 88 | 2.282543 | 8 | 914.42 | 0.7157 |
| Vertical with raw surface (as-built) | 89 | 2.29335 | 8 | 918.84 | 0.7251 |
| Vertical with raw surface (as-built) | 90 | 2.303879 | 8 | 923.14 | 0.7345 |
| Vertical with raw surface (as-built) | 91 | 2.314136 | 8 | 927.34 | 0.7438 |
| Vertical with raw surface (as-built) | 92 | 2.324127 | 8 | 931.42 | 0.7531 |
| Vertical with raw surface (as-built) | 93 | 2.333858 | 8 | 935.42 | 0.7625 |
| Vertical with raw surface (as-built) | 94 | 2.343334 | 8 | 939.3 | 0.7718 |
| Vertical with raw surface (as-built) | 95 | 2.35256 | 8 | 943.08 | 0.7812 |
| Vertical with raw surface (as-built) | 96 | 2.361543 | 8 | 946.78 | 0.7905 |
| Vertical with raw surface (as-built) | 97 | 2.370287 | 8 | 950.36 | 0.7999 |
| Vertical with raw surface (as-built) | 98 | 2.378798 | 8 | 953.86 | 0.8094 |
| Vertical with raw surface (as-built) | 99 | 2.387081 | 8 | 957.28 | 0.8188 |
| Vertical with raw surface (as-built) | 100 | 2.395142 | 8 | 960.6 | 0.8283 |
| Vertical with raw surface (as-built) | 101 | 2.402984 | 8 | 963.84 | 0.8378 |
| Vertical with raw surface (as-built) | 102 | 2.410615 | 8 | 967 | 0.8473 |
| Vertical with raw surface (as-built) | 103 | 2.41804 | 8 | 970.06 | 0.8569 |
| Vertical with raw surface (as-built) | 104 | 2.425266 | 8 | 973.06 | 0.8665 |
| Vertical with raw surface (as-built) | 105 | 2.4323 | 8 | 975.96 | 0.876 |
| Vertical with raw surface (as-built) | 106 | 2.439149 | 8 | 978.8 | 0.8856 |
| Vertical with raw surface (as-built) | 107 | 2.445819 | 8 | 981.58 | 0.8952 |
| Vertical with raw surface (as-built) | 108 | 2.452314 | 8 | 984.28 | 0.9047 |
| Vertical with raw surface (as-built) | 109 | 2.458641 | 8 | 986.92 | 0.9143 |
| Vertical with raw surface (as-built) | 110 | 2.464802 | 8 | 989.48 | 0.9238 |
| Vertical with raw surface (as-built) | 111 | 2.470803 | 8 | 991.98 | 0.9332 |
| Vertical with raw surface (as-built) | 112 | 2.476649 | 8 | 994.42 | 0.9427 |
| Vertical with raw surface (as-built) | 113 | 2.482343 | 8 | 996.8 | 0.9521 |
| Vertical with raw surface (as-built) | 114 | 2.487891 | 8 | 999.12 | 0.9615 |
| Vertical with raw surface (as-built) | 115 | 2.493297 | 8 | 1001.38 | 0.9709 |
| Vertical with raw surface (as-built) | 116 | 2.498566 | 8 | 1003.58 | 0.9802 |
| Vertical with raw surface (as-built) | 117 | 2.503702 | 8 | 1005.74 | 0.9895 |
| Vertical with raw surface (as-built) | 118 | 2.50871 | 8 | 1007.84 | 0.9989 |
| Vertical with raw surface (as-built) | 119 | 2.513592 | 8 | 1009.9 | 1.0081 |
| Vertical with raw surface (as-built) | 120 | 2.518353 | 8 | 1011.9 | 1.0174 |
| Vertical with raw surface (as-built) | 121 | 2.522998 | 8 | 1013.86 | 1.0267 |
| Vertical with raw surface (as-built) | 122 | 2.52753 | 8 | 1015.76 | 1.036 |
| Vertical with raw surface (as-built) | 123 | 2.531954 | 8 | 1017.64 | 1.0452 |
| Vertical with raw surface (as-built) | 124 | 2.536274 | 8 | 1019.46 | 1.0545 |
| Vertical with raw surface (as-built) | 125 | 2.540495 | 8 | 1021.26 | 1.0637 |
| Vertical with raw surface (as-built) | 126 | 2.54462 | 8 | 1023 | 1.0729 |
| Vertical with raw surface (as-built) | 127 | 2.548655 | 8 | 1024.72 | 1.082 |
| Vertical with raw surface (as-built) | 128 | 2.552604 | 8 | 1026.4 | 1.0912 |
| Vertical with raw surface (as-built) | 129 | 2.55647 | 8 | 1028.04 | 1.1003 |
| Vertical with raw surface (as-built) | 130 | 2.560258 | 8 | 1029.64 | 1.1093 |
| Vertical with raw surface (as-built) | 131 | 2.563972 | 8 | 1031.24 | 1.1184 |
| Vertical with raw surface (as-built) | 132 | 2.567615 | 8 | 1032.78 | 1.1273 |
| Vertical with raw surface (as-built) | 133 | 2.571189 | 8 | 1034.32 | 1.1363 |
| Vertical with raw surface (as-built) | 134 | 2.574695 | 8 | 1035.82 | 1.1452 |
| Vertical with raw surface (as-built) | 135 | 2.578132 | 8 | 1037.28 | 1.154 |
| Vertical with raw surface (as-built) | 136 | 2.581502 | 8 | 1038.72 | 1.1629 |
| Vertical with raw surface (as-built) | 137 | 2.584807 | 8 | 1040.14 | 1.1717 |
| Vertical with raw surface (as-built) | 138 | 2.588047 | 8 | 1041.54 | 1.1805 |
| Vertical with raw surface (as-built) | 139 | 2.591224 | 8 | 1042.9 | 1.1893 |
| Vertical with raw surface (as-built) | 140 | 2.594342 | 8 | 1044.24 | 1.1981 |
| Vertical with raw surface (as-built) | 141 | 2.597404 | 8 | 1045.56 | 1.2069 |
| Vertical with raw surface (as-built) | 142 | 2.600412 | 8 | 1046.86 | 1.2158 |
| Vertical with raw surface (as-built) | 143 | 2.603372 | 8 | 1048.14 | 1.2247 |
| Vertical with raw surface (as-built) | 144 | 2.606286 | 8 | 1049.4 | 1.2337 |
| Vertical with raw surface (as-built) | 145 | 2.609158 | 8 | 1050.66 | 1.2428 |
| Vertical with raw surface (as-built) | 146 | 2.611991 | 8 | 1051.88 | 1.2519 |
| Vertical with raw surface (as-built) | 147 | 2.614788 | 8 | 1053.1 | 1.2612 |
| Vertical with raw surface (as-built) | 148 | 2.61755 | 8 | 1054.32 | 1.2706 |
| Vertical with raw surface (as-built) | 149 | 2.620281 | 8 | 1055.5 | 1.2801 |
| Vertical with raw surface (as-built) | 150 | 2.622983 | 8 | 1056.7 | 1.2898 |
| Vertical with raw surface (as-built) | 151 | 2.625659 | 8 | 1057.88 | 1.2996 |
| Vertical with raw surface (as-built) | 152 | 2.628311 | 8 | 1059.04 | 1.3096 |
| Vertical with raw surface (as-built) | 153 | 2.630943 | 8 | 1060.2 | 1.3197 |
| Vertical with raw surface (as-built) | 154 | 2.633559 | 8 | 1061.36 | 1.3302 |
| Vertical with raw surface (as-built) | 155 | 2.636162 | 8 | 1062.52 | 1.3408 |
| Vertical with raw surface (as-built) | 156 | 2.638756 | 8 | 1063.68 | 1.3517 |
| Vertical with raw surface (as-built) | 157 | 2.641346 | 8 | 1064.84 | 1.363 |
| Vertical with raw surface (as-built) | 158 | 2.643933 | 8 | 1066 | 1.3745 |
| Vertical with raw surface (as-built) | 159 | 2.646521 | 8 | 1067.18 | 1.3865 |
| Vertical with raw surface (as-built) | 160 | 2.649112 | 8 | 1068.34 | 1.3988 |
| Vertical with raw surface (as-built) | 161 | 2.651708 | 8 | 1069.52 | 1.4116 |
| Vertical with raw surface (as-built) | 162 | 2.654309 | 8 | 1070.72 | 1.4248 |
| Vertical with raw surface (as-built) | 163 | 2.656919 | 8 | 1071.9 | 1.4386 |
| Vertical with raw surface (as-built) | 164 | 2.659536 | 8 | 1073.12 | 1.453 |
| Vertical with raw surface (as-built) | 165 | 2.66216 | 8 | 1074.32 | 1.4679 |
| Vertical with raw surface (as-built) | 166 | 2.664789 | 8 | 1075.56 | 1.4836 |
| Vertical with raw surface (as-built) | 167 | 2.667421 | 8 | 1076.78 | 1.5 |
| Vertical with raw surface (as-built) | 168 | 2.670051 | 8 | 1078.04 | 1.5173 |
| Vertical with raw surface (as-built) | 169 | 2.672675 | 8 | 1079.28 | 1.5354 |
| Vertical with raw surface (as-built) | 170 | 2.675286 | 8 | 1080.54 | 1.5544 |
| Vertical with raw surface (as-built) | 171 | 2.677877 | 8 | 1081.8 | 1.5743 |
| Vertical with raw surface (as-built) | 172 | 2.68044 | 8 | 1083.04 | 1.5953 |
| Vertical with raw surface (as-built) | 173 | 2.682967 | 8 | 1084.3 | 1.6174 |
| Vertical with raw surface (as-built) | 174 | 2.685448 | 8 | 1085.54 | 1.6405 |
| Vertical with raw surface (as-built) | 175 | 2.687876 | 8 | 1086.78 | 1.6648 |
| Vertical with raw surface (as-built) | 176 | 2.690242 | 8 | 1088.02 | 1.6902 |
| Vertical with raw surface (as-built) | 177 | 2.692539 | 8 | 1089.22 | 1.7168 |
| Vertical with raw surface (as-built) | 178 | 2.694762 | 8 | 1090.42 | 1.7447 |
| Vertical with raw surface (as-built) | 179 | 2.696903 | 8 | 1091.58 | 1.7737 |
| Vertical with raw surface (as-built) | 180 | 2.698957 | 8 | 1092.74 | 1.8041 |
| Vertical with raw surface (as-built) | 181 | 2.700921 | 8 | 1093.88 | 1.8358 |
| Vertical with raw surface (as-built) | 182 | 2.702789 | 8 | 1094.98 | 1.8688 |
| Vertical with raw surface (as-built) | 183 | 2.704558 | 8 | 1096.06 | 1.9031 |
| Vertical with raw surface (as-built) | 184 | 2.706225 | 8 | 1097.12 | 1.9389 |
| Vertical with raw surface (as-built) | 185 | 2.707787 | 8 | 1098.14 | 1.9761 |
| Vertical with raw surface (as-built) | 186 | 2.70924 | 8 | 1099.14 | 2.0147 |
| Vertical with raw surface (as-built) | 187 | 2.71058 | 8 | 1100.1 | 2.0548 |
| Vertical with raw surface (as-built) | 188 | 2.711805 | 8 | 1101.04 | 2.0965 |
| Vertical with raw surface (as-built) | 189 | 2.712912 | 8 | 1101.94 | 2.1396 |
| Vertical with raw surface (as-built) | 190 | 2.713899 | 8 | 1102.82 | 2.1843 |
| Vertical with raw surface (as-built) | 191 | 2.714764 | 8 | 1103.66 | 2.2306 |
| Vertical with raw surface (as-built) | 192 | 2.715509 | 8 | 1104.48 | 2.2785 |
| Vertical with raw surface (as-built) | 193 | 2.71613 | 8 | 1105.26 | 2.328 |
| Vertical with raw surface (as-built) | 194 | 2.716626 | 8 | 1106 | 2.3791 |
| Vertical with raw surface (as-built) | 195 | 2.716996 | 8 | 1106.7 | 2.4318 |
| Vertical with raw surface (as-built) | 196 | 2.717239 | 8 | 1107.38 | 2.4862 |
| Vertical with raw surface (as-built) | 197 | **2.717353** | 8 | **1108.02** | **2.5422** |
| Vertical with raw surface (as-built) | 198 | 2.717337 | 8 | 1108.62 | 2.5999 |
| Vertical with raw surface (as-built) | 199 | 2.717191 | 8 | 1109.2 | 2.6593 |
| Vertical with raw surface (as-built) | 200 | 2.716915 | 8 | 1109.74 | 2.7204 |
| Vertical with raw surface (as-built) | 201 | 2.71651 | 8 | 1110.24 | 2.7832 |
| Vertical with raw surface (as-built) | 202 | 2.715978 | 8 | 1110.7 | 2.8476 |
| Vertical with raw surface (as-built) | 203 | 2.71532 | 8 | 1111.14 | 2.9137 |
| Vertical with raw surface (as-built) | 204 | 2.714538 | 8 | 1111.54 | 2.9815 |
| Vertical with raw surface (as-built) | 205 | 2.713634 | 8 | 1111.9 | 3.0509 |
| Vertical with raw surface (as-built) | 206 | 2.71261 | 8 | 1112.24 | 3.1219 |
| Vertical with raw surface (as-built) | 207 | 2.711465 | 8 | 1112.54 | 3.1944 |
| Vertical with raw surface (as-built) | 208 | 2.710202 | 8 | 1112.8 | 3.2685 |
| Vertical with raw surface (as-built) | 209 | 2.70882 | 8 | 1113.04 | 3.3441 |
| Vertical with raw surface (as-built) | 210 | 2.707322 | 8 | 1113.24 | 3.4212 |
| Vertical with raw surface (as-built) | 211 | 2.705709 | 8 | 1113.42 | 3.4997 |
| Vertical with raw surface (as-built) | 212 | 2.703983 | 8 | 1113.54 | 3.5795 |
| Vertical with raw surface (as-built) | 213 | 2.702146 | 8 | 1113.66 | 3.6607 |
| Vertical with raw surface (as-built) | 214 | 2.700201 | 8 | 1113.72 | 3.7431 |
| Vertical with raw surface (as-built) | 215 | 2.698151 | 8 | 1113.78 | 3.8268 |
| Vertical with raw surface (as-built) | 216 | 2.696 | 8 | 1113.78 | 3.9117 |
| Vertical with raw surface (as-built) | 217 | 2.693751 | 8 | 1113.76 | 3.9977 |
| Vertical with raw surface (as-built) | 218 | 2.691409 | 8 | 1113.72 | 4.0849 |
| Vertical with raw surface (as-built) | 219 | 2.688978 | 8 | 1113.66 | 4.1731 |
| Vertical with raw surface (as-built) | 220 | 2.686462 | 8 | 1113.56 | 4.2624 |
| Vertical with raw surface (as-built) | 221 | 2.683863 | 8 | 1113.44 | 4.3526 |
| Vertical with raw surface (as-built) | 222 | 2.681186 | 8 | 1113.3 | 4.4438 |
| Vertical with raw surface (as-built) | 223 | 2.678435 | 8 | 1113.14 | 4.536 |
| Vertical with raw surface (as-built) | 224 | 2.675618 | 8 | 1112.94 | 4.6289 |
| Vertical with raw surface (as-built) | 225 | 2.672739 | 8 | 1112.74 | 4.7226 |
| Vertical with raw surface (as-built) | 226 | 2.669804 | 8 | 1112.52 | 4.8171 |
| Vertical with raw surface (as-built) | 227 | 2.66682 | 8 | 1112.3 | 4.9123 |
| Vertical with raw surface (as-built) | 228 | 2.663791 | 8 | 1112.04 | 5.008 |
| Vertical with raw surface (as-built) | 229 | 2.660722 | 8 | 1111.78 | 5.1043 |
| Vertical with raw surface (as-built) | 230 | 2.657618 | 8 | 1111.52 | 5.2012 |
| Vertical with raw surface (as-built) | 231 | 2.654481 | 8 | 1111.24 | 5.2984 |
| Vertical with raw surface (as-built) | 232 | 2.651315 | 8 | 1110.94 | 5.3961 |
| Vertical with raw surface (as-built) | 233 | 2.648123 | 8 | 1110.64 | 5.4942 |
| Vertical with raw surface (as-built) | 234 | 2.644908 | 8 | 1110.34 | 5.5926 |
| Vertical with raw surface (as-built) | 235 | 2.641673 | 8 | 1110.02 | 5.6913 |
| Vertical with raw surface (as-built) | 236 | 2.638422 | 8 | 1109.72 | 5.7902 |
| Vertical with raw surface (as-built) | 237 | 2.635157 | 8 | 1109.38 | 5.8894 |
| Vertical with raw surface (as-built) | 238 | 2.631882 | 8 | 1109.06 | 5.9887 |
| Vertical with raw surface (as-built) | 239 | 2.628599 | 8 | 1108.74 | 6.0882 |
| Vertical with raw surface (as-built) | 240 | 2.625312 | 8 | 1108.4 | 6.1879 |
| Vertical with raw surface (as-built) | 241 | 2.622022 | 8 | 1108.06 | 6.2878 |
| Vertical with raw surface (as-built) | 242 | 2.618731 | 8 | 1107.74 | 6.3878 |
| Vertical with raw surface (as-built) | 243 | 2.615442 | 8 | 1107.4 | 6.488 |
| Vertical with raw surface (as-built) | 244 | 2.612155 | 8 | 1107.08 | 6.5884 |
| Vertical with raw surface (as-built) | 245 | 2.608871 | 8 | 1106.74 | 6.6889 |
| Vertical with raw surface (as-built) | 246 | 2.605592 | 8 | 1106.42 | 6.7897 |
| Vertical with raw surface (as-built) | 247 | 2.602318 | 8 | 1106.1 | 6.8906 |
| Vertical with raw surface (as-built) | 248 | 2.599049 | 8 | 1105.78 | 6.9918 |
| Vertical with raw surface (as-built) | 249 | 2.595787 | 8 | 1105.46 | 7.0932 |
| Vertical with raw surface (as-built) | 250 | 2.592533 | 8 | 1105.14 | 7.1949 |
| Vertical with raw surface (as-built) | 251 | 2.589288 | 8 | 1104.84 | 7.2969 |
| Vertical with raw surface (as-built) | 252 | 2.586053 | 8 | 1104.52 | 7.3992 |
| Vertical with raw surface (as-built) | 253 | 2.582829 | 8 | 1104.24 | 7.5018 |
| Vertical with raw surface (as-built) | 254 | 2.579616 | 8 | 1103.94 | 7.6048 |
| Vertical with raw surface (as-built) | 255 | 2.576415 | 8 | 1103.66 | 7.7081 |
| Vertical with raw surface (as-built) | 256 | 2.573225 | 8 | 1103.38 | 7.8118 |
| Vertical with raw surface (as-built) | 257 | 2.570046 | 8 | 1103.12 | 7.9159 |
| Vertical with raw surface (as-built) | 258 | 2.566878 | 8 | 1102.86 | 8.0204 |
| Vertical with raw surface (as-built) | 259 | 2.563722 | 8 | 1102.6 | 8.1253 |
| Vertical with raw surface (as-built) | 260 | 2.560578 | 8 | 1102.36 | 8.2306 |
| Vertical with raw surface (as-built) | 261 | 2.557445 | 8 | 1102.12 | 8.3364 |
| Vertical with raw surface (as-built) | 262 | 2.554325 | 8 | 1101.88 | 8.4426 |
| Vertical with raw surface (as-built) | 263 | 2.551215 | 8 | 1101.66 | 8.5492 |
| Vertical with raw surface (as-built) | 264 | 2.548118 | 8 | 1101.46 | 8.6563 |
| Vertical with raw surface (as-built) | 265 | 2.545032 | 8 | 1101.24 | 8.7638 |
| Vertical with raw surface (as-built) | 266 | 2.541957 | 8 | 1101.04 | 8.8717 |
| Vertical with raw surface (as-built) | 267 | 2.538892 | 8 | 1100.86 | 8.9801 |
| Vertical with raw surface (as-built) | 268 | 2.535838 | 8 | 1100.68 | 9.0888 |
| Vertical with raw surface (as-built) | 269 | 2.532794 | 8 | 1100.5 | 9.198 |
| Vertical with raw surface (as-built) | 270 | 2.529761 | 8 | 1100.32 | 9.3076 |
| Vertical with raw surface (as-built) | 271 | 2.526738 | 8 | 1100.16 | 9.4176 |
| Vertical with raw surface (as-built) | 272 | 2.523727 | 8 | 1100 | 9.5279 |
| Vertical with raw surface (as-built) | 273 | 2.520726 | 8 | 1099.86 | 9.6386 |
| Vertical with raw surface (as-built) | 274 | 2.517737 | 8 | 1099.72 | 9.7497 |
| Vertical with raw surface (as-built) | 275 | 2.514759 | 8 | 1099.58 | 9.8611 |
| Vertical with raw surface (as-built) | 276 | 2.511793 | 8 | 1099.46 | 9.9728 |
| Vertical with raw surface (as-built) | 277 | 2.508839 | 8 | 1099.34 | 10.0848 |
| Vertical with raw surface (as-built) | 278 | 2.505895 | 8 | 1099.22 | 10.197 |
| Vertical with raw surface (as-built) | 279 | 2.502962 | 8 | 1099.1 | 10.3095 |
| Vertical with raw surface (as-built) | 280 | 2.500039 | 8 | 1099 | 10.4223 |
| Vertical with raw surface (as-built) | 281 | 2.497126 | 8 | 1098.9 | 10.5352 |
| Vertical with raw surface (as-built) | 282 | 2.494223 | 8 | 1098.8 | 10.6484 |
| Vertical with raw surface (as-built) | 283 | 2.491329 | 8 | 1098.72 | 10.7618 |
| Vertical with raw surface (as-built) | 284 | 2.488446 | 8 | 1098.64 | 10.8754 |
| Vertical with raw surface (as-built) | 285 | 2.485574 | 8 | 1098.56 | 10.9892 |
| Vertical with raw surface (as-built) | 286 | 2.482713 | 8 | 1098.48 | 11.1031 |
| Vertical with raw surface (as-built) | 287 | 2.479864 | 8 | 1098.42 | 11.2172 |
| Vertical with raw surface (as-built) | 288 | 2.477027 | 8 | 1098.34 | 11.3314 |
| Vertical with raw surface (as-built) | 289 | 2.474203 | 8 | 1098.3 | 11.4458 |
| Vertical with raw surface (as-built) | 290 | 2.471391 | 8 | 1098.24 | 11.5604 |
| Vertical with raw surface (as-built) | 291 | 2.468593 | 8 | 1098.2 | 11.6751 |
| Vertical with raw surface (as-built) | 292 | 2.465808 | 8 | 1098.16 | 11.7899 |
| Vertical with raw surface (as-built) | 293 | 2.463038 | 8 | 1098.12 | 11.905 |
| Vertical with raw surface (as-built) | 294 | 2.460283 | 8 | 1098.1 | 12.0202 |
| Vertical with raw surface (as-built) | 295 | 2.457543 | 8 | 1098.08 | 12.1357 |
| Vertical with raw surface (as-built) | 296 | 2.454821 | 8 | 1098.08 | 12.2514 |
| Vertical with raw surface (as-built) | 297 | 2.452116 | 8 | 1098.08 | 12.3673 |
| Vertical with raw surface (as-built) | 298 | 2.449428 | 8 | 1098.08 | 12.4834 |
| Vertical with raw surface (as-built) | 299 | 2.446757 | 8 | 1098.1 | 12.5998 |
| Vertical with raw surface (as-built) | 300 | 2.444105 | 8 | 1098.12 | 12.7163 |
| Vertical with raw surface (as-built) | 301 | 2.44147 | 8 | 1098.16 | 12.833 |
| Vertical with raw surface (as-built) | 302 | 2.438855 | 8 | 1098.2 | 12.9499 |
| Vertical with raw surface (as-built) | 303 | 2.436259 | 8 | 1098.26 | 13.067 |
| Vertical with raw surface (as-built) | 304 | 2.433681 | 8 | 1098.32 | 13.1842 |
| Vertical with raw surface (as-built) | 305 | 2.43112 | 8 | 1098.4 | 13.3015 |
| Vertical with raw surface (as-built) | 306 | 2.428575 | 8 | 1098.48 | 13.4189 |
| Vertical with raw surface (as-built) | 307 | 2.426046 | 8 | 1098.56 | 13.5363 |
| Vertical with raw surface (as-built) | 308 | 2.423531 | 8 | 1098.64 | 13.6537 |
| Vertical with raw surface (as-built) | 309 | 2.421029 | 8 | 1098.74 | 13.7712 |
| Vertical with raw surface (as-built) | 310 | 2.418541 | 8 | 1098.84 | 13.8886 |
| Vertical with raw surface (as-built) | 311 | 2.416067 | 8 | 1098.94 | 14.006 |
| Vertical with raw surface (as-built) | 312 | 2.413605 | 8 | 1099.04 | 14.1233 |
| Vertical with raw surface (as-built) | 313 | 2.411156 | 8 | 1099.16 | 14.2406 |
| Vertical with raw surface (as-built) | 314 | 2.408717 | 8 | 1099.26 | 14.3578 |
| Vertical with raw surface (as-built) | 315 | 2.406288 | 8 | 1099.38 | 14.4749 |
| Vertical with raw surface (as-built) | 316 | 2.403868 | 8 | 1099.5 | 14.5919 |
| Vertical with raw surface (as-built) | 317 | 2.401455 | 8 | 1099.62 | 14.7088 |
| Vertical with raw surface (as-built) | 318 | 2.39905 | 8 | 1099.74 | 14.8257 |
| Vertical with raw surface (as-built) | 319 | 2.396653 | 8 | 1099.86 | 14.9424 |
| Vertical with raw surface (as-built) | 320 | 2.394264 | 8 | 1099.98 | 15.0592 |
| Vertical with raw surface (as-built) | 321 | 2.391882 | 8 | 1100.12 | 15.1759 |
| Vertical with raw surface (as-built) | 322 | 2.389509 | 8 | 1100.24 | 15.2927 |
| Vertical with raw surface (as-built) | 323 | 2.387144 | 8 | 1100.38 | 15.4094 |
| Vertical with raw surface (as-built) | 324 | 2.384787 | 8 | 1100.5 | 15.5263 |
| Vertical with raw surface (as-built) | 325 | 2.382438 | 8 | 1100.64 | 15.6432 |
| Vertical with raw surface (as-built) | 326 | 2.380096 | 8 | 1100.78 | 15.7602 |
| Vertical with raw surface (as-built) | 327 | 2.377761 | 8 | 1100.94 | 15.8773 |
| Vertical with raw surface (as-built) | 328 | 2.375433 | 8 | 1101.08 | 15.9946 |
| Vertical with raw surface (as-built) | 329 | 2.37311 | 8 | 1101.24 | 16.112 |
| Vertical with raw surface (as-built) | 330 | 2.370795 | 8 | 1101.4 | 16.2297 |
| Vertical with raw surface (as-built) | 331 | 2.368487 | 8 | 1101.56 | 16.3475 |
| Vertical with raw surface (as-built) | 332 | 2.366187 | 8 | 1101.72 | 16.4656 |
| Vertical with raw surface (as-built) | 333 | 2.363896 | 8 | 1101.9 | 16.584 |
| Vertical with raw surface (as-built) | 334 | 2.361613 | 8 | 1102.06 | 16.7026 |
| Vertical with raw surface (as-built) | 335 | 2.359337 | 8 | 1102.26 | 16.8214 |
| Vertical with raw surface (as-built) | 336 | 2.357068 | 8 | 1102.44 | 16.9406 |
| Vertical with raw surface (as-built) | 337 | 2.354803 | 8 | 1102.64 | 17.0601 |
| Vertical with raw surface (as-built) | 338 | 2.352543 | 8 | 1102.82 | 17.1799 |
| Vertical with raw surface (as-built) | 339 | 2.350285 | 8 | 1103.02 | 17.3 |
| Vertical with raw surface (as-built) | 340 | 2.348028 | 8 | 1103.24 | 17.4204 |
| Vertical with raw surface (as-built) | 341 | 2.345771 | 8 | 1103.44 | 17.5412 |
| Vertical with raw surface (as-built) | 342 | 2.343514 | 8 | 1103.64 | 17.6623 |
| Vertical with raw surface (as-built) | 343 | 2.341254 | 8 | 1103.86 | 17.7837 |
| Vertical with raw surface (as-built) | 344 | 2.338993 | 8 | 1104.06 | 17.9055 |
| Vertical with raw surface (as-built) | 345 | 2.336731 | 8 | 1104.28 | 18.0276 |
| Vertical with raw surface (as-built) | 346 | 2.334468 | 8 | 1104.48 | 18.15 |
| Vertical with raw surface (as-built) | 347 | 2.332204 | 8 | 1104.7 | 18.2729 |
| Vertical with raw surface (as-built) | 348 | 2.32994 | 8 | 1104.92 | 18.3961 |
| Vertical with raw surface (as-built) | 349 | 2.327676 | 8 | 1105.14 | 18.5196 |
| Vertical with raw surface (as-built) | 350 | 2.325411 | 8 | 1105.38 | 18.6434 |
| Vertical with raw surface (as-built) | 351 | 2.323144 | 8 | 1105.6 | 18.7676 |
| Vertical with raw surface (as-built) | 352 | 2.320876 | 8 | 1105.82 | 18.8922 |
| Vertical with raw surface (as-built) | 353 | 2.318607 | 8 | 1106.06 | 19.017 |
| Vertical with raw surface (as-built) | 354 | 2.316339 | 8 | 1106.28 | 19.1421 |
| Vertical with raw surface (as-built) | 355 | 2.314074 | 8 | 1106.52 | 19.2676 |
| Vertical with raw surface (as-built) | 356 | 2.311812 | 8 | 1106.76 | 19.3933 |
| Vertical with raw surface (as-built) | 357 | 2.309556 | 8 | 1107 | 19.5193 |
| Vertical with raw surface (as-built) | 358 | 2.307307 | 8 | 1107.26 | 19.6456 |
| Vertical with raw surface (as-built) | 359 | 2.305067 | 8 | 1107.52 | 19.7722 |
| Vertical with raw surface (as-built) | 360 | 2.302836 | 8 | 1107.78 | 19.8991 |
| Vertical with raw surface (as-built) | 361 | 2.300616 | 8 | 1108.04 | 20.0263 |
| Vertical with raw surface (as-built) | 362 | 2.298407 | 8 | 1108.32 | 20.1537 |
| Vertical with raw surface (as-built) | 363 | 2.296212 | 8 | 1108.62 | 20.2815 |
| Vertical with raw surface (as-built) | 364 | 2.294031 | 8 | 1108.9 | 20.4095 |
| Vertical with raw surface (as-built) | 365 | 2.291865 | 8 | 1109.22 | 20.5377 |
| Vertical with raw surface (as-built) | 366 | 2.289712 | 8 | 1109.52 | 20.6663 |
| Vertical with raw surface (as-built) | 367 | 2.287572 | 8 | 1109.84 | 20.7951 |
| Vertical with raw surface (as-built) | 368 | 2.285443 | 8 | 1110.18 | 20.9241 |
| Vertical with raw surface (as-built) | 369 | 2.283323 | 8 | 1110.52 | 21.0535 |
| Vertical with raw surface (as-built) | 370 | 2.281212 | 8 | 1110.86 | 21.1831 |
| Vertical with raw surface (as-built) | 371 | 2.279109 | 8 | 1111.2 | 21.313 |
| Vertical with raw surface (as-built) | 372 | 2.277014 | 8 | 1111.56 | 21.4431 |
| Vertical with raw surface (as-built) | 373 | 2.274926 | 8 | 1111.92 | 21.5736 |
| Vertical with raw surface (as-built) | 374 | 2.272844 | 8 | 1112.28 | 21.7043 |
| Vertical with raw surface (as-built) | 375 | 2.270766 | 8 | 1112.64 | 21.8354 |
| Vertical with raw surface (as-built) | 376 | 2.268689 | 8 | 1113.02 | 21.9667 |
| Vertical with raw surface (as-built) | 377 | 2.266612 | 8 | 1113.38 | 22.0983 |
| Vertical with raw surface (as-built) | 378 | 2.264533 | 8 | 1113.76 | 22.2303 |
| Vertical with raw surface (as-built) | 379 | 2.262452 | 8 | 1114.14 | 22.3625 |
| Vertical with raw surface (as-built) | 380 | 2.260369 | 8 | 1114.52 | 22.4951 |
| Vertical with raw surface (as-built) | 381 | 2.258285 | 8 | 1114.88 | 22.628 |
| Vertical with raw surface (as-built) | 382 | 2.256198 | 8 | 1115.26 | 22.7612 |
| Vertical with raw surface (as-built) | 383 | 2.254108 | 8 | 1115.66 | 22.8948 |
| Vertical with raw surface (as-built) | 384 | 2.252016 | 8 | 1116.04 | 23.0287 |
| Vertical with raw surface (as-built) | 385 | 2.24992 | 8 | 1116.42 | 23.163 |
| Vertical with raw surface (as-built) | 386 | 2.24782 | 8 | 1116.8 | 23.2976 |
| Vertical with raw surface (as-built) | 387 | 2.245716 | 8 | 1117.18 | 23.4326 |
| Vertical with raw surface (as-built) | 388 | 2.243607 | 8 | 1117.58 | 23.568 |
| Vertical with raw surface (as-built) | 389 | 2.241495 | 8 | 1117.96 | 23.7038 |
| Vertical with raw surface (as-built) | 390 | 2.23938 | 8 | 1118.36 | 23.84 |
| Vertical with raw surface (as-built) | 391 | 2.237262 | 8 | 1118.74 | 23.9766 |
| Vertical with raw surface (as-built) | 392 | 2.235143 | 8 | 1119.14 | 24.1136 |
| Vertical with raw surface (as-built) | 393 | 2.233022 | 8 | 1119.54 | 24.251 |
| Vertical with raw surface (as-built) | 394 | 2.230901 | 8 | 1119.94 | 24.3888 |
| Vertical with raw surface (as-built) | 395 | 2.228779 | 8 | 1120.34 | 24.5269 |
| Vertical with raw surface (as-built) | 396 | 2.226657 | 8 | 1120.74 | 24.6655 |
| Vertical with raw surface (as-built) | 397 | 2.224535 | 8 | 1121.16 | 24.8044 |
| Vertical with raw surface (as-built) | 398 | 2.222412 | 8 | 1121.56 | 24.9437 |
| Vertical with raw surface (as-built) | 399 | 2.220288 | 8 | 1121.98 | 25.0834 |
| Vertical with raw surface (as-built) | 400 | 2.218164 | 8 | 1122.4 | 25.2234 |
| Vertical with raw surface (as-built) | 401 | 2.216039 | 8 | 1122.82 | 25.3637 |
| Vertical with raw surface (as-built) | 402 | 2.213914 | 8 | 1123.24 | 25.5043 |
| Vertical with raw surface (as-built) | 403 | 2.211787 | 8 | 1123.66 | 25.6453 |
| Vertical with raw surface (as-built) | 404 | 2.209661 | 8 | 1124.08 | 25.7865 |
| Vertical with raw surface (as-built) | 405 | 2.207533 | 8 | 1124.52 | 25.9281 |
| Vertical with raw surface (as-built) | 406 | 2.205403 | 8 | 1124.94 | 26.0701 |
| Vertical with raw surface (as-built) | 407 | 2.203272 | 8 | 1125.38 | 26.2123 |
| Vertical with raw surface (as-built) | 408 | 2.201138 | 8 | 1125.82 | 26.355 |
| Vertical with raw surface (as-built) | 409 | 2.199001 | 8 | 1126.24 | 26.498 |
| Vertical with raw surface (as-built) | 410 | 2.196861 | 8 | 1126.68 | 26.6414 |
| Vertical with raw surface (as-built) | 411 | 2.194718 | 8 | 1127.12 | 26.7851 |
| Vertical with raw surface (as-built) | 412 | 2.192572 | 8 | 1127.56 | 26.9293 |
| Vertical with raw surface (as-built) | 413 | 2.190425 | 8 | 1128 | 27.0738 |
| Vertical with raw surface (as-built) | 414 | 2.188276 | 8 | 1128.44 | 27.2188 |
| Vertical with raw surface (as-built) | 415 | 2.186127 | 8 | 1128.9 | 27.3641 |
| Vertical with raw surface (as-built) | 416 | 2.183978 | 8 | 1129.34 | 27.5099 |
| Vertical with raw surface (as-built) | 417 | 2.18183 | 8 | 1129.8 | 27.6561 |
| Vertical with raw surface (as-built) | 418 | 2.179682 | 8 | 1130.26 | 27.8027 |
| Vertical with raw surface (as-built) | 419 | 2.177536 | 8 | 1130.72 | 27.9497 |
| Vertical with raw surface (as-built) | 420 | 2.17539 | 8 | 1131.18 | 28.097 |
| Vertical with raw surface (as-built) | 421 | 2.173246 | 8 | 1131.66 | 28.2447 |
| Vertical with raw surface (as-built) | 422 | 2.171104 | 8 | 1132.14 | 28.3927 |
| Vertical with raw surface (as-built) | 423 | 2.168965 | 8 | 1132.6 | 28.5409 |
| Vertical with raw surface (as-built) | 424 | 2.166831 | 8 | 1133.1 | 28.6894 |
| Vertical with raw surface (as-built) | 425 | 2.164701 | 8 | 1133.58 | 28.8382 |
| Vertical with raw surface (as-built) | 426 | 2.162576 | 8 | 1134.06 | 28.9872 |
| Vertical with raw surface (as-built) | 427 | 2.160457 | 8 | 1134.56 | 29.1364 |
| Vertical with raw surface (as-built) | 428 | 2.158343 | 8 | 1135.06 | 29.2857 |
| Vertical with raw surface (as-built) | 429 | 2.156234 | 8 | 1135.56 | 29.4353 |
| Vertical with raw surface (as-built) | 430 | 2.15413 | 8 | 1136.06 | 29.585 |
| Vertical with raw surface (as-built) | 431 | 2.152032 | 8 | 1136.58 | 29.7349 |
| Vertical with raw surface (as-built) | 432 | 2.149939 | 8 | 1137.08 | 29.8849 |
| Vertical with raw surface (as-built) | 433 | 2.147851 | 8 | 1137.6 | 30.0351 |
| Vertical with raw surface (as-built) | 434 | 2.145766 | 8 | 1138.12 | 30.1855 |
| Vertical with raw surface (as-built) | 435 | 2.143685 | 8 | 1138.64 | 30.336 |
| Vertical with raw surface (as-built) | 436 | 2.141604 | 8 | 1139.16 | 30.4866 |
| Vertical with raw surface (as-built) | 437 | 2.139525 | 8 | 1139.68 | 30.6374 |
| Vertical with raw surface (as-built) | 438 | 2.137445 | 8 | 1140.22 | 30.7884 |
| Vertical with raw surface (as-built) | 439 | 2.135366 | 8 | 1140.74 | 30.9396 |
| Vertical with raw surface (as-built) | 440 | 2.133285 | 8 | 1141.26 | 31.091 |
| Vertical with raw surface (as-built) | 441 | 2.131204 | 8 | 1141.8 | 31.2427 |
| Vertical with raw surface (as-built) | 442 | 2.129123 | 8 | 1142.32 | 31.3948 |
| Vertical with raw surface (as-built) | 443 | 2.12704 | 8 | 1142.86 | 31.5472 |
| Vertical with raw surface (as-built) | 444 | 2.124957 | 8 | 1143.4 | 31.7 |
| Vertical with raw surface (as-built) | 445 | 2.122871 | 8 | 1143.94 | 31.8533 |
| Vertical with raw surface (as-built) | 446 | 2.120783 | 8 | 1144.48 | 32.007 |
| Vertical with raw surface (as-built) | 447 | 2.118693 | 8 | 1145.02 | 32.1613 |
| Vertical with raw surface (as-built) | 448 | 2.116598 | 8 | 1145.56 | 32.3161 |
| Vertical with raw surface (as-built) | 449 | 2.1145 | 8 | 1146.12 | 32.4715 |
| Vertical with raw surface (as-built) | 450 | 2.112396 | 8 | 1146.68 | 32.6274 |
| Vertical with raw surface (as-built) | 451 | 2.110287 | 8 | 1147.22 | 32.7839 |
| Vertical with raw surface (as-built) | 452 | 2.108173 | 8 | 1147.78 | 32.941 |
| Vertical with raw surface (as-built) | 453 | 2.106053 | 8 | 1148.34 | 33.0987 |
| Vertical with raw surface (as-built) | 454 | 2.103927 | 8 | 1148.9 | 33.257 |
| Vertical with raw surface (as-built) | 455 | 2.101796 | 8 | 1149.48 | 33.416 |
| Vertical with raw surface (as-built) | 456 | 2.099659 | 8 | 1150.04 | 33.5756 |
| Vertical with raw surface (as-built) | 457 | 2.097515 | 8 | 1150.62 | 33.7359 |
| Vertical with raw surface (as-built) | 458 | 2.095363 | 8 | 1151.2 | 33.8968 |
| Vertical with raw surface (as-built) | 459 | 2.093203 | 8 | 1151.76 | 34.0584 |
| Vertical with raw surface (as-built) | 460 | 2.091035 | 8 | 1152.34 | 34.2206 |
| Vertical with raw surface (as-built) | 461 | 2.088859 | 8 | 1152.92 | 34.3834 |
| Vertical with raw surface (as-built) | 462 | 2.086674 | 8 | 1153.5 | 34.5469 |
| Vertical with raw surface (as-built) | 463 | 2.084479 | 8 | 1154.08 | 34.7111 |
| Vertical with raw surface (as-built) | 464 | 2.082275 | 8 | 1154.66 | 34.8759 |
| Vertical with raw surface (as-built) | 465 | 2.080064 | 8 | 1155.24 | 35.0414 |
| Vertical with raw surface (as-built) | 466 | 2.077845 | 8 | 1155.82 | 35.2075 |
| Vertical with raw surface (as-built) | 467 | 2.075622 | 8 | 1156.42 | 35.3742 |
| Vertical with raw surface (as-built) | 468 | 2.073393 | 8 | 1157 | 35.5416 |
| Vertical with raw surface (as-built) | 469 | 2.07116 | 8 | 1157.6 | 35.7095 |
| Vertical with raw surface (as-built) | 470 | 2.068922 | 8 | 1158.2 | 35.878 |
| Vertical with raw surface (as-built) | 471 | 2.066681 | 8 | 1158.8 | 36.0471 |
| Vertical with raw surface (as-built) | 472 | 2.064435 | 8 | 1159.4 | 36.2168 |
| Vertical with raw surface (as-built) | 473 | 2.062186 | 8 | 1160 | 36.387 |
| Vertical with raw surface (as-built) | 474 | 2.059933 | 8 | 1160.6 | 36.5576 |
| Vertical with raw surface (as-built) | 475 | 2.057678 | 8 | 1161.22 | 36.7288 |
| Vertical with raw surface (as-built) | 476 | 2.05542 | 8 | 1161.84 | 36.9005 |
| Vertical with raw surface (as-built) | 477 | 2.053158 | 8 | 1162.44 | 37.0726 |
| Vertical with raw surface (as-built) | 478 | 2.050893 | 8 | 1163.06 | 37.2452 |
| Vertical with raw surface (as-built) | 479 | 2.048625 | 8 | 1163.68 | 37.4183 |
| Vertical with raw surface (as-built) | 480 | 2.046353 | 8 | 1164.3 | 37.5918 |
| Vertical with raw surface (as-built) | 481 | 2.044078 | 8 | 1164.92 | 37.7657 |
| Vertical with raw surface (as-built) | 482 | 2.041801 | 8 | 1165.56 | 37.9401 |
| Vertical with raw surface (as-built) | 483 | 2.039522 | 8 | 1166.18 | 38.1149 |
| Vertical with raw surface (as-built) | 484 | 2.037244 | 8 | 1166.82 | 38.2902 |
| Vertical with raw surface (as-built) | 485 | 2.034969 | 8 | 1167.46 | 38.466 |
| Vertical with raw surface (as-built) | 486 | 2.032698 | 8 | 1168.1 | 38.6423 |
| Vertical with raw surface (as-built) | 487 | 2.030434 | 8 | 1168.76 | 38.8191 |
| Vertical with raw surface (as-built) | 488 | 2.028176 | 8 | 1169.42 | 38.9965 |
| Vertical with raw surface (as-built) | 489 | 2.025925 | 8 | 1170.08 | 39.1743 |
| Vertical with raw surface (as-built) | 490 | 2.023683 | 8 | 1170.76 | 39.3528 |
| Vertical with raw surface (as-built) | 491 | 2.021446 | 8 | 1171.46 | 39.5318 |
| Vertical with raw surface (as-built) | 492 | 2.019216 | 8 | 1172.16 | 39.7113 |
| Vertical with raw surface (as-built) | 493 | 2.016991 | 8 | 1172.86 | 39.8914 |
| Vertical with raw surface (as-built) | 494 | 2.01477 | 8 | 1173.58 | 40.0721 |
| Vertical with raw surface (as-built) | 495 | 2.012549 | 8 | 1174.3 | 40.2534 |
| Vertical with raw surface (as-built) | 496 | 2.010328 | 8 | 1175.02 | 40.4351 |
| Vertical with raw surface (as-built) | 497 | 2.008104 | 8 | 1175.76 | 40.6174 |
| Vertical with raw surface (as-built) | 498 | 2.005876 | 8 | 1176.48 | 40.8003 |
| Vertical with raw surface (as-built) | 499 | 2.003642 | 8 | 1177.22 | 40.9836 |
| Vertical with raw surface (as-built) | 500 | 2.001401 | 8 | 1177.94 | 41.1675 |
| Vertical with raw surface (as-built) | 501 | 1.999153 | 8 | 1178.68 | 41.3518 |
| Vertical with raw surface (as-built) | 502 | 1.996898 | 8 | 1179.4 | 41.5367 |
| Vertical with raw surface (as-built) | 503 | 1.994635 | 8 | 1180.14 | 41.722 |
| Vertical with raw surface (as-built) | 504 | 1.992365 | 8 | 1180.88 | 41.9078 |
| Vertical with raw surface (as-built) | 505 | 1.990087 | 8 | 1181.6 | 42.0941 |
| Vertical with raw surface (as-built) | 506 | 1.987801 | 8 | 1182.34 | 42.281 |
| Vertical with raw surface (as-built) | 507 | 1.985504 | 8 | 1183.06 | 42.4684 |
| Vertical with raw surface (as-built) | 508 | 1.983198 | 8 | 1183.78 | 42.6563 |
| Vertical with raw surface (as-built) | 509 | 1.98088 | 8 | 1184.52 | 42.8447 |
| Vertical with raw surface (as-built) | 510 | 1.978551 | 8 | 1185.24 | 43.0337 |
| Vertical with raw surface (as-built) | 511 | 1.97621 | 8 | 1185.96 | 43.2232 |
| Vertical with raw surface (as-built) | 512 | 1.973858 | 8 | 1186.68 | 43.4133 |
| Vertical with raw surface (as-built) | 513 | 1.971495 | 8 | 1187.4 | 43.6039 |
| Vertical with raw surface (as-built) | 514 | 1.969121 | 8 | 1188.12 | 43.7951 |
| Vertical with raw surface (as-built) | 515 | 1.966737 | 8 | 1188.82 | 43.9869 |
| Vertical with raw surface (as-built) | 516 | 1.964341 | 8 | 1189.54 | 44.1794 |
| Vertical with raw surface (as-built) | 517 | 1.961935 | 8 | 1190.26 | 44.3726 |
| Vertical with raw surface (as-built) | 518 | 1.959517 | 8 | 1190.96 | 44.5665 |
| Vertical with raw surface (as-built) | 519 | 1.957088 | 8 | 1191.68 | 44.7612 |
| Vertical with raw surface (as-built) | 520 | 1.954648 | 8 | 1192.4 | 44.9568 |
| Vertical with raw surface (as-built) | 521 | 1.952198 | 8 | 1193.12 | 45.1531 |
| Vertical with raw surface (as-built) | 522 | 1.949739 | 8 | 1193.84 | 45.3504 |
| Vertical with raw surface (as-built) | 523 | 1.94727 | 8 | 1194.56 | 45.5485 |
| Vertical with raw surface (as-built) | 524 | 1.944793 | 8 | 1195.28 | 45.7474 |
| Vertical with raw surface (as-built) | 525 | 1.942308 | 8 | 1196 | 45.9473 |
| Vertical with raw surface (as-built) | 526 | 1.939816 | 8 | 1196.74 | 46.148 |
| Vertical with raw surface (as-built) | 527 | 1.937318 | 8 | 1197.48 | 46.3495 |
| Vertical with raw surface (as-built) | 528 | 1.934813 | 8 | 1198.22 | 46.552 |
| Vertical with raw surface (as-built) | 529 | 1.932302 | 8 | 1198.96 | 46.7553 |
| Vertical with raw surface (as-built) | 530 | 1.929785 | 8 | 1199.72 | 46.9595 |
| Vertical with raw surface (as-built) | 531 | 1.927262 | 8 | 1200.46 | 47.1645 |
| Vertical with raw surface (as-built) | 532 | 1.924732 | 8 | 1201.22 | 47.3702 |
| Vertical with raw surface (as-built) | 533 | 1.922198 | 8 | 1201.98 | 47.5768 |
| Vertical with raw surface (as-built) | 534 | 1.919659 | 8 | 1202.76 | 47.784 |
| Vertical with raw surface (as-built) | 535 | 1.917118 | 8 | 1203.52 | 47.992 |
| Vertical with raw surface (as-built) | 536 | 1.914575 | 8 | 1204.3 | 48.2007 |
| Vertical with raw surface (as-built) | 537 | 1.912032 | 8 | 1205.08 | 48.41 |
| Vertical with raw surface (as-built) | 538 | 1.909488 | 8 | 1205.86 | 48.6199 |
| Vertical with raw surface (as-built) | 539 | 1.906946 | 8 | 1206.66 | 48.8304 |
| Vertical with raw surface (as-built) | 540 | 1.904406 | 8 | 1207.46 | 49.0415 |
| Vertical with raw surface (as-built) | 541 | 1.901869 | 8 | 1208.26 | 49.2531 |
| Vertical with raw surface (as-built) | 542 | 1.899337 | 8 | 1209.06 | 49.4653 |
| Vertical with raw surface (as-built) | 543 | 1.896811 | 8 | 1209.88 | 49.6779 |
| Vertical with raw surface (as-built) | 544 | 1.894291 | 8 | 1210.72 | 49.891 |
| Vertical with raw surface (as-built) | 545 | 1.891778 | 8 | 1211.54 | 50.1045 |
| Vertical with raw surface (as-built) | 546 | 1.88927 | 8 | 1212.38 | 50.3185 |
| Vertical with raw surface (as-built) | 547 | 1.886768 | 8 | 1213.24 | 50.5328 |
| Vertical with raw surface (as-built) | 548 | 1.88427 | 8 | 1214.08 | 50.7475 |
| Vertical with raw surface (as-built) | 549 | 1.881776 | 8 | 1214.94 | 50.9626 |
| Vertical with raw surface (as-built) | 550 | 1.879287 | 8 | 1215.82 | 51.1781 |
| Vertical with raw surface (as-built) | 551 | 1.876802 | 8 | 1216.68 | 51.3939 |
| Vertical with raw surface (as-built) | 552 | 1.874319 | 8 | 1217.56 | 51.6101 |
| Vertical with raw surface (as-built) | 553 | 1.871839 | 8 | 1218.44 | 51.8267 |
| Vertical with raw surface (as-built) | 554 | 1.869358 | 8 | 1219.32 | 52.0438 |
| Vertical with raw surface (as-built) | 555 | 1.866875 | 8 | 1220.2 | 52.2614 |
| Vertical with raw surface (as-built) | 556 | 1.864387 | 8 | 1221.08 | 52.4794 |
| Vertical with raw surface (as-built) | 557 | 1.861893 | 8 | 1221.96 | 52.6981 |
| Vertical with raw surface (as-built) | 558 | 1.859392 | 8 | 1222.84 | 52.9173 |
| Vertical with raw surface (as-built) | 559 | 1.856884 | 8 | 1223.74 | 53.1371 |
| Vertical with raw surface (as-built) | 560 | 1.854367 | 8 | 1224.62 | 53.3576 |
| Vertical with raw surface (as-built) | 561 | 1.851842 | 8 | 1225.5 | 53.5787 |
| Vertical with raw surface (as-built) | 562 | 1.849309 | 8 | 1226.38 | 53.8005 |
| Vertical with raw surface (as-built) | 563 | 1.846768 | 8 | 1227.28 | 54.023 |
| Vertical with raw surface (as-built) | 564 | 1.84422 | 8 | 1228.16 | 54.2463 |
| Vertical with raw surface (as-built) | 565 | 1.841663 | 8 | 1229.04 | 54.4703 |
| Vertical with raw surface (as-built) | 566 | 1.839097 | 8 | 1229.94 | 54.6951 |
| Vertical with raw surface (as-built) | 567 | 1.836521 | 8 | 1230.82 | 54.9207 |
| Vertical with raw surface (as-built) | 568 | 1.833934 | 8 | 1231.72 | 55.147 |
| Vertical with raw surface (as-built) | 569 | 1.831335 | 8 | 1232.6 | 55.3742 |
| Vertical with raw surface (as-built) | 570 | 1.828724 | 8 | 1233.5 | 55.6021 |
| Vertical with raw surface (as-built) | 571 | 1.8261 | 8 | 1234.38 | 55.8309 |
| Vertical with raw surface (as-built) | 572 | 1.823463 | 8 | 1235.26 | 56.0606 |
| Vertical with raw surface (as-built) | 573 | 1.820812 | 8 | 1236.14 | 56.291 |
| Vertical with raw surface (as-built) | 574 | 1.818148 | 8 | 1237.02 | 56.5224 |
| Vertical with raw surface (as-built) | 575 | 1.815471 | 8 | 1237.9 | 56.7548 |
| Vertical with raw surface (as-built) | 576 | 1.81278 | 8 | 1238.8 | 56.9881 |
| Vertical with raw surface (as-built) | 577 | 1.810074 | 8 | 1239.68 | 57.2224 |
| Vertical with raw surface (as-built) | 578 | 1.807351 | 8 | 1240.56 | 57.4577 |
| Vertical with raw surface (as-built) | 579 | 1.804612 | 8 | 1241.42 | 57.6941 |
| Vertical with raw surface (as-built) | 580 | 1.801855 | 8 | 1242.3 | 57.9316 |
| Vertical with raw surface (as-built) | 581 | 1.79908 | 8 | 1243.18 | 58.1702 |
| Vertical with raw surface (as-built) | 582 | 1.796288 | 8 | 1244.04 | 58.41 |
| Vertical with raw surface (as-built) | 583 | 1.793477 | 8 | 1244.92 | 58.6509 |
| Vertical with raw surface (as-built) | 584 | 1.790651 | 8 | 1245.78 | 58.8929 |
| Vertical with raw surface (as-built) | 585 | 1.78781 | 8 | 1246.66 | 59.1361 |
| Vertical with raw surface (as-built) | 586 | 1.784956 | 8 | 1247.54 | 59.3804 |
| Vertical with raw surface (as-built) | 587 | 1.782092 | 8 | 1248.4 | 59.6259 |
| Vertical with raw surface (as-built) | 588 | 1.779216 | 8 | 1249.28 | 59.8725 |
| Vertical with raw surface (as-built) | 589 | 1.776332 | 8 | 1250.16 | 60.1202 |
| Vertical with raw surface (as-built) | 590 | 1.773437 | 8 | 1251.06 | 60.369 |
| Vertical with raw surface (as-built) | 591 | 1.770533 | 8 | 1251.94 | 60.619 |
| Vertical with raw surface (as-built) | 592 | 1.76762 | 8 | 1252.84 | 60.87 |
| Vertical with raw surface (as-built) | 593 | 1.764697 | 8 | 1253.74 | 61.1222 |
| Vertical with raw surface (as-built) | 594 | 1.761765 | 8 | 1254.64 | 61.3754 |
| Vertical with raw surface (as-built) | 595 | 1.758824 | 8 | 1255.54 | 61.6298 |
| Vertical with raw surface (as-built) | 596 | 1.755875 | 8 | 1256.44 | 61.8853 |
| Vertical with raw surface (as-built) | 597 | 1.75292 | 8 | 1257.36 | 62.1419 |
| Vertical with raw surface (as-built) | 598 | 1.749958 | 8 | 1258.28 | 62.3997 |
| Vertical with raw surface (as-built) | 599 | 1.746991 | 8 | 1259.2 | 62.6587 |
| Vertical with raw surface (as-built) | 600 | 1.74402 | 8 | 1260.14 | 62.9188 |
| Vertical with raw surface (as-built) | 601 | 1.741043 | 8 | 1261.08 | 63.1802 |
| Vertical with raw surface (as-built) | 602 | 1.738062 | 8 | 1262.02 | 63.4427 |
| Vertical with raw surface (as-built) | 603 | 1.735078 | 8 | 1262.98 | 63.7064 |
| Vertical with raw surface (as-built) | 604 | 1.73209 | 8 | 1263.96 | 63.9714 |
| Vertical with raw surface (as-built) | 605 | 1.7291 | 8 | 1264.92 | 64.2376 |
| Vertical with raw surface (as-built) | 606 | 1.726111 | 8 | 1265.92 | 64.5051 |
| Vertical with raw surface (as-built) | 607 | 1.723122 | 8 | 1266.92 | 64.7737 |
| Vertical with raw surface (as-built) | 608 | 1.720134 | 8 | 1267.92 | 65.0434 |
| Vertical with raw surface (as-built) | 609 | 1.717149 | 8 | 1268.96 | 65.3143 |
| Vertical with raw surface (as-built) | 610 | 1.714166 | 8 | 1269.98 | 65.5862 |
| Vertical with raw surface (as-built) | 611 | 1.711186 | 8 | 1271.04 | 65.8591 |
| Vertical with raw surface (as-built) | 612 | 1.708209 | 8 | 1272.08 | 66.133 |
| Vertical with raw surface (as-built) | 613 | 1.705235 | 8 | 1273.16 | 66.4079 |
| Vertical with raw surface (as-built) | 614 | 1.702264 | 8 | 1274.22 | 66.6837 |
| Vertical with raw surface (as-built) | 615 | 1.699294 | 8 | 1275.32 | 66.9604 |
| Vertical with raw surface (as-built) | 616 | 1.696326 | 8 | 1276.4 | 67.238 |
| Vertical with raw surface (as-built) | 617 | 1.693358 | 8 | 1277.52 | 67.5164 |
| Vertical with raw surface (as-built) | 618 | 1.690391 | 8 | 1278.62 | 67.7958 |
| Vertical with raw surface (as-built) | 619 | 1.687424 | 8 | 1279.74 | 68.0761 |
| Vertical with raw surface (as-built) | 620 | 1.684458 | 8 | 1280.86 | 68.3574 |
| Vertical with raw surface (as-built) | 621 | 1.681493 | 8 | 1282 | 68.6395 |
| Vertical with raw surface (as-built) | 622 | 1.678529 | 8 | 1283.14 | 68.9226 |
| Vertical with raw surface (as-built) | 623 | 1.675566 | 8 | 1284.3 | 69.2066 |
| Vertical with raw surface (as-built) | 624 | 1.672603 | 8 | 1285.46 | 69.4917 |
| Vertical with raw surface (as-built) | 625 | 1.669638 | 8 | 1286.64 | 69.7777 |
| Vertical with raw surface (as-built) | 626 | 1.666672 | 8 | 1287.82 | 70.0649 |
| Vertical with raw surface (as-built) | 627 | 1.663701 | 8 | 1289 | 70.3532 |
| Vertical with raw surface (as-built) | 628 | 1.660727 | 8 | 1290.2 | 70.6428 |
| Vertical with raw surface (as-built) | 629 | 1.657747 | 8 | 1291.42 | 70.9336 |
| Vertical with raw surface (as-built) | 630 | 1.65476 | 8 | 1292.62 | 71.2257 |
| Vertical with raw surface (as-built) | 631 | 1.651768 | 8 | 1293.86 | 71.5191 |
| Vertical with raw surface (as-built) | 632 | 1.64877 | 8 | 1295.1 | 71.814 |
| Vertical with raw surface (as-built) | 633 | 1.645765 | 8 | 1296.34 | 72.1103 |
| Vertical with raw surface (as-built) | 634 | 1.642756 | 8 | 1297.62 | 72.4081 |
| Vertical with raw surface (as-built) | 635 | 1.639741 | 8 | 1298.88 | 72.7072 |
| Vertical with raw surface (as-built) | 636 | 1.636719 | 8 | 1300.18 | 73.0078 |
| Vertical with raw surface (as-built) | 637 | 1.633692 | 8 | 1301.48 | 73.3098 |
| Vertical with raw surface (as-built) | 638 | 1.630658 | 8 | 1302.78 | 73.6132 |
| Vertical with raw surface (as-built) | 639 | 1.62762 | 8 | 1304.12 | 73.9178 |
| Vertical with raw surface (as-built) | 640 | 1.624577 | 8 | 1305.46 | 74.2237 |
| Vertical with raw surface (as-built) | 641 | 1.621534 | 8 | 1306.8 | 74.5309 |
| Vertical with raw surface (as-built) | 642 | 1.618491 | 8 | 1308.18 | 74.8394 |
| Vertical with raw surface (as-built) | 643 | 1.615452 | 8 | 1309.56 | 75.149 |
| Vertical with raw surface (as-built) | 644 | 1.612421 | 8 | 1310.98 | 75.4598 |
| Vertical with raw surface (as-built) | 645 | 1.609399 | 8 | 1312.42 | 75.7718 |
| Vertical with raw surface (as-built) | 646 | 1.606391 | 8 | 1313.88 | 76.0849 |
| Vertical with raw surface (as-built) | 647 | 1.6034 | 8 | 1315.38 | 76.3992 |
| Vertical with raw surface (as-built) | 648 | 1.600427 | 8 | 1316.9 | 76.7146 |
| Vertical with raw surface (as-built) | 649 | 1.597474 | 8 | 1318.46 | 77.0311 |
| Vertical with raw surface (as-built) | 650 | 1.594543 | 8 | 1320.06 | 77.3486 |
| Vertical with raw surface (as-built) | 651 | 1.591633 | 8 | 1321.68 | 77.667 |
| Vertical with raw surface (as-built) | 652 | 1.588746 | 8 | 1323.34 | 77.9865 |
| Vertical with raw surface (as-built) | 653 | 1.58588 | 8 | 1325.02 | 78.3068 |
| Vertical with raw surface (as-built) | 654 | 1.583035 | 8 | 1326.74 | 78.6279 |
| Vertical with raw surface (as-built) | 655 | 1.580212 | 8 | 1328.48 | 78.9499 |
| Vertical with raw surface (as-built) | 656 | 1.577408 | 8 | 1330.26 | 79.2726 |
| Vertical with raw surface (as-built) | 657 | 1.574622 | 8 | 1332.04 | 79.596 |
| Vertical with raw surface (as-built) | 658 | 1.571852 | 8 | 1333.86 | 79.9202 |
| Vertical with raw surface (as-built) | 659 | 1.569094 | 8 | 1335.7 | 80.245 |
| Vertical with raw surface (as-built) | 660 | 1.566344 | 8 | 1337.54 | 80.5705 |
| Vertical with raw surface (as-built) | 661 | 1.563597 | 8 | 1339.38 | 80.8966 |
| Vertical with raw surface (as-built) | 662 | 1.560846 | 8 | 1341.22 | 81.2232 |
| Vertical with raw surface (as-built) | 663 | 1.558083 | 8 | 1343.04 | 81.5504 |
| Vertical with raw surface (as-built) | 664 | 1.555302 | 8 | 1344.86 | 81.878 |
| Vertical with raw surface (as-built) | 665 | 1.55249 | 8 | 1346.62 | 82.2062 |
| Vertical with raw surface (as-built) | 666 | 1.549638 | 8 | 1348.36 | 82.5348 |
| Vertical with raw surface (as-built) | 667 | 1.54673 | 8 | 1350.02 | 82.8638 |
| Vertical with raw surface (as-built) | 668 | 1.543752 | 8 | 1351.62 | 83.1934 |
| Vertical with raw surface (as-built) | 669 | 1.540686 | 8 | 1353.12 | 83.5234 |
| Vertical with raw surface (as-built) | 670 | 1.537514 | 8 | 1354.5 | 83.8539 |
| Vertical with raw surface (as-built) | 671 | 1.534216 | 8 | 1355.74 | 84.185 |
| Vertical with raw surface (as-built) | 672 | 1.53077 | 8 | 1356.84 | 84.5169 |
| Vertical with raw surface (as-built) | 673 | 1.527155 | 8 | 1357.76 | 84.8496 |
| Vertical with raw surface (as-built) | 674 | 1.523347 | 8 | 1358.48 | 85.1835 |
| Vertical with raw surface (as-built) | 675 | 1.519319 | 8 | 1358.98 | 85.5187 |
| Vertical with machined surface (as-built) | 1 | 0 | 6 | 0 | 0 |
| Vertical with machined surface (as-built) | 2 | 0.015646 | 6 | 11.08 | 0.0034 |
| Vertical with machined surface (as-built) | 3 | 0.031305 | 6 | 22.18 | 0.0068 |
| Vertical with machined surface (as-built) | 4 | 0.046997 | 6 | 33.28 | 0.0101 |
| Vertical with machined surface (as-built) | 5 | 0.062746 | 6 | 44.44 | 0.0135 |
| Vertical with machined surface (as-built) | 6 | 0.078582 | 6 | 55.66 | 0.0168 |
| Vertical with machined surface (as-built) | 7 | 0.094535 | 6 | 66.96 | 0.0201 |
| Vertical with machined surface (as-built) | 8 | 0.110641 | 6 | 78.36 | 0.0233 |
| Vertical with machined surface (as-built) | 9 | 0.126933 | 6 | 89.9 | 0.0266 |
| Vertical with machined surface (as-built) | 10 | 0.143443 | 6 | 101.6 | 0.0299 |
| Vertical with machined surface (as-built) | 11 | 0.160201 | 6 | 113.46 | 0.0333 |
| Vertical with machined surface (as-built) | 12 | 0.177231 | 6 | 125.52 | 0.0369 |
| Vertical with machined surface (as-built) | 13 | 0.19455 | 6 | 137.8 | 0.0407 |
| Vertical with machined surface (as-built) | 14 | 0.212171 | 6 | 150.28 | 0.0447 |
| Vertical with machined surface (as-built) | 15 | 0.230097 | 6 | 162.98 | 0.0489 |
| Vertical with machined surface (as-built) | 16 | 0.248326 | 6 | 175.9 | 0.0534 |
| Vertical with machined surface (as-built) | 17 | 0.266852 | 6 | 189.02 | 0.0581 |
| Vertical with machined surface (as-built) | 18 | 0.285661 | 6 | 202.36 | 0.063 |
| Vertical with machined surface (as-built) | 19 | 0.304733 | 6 | 215.88 | 0.068 |
| Vertical with machined surface (as-built) | 20 | 0.324046 | 6 | 229.56 | 0.0731 |
| Vertical with machined surface (as-built) | 21 | 0.343572 | 6 | 243.4 | 0.0783 |
| Vertical with machined surface (as-built) | 22 | 0.36328 | 6 | 257.38 | 0.0835 |
| Vertical with machined surface (as-built) | 23 | 0.383135 | 6 | 271.46 | 0.0888 |
| Vertical with machined surface (as-built) | 24 | 0.403105 | 6 | 285.62 | 0.0941 |
| Vertical with machined surface (as-built) | 25 | 0.423154 | 6 | 299.82 | 0.0995 |
| Vertical with machined surface (as-built) | 26 | 0.443249 | 6 | 314.08 | 0.105 |
| Vertical with machined surface (as-built) | 27 | 0.463356 | 6 | 328.34 | 0.1106 |
| Vertical with machined surface (as-built) | 28 | 0.483445 | 6 | 342.6 | 0.1163 |
| Vertical with machined surface (as-built) | 29 | 0.503486 | 6 | 356.8 | 0.1221 |
| Vertical with machined surface (as-built) | 30 | 0.52345 | 6 | 370.98 | 0.128 |
| Vertical with machined surface (as-built) | 31 | 0.543313 | 6 | 385.08 | 0.134 |
| Vertical with machined surface (as-built) | 32 | 0.56305 | 6 | 399.08 | 0.1401 |
| Vertical with machined surface (as-built) | 33 | 0.582639 | 6 | 412.98 | 0.1462 |
| Vertical with machined surface (as-built) | 34 | 0.602059 | 6 | 426.78 | 0.1525 |
| Vertical with machined surface (as-built) | 35 | 0.621291 | 6 | 440.44 | 0.1589 |
| Vertical with machined surface (as-built) | 36 | 0.64032 | 6 | 453.96 | 0.1654 |
| Vertical with machined surface (as-built) | 37 | 0.65913 | 6 | 467.32 | 0.172 |
| Vertical with machined surface (as-built) | 38 | 0.677709 | 6 | 480.52 | 0.1788 |
| Vertical with machined surface (as-built) | 39 | 0.696047 | 6 | 493.54 | 0.1858 |
| Vertical with machined surface (as-built) | 40 | 0.714136 | 6 | 506.4 | 0.1928 |
| Vertical with machined surface (as-built) | 41 | 0.73197 | 6 | 519.08 | 0.2 |
| Vertical with machined surface (as-built) | 42 | 0.749542 | 6 | 531.58 | 0.2073 |
| Vertical with machined surface (as-built) | 43 | 0.766847 | 6 | 543.9 | 0.2147 |
| Vertical with machined surface (as-built) | 44 | 0.78388 | 6 | 556.02 | 0.2221 |
| Vertical with machined surface (as-built) | 45 | 0.800638 | 6 | 567.94 | 0.2296 |
| Vertical with machined surface (as-built) | 46 | 0.817116 | 6 | 579.68 | 0.2372 |
| Vertical with machined surface (as-built) | 47 | 0.833314 | 6 | 591.2 | 0.2447 |
| Vertical with machined surface (as-built) | 48 | 0.849229 | 6 | 602.54 | 0.2523 |
| Vertical with machined surface (as-built) | 49 | 0.864862 | 6 | 613.68 | 0.26 |
| Vertical with machined surface (as-built) | 50 | 0.880213 | 6 | 624.62 | 0.2676 |
| Vertical with machined surface (as-built) | 51 | 0.895281 | 6 | 635.36 | 0.2753 |
| Vertical with machined surface (as-built) | 52 | 0.910067 | 6 | 645.9 | 0.2831 |
| Vertical with machined surface (as-built) | 53 | 0.924569 | 6 | 656.24 | 0.2909 |
| Vertical with machined surface (as-built) | 54 | 0.938789 | 6 | 666.38 | 0.2989 |
| Vertical with machined surface (as-built) | 55 | 0.952727 | 6 | 676.34 | 0.3071 |
| Vertical with machined surface (as-built) | 56 | 0.966385 | 6 | 686.08 | 0.3154 |
| Vertical with machined surface (as-built) | 57 | 0.979763 | 6 | 695.64 | 0.3239 |
| Vertical with machined surface (as-built) | 58 | 0.992862 | 6 | 705 | 0.3327 |
| Vertical with machined surface (as-built) | 59 | 1.005679 | 6 | 714.18 | 0.3418 |
| Vertical with machined surface (as-built) | 60 | 1.018215 | 6 | 723.14 | 0.3513 |
| Vertical with machined surface (as-built) | 61 | 1.030469 | 6 | 731.92 | 0.3611 |
| Vertical with machined surface (as-built) | 62 | 1.042438 | 6 | 740.5 | 0.3713 |
| Vertical with machined surface (as-built) | 63 | 1.054121 | 6 | 748.88 | 0.382 |
| Vertical with machined surface (as-built) | 64 | 1.065517 | 6 | 757.06 | 0.3931 |
| Vertical with machined surface (as-built) | 65 | 1.076626 | 6 | 765.04 | 0.4046 |
| Vertical with machined surface (as-built) | 66 | 1.087455 | 6 | 772.82 | 0.4164 |
| Vertical with machined surface (as-built) | 67 | 1.098012 | 6 | 780.42 | 0.4286 |
| Vertical with machined surface (as-built) | 68 | 1.108305 | 6 | 787.84 | 0.441 |
| Vertical with machined surface (as-built) | 69 | 1.118339 | 6 | 795.06 | 0.4536 |
| Vertical with machined surface (as-built) | 70 | 1.128124 | 6 | 802.12 | 0.4663 |
| Vertical with machined surface (as-built) | 71 | 1.137666 | 6 | 809 | 0.4789 |
| Vertical with machined surface (as-built) | 72 | 1.146974 | 6 | 815.72 | 0.4915 |
| Vertical with machined surface (as-built) | 73 | 1.156056 | 6 | 822.26 | 0.5038 |
| Vertical with machined surface (as-built) | 74 | 1.16492 | 6 | 828.66 | 0.5158 |
| Vertical with machined surface (as-built) | 75 | 1.173573 | 6 | 834.92 | 0.5275 |
| Vertical with machined surface (as-built) | 76 | 1.182024 | 6 | 841.02 | 0.5388 |
| Vertical with machined surface (as-built) | 77 | 1.190281 | 6 | 846.98 | 0.5495 |
| Vertical with machined surface (as-built) | 78 | 1.198348 | 6 | 852.8 | 0.5599 |
| Vertical with machined surface (as-built) | 79 | 1.206232 | 6 | 858.5 | 0.5697 |
| Vertical with machined surface (as-built) | 80 | 1.213937 | 6 | 864.06 | 0.579 |
| Vertical with machined surface (as-built) | 81 | 1.221466 | 6 | 869.48 | 0.588 |
| Vertical with machined surface (as-built) | 82 | 1.228824 | 6 | 874.78 | 0.5964 |
| Vertical with machined surface (as-built) | 83 | 1.236015 | 6 | 879.98 | 0.6045 |
| Vertical with machined surface (as-built) | 84 | 1.243042 | 6 | 885.04 | 0.6122 |
| Vertical with machined surface (as-built) | 85 | 1.249909 | 6 | 890 | 0.6195 |
| Vertical with machined surface (as-built) | 86 | 1.256618 | 6 | 894.84 | 0.6266 |
| Vertical with machined surface (as-built) | 87 | 1.263175 | 6 | 899.56 | 0.6334 |
| Vertical with machined surface (as-built) | 88 | 1.269582 | 6 | 904.18 | 0.64 |
| Vertical with machined surface (as-built) | 89 | 1.275841 | 6 | 908.7 | 0.6465 |
| Vertical with machined surface (as-built) | 90 | 1.281956 | 6 | 913.1 | 0.6528 |
| Vertical with machined surface (as-built) | 91 | 1.287929 | 6 | 917.42 | 0.6592 |
| Vertical with machined surface (as-built) | 92 | 1.293762 | 6 | 921.62 | 0.6655 |
| Vertical with machined surface (as-built) | 93 | 1.299458 | 6 | 925.74 | 0.672 |
| Vertical with machined surface (as-built) | 94 | **1.30502** | 6 | **929.76** | **0.6786** |
| Vertical with machined surface (as-built) | 95 | 1.310449 | 6 | 933.7 | 0.6854 |
| Vertical with machined surface (as-built) | 96 | 1.315746 | 6 | 937.54 | 0.6925 |
| Vertical with machined surface (as-built) | 97 | 1.320914 | 6 | 941.3 | 0.6999 |
| Vertical with machined surface (as-built) | 98 | 1.325959 | 6 | 944.96 | 0.7078 |
| Vertical with machined surface (as-built) | 99 | 1.330884 | 6 | 948.54 | 0.716 |
| Vertical with machined surface (as-built) | 100 | 1.335695 | 6 | 952.06 | 0.7246 |
| Vertical with machined surface (as-built) | 101 | 1.340396 | 6 | 955.5 | 0.7337 |
| Vertical with machined surface (as-built) | 102 | 1.344991 | 6 | 958.86 | 0.7432 |
| Vertical with machined surface (as-built) | 103 | 1.349485 | 6 | 962.16 | 0.7531 |
| Vertical with machined surface (as-built) | 104 | 1.353878 | 6 | 965.38 | 0.7634 |
| Vertical with machined surface (as-built) | 105 | 1.358175 | 6 | 968.54 | 0.774 |
| Vertical with machined surface (as-built) | 106 | 1.362376 | 6 | 971.64 | 0.7849 |
| Vertical with machined surface (as-built) | 107 | 1.366483 | 6 | 974.68 | 0.7959 |
| Vertical with machined surface (as-built) | 108 | 1.370499 | 6 | 977.66 | 0.8072 |
| Vertical with machined surface (as-built) | 109 | 1.374424 | 6 | 980.56 | 0.8186 |
| Vertical with machined surface (as-built) | 110 | 1.378263 | 6 | 983.4 | 0.83 |
| Vertical with machined surface (as-built) | 111 | 1.382019 | 6 | 986.2 | 0.8414 |
| Vertical with machined surface (as-built) | 112 | 1.385694 | 6 | 988.92 | 0.8528 |
| Vertical with machined surface (as-built) | 113 | 1.389294 | 6 | 991.6 | 0.8642 |
| Vertical with machined surface (as-built) | 114 | 1.392819 | 6 | 994.22 | 0.8754 |
| Vertical with machined surface (as-built) | 115 | 1.396272 | 6 | 996.8 | 0.8865 |
| Vertical with machined surface (as-built) | 116 | 1.399653 | 6 | 999.32 | 0.8974 |
| Vertical with machined surface (as-built) | 117 | 1.402963 | 6 | 1001.78 | 0.9082 |
| Vertical with machined surface (as-built) | 118 | 1.406203 | 6 | 1004.2 | 0.9189 |
| Vertical with machined surface (as-built) | 119 | 1.40937 | 6 | 1006.56 | 0.9293 |
| Vertical with machined surface (as-built) | 120 | 1.412465 | 6 | 1008.86 | 0.9396 |
| Vertical with machined surface (as-built) | 121 | 1.415487 | 6 | 1011.12 | 0.9498 |
| Vertical with machined surface (as-built) | 122 | 1.418439 | 6 | 1013.32 | 0.9597 |
| Vertical with machined surface (as-built) | 123 | 1.421319 | 6 | 1015.48 | 0.9694 |
| Vertical with machined surface (as-built) | 124 | 1.42413 | 6 | 1017.58 | 0.979 |
| Vertical with machined surface (as-built) | 125 | 1.426873 | 6 | 1019.64 | 0.9883 |
| Vertical with machined surface (as-built) | 126 | 1.429552 | 6 | 1021.64 | 0.9974 |
| Vertical with machined surface (as-built) | 127 | 1.432167 | 6 | 1023.6 | 1.0063 |
| Vertical with machined surface (as-built) | 128 | 1.434721 | 6 | 1025.5 | 1.0149 |
| Vertical with machined surface (as-built) | 129 | 1.437216 | 6 | 1027.38 | 1.0232 |
| Vertical with machined surface (as-built) | 130 | 1.439654 | 6 | 1029.2 | 1.0313 |
| Vertical with machined surface (as-built) | 131 | 1.442037 | 6 | 1030.98 | 1.0391 |
| Vertical with machined surface (as-built) | 132 | 1.444368 | 6 | 1032.72 | 1.0466 |
| Vertical with machined surface (as-built) | 133 | 1.44665 | 6 | 1034.44 | 1.0539 |
| Vertical with machined surface (as-built) | 134 | 1.448885 | 6 | 1036.1 | 1.0609 |
| Vertical with machined surface (as-built) | 135 | 1.451076 | 6 | 1037.74 | 1.0678 |
| Vertical with machined surface (as-built) | 136 | 1.453225 | 6 | 1039.34 | 1.0745 |
| Vertical with machined surface (as-built) | 137 | 1.455336 | 6 | 1040.92 | 1.081 |
| Vertical with machined surface (as-built) | 138 | 1.457409 | 6 | 1042.46 | 1.0874 |
| Vertical with machined surface (as-built) | 139 | 1.459446 | 6 | 1043.98 | 1.0936 |
| Vertical with machined surface (as-built) | 140 | 1.461447 | 6 | 1045.48 | 1.0998 |
| Vertical with machined surface (as-built) | 141 | 1.463412 | 6 | 1046.96 | 1.106 |
| Vertical with machined surface (as-built) | 142 | 1.465342 | 6 | 1048.4 | 1.1121 |
| Vertical with machined surface (as-built) | 143 | 1.467237 | 6 | 1049.82 | 1.1184 |
| Vertical with machined surface (as-built) | 144 | 1.469098 | 6 | 1051.22 | 1.1247 |
| Vertical with machined surface (as-built) | 145 | 1.470927 | 6 | 1052.6 | 1.1311 |
| Vertical with machined surface (as-built) | 146 | 1.472727 | 6 | 1053.94 | 1.1377 |
| Vertical with machined surface (as-built) | 147 | 1.474499 | 6 | 1055.28 | 1.1446 |
| Vertical with machined surface (as-built) | 148 | 1.476246 | 6 | 1056.62 | 1.1517 |
| Vertical with machined surface (as-built) | 149 | 1.477969 | 6 | 1057.92 | 1.1592 |
| Vertical with machined surface (as-built) | 150 | 1.47967 | 6 | 1059.22 | 1.1671 |
| Vertical with machined surface (as-built) | 151 | 1.48135 | 6 | 1060.5 | 1.1753 |
| Vertical with machined surface (as-built) | 152 | 1.483013 | 6 | 1061.78 | 1.184 |
| Vertical with machined surface (as-built) | 153 | 1.48466 | 6 | 1063.06 | 1.1932 |
| Vertical with machined surface (as-built) | 154 | 1.486295 | 6 | 1064.34 | 1.2029 |
| Vertical with machined surface (as-built) | 155 | 1.487923 | 6 | 1065.6 | 1.213 |
| Vertical with machined surface (as-built) | 156 | 1.489547 | 6 | 1066.88 | 1.2237 |
| Vertical with machined surface (as-built) | 157 | 1.491173 | 6 | 1068.16 | 1.2348 |
| Vertical with machined surface (as-built) | 158 | 1.492806 | 6 | 1069.44 | 1.2464 |
| Vertical with machined surface (as-built) | 159 | 1.49445 | 6 | 1070.76 | 1.2586 |
| Vertical with machined surface (as-built) | 160 | 1.496112 | 6 | 1072.08 | 1.2713 |
| Vertical with machined surface (as-built) | 161 | 1.497793 | 6 | 1073.42 | 1.2845 |
| Vertical with machined surface (as-built) | 162 | 1.499495 | 6 | 1074.78 | 1.2983 |
| Vertical with machined surface (as-built) | 163 | 1.501218 | 6 | 1076.16 | 1.3127 |
| Vertical with machined surface (as-built) | 164 | 1.502962 | 6 | 1077.58 | 1.3279 |
| Vertical with machined surface (as-built) | 165 | 1.504728 | 6 | 1079 | 1.3438 |
| Vertical with machined surface (as-built) | 166 | 1.506513 | 6 | 1080.46 | 1.3606 |
| Vertical with machined surface (as-built) | 167 | 1.508315 | 6 | 1081.94 | 1.3783 |
| Vertical with machined surface (as-built) | 168 | 1.51013 | 6 | 1083.44 | 1.3971 |
| Vertical with machined surface (as-built) | 169 | 1.511954 | 6 | 1084.96 | 1.417 |
| Vertical with machined surface (as-built) | 170 | 1.513778 | 6 | 1086.5 | 1.4382 |
| Vertical with machined surface (as-built) | 171 | 1.515593 | 6 | 1088.04 | 1.4606 |
| Vertical with machined surface (as-built) | 172 | 1.51739 | 6 | 1089.58 | 1.4845 |
| Vertical with machined surface (as-built) | 173 | 1.519161 | 6 | 1091.12 | 1.5099 |
| Vertical with machined surface (as-built) | 174 | 1.520897 | 6 | 1092.66 | 1.5369 |
| Vertical with machined surface (as-built) | 175 | 1.522593 | 6 | 1094.18 | 1.5655 |
| Vertical with machined surface (as-built) | 176 | 1.524242 | 6 | 1095.7 | 1.5957 |
| Vertical with machined surface (as-built) | 177 | 1.525838 | 6 | 1097.18 | 1.6277 |
| Vertical with machined surface (as-built) | 178 | 1.527377 | 6 | 1098.64 | 1.6612 |
| Vertical with machined surface (as-built) | 179 | 1.528851 | 6 | 1100.08 | 1.6965 |
| Vertical with machined surface (as-built) | 180 | 1.530257 | 6 | 1101.5 | 1.7334 |
| Vertical with machined surface (as-built) | 181 | 1.531588 | 6 | 1102.86 | 1.7719 |
| Vertical with machined surface (as-built) | 182 | 1.53284 | 6 | 1104.2 | 1.8121 |
| Vertical with machined surface (as-built) | 183 | 1.534012 | 6 | 1105.5 | 1.854 |
| Vertical with machined surface (as-built) | 184 | 1.535101 | 6 | 1106.74 | 1.8974 |
| Vertical with machined surface (as-built) | 185 | 1.536107 | 6 | 1107.96 | 1.9426 |
| Vertical with machined surface (as-built) | 186 | 1.53703 | 6 | 1109.12 | 1.9894 |
| Vertical with machined surface (as-built) | 187 | 1.537866 | 6 | 1110.26 | 2.0379 |
| Vertical with machined surface (as-built) | 188 | 1.538615 | 6 | 1111.34 | 2.0882 |
| Vertical with machined surface (as-built) | 189 | 1.539275 | 6 | 1112.38 | 2.1403 |
| Vertical with machined surface (as-built) | 190 | 1.539844 | 6 | 1113.38 | 2.1942 |
| Vertical with machined surface (as-built) | 191 | 1.540323 | 6 | 1114.32 | 2.25 |
| Vertical with machined surface (as-built) | 192 | 1.540708 | 6 | 1115.22 | 2.3078 |
| Vertical with machined surface (as-built) | 193 | 1.541001 | 6 | 1116.08 | 2.3676 |
| Vertical with machined surface (as-built) | 194 | 1.541199 | 6 | 1116.9 | 2.4296 |
| Vertical with machined surface (as-built) | 195 | 1.541302 | 6 | 1117.66 | 2.4938 |
| Vertical with machined surface (as-built) | 196 | **1.541309** | 6 | **1118.38** | **2.5603** |
| Vertical with machined surface (as-built) | 197 | 1.541218 | 6 | 1119.06 | 2.6292 |
| Vertical with machined surface (as-built) | 198 | 1.541028 | 6 | 1119.7 | 2.7005 |
| Vertical with machined surface (as-built) | 199 | 1.540737 | 6 | 1120.3 | 2.7743 |
| Vertical with machined surface (as-built) | 200 | 1.540345 | 6 | 1120.84 | 2.8506 |
| Vertical with machined surface (as-built) | 201 | 1.539852 | 6 | 1121.34 | 2.9295 |
| Vertical with machined surface (as-built) | 202 | 1.539258 | 6 | 1121.78 | 3.011 |
| Vertical with machined surface (as-built) | 203 | 1.538565 | 6 | 1122.18 | 3.095 |
| Vertical with machined surface (as-built) | 204 | 1.537774 | 6 | 1122.56 | 3.1817 |
| Vertical with machined surface (as-built) | 205 | 1.536887 | 6 | 1122.86 | 3.2708 |
| Vertical with machined surface (as-built) | 206 | 1.535905 | 6 | 1123.14 | 3.3625 |
| Vertical with machined surface (as-built) | 207 | 1.534831 | 6 | 1123.38 | 3.4566 |
| Vertical with machined surface (as-built) | 208 | 1.533668 | 6 | 1123.58 | 3.5532 |
| Vertical with machined surface (as-built) | 209 | 1.53242 | 6 | 1123.74 | 3.6523 |
| Vertical with machined surface (as-built) | 210 | 1.531089 | 6 | 1123.86 | 3.7536 |
| Vertical with machined surface (as-built) | 211 | 1.529681 | 6 | 1123.96 | 3.8572 |
| Vertical with machined surface (as-built) | 212 | 1.528197 | 6 | 1124.02 | 3.963 |
| Vertical with machined surface (as-built) | 213 | 1.526642 | 6 | 1124.04 | 4.0709 |
| Vertical with machined surface (as-built) | 214 | 1.525019 | 6 | 1124.04 | 4.1807 |
| Vertical with machined surface (as-built) | 215 | 1.523331 | 6 | 1124 | 4.2923 |
| Vertical with machined surface (as-built) | 216 | 1.521582 | 6 | 1123.94 | 4.4057 |
| Vertical with machined surface (as-built) | 217 | 1.519775 | 6 | 1123.86 | 4.5206 |
| Vertical with machined surface (as-built) | 218 | 1.517915 | 6 | 1123.74 | 4.637 |
| Vertical with machined surface (as-built) | 219 | 1.516005 | 6 | 1123.62 | 4.7548 |
| Vertical with machined surface (as-built) | 220 | 1.51405 | 6 | 1123.46 | 4.8739 |
| Vertical with machined surface (as-built) | 221 | 1.512051 | 6 | 1123.28 | 4.9942 |
| Vertical with machined surface (as-built) | 222 | 1.510013 | 6 | 1123.08 | 5.1157 |
| Vertical with machined surface (as-built) | 223 | 1.507936 | 6 | 1122.86 | 5.2383 |
| Vertical with machined surface (as-built) | 224 | 1.505824 | 6 | 1122.64 | 5.3621 |
| Vertical with machined surface (as-built) | 225 | 1.503678 | 6 | 1122.38 | 5.4868 |
| Vertical with machined surface (as-built) | 226 | 1.501501 | 6 | 1122.12 | 5.6127 |
| Vertical with machined surface (as-built) | 227 | 1.499295 | 6 | 1121.86 | 5.7395 |
| Vertical with machined surface (as-built) | 228 | 1.497065 | 6 | 1121.58 | 5.8674 |
| Vertical with machined surface (as-built) | 229 | 1.494814 | 6 | 1121.28 | 5.9961 |
| Vertical with machined surface (as-built) | 230 | 1.492544 | 6 | 1120.98 | 6.1258 |
| Vertical with machined surface (as-built) | 231 | 1.490258 | 6 | 1120.68 | 6.2564 |
| Vertical with machined surface (as-built) | 232 | 1.487957 | 6 | 1120.38 | 6.3879 |
| Vertical with machined surface (as-built) | 233 | 1.485643 | 6 | 1120.06 | 6.5202 |
| Vertical with machined surface (as-built) | 234 | 1.483316 | 6 | 1119.74 | 6.6534 |
| Vertical with machined surface (as-built) | 235 | 1.480977 | 6 | 1119.44 | 6.7875 |
| Vertical with machined surface (as-built) | 236 | 1.478624 | 6 | 1119.12 | 6.9224 |
| Vertical with machined surface (as-built) | 237 | 1.476259 | 6 | 1118.8 | 7.0583 |
| Vertical with machined surface (as-built) | 238 | 1.473878 | 6 | 1118.46 | 7.1951 |
| Vertical with machined surface (as-built) | 239 | 1.471481 | 6 | 1118.14 | 7.3329 |
| Vertical with machined surface (as-built) | 240 | 1.469067 | 6 | 1117.8 | 7.4717 |
| Vertical with machined surface (as-built) | 241 | 1.466642 | 6 | 1117.46 | 7.6114 |
| Vertical with machined surface (as-built) | 242 | 1.464212 | 6 | 1117.12 | 7.7522 |
| Vertical with machined surface (as-built) | 243 | 1.461782 | 6 | 1116.8 | 7.8939 |
| Vertical with machined surface (as-built) | 244 | 1.459354 | 6 | 1116.48 | 8.0367 |
| Vertical with machined surface (as-built) | 245 | 1.456933 | 6 | 1116.18 | 8.1804 |
| Vertical with machined surface (as-built) | 246 | 1.454518 | 6 | 1115.9 | 8.325 |
| Vertical with machined surface (as-built) | 247 | 1.452111 | 6 | 1115.62 | 8.4705 |
| Vertical with machined surface (as-built) | 248 | 1.449713 | 6 | 1115.36 | 8.6168 |
| Vertical with machined surface (as-built) | 249 | 1.447325 | 6 | 1115.1 | 8.7638 |
| Vertical with machined surface (as-built) | 250 | 1.444947 | 6 | 1114.86 | 8.9116 |
| Vertical with machined surface (as-built) | 251 | 1.442581 | 6 | 1114.64 | 9.0601 |
| Vertical with machined surface (as-built) | 252 | 1.44023 | 6 | 1114.42 | 9.2091 |
| Vertical with machined surface (as-built) | 253 | 1.437893 | 6 | 1114.22 | 9.3587 |
| Vertical with machined surface (as-built) | 254 | 1.435573 | 6 | 1114.04 | 9.5087 |
| Vertical with machined surface (as-built) | 255 | 1.433268 | 6 | 1113.86 | 9.6592 |
| Vertical with machined surface (as-built) | 256 | 1.430979 | 6 | 1113.72 | 9.8101 |
| Vertical with machined surface (as-built) | 257 | 1.428704 | 6 | 1113.56 | 9.9613 |
| Vertical with machined surface (as-built) | 258 | 1.426444 | 6 | 1113.44 | 10.1129 |
| Vertical with machined surface (as-built) | 259 | 1.424197 | 6 | 1113.32 | 10.2649 |
| Vertical with machined surface (as-built) | 260 | 1.421964 | 6 | 1113.2 | 10.4171 |
| Vertical with machined surface (as-built) | 261 | 1.419744 | 6 | 1113.1 | 10.5697 |
| Vertical with machined surface (as-built) | 262 | 1.417538 | 6 | 1113.02 | 10.7227 |
| Vertical with machined surface (as-built) | 263 | 1.415346 | 6 | 1112.94 | 10.876 |
| Vertical with machined surface (as-built) | 264 | 1.41317 | 6 | 1112.88 | 11.0297 |
| Vertical with machined surface (as-built) | 265 | 1.411011 | 6 | 1112.84 | 11.1838 |
| Vertical with machined surface (as-built) | 266 | 1.40887 | 6 | 1112.82 | 11.3382 |
| Vertical with machined surface (as-built) | 267 | 1.406748 | 6 | 1112.8 | 11.4931 |
| Vertical with machined surface (as-built) | 268 | 1.404645 | 6 | 1112.8 | 11.6484 |
| Vertical with machined surface (as-built) | 269 | 1.402563 | 6 | 1112.84 | 11.8042 |
| Vertical with machined surface (as-built) | 270 | 1.400499 | 6 | 1112.88 | 11.9605 |
| Vertical with machined surface (as-built) | 271 | 1.398451 | 6 | 1112.92 | 12.1173 |
| Vertical with machined surface (as-built) | 272 | 1.396416 | 6 | 1113 | 12.2746 |
| Vertical with machined surface (as-built) | 273 | 1.394394 | 6 | 1113.08 | 12.4323 |
| Vertical with machined surface (as-built) | 274 | 1.392385 | 6 | 1113.18 | 12.5905 |
| Vertical with machined surface (as-built) | 275 | 1.390388 | 6 | 1113.28 | 12.7491 |
| Vertical with machined surface (as-built) | 276 | 1.388404 | 6 | 1113.4 | 12.9082 |
| Vertical with machined surface (as-built) | 277 | 1.386432 | 6 | 1113.54 | 13.0676 |
| Vertical with machined surface (as-built) | 278 | 1.384473 | 6 | 1113.68 | 13.2273 |
| Vertical with machined surface (as-built) | 279 | 1.382526 | 6 | 1113.82 | 13.3874 |
| Vertical with machined surface (as-built) | 280 | 1.38059 | 6 | 1113.98 | 13.5476 |
| Vertical with machined surface (as-built) | 281 | 1.378665 | 6 | 1114.16 | 13.708 |
| Vertical with machined surface (as-built) | 282 | 1.376749 | 6 | 1114.34 | 13.8687 |
| Vertical with machined surface (as-built) | 283 | 1.374843 | 6 | 1114.52 | 14.0295 |
| Vertical with machined surface (as-built) | 284 | 1.372946 | 6 | 1114.72 | 14.1906 |
| Vertical with machined surface (as-built) | 285 | 1.37106 | 6 | 1114.92 | 14.3519 |
| Vertical with machined surface (as-built) | 286 | 1.369186 | 6 | 1115.14 | 14.5135 |
| Vertical with machined surface (as-built) | 287 | 1.367324 | 6 | 1115.36 | 14.6755 |
| Vertical with machined surface (as-built) | 288 | 1.365475 | 6 | 1115.6 | 14.8379 |
| Vertical with machined surface (as-built) | 289 | 1.36364 | 6 | 1115.86 | 15.0007 |
| Vertical with machined surface (as-built) | 290 | 1.36182 | 6 | 1116.12 | 15.164 |
| Vertical with machined surface (as-built) | 291 | 1.360014 | 6 | 1116.4 | 15.3278 |
| Vertical with machined surface (as-built) | 292 | 1.358225 | 6 | 1116.72 | 15.4921 |
| Vertical with machined surface (as-built) | 293 | 1.356452 | 6 | 1117.02 | 15.6569 |
| Vertical with machined surface (as-built) | 294 | 1.354698 | 6 | 1117.36 | 15.8222 |
| Vertical with machined surface (as-built) | 295 | 1.352963 | 6 | 1117.72 | 15.988 |
| Vertical with machined surface (as-built) | 296 | 1.351247 | 6 | 1118.1 | 16.1543 |
| Vertical with machined surface (as-built) | 297 | 1.349551 | 6 | 1118.5 | 16.321 |
| Vertical with machined surface (as-built) | 298 | 1.347871 | 6 | 1118.9 | 16.4881 |
| Vertical with machined surface (as-built) | 299 | 1.346206 | 6 | 1119.34 | 16.6555 |
| Vertical with machined surface (as-built) | 300 | 1.344552 | 6 | 1119.76 | 16.8232 |
| Vertical with machined surface (as-built) | 301 | 1.342906 | 6 | 1120.22 | 16.9912 |
| Vertical with machined surface (as-built) | 302 | 1.341264 | 6 | 1120.66 | 17.1594 |
| Vertical with machined surface (as-built) | 303 | 1.339623 | 6 | 1121.1 | 17.3277 |
| Vertical with machined surface (as-built) | 304 | 1.337982 | 6 | 1121.56 | 17.4961 |
| Vertical with machined surface (as-built) | 305 | 1.336339 | 6 | 1122 | 17.6645 |
| Vertical with machined surface (as-built) | 306 | 1.334692 | 6 | 1122.44 | 17.8329 |
| Vertical with machined surface (as-built) | 307 | 1.333039 | 6 | 1122.86 | 18.0012 |
| Vertical with machined surface (as-built) | 308 | 1.33138 | 6 | 1123.28 | 18.1694 |
| Vertical with machined surface (as-built) | 309 | 1.329712 | 6 | 1123.7 | 18.3373 |
| Vertical with machined surface (as-built) | 310 | 1.328036 | 6 | 1124.1 | 18.5051 |
| Vertical with machined surface (as-built) | 311 | 1.326354 | 6 | 1124.48 | 18.6727 |
| Vertical with machined surface (as-built) | 312 | 1.324665 | 6 | 1124.86 | 18.8402 |
| Vertical with machined surface (as-built) | 313 | 1.322971 | 6 | 1125.24 | 19.0077 |
| Vertical with machined surface (as-built) | 314 | 1.321274 | 6 | 1125.62 | 19.1752 |
| Vertical with machined surface (as-built) | 315 | 1.319573 | 6 | 1125.98 | 19.3428 |
| Vertical with machined surface (as-built) | 316 | 1.317869 | 6 | 1126.34 | 19.5105 |
| Vertical with machined surface (as-built) | 317 | 1.316162 | 6 | 1126.72 | 19.6785 |
| Vertical with machined surface (as-built) | 318 | 1.314452 | 6 | 1127.08 | 19.8468 |
| Vertical with machined surface (as-built) | 319 | 1.312739 | 6 | 1127.44 | 20.0154 |
| Vertical with machined surface (as-built) | 320 | 1.311025 | 6 | 1127.8 | 20.1844 |
| Vertical with machined surface (as-built) | 321 | 1.30931 | 6 | 1128.16 | 20.3538 |
| Vertical with machined surface (as-built) | 322 | 1.307596 | 6 | 1128.54 | 20.5236 |
| Vertical with machined surface (as-built) | 323 | 1.305884 | 6 | 1128.9 | 20.6937 |
| Vertical with machined surface (as-built) | 324 | 1.304173 | 6 | 1129.28 | 20.8642 |
| Vertical with machined surface (as-built) | 325 | 1.302463 | 6 | 1129.66 | 21.0351 |
| Vertical with machined surface (as-built) | 326 | 1.300756 | 6 | 1130.04 | 21.2063 |
| Vertical with machined surface (as-built) | 327 | 1.299052 | 6 | 1130.42 | 21.3779 |
| Vertical with machined surface (as-built) | 328 | 1.29735 | 6 | 1130.82 | 21.5498 |
| Vertical with machined surface (as-built) | 329 | 1.295651 | 6 | 1131.22 | 21.722 |
| Vertical with machined surface (as-built) | 330 | 1.293955 | 6 | 1131.62 | 21.8945 |
| Vertical with machined surface (as-built) | 331 | 1.292265 | 6 | 1132.02 | 22.0674 |
| Vertical with machined surface (as-built) | 332 | 1.290583 | 6 | 1132.44 | 22.2408 |
| Vertical with machined surface (as-built) | 333 | 1.288911 | 6 | 1132.86 | 22.4146 |
| Vertical with machined surface (as-built) | 334 | 1.28725 | 6 | 1133.32 | 22.5889 |
| Vertical with machined surface (as-built) | 335 | 1.285603 | 6 | 1133.78 | 22.7638 |
| Vertical with machined surface (as-built) | 336 | 1.28397 | 6 | 1134.24 | 22.9391 |
| Vertical with machined surface (as-built) | 337 | 1.282351 | 6 | 1134.74 | 23.1149 |
| Vertical with machined surface (as-built) | 338 | 1.280748 | 6 | 1135.26 | 23.2911 |
| Vertical with machined surface (as-built) | 339 | 1.279159 | 6 | 1135.78 | 23.4677 |
| Vertical with machined surface (as-built) | 340 | 1.277584 | 6 | 1136.32 | 23.6446 |
| Vertical with machined surface (as-built) | 341 | 1.276022 | 6 | 1136.86 | 23.8217 |
| Vertical with machined surface (as-built) | 342 | 1.274472 | 6 | 1137.42 | 23.9989 |
| Vertical with machined surface (as-built) | 343 | 1.272932 | 6 | 1138 | 24.1761 |
| Vertical with machined surface (as-built) | 344 | 1.271402 | 6 | 1138.58 | 24.3534 |
| Vertical with machined surface (as-built) | 345 | 1.269882 | 6 | 1139.16 | 24.5308 |
| Vertical with machined surface (as-built) | 346 | 1.268371 | 6 | 1139.76 | 24.7082 |
| Vertical with machined surface (as-built) | 347 | 1.266869 | 6 | 1140.34 | 24.8856 |
| Vertical with machined surface (as-built) | 348 | 1.265374 | 6 | 1140.96 | 25.0633 |
| Vertical with machined surface (as-built) | 349 | 1.263886 | 6 | 1141.56 | 25.2412 |
| Vertical with machined surface (as-built) | 350 | 1.262403 | 6 | 1142.18 | 25.4193 |
| Vertical with machined surface (as-built) | 351 | 1.260925 | 6 | 1142.82 | 25.5979 |
| Vertical with machined surface (as-built) | 352 | 1.259449 | 6 | 1143.44 | 25.7769 |
| Vertical with machined surface (as-built) | 353 | 1.257974 | 6 | 1144.08 | 25.9565 |
| Vertical with machined surface (as-built) | 354 | 1.256497 | 6 | 1144.72 | 26.1366 |
| Vertical with machined surface (as-built) | 355 | 1.255016 | 6 | 1145.36 | 26.3173 |
| Vertical with machined surface (as-built) | 356 | 1.253528 | 6 | 1146 | 26.4987 |
| Vertical with machined surface (as-built) | 357 | 1.252032 | 6 | 1146.64 | 26.6808 |
| Vertical with machined surface (as-built) | 358 | 1.250527 | 6 | 1147.28 | 26.8636 |
| Vertical with machined surface (as-built) | 359 | 1.249015 | 6 | 1147.92 | 27.0472 |
| Vertical with machined surface (as-built) | 360 | 1.247499 | 6 | 1148.58 | 27.2316 |
| Vertical with machined surface (as-built) | 361 | 1.245979 | 6 | 1149.22 | 27.4168 |
| Vertical with machined surface (as-built) | 362 | 1.244454 | 6 | 1149.86 | 27.6028 |
| Vertical with machined surface (as-built) | 363 | 1.242925 | 6 | 1150.52 | 27.7895 |
| Vertical with machined surface (as-built) | 364 | 1.24139 | 6 | 1151.18 | 27.977 |
| Vertical with machined surface (as-built) | 365 | 1.239847 | 6 | 1151.84 | 28.1653 |
| Vertical with machined surface (as-built) | 366 | 1.238296 | 6 | 1152.48 | 28.3545 |
| Vertical with machined surface (as-built) | 367 | 1.236736 | 6 | 1153.14 | 28.5446 |
| Vertical with machined surface (as-built) | 368 | 1.235166 | 6 | 1153.8 | 28.7356 |
| Vertical with machined surface (as-built) | 369 | 1.233587 | 6 | 1154.46 | 28.9274 |
| Vertical with machined surface (as-built) | 370 | 1.232 | 6 | 1155.1 | 29.1202 |
| Vertical with machined surface (as-built) | 371 | 1.230405 | 6 | 1155.76 | 29.3138 |
| Vertical with machined surface (as-built) | 372 | 1.228803 | 6 | 1156.42 | 29.5085 |
| Vertical with machined surface (as-built) | 373 | 1.227194 | 6 | 1157.08 | 29.7041 |
| Vertical with machined surface (as-built) | 374 | 1.225579 | 6 | 1157.76 | 29.9007 |
| Vertical with machined surface (as-built) | 375 | 1.223957 | 6 | 1158.42 | 30.0984 |
| Vertical with machined surface (as-built) | 376 | 1.222329 | 6 | 1159.1 | 30.2971 |
| Vertical with machined surface (as-built) | 377 | 1.220693 | 6 | 1159.78 | 30.4969 |
| Vertical with machined surface (as-built) | 378 | 1.219049 | 6 | 1160.46 | 30.6979 |
| Vertical with machined surface (as-built) | 379 | 1.217398 | 6 | 1161.14 | 30.9001 |
| Vertical with machined surface (as-built) | 380 | 1.215739 | 6 | 1161.82 | 31.1036 |
| Vertical with machined surface (as-built) | 381 | 1.214075 | 6 | 1162.52 | 31.3083 |
| Vertical with machined surface (as-built) | 382 | 1.212406 | 6 | 1163.24 | 31.5144 |
| Vertical with machined surface (as-built) | 383 | 1.210736 | 6 | 1163.96 | 31.7219 |
| Vertical with machined surface (as-built) | 384 | 1.209066 | 6 | 1164.7 | 31.9308 |
| Vertical with machined surface (as-built) | 385 | 1.207398 | 6 | 1165.44 | 32.1411 |
| Vertical with machined surface (as-built) | 386 | 1.205734 | 6 | 1166.22 | 32.3527 |
| Vertical with machined surface (as-built) | 387 | 1.204075 | 6 | 1167 | 32.5656 |
| Vertical with machined surface (as-built) | 388 | 1.202419 | 6 | 1167.8 | 32.7798 |
| Vertical with machined surface (as-built) | 389 | 1.200764 | 6 | 1168.62 | 32.9952 |
| Vertical with machined surface (as-built) | 390 | 1.199113 | 6 | 1169.44 | 33.2118 |
| Vertical with machined surface (as-built) | 391 | 1.197465 | 6 | 1170.3 | 33.4296 |
| Vertical with machined surface (as-built) | 392 | 1.195823 | 6 | 1171.16 | 33.6486 |
| Vertical with machined surface (as-built) | 393 | 1.194188 | 6 | 1172.04 | 33.8686 |
| Vertical with machined surface (as-built) | 394 | 1.192562 | 6 | 1172.94 | 34.0897 |
| Vertical with machined surface (as-built) | 395 | 1.190943 | 6 | 1173.86 | 34.3119 |
| Vertical with machined surface (as-built) | 396 | 1.189332 | 6 | 1174.78 | 34.5351 |
| Vertical with machined surface (as-built) | 397 | 1.187728 | 6 | 1175.74 | 34.7592 |
| Vertical with machined surface (as-built) | 398 | 1.186129 | 6 | 1176.7 | 34.9843 |
| Vertical with machined surface (as-built) | 399 | 1.184535 | 6 | 1177.68 | 35.2103 |
| Vertical with machined surface (as-built) | 400 | 1.182943 | 6 | 1178.68 | 35.4371 |
| Vertical with machined surface (as-built) | 401 | 1.181352 | 6 | 1179.68 | 35.6648 |
| Vertical with machined surface (as-built) | 402 | 1.17976 | 6 | 1180.68 | 35.8933 |
| Vertical with machined surface (as-built) | 403 | 1.178165 | 6 | 1181.68 | 36.1226 |
| Vertical with machined surface (as-built) | 404 | 1.176565 | 6 | 1182.7 | 36.3529 |
| Vertical with machined surface (as-built) | 405 | 1.17496 | 6 | 1183.72 | 36.584 |
| Vertical with machined surface (as-built) | 406 | 1.17335 | 6 | 1184.74 | 36.8162 |
| Vertical with machined surface (as-built) | 407 | 1.171734 | 6 | 1185.76 | 37.0493 |
| Vertical with machined surface (as-built) | 408 | 1.170113 | 6 | 1186.8 | 37.2835 |
| Vertical with machined surface (as-built) | 409 | 1.168486 | 6 | 1187.82 | 37.5186 |
| Vertical with machined surface (as-built) | 410 | 1.166852 | 6 | 1188.86 | 37.7547 |
| Vertical with machined surface (as-built) | 411 | 1.165211 | 6 | 1189.9 | 37.9919 |
| Vertical with machined surface (as-built) | 412 | 1.163562 | 6 | 1190.94 | 38.2302 |
| Vertical with machined surface (as-built) | 413 | 1.161903 | 6 | 1191.98 | 38.4695 |
| Vertical with machined surface (as-built) | 414 | 1.160234 | 6 | 1193.02 | 38.7099 |
| Vertical with machined surface (as-built) | 415 | 1.158555 | 6 | 1194.06 | 38.9513 |
| Vertical with machined surface (as-built) | 416 | 1.156867 | 6 | 1195.1 | 39.1936 |
| Vertical with machined surface (as-built) | 417 | 1.15517 | 6 | 1196.16 | 39.4369 |
| Vertical with machined surface (as-built) | 418 | 1.153465 | 6 | 1197.2 | 39.6811 |
| Vertical with machined surface (as-built) | 419 | 1.151751 | 6 | 1198.24 | 39.9262 |
| Vertical with machined surface (as-built) | 420 | 1.150028 | 6 | 1199.28 | 40.1723 |
| Vertical with machined surface (as-built) | 421 | 1.148296 | 6 | 1200.32 | 40.4191 |
| Vertical with machined surface (as-built) | 422 | 1.146558 | 6 | 1201.36 | 40.6669 |
| Vertical with machined surface (as-built) | 423 | 1.144813 | 6 | 1202.4 | 40.9154 |
| Vertical with machined surface (as-built) | 424 | 1.143063 | 6 | 1203.46 | 41.1648 |
| Vertical with machined surface (as-built) | 425 | 1.141311 | 6 | 1204.5 | 41.4151 |
| Vertical with machined surface (as-built) | 426 | 1.139557 | 6 | 1205.56 | 41.6663 |
| Vertical with machined surface (as-built) | 427 | 1.1378 | 6 | 1206.64 | 41.9185 |
| Vertical with machined surface (as-built) | 428 | 1.136042 | 6 | 1207.7 | 42.1718 |
| Vertical with machined surface (as-built) | 429 | 1.134282 | 6 | 1208.8 | 42.4261 |
| Vertical with machined surface (as-built) | 430 | 1.132521 | 6 | 1209.88 | 42.6814 |
| Vertical with machined surface (as-built) | 431 | 1.13076 | 6 | 1211 | 42.9377 |
| Vertical with machined surface (as-built) | 432 | 1.128998 | 6 | 1212.1 | 43.1951 |
| Vertical with machined surface (as-built) | 433 | 1.127236 | 6 | 1213.22 | 43.4534 |
| Vertical with machined surface (as-built) | 434 | 1.125474 | 6 | 1214.36 | 43.7126 |
| Vertical with machined surface (as-built) | 435 | 1.12371 | 6 | 1215.5 | 43.9729 |
| Vertical with machined surface (as-built) | 436 | 1.121944 | 6 | 1216.64 | 44.2341 |
| Vertical with machined surface (as-built) | 437 | 1.120174 | 6 | 1217.8 | 44.4961 |
| Vertical with machined surface (as-built) | 438 | 1.1184 | 6 | 1218.94 | 44.7591 |
| Vertical with machined surface (as-built) | 439 | 1.116622 | 6 | 1220.1 | 45.0229 |
| Vertical with machined surface (as-built) | 440 | 1.114841 | 6 | 1221.26 | 45.2874 |
| Vertical with machined surface (as-built) | 441 | 1.11306 | 6 | 1222.42 | 45.5526 |
| Vertical with machined surface (as-built) | 442 | 1.111279 | 6 | 1223.6 | 45.8183 |
| Vertical with machined surface (as-built) | 443 | 1.1095 | 6 | 1224.78 | 46.0845 |
| Vertical with machined surface (as-built) | 444 | 1.107726 | 6 | 1225.96 | 46.3511 |
| Vertical with machined surface (as-built) | 445 | 1.105954 | 6 | 1227.14 | 46.6181 |
| Vertical with machined surface (as-built) | 446 | 1.104184 | 6 | 1228.34 | 46.8855 |
| Vertical with machined surface (as-built) | 447 | 1.102414 | 6 | 1229.52 | 47.1531 |
| Vertical with machined surface (as-built) | 448 | 1.100645 | 6 | 1230.72 | 47.421 |
| Vertical with machined surface (as-built) | 449 | 1.098875 | 6 | 1231.92 | 47.6892 |
| Vertical with machined surface (as-built) | 450 | 1.097105 | 6 | 1233.12 | 47.9577 |
| Vertical with machined surface (as-built) | 451 | 1.095332 | 6 | 1234.3 | 48.2264 |
| Vertical with machined surface (as-built) | 452 | 1.093556 | 6 | 1235.5 | 48.4953 |
| Vertical with machined surface (as-built) | 453 | 1.091776 | 6 | 1236.68 | 48.7646 |
| Vertical with machined surface (as-built) | 454 | 1.089989 | 6 | 1237.86 | 49.0342 |
| Vertical with machined surface (as-built) | 455 | 1.088196 | 6 | 1239.04 | 49.3041 |
| Vertical with machined surface (as-built) | 456 | 1.086394 | 6 | 1240.2 | 49.5745 |
| Vertical with machined surface (as-built) | 457 | 1.084582 | 6 | 1241.36 | 49.8453 |
| Vertical with machined surface (as-built) | 458 | 1.08276 | 6 | 1242.5 | 50.1168 |
| Vertical with machined surface (as-built) | 459 | 1.080926 | 6 | 1243.64 | 50.3889 |
| Vertical with machined surface (as-built) | 460 | 1.079079 | 6 | 1244.78 | 50.6617 |
| Vertical with machined surface (as-built) | 461 | 1.077219 | 6 | 1245.9 | 50.9355 |
| Vertical with machined surface (as-built) | 462 | 1.075347 | 6 | 1247.02 | 51.2102 |
| Vertical with machined surface (as-built) | 463 | 1.073462 | 6 | 1248.14 | 51.486 |
| Vertical with machined surface (as-built) | 464 | 1.071566 | 6 | 1249.26 | 51.7629 |
| Vertical with machined surface (as-built) | 465 | 1.06966 | 6 | 1250.36 | 52.041 |
| Vertical with machined surface (as-built) | 466 | 1.067745 | 6 | 1251.48 | 52.3204 |
| Vertical with machined surface (as-built) | 467 | 1.065823 | 6 | 1252.6 | 52.6011 |
| Vertical with machined surface (as-built) | 468 | 1.063895 | 6 | 1253.72 | 52.8831 |
| Vertical with machined surface (as-built) | 469 | 1.06196 | 6 | 1254.86 | 53.1666 |
| Vertical with machined surface (as-built) | 470 | 1.060018 | 6 | 1256 | 53.4515 |
| Vertical with machined surface (as-built) | 471 | 1.058069 | 6 | 1257.14 | 53.738 |
| Vertical with machined surface (as-built) | 472 | 1.056113 | 6 | 1258.28 | 54.0259 |
| Vertical with machined surface (as-built) | 473 | 1.054151 | 6 | 1259.44 | 54.3154 |
| Vertical with machined surface (as-built) | 474 | 1.052182 | 6 | 1260.6 | 54.6063 |
| Vertical with machined surface (as-built) | 475 | 1.050205 | 6 | 1261.78 | 54.8987 |
| Vertical with machined surface (as-built) | 476 | 1.048223 | 6 | 1262.94 | 55.1925 |
| Vertical with machined surface (as-built) | 477 | 1.046234 | 6 | 1264.12 | 55.4876 |
| Vertical with machined surface (as-built) | 478 | 1.04424 | 6 | 1265.32 | 55.784 |
| Vertical with machined surface (as-built) | 479 | 1.042242 | 6 | 1266.5 | 56.0817 |
| Vertical with machined surface (as-built) | 480 | 1.040236 | 6 | 1267.7 | 56.3804 |
| Vertical with machined surface (as-built) | 481 | 1.038224 | 6 | 1268.88 | 56.6801 |
| Vertical with machined surface (as-built) | 482 | 1.036202 | 6 | 1270.06 | 56.9806 |
| Vertical with machined surface (as-built) | 483 | 1.03417 | 6 | 1271.24 | 57.2819 |
| Vertical with machined surface (as-built) | 484 | 1.032125 | 6 | 1272.4 | 57.5839 |
| Vertical with machined surface (as-built) | 485 | 1.030067 | 6 | 1273.56 | 57.8865 |
| Vertical with machined surface (as-built) | 486 | 1.027995 | 6 | 1274.7 | 58.1897 |
| Vertical with machined surface (as-built) | 487 | 1.025912 | 6 | 1275.82 | 58.4935 |
| Vertical with machined surface (as-built) | 488 | 1.023819 | 6 | 1276.94 | 58.7979 |
| Vertical with machined surface (as-built) | 489 | 1.021716 | 6 | 1278.04 | 59.103 |
| Vertical with machined surface (as-built) | 490 | 1.019606 | 6 | 1279.14 | 59.4087 |
| Vertical with machined surface (as-built) | 491 | 1.017489 | 6 | 1280.24 | 59.7153 |
| Vertical with machined surface (as-built) | 492 | 1.015367 | 6 | 1281.34 | 60.0226 |
| Vertical with machined surface (as-built) | 493 | 1.013241 | 6 | 1282.44 | 60.3309 |
| Vertical with machined surface (as-built) | 494 | 1.011111 | 6 | 1283.54 | 60.6402 |
| Vertical with machined surface (as-built) | 495 | 1.008978 | 6 | 1284.64 | 60.9505 |
| Vertical with machined surface (as-built) | 496 | 1.006842 | 6 | 1285.76 | 61.2621 |
| Vertical with machined surface (as-built) | 497 | 1.004703 | 6 | 1286.88 | 61.5749 |
| Vertical with machined surface (as-built) | 498 | 1.00256 | 6 | 1288 | 61.8892 |
| Vertical with machined surface (as-built) | 499 | 1.000416 | 6 | 1289.14 | 62.205 |
| Vertical with machined surface (as-built) | 500 | 0.998269 | 6 | 1290.28 | 62.5224 |
| Vertical with machined surface (as-built) | 501 | 0.996118 | 6 | 1291.44 | 62.8415 |
| Vertical with machined surface (as-built) | 502 | 0.993963 | 6 | 1292.6 | 63.1625 |
| Vertical with machined surface (as-built) | 503 | 0.991803 | 6 | 1293.78 | 63.4854 |
| Vertical with machined surface (as-built) | 504 | 0.989634 | 6 | 1294.98 | 63.8102 |
| Vertical with machined surface (as-built) | 505 | 0.987456 | 6 | 1296.18 | 64.1372 |
| Vertical with machined surface (as-built) | 506 | 0.985266 | 6 | 1297.38 | 64.4664 |
| Vertical with machined surface (as-built) | 507 | 0.983066 | 6 | 1298.58 | 64.7978 |
| Vertical with machined surface (as-built) | 508 | 0.980855 | 6 | 1299.8 | 65.1315 |
| Vertical with machined surface (as-built) | 509 | 0.978636 | 6 | 1301.04 | 65.4677 |
| Vertical with machined surface (as-built) | 510 | 0.976412 | 6 | 1302.3 | 65.8061 |
| Vertical with machined surface (as-built) | 511 | 0.974184 | 6 | 1303.56 | 66.1468 |
| Vertical with machined surface (as-built) | 512 | 0.971955 | 6 | 1304.84 | 66.4896 |
| Vertical with machined surface (as-built) | 513 | 0.969725 | 6 | 1306.14 | 66.8345 |
| Vertical with machined surface (as-built) | 514 | 0.967496 | 6 | 1307.48 | 67.1813 |
| Vertical with machined surface (as-built) | 515 | 0.965266 | 6 | 1308.82 | 67.5299 |
| Vertical with machined surface (as-built) | 516 | 0.963036 | 6 | 1310.16 | 67.8802 |
| Vertical with machined surface (as-built) | 517 | 0.960803 | 6 | 1311.54 | 68.2321 |
| Vertical with machined surface (as-built) | 518 | 0.958568 | 6 | 1312.9 | 68.5854 |
| Vertical with machined surface (as-built) | 519 | 0.956328 | 6 | 1314.28 | 68.94 |
| Vertical with machined surface (as-built) | 520 | 0.954082 | 6 | 1315.66 | 69.2958 |
| Vertical with machined surface (as-built) | 521 | 0.951829 | 6 | 1317.04 | 69.6527 |
| Vertical with machined surface (as-built) | 522 | 0.94957 | 6 | 1318.44 | 70.0108 |
| Vertical with machined surface (as-built) | 523 | 0.947305 | 6 | 1319.82 | 70.3699 |
| Vertical with machined surface (as-built) | 524 | 0.945034 | 6 | 1321.2 | 70.7299 |
| Vertical with machined surface (as-built) | 525 | 0.94276 | 6 | 1322.58 | 71.0908 |
| Vertical with machined surface (as-built) | 526 | 0.940484 | 6 | 1323.96 | 71.4523 |
| Vertical with machined surface (as-built) | 527 | 0.938208 | 6 | 1325.36 | 71.8143 |
| Vertical with machined surface (as-built) | 528 | 0.935934 | 6 | 1326.74 | 72.1766 |
| Vertical with machined surface (as-built) | 529 | 0.933662 | 6 | 1328.14 | 72.5391 |
| Vertical with machined surface (as-built) | 530 | 0.931393 | 6 | 1329.54 | 72.9014 |
| Vertical with machined surface (as-built) | 531 | 0.929128 | 6 | 1330.94 | 73.2636 |
| Vertical with machined surface (as-built) | 532 | 0.926868 | 6 | 1332.34 | 73.6256 |
| Vertical with machined surface (as-built) | 533 | 0.924614 | 6 | 1333.74 | 73.9872 |
| Vertical with machined surface (as-built) | 534 | 0.922368 | 6 | 1335.14 | 74.3486 |
| Vertical with machined surface (as-built) | 535 | 0.92013 | 6 | 1336.58 | 74.7098 |
| Vertical with machined surface (as-built) | 536 | 0.917901 | 6 | 1338 | 75.0708 |
| Vertical with machined surface (as-built) | 537 | 0.915681 | 6 | 1339.44 | 75.4317 |
| Vertical with machined surface (as-built) | 538 | 0.913472 | 6 | 1340.92 | 75.7926 |
| Vertical with machined surface (as-built) | 539 | 0.911276 | 6 | 1342.4 | 76.1538 |
| Vertical with machined surface (as-built) | 540 | 0.909092 | 6 | 1343.9 | 76.5153 |
| Vertical with machined surface (as-built) | 541 | 0.906923 | 6 | 1345.44 | 76.8773 |
| Vertical with machined surface (as-built) | 542 | 0.90477 | 6 | 1347 | 77.2398 |
| Vertical with machined surface (as-built) | 543 | 0.902631 | 6 | 1348.6 | 77.603 |
| Vertical with machined surface (as-built) | 544 | 0.900505 | 6 | 1350.24 | 77.9667 |
| Vertical with machined surface (as-built) | 545 | 0.898392 | 6 | 1351.88 | 78.3312 |
| Vertical with machined surface (as-built) | 546 | 0.896286 | 6 | 1353.54 | 78.6963 |
| Vertical with machined surface (as-built) | 547 | 0.894185 | 6 | 1355.22 | 79.0624 |
| Vertical with machined surface (as-built) | 548 | 0.892082 | 6 | 1356.92 | 79.4293 |
| Vertical with machined surface (as-built) | 549 | 0.889975 | 6 | 1358.6 | 79.7973 |
| Vertical with machined surface (as-built) | 550 | 0.887856 | 6 | 1360.26 | 80.1665 |
| Vertical with machined surface (as-built) | 551 | 0.88572 | 6 | 1361.92 | 80.537 |
| Vertical with machined surface (as-built) | 552 | 0.88356 | 6 | 1363.52 | 80.9091 |
| Vertical with machined surface (as-built) | 553 | 0.881368 | 6 | 1365.1 | 81.283 |
| Vertical with machined surface (as-built) | 554 | 0.879133 | 6 | 1366.6 | 81.659 |
| Vertical with machined surface (as-built) | 555 | 0.876844 | 6 | 1368.04 | 82.0375 |
| Vertical with machined surface (as-built) | 556 | 0.874488 | 6 | 1369.36 | 82.4187 |
| Vertical with machined surface (as-built) | 557 | 0.872051 | 6 | 1370.56 | 82.8031 |
| Vertical with machined surface (as-built) | 558 | 0.869517 | 6 | 1371.62 | 83.1911 |
| Vertical with machined surface (as-built) | 559 | 0.866868 | 6 | 1372.5 | 83.5832 |
| Vertical with machined surface (as-built) | 560 | 0.864087 | 6 | 1373.16 | 83.98 |
| Vertical with machined surface (as-built) | 561 | 0.861153 | 6 | 1373.6 | 84.382 |
| Horizontal with machined surface (as-built) | 1 | 0 | 6 | 0 | 0 |
| Horizontal with machined surface (as-built) | 2 | 0.018051 | 6 | 12.8 | 0.0095 |
| Horizontal with machined surface (as-built) | 3 | 0.03611 | 6 | 25.62 | 0.019 |
| Horizontal with machined surface (as-built) | 4 | 0.05419 | 6 | 38.44 | 0.0285 |
| Horizontal with machined surface (as-built) | 5 | 0.072308 | 6 | 51.28 | 0.038 |
| Horizontal with machined surface (as-built) | 6 | 0.090486 | 6 | 64.18 | 0.0475 |
| Horizontal with machined surface (as-built) | 7 | 0.108745 | 6 | 77.14 | 0.057 |
| Horizontal with machined surface (as-built) | 8 | 0.127111 | 6 | 90.16 | 0.0665 |
| Horizontal with machined surface (as-built) | 9 | 0.145608 | 6 | 103.28 | 0.0759 |
| Horizontal with machined surface (as-built) | 10 | 0.16426 | 6 | 116.5 | 0.0854 |
| Horizontal with machined surface (as-built) | 11 | 0.18309 | 6 | 129.86 | 0.0949 |
| Horizontal with machined surface (as-built) | 12 | 0.202118 | 6 | 143.36 | 0.1043 |
| Horizontal with machined surface (as-built) | 13 | 0.221362 | 6 | 157.02 | 0.1137 |
| Horizontal with machined surface (as-built) | 14 | 0.240834 | 6 | 170.82 | 0.1231 |
| Horizontal with machined surface (as-built) | 15 | 0.260544 | 6 | 184.82 | 0.1324 |
| Horizontal with machined surface (as-built) | 16 | 0.280497 | 6 | 198.98 | 0.1416 |
| Horizontal with machined surface (as-built) | 17 | 0.300692 | 6 | 213.3 | 0.1508 |
| Horizontal with machined surface (as-built) | 18 | 0.321129 | 6 | 227.8 | 0.1599 |
| Horizontal with machined surface (as-built) | 19 | 0.341801 | 6 | 242.48 | 0.1689 |
| Horizontal with machined surface (as-built) | 20 | 0.362703 | 6 | 257.3 | 0.1778 |
| Horizontal with machined surface (as-built) | 21 | 0.383822 | 6 | 272.3 | 0.1866 |
| Horizontal with machined surface (as-built) | 22 | 0.405148 | 6 | 287.44 | 0.1954 |
| Horizontal with machined surface (as-built) | 23 | 0.426666 | 6 | 302.72 | 0.204 |
| Horizontal with machined surface (as-built) | 24 | 0.448361 | 6 | 318.12 | 0.2126 |
| Horizontal with machined surface (as-built) | 25 | 0.470216 | 6 | 333.64 | 0.221 |
| Horizontal with machined surface (as-built) | 26 | 0.492216 | 6 | 349.26 | 0.2294 |
| Horizontal with machined surface (as-built) | 27 | 0.514343 | 6 | 364.96 | 0.2377 |
| Horizontal with machined surface (as-built) | 28 | 0.536578 | 6 | 380.76 | 0.2459 |
| Horizontal with machined surface (as-built) | 29 | 0.558903 | 6 | 396.62 | 0.254 |
| Horizontal with machined surface (as-built) | 30 | 0.5813 | 6 | 412.54 | 0.262 |
| Horizontal with machined surface (as-built) | 31 | 0.603751 | 6 | 428.48 | 0.27 |
| Horizontal with machined surface (as-built) | 32 | 0.626237 | 6 | 444.46 | 0.2779 |
| Horizontal with machined surface (as-built) | 33 | 0.64874 | 6 | 460.46 | 0.2857 |
| Horizontal with machined surface (as-built) | 34 | 0.67124 | 6 | 476.44 | 0.2934 |
| Horizontal with machined surface (as-built) | 35 | 0.693719 | 6 | 492.42 | 0.3011 |
| Horizontal with machined surface (as-built) | 36 | 0.716158 | 6 | 508.38 | 0.3087 |
| Horizontal with machined surface (as-built) | 37 | 0.738538 | 6 | 524.3 | 0.3163 |
| Horizontal with machined surface (as-built) | 38 | 0.760839 | 6 | 540.16 | 0.3238 |
| Horizontal with machined surface (as-built) | 39 | 0.783042 | 6 | 555.96 | 0.3313 |
| Horizontal with machined surface (as-built) | 40 | 0.805126 | 6 | 571.66 | 0.3388 |
| Horizontal with machined surface (as-built) | 41 | 0.827072 | 6 | 587.28 | 0.3462 |
| Horizontal with machined surface (as-built) | 42 | 0.848857 | 6 | 602.78 | 0.3536 |
| Horizontal with machined surface (as-built) | 43 | 0.870461 | 6 | 618.16 | 0.3611 |
| Horizontal with machined surface (as-built) | 44 | 0.891861 | 6 | 633.4 | 0.3685 |
| Horizontal with machined surface (as-built) | 45 | 0.913037 | 6 | 648.48 | 0.3759 |
| Horizontal with machined surface (as-built) | 46 | 0.933964 | 6 | 663.38 | 0.3832 |
| Horizontal with machined surface (as-built) | 47 | 0.954621 | 6 | 678.08 | 0.3906 |
| Horizontal with machined surface (as-built) | 48 | 0.974983 | 6 | 692.6 | 0.398 |
| Horizontal with machined surface (as-built) | 49 | 0.995029 | 6 | 706.88 | 0.4053 |
| Horizontal with machined surface (as-built) | 50 | 1.014737 | 6 | 720.92 | 0.4126 |
| Horizontal with machined surface (as-built) | 51 | 1.034084 | 6 | 734.72 | 0.4199 |
| Horizontal with machined surface (as-built) | 52 | 1.05305 | 6 | 748.24 | 0.4272 |
| Horizontal with machined surface (as-built) | 53 | 1.071615 | 6 | 761.48 | 0.4345 |
| Horizontal with machined surface (as-built) | 54 | 1.08976 | 6 | 774.42 | 0.4417 |
| Horizontal with machined surface (as-built) | 55 | 1.107468 | 6 | 787.06 | 0.449 |
| Horizontal with machined surface (as-built) | 56 | 1.124723 | 6 | 799.38 | 0.4562 |
| Horizontal with machined surface (as-built) | 57 | 1.141508 | 6 | 811.36 | 0.4634 |
| Horizontal with machined surface (as-built) | 58 | 1.15781 | 6 | 823 | 0.4707 |
| Horizontal with machined surface (as-built) | 59 | 1.173616 | 6 | 834.28 | 0.4779 |
| Horizontal with machined surface (as-built) | 60 | 1.188915 | 6 | 845.22 | 0.4852 |
| Horizontal with machined surface (as-built) | 61 | 1.203698 | 6 | 855.78 | 0.4925 |
| Horizontal with machined surface (as-built) | 62 | 1.217957 | 6 | 865.98 | 0.4998 |
| Horizontal with machined surface (as-built) | 63 | 1.231687 | 6 | 875.8 | 0.5072 |
| Horizontal with machined surface (as-built) | 64 | 1.244884 | 6 | 885.24 | 0.5145 |
| Horizontal with machined surface (as-built) | 65 | 1.257548 | 6 | 894.3 | 0.522 |
| Horizontal with machined surface (as-built) | 66 | 1.26968 | 6 | 903 | 0.5294 |
| Horizontal with machined surface (as-built) | 67 | 1.281282 | 6 | 911.3 | 0.5368 |
| Horizontal with machined surface (as-built) | 68 | 1.292359 | 6 | 919.24 | 0.5442 |
| Horizontal with machined surface (as-built) | 69 | 1.302917 | 6 | 926.82 | 0.5517 |
| Horizontal with machined surface (as-built) | 70 | 1.312966 | 6 | 934.04 | 0.5592 |
| Horizontal with machined surface (as-built) | 71 | 1.322514 | 6 | 940.88 | 0.5667 |
| Horizontal with machined surface (as-built) | 72 | 1.331573 | 6 | 947.4 | 0.5742 |
| Horizontal with machined surface (as-built) | 73 | 1.340156 | 6 | 953.58 | 0.5818 |
| Horizontal with machined surface (as-built) | 74 | 1.348277 | 6 | 959.42 | 0.5895 |
| Horizontal with machined surface (as-built) | 75 | 1.355951 | 6 | 964.94 | 0.5972 |
| Horizontal with machined surface (as-built) | 76 | 1.363191 | 6 | 970.18 | 0.6051 |
| Horizontal with machined surface (as-built) | 77 | 1.370014 | 6 | 975.1 | 0.6131 |
| Horizontal with machined surface (as-built) | 78 | 1.376435 | 6 | 979.74 | 0.6212 |
| Horizontal with machined surface (as-built) | 79 | 1.38247 | 6 | 984.12 | 0.6294 |
| Horizontal with machined surface (as-built) | 80 | 1.388135 | 6 | 988.22 | 0.6379 |
| Horizontal with machined surface (as-built) | 81 | 1.393448 | 6 | 992.1 | 0.6466 |
| Horizontal with machined surface (as-built) | 82 | 1.398426 | 6 | 995.72 | 0.6555 |
| Horizontal with machined surface (as-built) | 83 | 1.403086 | 6 | 999.12 | 0.6646 |
| Horizontal with machined surface (as-built) | 84 | 1.407446 | 6 | 1002.32 | 0.674 |
| Horizontal with machined surface (as-built) | 85 | 1.411523 | 6 | 1005.3 | 0.6836 |
| Horizontal with machined surface (as-built) | 86 | 1.415333 | 6 | 1008.12 | 0.6935 |
| Horizontal with machined surface (as-built) | 87 | 1.418893 | 6 | 1010.74 | 0.7037 |
| Horizontal with machined surface (as-built) | 88 | 1.422218 | 6 | 1013.22 | 0.7142 |
| Horizontal with machined surface (as-built) | 89 | **1.425322** | 6 | **1015.54** | **0.725** |
| Horizontal with machined surface (as-built) | 90 | 1.428222 | 6 | 1017.7 | 0.736 |
| Horizontal with machined surface (as-built) | 91 | 1.430932 | 6 | 1019.74 | 0.7474 |
| Horizontal with machined surface (as-built) | 92 | 1.433466 | 6 | 1021.66 | 0.759 |
| Horizontal with machined surface (as-built) | 93 | 1.435838 | 6 | 1023.46 | 0.7708 |
| Horizontal with machined surface (as-built) | 94 | 1.438059 | 6 | 1025.16 | 0.7828 |
| Horizontal with machined surface (as-built) | 95 | 1.440142 | 6 | 1026.76 | 0.7951 |
| Horizontal with machined surface (as-built) | 96 | 1.442098 | 6 | 1028.28 | 0.8076 |
| Horizontal with machined surface (as-built) | 97 | 1.443937 | 6 | 1029.72 | 0.8203 |
| Horizontal with machined surface (as-built) | 98 | 1.445669 | 6 | 1031.08 | 0.8331 |
| Horizontal with machined surface (as-built) | 99 | 1.447304 | 6 | 1032.36 | 0.8462 |
| Horizontal with machined surface (as-built) | 100 | 1.448851 | 6 | 1033.6 | 0.8593 |
| Horizontal with machined surface (as-built) | 101 | 1.450318 | 6 | 1034.78 | 0.8727 |
| Horizontal with machined surface (as-built) | 102 | 1.451712 | 6 | 1035.9 | 0.8861 |
| Horizontal with machined surface (as-built) | 103 | 1.453041 | 6 | 1036.98 | 0.8996 |
| Horizontal with machined surface (as-built) | 104 | 1.454312 | 6 | 1038.02 | 0.9132 |
| Horizontal with machined surface (as-built) | 105 | 1.455529 | 6 | 1039.02 | 0.9268 |
| Horizontal with machined surface (as-built) | 106 | 1.456699 | 6 | 1039.98 | 0.9404 |
| Horizontal with machined surface (as-built) | 107 | 1.457826 | 6 | 1040.92 | 0.954 |
| Horizontal with machined surface (as-built) | 108 | 1.458915 | 6 | 1041.84 | 0.9676 |
| Horizontal with machined surface (as-built) | 109 | 1.459972 | 6 | 1042.72 | 0.9812 |
| Horizontal with machined surface (as-built) | 110 | 1.461 | 6 | 1043.58 | 0.9947 |
| Horizontal with machined surface (as-built) | 111 | 1.462004 | 6 | 1044.42 | 1.0081 |
| Horizontal with machined surface (as-built) | 112 | 1.462987 | 6 | 1045.26 | 1.0214 |
| Horizontal with machined surface (as-built) | 113 | 1.463953 | 6 | 1046.08 | 1.0345 |
| Horizontal with machined surface (as-built) | 114 | 1.464904 | 6 | 1046.88 | 1.0475 |
| Horizontal with machined surface (as-built) | 115 | 1.465845 | 6 | 1047.68 | 1.0604 |
| Horizontal with machined surface (as-built) | 116 | 1.466776 | 6 | 1048.48 | 1.0731 |
| Horizontal with machined surface (as-built) | 117 | 1.4677 | 6 | 1049.26 | 1.0856 |
| Horizontal with machined surface (as-built) | 118 | 1.468617 | 6 | 1050.04 | 1.0979 |
| Horizontal with machined surface (as-built) | 119 | 1.469528 | 6 | 1050.8 | 1.1101 |
| Horizontal with machined surface (as-built) | 120 | 1.470434 | 6 | 1051.58 | 1.1221 |
| Horizontal with machined surface (as-built) | 121 | 1.471335 | 6 | 1052.34 | 1.1339 |
| Horizontal with machined surface (as-built) | 122 | 1.472233 | 6 | 1053.1 | 1.1456 |
| Horizontal with machined surface (as-built) | 123 | 1.473127 | 6 | 1053.84 | 1.1571 |
| Horizontal with machined surface (as-built) | 124 | 1.474019 | 6 | 1054.6 | 1.1685 |
| Horizontal with machined surface (as-built) | 125 | 1.474908 | 6 | 1055.34 | 1.1797 |
| Horizontal with machined surface (as-built) | 126 | 1.475796 | 6 | 1056.08 | 1.1908 |
| Horizontal with machined surface (as-built) | 127 | 1.476683 | 6 | 1056.84 | 1.2018 |
| Horizontal with machined surface (as-built) | 128 | 1.477569 | 6 | 1057.58 | 1.2126 |
| Horizontal with machined surface (as-built) | 129 | 1.478454 | 6 | 1058.32 | 1.2233 |
| Horizontal with machined surface (as-built) | 130 | 1.47934 | 6 | 1059.06 | 1.2339 |
| Horizontal with machined surface (as-built) | 131 | 1.480226 | 6 | 1059.8 | 1.2445 |
| Horizontal with machined surface (as-built) | 132 | 1.481113 | 6 | 1060.54 | 1.2549 |
| Horizontal with machined surface (as-built) | 133 | 1.482 | 6 | 1061.28 | 1.2652 |
| Horizontal with machined surface (as-built) | 134 | 1.48289 | 6 | 1062.02 | 1.2754 |
| Horizontal with machined surface (as-built) | 135 | 1.483782 | 6 | 1062.76 | 1.2856 |
| Horizontal with machined surface (as-built) | 136 | 1.484676 | 6 | 1063.5 | 1.2956 |
| Horizontal with machined surface (as-built) | 137 | 1.485575 | 6 | 1064.24 | 1.3055 |
| Horizontal with machined surface (as-built) | 138 | 1.486479 | 6 | 1065 | 1.3154 |
| Horizontal with machined surface (as-built) | 139 | 1.487388 | 6 | 1065.74 | 1.3252 |
| Horizontal with machined surface (as-built) | 140 | 1.488304 | 6 | 1066.5 | 1.3349 |
| Horizontal with machined surface (as-built) | 141 | 1.489225 | 6 | 1067.26 | 1.3446 |
| Horizontal with machined surface (as-built) | 142 | 1.490154 | 6 | 1068.02 | 1.3543 |
| Horizontal with machined surface (as-built) | 143 | 1.491089 | 6 | 1068.8 | 1.3639 |
| Horizontal with machined surface (as-built) | 144 | 1.492032 | 6 | 1069.56 | 1.3735 |
| Horizontal with machined surface (as-built) | 145 | 1.492983 | 6 | 1070.34 | 1.3832 |
| Horizontal with machined surface (as-built) | 146 | 1.493943 | 6 | 1071.14 | 1.3928 |
| Horizontal with machined surface (as-built) | 147 | 1.494913 | 6 | 1071.94 | 1.4025 |
| Horizontal with machined surface (as-built) | 148 | 1.495895 | 6 | 1072.74 | 1.4123 |
| Horizontal with machined surface (as-built) | 149 | 1.49689 | 6 | 1073.54 | 1.4222 |
| Horizontal with machined surface (as-built) | 150 | 1.497899 | 6 | 1074.38 | 1.4322 |
| Horizontal with machined surface (as-built) | 151 | 1.498925 | 6 | 1075.22 | 1.4423 |
| Horizontal with machined surface (as-built) | 152 | 1.499968 | 6 | 1076.06 | 1.4526 |
| Horizontal with machined surface (as-built) | 153 | 1.50103 | 6 | 1076.94 | 1.463 |
| Horizontal with machined surface (as-built) | 154 | 1.502112 | 6 | 1077.82 | 1.4736 |
| Horizontal with machined surface (as-built) | 155 | 1.503215 | 6 | 1078.72 | 1.4844 |
| Horizontal with machined surface (as-built) | 156 | 1.504338 | 6 | 1079.64 | 1.4953 |
| Horizontal with machined surface (as-built) | 157 | 1.505483 | 6 | 1080.58 | 1.5064 |
| Horizontal with machined surface (as-built) | 158 | 1.506649 | 6 | 1081.52 | 1.5177 |
| Horizontal with machined surface (as-built) | 159 | 1.507838 | 6 | 1082.5 | 1.5291 |
| Horizontal with machined surface (as-built) | 160 | 1.509051 | 6 | 1083.48 | 1.5407 |
| Horizontal with machined surface (as-built) | 161 | 1.510287 | 6 | 1084.5 | 1.5525 |
| Horizontal with machined surface (as-built) | 162 | 1.511548 | 6 | 1085.52 | 1.5645 |
| Horizontal with machined surface (as-built) | 163 | 1.512835 | 6 | 1086.58 | 1.5766 |
| Horizontal with machined surface (as-built) | 164 | 1.514147 | 6 | 1087.64 | 1.5889 |
| Horizontal with machined surface (as-built) | 165 | 1.515484 | 6 | 1088.74 | 1.6013 |
| Horizontal with machined surface (as-built) | 166 | 1.516846 | 6 | 1089.84 | 1.6139 |
| Horizontal with machined surface (as-built) | 167 | 1.518232 | 6 | 1090.96 | 1.6267 |
| Horizontal with machined surface (as-built) | 168 | 1.519642 | 6 | 1092.12 | 1.6396 |
| Horizontal with machined surface (as-built) | 169 | 1.521075 | 6 | 1093.28 | 1.6527 |
| Horizontal with machined surface (as-built) | 170 | 1.522528 | 6 | 1094.46 | 1.666 |
| Horizontal with machined surface (as-built) | 171 | 1.524002 | 6 | 1095.66 | 1.6795 |
| Horizontal with machined surface (as-built) | 172 | 1.525493 | 6 | 1096.88 | 1.6931 |
| Horizontal with machined surface (as-built) | 173 | 1.526998 | 6 | 1098.1 | 1.707 |
| Horizontal with machined surface (as-built) | 174 | 1.528513 | 6 | 1099.34 | 1.721 |
| Horizontal with machined surface (as-built) | 175 | 1.530036 | 6 | 1100.58 | 1.7353 |
| Horizontal with machined surface (as-built) | 176 | 1.531562 | 6 | 1101.82 | 1.7498 |
| Horizontal with machined surface (as-built) | 177 | 1.533089 | 6 | 1103.08 | 1.7645 |
| Horizontal with machined surface (as-built) | 178 | 1.534614 | 6 | 1104.34 | 1.7795 |
| Horizontal with machined surface (as-built) | 179 | 1.536133 | 6 | 1105.58 | 1.7947 |
| Horizontal with machined surface (as-built) | 180 | 1.537645 | 6 | 1106.84 | 1.8101 |
| Horizontal with machined surface (as-built) | 181 | 1.539147 | 6 | 1108.08 | 1.8259 |
| Horizontal with machined surface (as-built) | 182 | 1.540637 | 6 | 1109.32 | 1.8419 |
| Horizontal with machined surface (as-built) | 183 | 1.542114 | 6 | 1110.56 | 1.8583 |
| Horizontal with machined surface (as-built) | 184 | 1.543576 | 6 | 1111.8 | 1.8749 |
| Horizontal with machined surface (as-built) | 185 | 1.545022 | 6 | 1113.02 | 1.8918 |
| Horizontal with machined surface (as-built) | 186 | 1.546451 | 6 | 1114.22 | 1.9091 |
| Horizontal with machined surface (as-built) | 187 | 1.547861 | 6 | 1115.42 | 1.9266 |
| Horizontal with machined surface (as-built) | 188 | 1.549252 | 6 | 1116.62 | 1.9445 |
| Horizontal with machined surface (as-built) | 189 | 1.550623 | 6 | 1117.8 | 1.9627 |
| Horizontal with machined surface (as-built) | 190 | 1.551974 | 6 | 1118.96 | 1.9812 |
| Horizontal with machined surface (as-built) | 191 | 1.553303 | 6 | 1120.12 | 1.9999 |
| Horizontal with machined surface (as-built) | 192 | 1.554611 | 6 | 1121.26 | 2.019 |
| Horizontal with machined surface (as-built) | 193 | 1.555898 | 6 | 1122.4 | 2.0383 |
| Horizontal with machined surface (as-built) | 194 | 1.557165 | 6 | 1123.52 | 2.0578 |
| Horizontal with machined surface (as-built) | 195 | 1.558411 | 6 | 1124.64 | 2.0776 |
| Horizontal with machined surface (as-built) | 196 | 1.559637 | 6 | 1125.74 | 2.0975 |
| Horizontal with machined surface (as-built) | 197 | 1.560844 | 6 | 1126.82 | 2.1177 |
| Horizontal with machined surface (as-built) | 198 | 1.562031 | 6 | 1127.9 | 2.1379 |
| Horizontal with machined surface (as-built) | 199 | 1.5632 | 6 | 1128.96 | 2.1583 |
| Horizontal with machined surface (as-built) | 200 | 1.564351 | 6 | 1130 | 2.1788 |
| Horizontal with machined surface (as-built) | 201 | 1.565484 | 6 | 1131.04 | 2.1994 |
| Horizontal with machined surface (as-built) | 202 | 1.566602 | 6 | 1132.08 | 2.2201 |
| Horizontal with machined surface (as-built) | 203 | 1.567704 | 6 | 1133.08 | 2.2407 |
| Horizontal with machined surface (as-built) | 204 | 1.568792 | 6 | 1134.1 | 2.2615 |
| Horizontal with machined surface (as-built) | 205 | 1.569866 | 6 | 1135.1 | 2.2822 |
| Horizontal with machined surface (as-built) | 206 | 1.570927 | 6 | 1136.08 | 2.303 |
| Horizontal with machined surface (as-built) | 207 | 1.571975 | 6 | 1137.06 | 2.3237 |
| Horizontal with machined surface (as-built) | 208 | 1.573011 | 6 | 1138.04 | 2.3445 |
| Horizontal with machined surface (as-built) | 209 | 1.574034 | 6 | 1139 | 2.3652 |
| Horizontal with machined surface (as-built) | 210 | 1.575046 | 6 | 1139.96 | 2.3859 |
| Horizontal with machined surface (as-built) | 211 | 1.576046 | 6 | 1140.9 | 2.4066 |
| Horizontal with machined surface (as-built) | 212 | 1.577035 | 6 | 1141.84 | 2.4272 |
| Horizontal with machined surface (as-built) | 213 | 1.578014 | 6 | 1142.78 | 2.4477 |
| Horizontal with machined surface (as-built) | 214 | 1.578981 | 6 | 1143.7 | 2.4682 |
| Horizontal with machined surface (as-built) | 215 | 1.579936 | 6 | 1144.6 | 2.4885 |
| Horizontal with machined surface (as-built) | 216 | 1.580878 | 6 | 1145.52 | 2.5088 |
| Horizontal with machined surface (as-built) | 217 | 1.581808 | 6 | 1146.4 | 2.5291 |
| Horizontal with machined surface (as-built) | 218 | 1.582724 | 6 | 1147.28 | 2.5493 |
| Horizontal with machined surface (as-built) | 219 | 1.583626 | 6 | 1148.16 | 2.5694 |
| Horizontal with machined surface (as-built) | 220 | 1.584515 | 6 | 1149.02 | 2.5894 |
| Horizontal with machined surface (as-built) | 221 | 1.585389 | 6 | 1149.88 | 2.6095 |
| Horizontal with machined surface (as-built) | 222 | 1.586249 | 6 | 1150.72 | 2.6295 |
| Horizontal with machined surface (as-built) | 223 | 1.587093 | 6 | 1151.54 | 2.6496 |
| Horizontal with machined surface (as-built) | 224 | 1.587924 | 6 | 1152.36 | 2.6697 |
| Horizontal with machined surface (as-built) | 225 | 1.588742 | 6 | 1153.18 | 2.6897 |
| Horizontal with machined surface (as-built) | 226 | 1.589547 | 6 | 1153.98 | 2.7099 |
| Horizontal with machined surface (as-built) | 227 | 1.590342 | 6 | 1154.78 | 2.73 |
| Horizontal with machined surface (as-built) | 228 | 1.591126 | 6 | 1155.56 | 2.7502 |
| Horizontal with machined surface (as-built) | 229 | 1.591902 | 6 | 1156.34 | 2.7704 |
| Horizontal with machined surface (as-built) | 230 | 1.592671 | 6 | 1157.12 | 2.7906 |
| Horizontal with machined surface (as-built) | 231 | 1.593431 | 6 | 1157.9 | 2.8109 |
| Horizontal with machined surface (as-built) | 232 | 1.594185 | 6 | 1158.68 | 2.8312 |
| Horizontal with machined surface (as-built) | 233 | 1.594933 | 6 | 1159.44 | 2.8515 |
| Horizontal with machined surface (as-built) | 234 | 1.595673 | 6 | 1160.2 | 2.8718 |
| Horizontal with machined surface (as-built) | 235 | 1.596408 | 6 | 1160.96 | 2.8922 |
| Horizontal with machined surface (as-built) | 236 | 1.597136 | 6 | 1161.72 | 2.9126 |
| Horizontal with machined surface (as-built) | 237 | 1.597859 | 6 | 1162.46 | 2.9329 |
| Horizontal with machined surface (as-built) | 238 | 1.598576 | 6 | 1163.2 | 2.9533 |
| Horizontal with machined surface (as-built) | 239 | 1.599288 | 6 | 1163.94 | 2.9736 |
| Horizontal with machined surface (as-built) | 240 | 1.599995 | 6 | 1164.68 | 2.9939 |
| Horizontal with machined surface (as-built) | 241 | 1.600698 | 6 | 1165.42 | 3.0142 |
| Horizontal with machined surface (as-built) | 242 | 1.601397 | 6 | 1166.16 | 3.0345 |
| Horizontal with machined surface (as-built) | 243 | 1.602092 | 6 | 1166.88 | 3.0547 |
| Horizontal with machined surface (as-built) | 244 | 1.602782 | 6 | 1167.62 | 3.075 |
| Horizontal with machined surface (as-built) | 245 | 1.603469 | 6 | 1168.34 | 3.0951 |
| Horizontal with machined surface (as-built) | 246 | 1.604151 | 6 | 1169.06 | 3.1153 |
| Horizontal with machined surface (as-built) | 247 | 1.60483 | 6 | 1169.78 | 3.1355 |
| Horizontal with machined surface (as-built) | 248 | 1.605505 | 6 | 1170.48 | 3.1556 |
| Horizontal with machined surface (as-built) | 249 | 1.606176 | 6 | 1171.2 | 3.1758 |
| Horizontal with machined surface (as-built) | 250 | 1.606842 | 6 | 1171.92 | 3.1959 |
| Horizontal with machined surface (as-built) | 251 | 1.607504 | 6 | 1172.62 | 3.2161 |
| Horizontal with machined surface (as-built) | 252 | 1.608162 | 6 | 1173.32 | 3.2363 |
| Horizontal with machined surface (as-built) | 253 | 1.608815 | 6 | 1174.02 | 3.2565 |
| Horizontal with machined surface (as-built) | 254 | 1.609463 | 6 | 1174.72 | 3.2768 |
| Horizontal with machined surface (as-built) | 255 | 1.610108 | 6 | 1175.42 | 3.2971 |
| Horizontal with machined surface (as-built) | 256 | 1.610749 | 6 | 1176.12 | 3.3174 |
| Horizontal with machined surface (as-built) | 257 | 1.611386 | 6 | 1176.8 | 3.3378 |
| Horizontal with machined surface (as-built) | 258 | 1.612019 | 6 | 1177.5 | 3.3583 |
| Horizontal with machined surface (as-built) | 259 | 1.612648 | 6 | 1178.18 | 3.3788 |
| Horizontal with machined surface (as-built) | 260 | 1.61327 | 6 | 1178.88 | 3.3994 |
| Horizontal with machined surface (as-built) | 261 | 1.613886 | 6 | 1179.56 | 3.42 |
| Horizontal with machined surface (as-built) | 262 | 1.614494 | 6 | 1180.22 | 3.4407 |
| Horizontal with machined surface (as-built) | 263 | 1.615095 | 6 | 1180.9 | 3.4614 |
| Horizontal with machined surface (as-built) | 264 | 1.615689 | 6 | 1181.56 | 3.4822 |
| Horizontal with machined surface (as-built) | 265 | 1.616275 | 6 | 1182.22 | 3.503 |
| Horizontal with machined surface (as-built) | 266 | 1.616854 | 6 | 1182.88 | 3.5239 |
| Horizontal with machined surface (as-built) | 267 | 1.617428 | 6 | 1183.54 | 3.5448 |
| Horizontal with machined surface (as-built) | 268 | 1.617996 | 6 | 1184.2 | 3.5657 |
| Horizontal with machined surface (as-built) | 269 | 1.618561 | 6 | 1184.84 | 3.5866 |
| Horizontal with machined surface (as-built) | 270 | 1.619122 | 6 | 1185.48 | 3.6075 |
| Horizontal with machined surface (as-built) | 271 | 1.61968 | 6 | 1186.12 | 3.6284 |
| Horizontal with machined surface (as-built) | 272 | 1.620234 | 6 | 1186.76 | 3.6493 |
| Horizontal with machined surface (as-built) | 273 | 1.620785 | 6 | 1187.4 | 3.6702 |
| Horizontal with machined surface (as-built) | 274 | 1.621333 | 6 | 1188.04 | 3.691 |
| Horizontal with machined surface (as-built) | 275 | 1.621877 | 6 | 1188.68 | 3.7118 |
| Horizontal with machined surface (as-built) | 276 | 1.622417 | 6 | 1189.3 | 3.7326 |
| Horizontal with machined surface (as-built) | 277 | 1.622952 | 6 | 1189.94 | 3.7534 |
| Horizontal with machined surface (as-built) | 278 | 1.623484 | 6 | 1190.56 | 3.7742 |
| Horizontal with machined surface (as-built) | 279 | 1.624011 | 6 | 1191.18 | 3.795 |
| Horizontal with machined surface (as-built) | 280 | 1.624535 | 6 | 1191.8 | 3.8158 |
| Horizontal with machined surface (as-built) | 281 | 1.625055 | 6 | 1192.42 | 3.8366 |
| Horizontal with machined surface (as-built) | 282 | 1.625571 | 6 | 1193.02 | 3.8574 |
| Horizontal with machined surface (as-built) | 283 | 1.626083 | 6 | 1193.64 | 3.8783 |
| Horizontal with machined surface (as-built) | 284 | 1.626591 | 6 | 1194.24 | 3.8992 |
| Horizontal with machined surface (as-built) | 285 | 1.627094 | 6 | 1194.86 | 3.9202 |
| Horizontal with machined surface (as-built) | 286 | 1.627591 | 6 | 1195.46 | 3.9412 |
| Horizontal with machined surface (as-built) | 287 | 1.628083 | 6 | 1196.06 | 3.9623 |
| Horizontal with machined surface (as-built) | 288 | 1.628569 | 6 | 1196.66 | 3.9834 |
| Horizontal with machined surface (as-built) | 289 | 1.62905 | 6 | 1197.24 | 4.0046 |
| Horizontal with machined surface (as-built) | 290 | 1.629524 | 6 | 1197.84 | 4.0258 |
| Horizontal with machined surface (as-built) | 291 | 1.629993 | 6 | 1198.42 | 4.0471 |
| Horizontal with machined surface (as-built) | 292 | 1.630455 | 6 | 1199 | 4.0684 |
| Horizontal with machined surface (as-built) | 293 | 1.63091 | 6 | 1199.58 | 4.0896 |
| Horizontal with machined surface (as-built) | 294 | 1.631358 | 6 | 1200.14 | 4.1109 |
| Horizontal with machined surface (as-built) | 295 | 1.631797 | 6 | 1200.72 | 4.1321 |
| Horizontal with machined surface (as-built) | 296 | 1.632228 | 6 | 1201.26 | 4.1533 |
| Horizontal with machined surface (as-built) | 297 | 1.632649 | 6 | 1201.82 | 4.1744 |
| Horizontal with machined surface (as-built) | 298 | 1.63306 | 6 | 1202.36 | 4.1956 |
| Horizontal with machined surface (as-built) | 299 | 1.633461 | 6 | 1202.9 | 4.2166 |
| Horizontal with machined surface (as-built) | 300 | 1.633852 | 6 | 1203.42 | 4.2377 |
| Horizontal with machined surface (as-built) | 301 | 1.634233 | 6 | 1203.94 | 4.2587 |
| Horizontal with machined surface (as-built) | 302 | 1.634604 | 6 | 1204.46 | 4.2798 |
| Horizontal with machined surface (as-built) | 303 | 1.634967 | 6 | 1204.96 | 4.3009 |
| Horizontal with machined surface (as-built) | 304 | 1.635322 | 6 | 1205.46 | 4.322 |
| Horizontal with machined surface (as-built) | 305 | 1.63567 | 6 | 1205.96 | 4.3431 |
| Horizontal with machined surface (as-built) | 306 | 1.636012 | 6 | 1206.46 | 4.3643 |
| Horizontal with machined surface (as-built) | 307 | 1.636348 | 6 | 1206.94 | 4.3856 |
| Horizontal with machined surface (as-built) | 308 | 1.636681 | 6 | 1207.44 | 4.407 |
| Horizontal with machined surface (as-built) | 309 | 1.637009 | 6 | 1207.92 | 4.4284 |
| Horizontal with machined surface (as-built) | 310 | 1.637334 | 6 | 1208.42 | 4.45 |
| Horizontal with machined surface (as-built) | 311 | 1.637657 | 6 | 1208.9 | 4.4716 |
| Horizontal with machined surface (as-built) | 312 | 1.637978 | 6 | 1209.38 | 4.4934 |
| Horizontal with machined surface (as-built) | 313 | 1.638297 | 6 | 1209.88 | 4.5154 |
| Horizontal with machined surface (as-built) | 314 | 1.638615 | 6 | 1210.36 | 4.5375 |
| Horizontal with machined surface (as-built) | 315 | 1.638931 | 6 | 1210.86 | 4.5597 |
| Horizontal with machined surface (as-built) | 316 | 1.639246 | 6 | 1211.34 | 4.5821 |
| Horizontal with machined surface (as-built) | 317 | 1.639558 | 6 | 1211.84 | 4.6047 |
| Horizontal with machined surface (as-built) | 318 | 1.639868 | 6 | 1212.32 | 4.6274 |
| Horizontal with machined surface (as-built) | 319 | 1.640175 | 6 | 1212.82 | 4.6504 |
| Horizontal with machined surface (as-built) | 320 | 1.640479 | 6 | 1213.3 | 4.6735 |
| Horizontal with machined surface (as-built) | 321 | 1.640781 | 6 | 1213.8 | 4.6968 |
| Horizontal with machined surface (as-built) | 322 | 1.64108 | 6 | 1214.28 | 4.7203 |
| Horizontal with machined surface (as-built) | 323 | 1.641375 | 6 | 1214.78 | 4.744 |
| Horizontal with machined surface (as-built) | 324 | 1.641667 | 6 | 1215.26 | 4.7679 |
| Horizontal with machined surface (as-built) | 325 | 1.641956 | 6 | 1215.76 | 4.7919 |
| Horizontal with machined surface (as-built) | 326 | 1.642241 | 6 | 1216.24 | 4.816 |
| Horizontal with machined surface (as-built) | 327 | 1.642522 | 6 | 1216.74 | 4.8403 |
| Horizontal with machined surface (as-built) | 328 | 1.642799 | 6 | 1217.22 | 4.8646 |
| Horizontal with machined surface (as-built) | 329 | 1.643072 | 6 | 1217.7 | 4.8889 |
| Horizontal with machined surface (as-built) | 330 | 1.643341 | 6 | 1218.18 | 4.9133 |
| Horizontal with machined surface (as-built) | 331 | 1.643605 | 6 | 1218.66 | 4.9377 |
| Horizontal with machined surface (as-built) | 332 | 1.643865 | 6 | 1219.14 | 4.9621 |
| Horizontal with machined surface (as-built) | 333 | 1.644121 | 6 | 1219.6 | 4.9864 |
| Horizontal with machined surface (as-built) | 334 | 1.644373 | 6 | 1220.08 | 5.0108 |
| Horizontal with machined surface (as-built) | 335 | 1.644621 | 6 | 1220.54 | 5.0351 |
| Horizontal with machined surface (as-built) | 336 | 1.644865 | 6 | 1221 | 5.0593 |
| Horizontal with machined surface (as-built) | 337 | 1.645104 | 6 | 1221.46 | 5.0836 |
| Horizontal with machined surface (as-built) | 338 | 1.64534 | 6 | 1221.92 | 5.1078 |
| Horizontal with machined surface (as-built) | 339 | 1.645572 | 6 | 1222.36 | 5.1321 |
| Horizontal with machined surface (as-built) | 340 | 1.645801 | 6 | 1222.82 | 5.1563 |
| Horizontal with machined surface (as-built) | 341 | 1.646026 | 6 | 1223.26 | 5.1806 |
| Horizontal with machined surface (as-built) | 342 | 1.646248 | 6 | 1223.72 | 5.2048 |
| Horizontal with machined surface (as-built) | 343 | 1.646466 | 6 | 1224.16 | 5.2292 |
| Horizontal with machined surface (as-built) | 344 | 1.646681 | 6 | 1224.6 | 5.2535 |
| Horizontal with machined surface (as-built) | 345 | 1.646892 | 6 | 1225.04 | 5.2779 |
| Horizontal with machined surface (as-built) | 346 | 1.647097 | 6 | 1225.48 | 5.3023 |
| Horizontal with machined surface (as-built) | 347 | 1.647299 | 6 | 1225.9 | 5.3267 |
| Horizontal with machined surface (as-built) | 348 | 1.647496 | 6 | 1226.34 | 5.3512 |
| Horizontal with machined surface (as-built) | 349 | 1.647689 | 6 | 1226.76 | 5.3757 |
| Horizontal with machined surface (as-built) | 350 | 1.647878 | 6 | 1227.2 | 5.4001 |
| Horizontal with machined surface (as-built) | 351 | 1.648063 | 6 | 1227.62 | 5.4246 |
| Horizontal with machined surface (as-built) | 352 | 1.648243 | 6 | 1228.04 | 5.449 |
| Horizontal with machined surface (as-built) | 353 | 1.648417 | 6 | 1228.44 | 5.4734 |
| Horizontal with machined surface (as-built) | 354 | 1.648585 | 6 | 1228.86 | 5.4978 |
| Horizontal with machined surface (as-built) | 355 | 1.648747 | 6 | 1229.26 | 5.5221 |
| Horizontal with machined surface (as-built) | 356 | 1.648903 | 6 | 1229.66 | 5.5463 |
| Horizontal with machined surface (as-built) | 357 | 1.649052 | 6 | 1230.06 | 5.5705 |
| Horizontal with machined surface (as-built) | 358 | 1.649197 | 6 | 1230.44 | 5.5947 |
| Horizontal with machined surface (as-built) | 359 | 1.649337 | 6 | 1230.82 | 5.6188 |
| Horizontal with machined surface (as-built) | 360 | 1.649474 | 6 | 1231.2 | 5.6429 |
| Horizontal with machined surface (as-built) | 361 | 1.649607 | 6 | 1231.6 | 5.667 |
| Horizontal with machined surface (as-built) | 362 | 1.649739 | 6 | 1231.96 | 5.6911 |
| Horizontal with machined surface (as-built) | 363 | 1.649869 | 6 | 1232.34 | 5.7151 |
| Horizontal with machined surface (as-built) | 364 | 1.649998 | 6 | 1232.72 | 5.7392 |
| Horizontal with machined surface (as-built) | 365 | 1.650127 | 6 | 1233.1 | 5.7634 |
| Horizontal with machined surface (as-built) | 366 | 1.650257 | 6 | 1233.48 | 5.7876 |
| Horizontal with machined surface (as-built) | 367 | 1.650388 | 6 | 1233.86 | 5.8118 |
| Horizontal with machined surface (as-built) | 368 | 1.65052 | 6 | 1234.24 | 5.8361 |
| Horizontal with machined surface (as-built) | 369 | 1.650653 | 6 | 1234.64 | 5.8605 |
| Horizontal with machined surface (as-built) | 370 | 1.650787 | 6 | 1235.02 | 5.8849 |
| Horizontal with machined surface (as-built) | 371 | 1.650923 | 6 | 1235.4 | 5.9095 |
| Horizontal with machined surface (as-built) | 372 | 1.651061 | 6 | 1235.8 | 5.9341 |
| Horizontal with machined surface (as-built) | 373 | 1.6512 | 6 | 1236.2 | 5.9589 |
| Horizontal with machined surface (as-built) | 374 | 1.651341 | 6 | 1236.6 | 5.9838 |
| Horizontal with machined surface (as-built) | 375 | 1.651485 | 6 | 1237 | 6.0089 |
| Horizontal with machined surface (as-built) | 376 | 1.65163 | 6 | 1237.4 | 6.0341 |
| Horizontal with machined surface (as-built) | 377 | 1.651775 | 6 | 1237.8 | 6.0594 |
| Horizontal with machined surface (as-built) | 378 | 1.65192 | 6 | 1238.22 | 6.0849 |
| Horizontal with machined surface (as-built) | 379 | 1.652064 | 6 | 1238.62 | 6.1105 |
| Horizontal with machined surface (as-built) | 380 | 1.652207 | 6 | 1239.04 | 6.1363 |
| Horizontal with machined surface (as-built) | 381 | 1.652349 | 6 | 1239.44 | 6.1623 |
| Horizontal with machined surface (as-built) | 382 | 1.652488 | 6 | 1239.86 | 6.1883 |
| Horizontal with machined surface (as-built) | 383 | 1.652625 | 6 | 1240.26 | 6.2146 |
| Horizontal with machined surface (as-built) | 384 | 1.65276 | 6 | 1240.68 | 6.2409 |
| Horizontal with machined surface (as-built) | 385 | 1.652892 | 6 | 1241.08 | 6.2674 |
| Horizontal with machined surface (as-built) | 386 | 1.653023 | 6 | 1241.5 | 6.294 |
| Horizontal with machined surface (as-built) | 387 | 1.65315 | 6 | 1241.9 | 6.3207 |
| Horizontal with machined surface (as-built) | 388 | 1.653275 | 6 | 1242.32 | 6.3475 |
| Horizontal with machined surface (as-built) | 389 | 1.653397 | 6 | 1242.72 | 6.3744 |
| Horizontal with machined surface (as-built) | 390 | 1.653515 | 6 | 1243.12 | 6.4014 |
| Horizontal with machined surface (as-built) | 391 | 1.653628 | 6 | 1243.54 | 6.4285 |
| Horizontal with machined surface (as-built) | 392 | 1.653737 | 6 | 1243.94 | 6.4558 |
| Horizontal with machined surface (as-built) | 393 | 1.65384 | 6 | 1244.34 | 6.4831 |
| Horizontal with machined surface (as-built) | 394 | 1.653938 | 6 | 1244.74 | 6.5106 |
| Horizontal with machined surface (as-built) | 395 | 1.654029 | 6 | 1245.12 | 6.5381 |
| Horizontal with machined surface (as-built) | 396 | 1.654114 | 6 | 1245.52 | 6.5658 |
| Horizontal with machined surface (as-built) | 397 | 1.654192 | 6 | 1245.9 | 6.5935 |
| Horizontal with machined surface (as-built) | 398 | 1.654263 | 6 | 1246.28 | 6.6213 |
| Horizontal with machined surface (as-built) | 399 | 1.654327 | 6 | 1246.66 | 6.6491 |
| Horizontal with machined surface (as-built) | 400 | 1.654385 | 6 | 1247.02 | 6.677 |
| Horizontal with machined surface (as-built) | 401 | 1.654437 | 6 | 1247.4 | 6.7049 |
| Horizontal with machined surface (as-built) | 402 | 1.654482 | 6 | 1247.76 | 6.7328 |
| Horizontal with machined surface (as-built) | 403 | 1.65452 | 6 | 1248.12 | 6.7608 |
| Horizontal with machined surface (as-built) | 404 | 1.654552 | 6 | 1248.48 | 6.7888 |
| Horizontal with machined surface (as-built) | 405 | 1.654577 | 6 | 1248.82 | 6.8169 |
| Horizontal with machined surface (as-built) | 406 | 1.654597 | 6 | 1249.18 | 6.8451 |
| Horizontal with machined surface (as-built) | 407 | 1.654612 | 6 | 1249.52 | 6.8735 |
| Horizontal with machined surface (as-built) | 408 | 1.654624 | 6 | 1249.86 | 6.902 |
| Horizontal with machined surface (as-built) | 409 | 1.654633 | 6 | 1250.22 | 6.9306 |
| Horizontal with machined surface (as-built) | 410 | 1.654641 | 6 | 1250.56 | 6.9595 |
| Horizontal with machined surface (as-built) | 411 | 1.654648 | 6 | 1250.9 | 6.9886 |
| Horizontal with machined surface (as-built) | 412 | 1.654654 | 6 | 1251.26 | 7.018 |
| Horizontal with machined surface (as-built) | 413 | 1.654658 | 6 | 1251.62 | 7.0476 |
| Horizontal with machined surface (as-built) | 414 | 1.654661 | 6 | 1251.98 | 7.0776 |
| Horizontal with machined surface (as-built) | 415 | **1.654661** | 6 | **1252.32** | **7.1079** |
| Horizontal with machined surface (as-built) | 416 | 1.65466 | 6 | 1252.7 | 7.1385 |
| Horizontal with machined surface (as-built) | 417 | 1.654656 | 6 | 1253.06 | 7.1696 |
| Horizontal with machined surface (as-built) | 418 | 1.65465 | 6 | 1253.42 | 7.2011 |
| Horizontal with machined surface (as-built) | 419 | 1.654641 | 6 | 1253.8 | 7.233 |
| Horizontal with machined surface (as-built) | 420 | 1.654631 | 6 | 1254.18 | 7.2653 |
| Horizontal with machined surface (as-built) | 421 | 1.654619 | 6 | 1254.56 | 7.2981 |
| Horizontal with machined surface (as-built) | 422 | 1.654604 | 6 | 1254.94 | 7.3314 |
| Horizontal with machined surface (as-built) | 423 | 1.654589 | 6 | 1255.32 | 7.3651 |
| Horizontal with machined surface (as-built) | 424 | 1.654572 | 6 | 1255.72 | 7.3992 |
| Horizontal with machined surface (as-built) | 425 | 1.654553 | 6 | 1256.12 | 7.4338 |
| Horizontal with machined surface (as-built) | 426 | 1.654532 | 6 | 1256.52 | 7.4689 |
| Horizontal with machined surface (as-built) | 427 | 1.654509 | 6 | 1256.92 | 7.5044 |
| Horizontal with machined surface (as-built) | 428 | 1.654485 | 6 | 1257.34 | 7.5403 |
| Horizontal with machined surface (as-built) | 429 | 1.654458 | 6 | 1257.74 | 7.5767 |
| Horizontal with machined surface (as-built) | 430 | 1.65443 | 6 | 1258.16 | 7.6134 |
| Horizontal with machined surface (as-built) | 431 | 1.6544 | 6 | 1258.58 | 7.6506 |
| Horizontal with machined surface (as-built) | 432 | 1.654369 | 6 | 1259 | 7.6881 |
| Horizontal with machined surface (as-built) | 433 | 1.654336 | 6 | 1259.42 | 7.7261 |
| Horizontal with machined surface (as-built) | 434 | 1.6543 | 6 | 1259.86 | 7.7643 |
| Horizontal with machined surface (as-built) | 435 | 1.654262 | 6 | 1260.3 | 7.803 |
| Horizontal with machined surface (as-built) | 436 | 1.654221 | 6 | 1260.72 | 7.842 |
| Horizontal with machined surface (as-built) | 437 | 1.654176 | 6 | 1261.16 | 7.8813 |
| Horizontal with machined surface (as-built) | 438 | 1.654126 | 6 | 1261.6 | 7.921 |
| Horizontal with machined surface (as-built) | 439 | 1.654072 | 6 | 1262.04 | 7.961 |
| Horizontal with machined surface (as-built) | 440 | 1.654013 | 6 | 1262.46 | 8.0013 |
| Horizontal with machined surface (as-built) | 441 | 1.653951 | 6 | 1262.9 | 8.0419 |
| Horizontal with machined surface (as-built) | 442 | 1.653884 | 6 | 1263.34 | 8.0828 |
| Horizontal with machined surface (as-built) | 443 | 1.653814 | 6 | 1263.78 | 8.124 |
| Horizontal with machined surface (as-built) | 444 | 1.65374 | 6 | 1264.22 | 8.1655 |
| Horizontal with machined surface (as-built) | 445 | 1.653663 | 6 | 1264.66 | 8.2072 |
| Horizontal with machined surface (as-built) | 446 | 1.653581 | 6 | 1265.1 | 8.2493 |
| Horizontal with machined surface (as-built) | 447 | 1.653493 | 6 | 1265.54 | 8.2916 |
| Horizontal with machined surface (as-built) | 448 | 1.6534 | 6 | 1265.98 | 8.3342 |
| Horizontal with machined surface (as-built) | 449 | 1.653301 | 6 | 1266.42 | 8.3772 |
| Horizontal with machined surface (as-built) | 450 | 1.653195 | 6 | 1266.86 | 8.4205 |
| Horizontal with machined surface (as-built) | 451 | 1.653083 | 6 | 1267.3 | 8.4641 |
| Horizontal with machined surface (as-built) | 452 | 1.652963 | 6 | 1267.74 | 8.5081 |
| Horizontal with machined surface (as-built) | 453 | 1.652837 | 6 | 1268.16 | 8.5525 |
| Horizontal with machined surface (as-built) | 454 | 1.652703 | 6 | 1268.6 | 8.5973 |
| Horizontal with machined surface (as-built) | 455 | 1.652562 | 6 | 1269.04 | 8.6426 |
| Horizontal with machined surface (as-built) | 456 | 1.652413 | 6 | 1269.48 | 8.6883 |
| Horizontal with machined surface (as-built) | 457 | 1.652255 | 6 | 1269.9 | 8.7345 |
| Horizontal with machined surface (as-built) | 458 | 1.65209 | 6 | 1270.34 | 8.7813 |
| Horizontal with machined surface (as-built) | 459 | 1.651916 | 6 | 1270.78 | 8.8287 |
| Horizontal with machined surface (as-built) | 460 | 1.651734 | 6 | 1271.22 | 8.8766 |
| Horizontal with machined surface (as-built) | 461 | 1.651543 | 6 | 1271.64 | 8.9251 |
| Horizontal with machined surface (as-built) | 462 | 1.651342 | 6 | 1272.08 | 8.9742 |
| Horizontal with machined surface (as-built) | 463 | 1.651133 | 6 | 1272.52 | 9.024 |
| Horizontal with machined surface (as-built) | 464 | 1.650915 | 6 | 1272.96 | 9.0745 |
| Horizontal with machined surface (as-built) | 465 | 1.650689 | 6 | 1273.4 | 9.1256 |
| Horizontal with machined surface (as-built) | 466 | 1.650455 | 6 | 1273.84 | 9.1775 |
| Horizontal with machined surface (as-built) | 467 | 1.650213 | 6 | 1274.3 | 9.2302 |
| Horizontal with machined surface (as-built) | 468 | 1.649964 | 6 | 1274.74 | 9.2836 |
| Horizontal with machined surface (as-built) | 469 | 1.649707 | 6 | 1275.2 | 9.3377 |
| Horizontal with machined surface (as-built) | 470 | 1.649443 | 6 | 1275.66 | 9.3927 |
| Horizontal with machined surface (as-built) | 471 | 1.649172 | 6 | 1276.12 | 9.4485 |
| Horizontal with machined surface (as-built) | 472 | 1.648891 | 6 | 1276.6 | 9.5052 |
| Horizontal with machined surface (as-built) | 473 | 1.648601 | 6 | 1277.06 | 9.5627 |
| Horizontal with machined surface (as-built) | 474 | 1.648302 | 6 | 1277.54 | 9.6211 |
| Horizontal with machined surface (as-built) | 475 | 1.647992 | 6 | 1278.02 | 9.6803 |
| Horizontal with machined surface (as-built) | 476 | 1.647672 | 6 | 1278.48 | 9.7404 |
| Horizontal with machined surface (as-built) | 477 | 1.647341 | 6 | 1278.96 | 9.8014 |
| Horizontal with machined surface (as-built) | 478 | 1.646998 | 6 | 1279.46 | 9.8633 |
| Horizontal with machined surface (as-built) | 479 | 1.646644 | 6 | 1279.94 | 9.9261 |
| Horizontal with machined surface (as-built) | 480 | 1.646277 | 6 | 1280.42 | 9.9899 |
| Horizontal with machined surface (as-built) | 481 | 1.645896 | 6 | 1280.92 | 10.0546 |
| Horizontal with machined surface (as-built) | 482 | 1.645502 | 6 | 1281.4 | 10.1203 |
| Horizontal with machined surface (as-built) | 483 | 1.645092 | 6 | 1281.88 | 10.187 |
| Horizontal with machined surface (as-built) | 484 | 1.644665 | 6 | 1282.38 | 10.2547 |
| Horizontal with machined surface (as-built) | 485 | 1.644221 | 6 | 1282.86 | 10.3234 |
| Horizontal with machined surface (as-built) | 486 | 1.643759 | 6 | 1283.34 | 10.3932 |
| Horizontal with machined surface (as-built) | 487 | 1.64328 | 6 | 1283.84 | 10.4641 |
| Horizontal with machined surface (as-built) | 488 | 1.642783 | 6 | 1284.32 | 10.5361 |
| Horizontal with machined surface (as-built) | 489 | 1.642268 | 6 | 1284.8 | 10.6091 |
| Horizontal with machined surface (as-built) | 490 | 1.641735 | 6 | 1285.28 | 10.6832 |
| Horizontal with machined surface (as-built) | 491 | 1.641184 | 6 | 1285.76 | 10.7584 |
| Horizontal with machined surface (as-built) | 492 | 1.640616 | 6 | 1286.24 | 10.8347 |
| Horizontal with machined surface (as-built) | 493 | 1.640031 | 6 | 1286.72 | 10.9121 |
| Horizontal with machined surface (as-built) | 494 | 1.639428 | 6 | 1287.22 | 10.9905 |
| Horizontal with machined surface (as-built) | 495 | 1.638808 | 6 | 1287.7 | 11.0701 |
| Horizontal with machined surface (as-built) | 496 | 1.63817 | 6 | 1288.18 | 11.1507 |
| Horizontal with machined surface (as-built) | 497 | 1.637516 | 6 | 1288.64 | 11.2324 |
| Horizontal with machined surface (as-built) | 498 | 1.636843 | 6 | 1289.12 | 11.3151 |
| Horizontal with machined surface (as-built) | 499 | 1.636154 | 6 | 1289.6 | 11.3989 |
| Horizontal with machined surface (as-built) | 500 | 1.635447 | 6 | 1290.08 | 11.4838 |
| Horizontal with machined surface (as-built) | 501 | 1.634724 | 6 | 1290.56 | 11.5696 |
| Horizontal with machined surface (as-built) | 502 | 1.633984 | 6 | 1291.04 | 11.6565 |
| Horizontal with machined surface (as-built) | 503 | 1.633229 | 6 | 1291.5 | 11.7443 |
| Horizontal with machined surface (as-built) | 504 | 1.632459 | 6 | 1291.98 | 11.8332 |
| Horizontal with machined surface (as-built) | 505 | 1.631674 | 6 | 1292.46 | 11.9229 |
| Horizontal with machined surface (as-built) | 506 | 1.630875 | 6 | 1292.92 | 12.0136 |
| Horizontal with machined surface (as-built) | 507 | 1.630061 | 6 | 1293.4 | 12.1052 |
| Horizontal with machined surface (as-built) | 508 | 1.629233 | 6 | 1293.88 | 12.1976 |
| Horizontal with machined surface (as-built) | 509 | 1.628388 | 6 | 1294.34 | 12.2909 |
| Horizontal with machined surface (as-built) | 510 | 1.627528 | 6 | 1294.8 | 12.3851 |
| Horizontal with machined surface (as-built) | 511 | 1.626652 | 6 | 1295.28 | 12.4802 |
| Horizontal with machined surface (as-built) | 512 | 1.625758 | 6 | 1295.74 | 12.5761 |
| Horizontal with machined surface (as-built) | 513 | 1.624848 | 6 | 1296.18 | 12.6729 |
| Horizontal with machined surface (as-built) | 514 | 1.62392 | 6 | 1296.64 | 12.7705 |
| Horizontal with machined surface (as-built) | 515 | 1.622975 | 6 | 1297.1 | 12.869 |
| Horizontal with machined surface (as-built) | 516 | 1.622013 | 6 | 1297.54 | 12.9684 |
| Horizontal with machined surface (as-built) | 517 | 1.621034 | 6 | 1297.98 | 13.0687 |
| Horizontal with machined surface (as-built) | 518 | 1.62004 | 6 | 1298.42 | 13.1698 |
| Horizontal with machined surface (as-built) | 519 | 1.619028 | 6 | 1298.86 | 13.2719 |
| Horizontal with machined surface (as-built) | 520 | 1.618001 | 6 | 1299.3 | 13.375 |
| Horizontal with machined surface (as-built) | 521 | 1.616958 | 6 | 1299.74 | 13.4789 |
| Horizontal with machined surface (as-built) | 522 | 1.615899 | 6 | 1300.18 | 13.5838 |
| Horizontal with machined surface (as-built) | 523 | 1.614824 | 6 | 1300.62 | 13.6896 |
| Horizontal with machined surface (as-built) | 524 | 1.613734 | 6 | 1301.04 | 13.7963 |
| Horizontal with machined surface (as-built) | 525 | 1.612629 | 6 | 1301.48 | 13.9039 |
| Horizontal with machined surface (as-built) | 526 | 1.611511 | 6 | 1301.9 | 14.0125 |
| Horizontal with machined surface (as-built) | 527 | 1.610379 | 6 | 1302.34 | 14.122 |
| Horizontal with machined surface (as-built) | 528 | 1.609234 | 6 | 1302.76 | 14.2324 |
| Horizontal with machined surface (as-built) | 529 | 1.608076 | 6 | 1303.2 | 14.3437 |
| Horizontal with machined surface (as-built) | 530 | 1.606905 | 6 | 1303.62 | 14.4559 |
| Horizontal with machined surface (as-built) | 531 | 1.605721 | 6 | 1304.06 | 14.5689 |
| Horizontal with machined surface (as-built) | 532 | 1.604524 | 6 | 1304.48 | 14.6829 |
| Horizontal with machined surface (as-built) | 533 | 1.603315 | 6 | 1304.92 | 14.7977 |
| Horizontal with machined surface (as-built) | 534 | 1.602093 | 6 | 1305.34 | 14.9134 |
| Horizontal with machined surface (as-built) | 535 | 1.600859 | 6 | 1305.78 | 15.0299 |
| Horizontal with machined surface (as-built) | 536 | 1.599614 | 6 | 1306.2 | 15.1472 |
| Horizontal with machined surface (as-built) | 537 | 1.598358 | 6 | 1306.64 | 15.2654 |
| Horizontal with machined surface (as-built) | 538 | 1.597093 | 6 | 1307.06 | 15.3844 |
| Horizontal with machined surface (as-built) | 539 | 1.595819 | 6 | 1307.5 | 15.5042 |
| Horizontal with machined surface (as-built) | 540 | 1.594537 | 6 | 1307.94 | 15.6248 |
| Horizontal with machined surface (as-built) | 541 | 1.593248 | 6 | 1308.38 | 15.7463 |
| Horizontal with machined surface (as-built) | 542 | 1.591953 | 6 | 1308.82 | 15.8686 |
| Horizontal with machined surface (as-built) | 543 | 1.590653 | 6 | 1309.28 | 15.9917 |
| Horizontal with machined surface (as-built) | 544 | 1.589346 | 6 | 1309.74 | 16.1157 |
| Horizontal with machined surface (as-built) | 545 | 1.588035 | 6 | 1310.2 | 16.2405 |
| Horizontal with machined surface (as-built) | 546 | 1.58672 | 6 | 1310.66 | 16.3662 |
| Horizontal with machined surface (as-built) | 547 | 1.5854 | 6 | 1311.14 | 16.4927 |
| Horizontal with machined surface (as-built) | 548 | 1.584076 | 6 | 1311.62 | 16.6201 |
| Horizontal with machined surface (as-built) | 549 | 1.582748 | 6 | 1312.1 | 16.7484 |
| Horizontal with machined surface (as-built) | 550 | 1.581417 | 6 | 1312.6 | 16.8775 |
| Horizontal with machined surface (as-built) | 551 | 1.580084 | 6 | 1313.1 | 17.0075 |
| Horizontal with machined surface (as-built) | 552 | 1.578749 | 6 | 1313.62 | 17.1385 |
| Horizontal with machined surface (as-built) | 553 | 1.577412 | 6 | 1314.14 | 17.2704 |
| Horizontal with machined surface (as-built) | 554 | 1.576071 | 6 | 1314.66 | 17.4032 |
| Horizontal with machined surface (as-built) | 555 | 1.574725 | 6 | 1315.2 | 17.537 |
| Horizontal with machined surface (as-built) | 556 | 1.573375 | 6 | 1315.74 | 17.6718 |
| Horizontal with machined surface (as-built) | 557 | 1.572018 | 6 | 1316.3 | 17.8077 |
| Horizontal with machined surface (as-built) | 558 | 1.570655 | 6 | 1316.86 | 17.9445 |
| Horizontal with machined surface (as-built) | 559 | 1.569284 | 6 | 1317.42 | 18.0824 |
| Horizontal with machined surface (as-built) | 560 | 1.567906 | 6 | 1318 | 18.2214 |
| Horizontal with machined surface (as-built) | 561 | 1.566521 | 6 | 1318.56 | 18.3614 |
| Horizontal with machined surface (as-built) | 562 | 1.565128 | 6 | 1319.14 | 18.5025 |
| Horizontal with machined surface (as-built) | 563 | 1.563727 | 6 | 1319.74 | 18.6447 |
| Horizontal with machined surface (as-built) | 564 | 1.562318 | 6 | 1320.32 | 18.7879 |
| Horizontal with machined surface (as-built) | 565 | 1.560901 | 6 | 1320.92 | 18.9322 |
| Horizontal with machined surface (as-built) | 566 | 1.559477 | 6 | 1321.54 | 19.0776 |
| Horizontal with machined surface (as-built) | 567 | 1.558045 | 6 | 1322.14 | 19.224 |
| Horizontal with machined surface (as-built) | 568 | 1.556606 | 6 | 1322.76 | 19.3714 |
| Horizontal with machined surface (as-built) | 569 | 1.55516 | 6 | 1323.38 | 19.5199 |
| Horizontal with machined surface (as-built) | 570 | 1.553707 | 6 | 1324 | 19.6693 |
| Horizontal with machined surface (as-built) | 571 | 1.552248 | 6 | 1324.64 | 19.8197 |
| Horizontal with machined surface (as-built) | 572 | 1.550783 | 6 | 1325.28 | 19.9711 |
| Horizontal with machined surface (as-built) | 573 | 1.549311 | 6 | 1325.92 | 20.1234 |
| Horizontal with machined surface (as-built) | 574 | 1.547831 | 6 | 1326.56 | 20.2766 |
| Horizontal with machined surface (as-built) | 575 | 1.546344 | 6 | 1327.22 | 20.4307 |
| Horizontal with machined surface (as-built) | 576 | 1.544848 | 6 | 1327.88 | 20.5856 |
| Horizontal with machined surface (as-built) | 577 | 1.543343 | 6 | 1328.52 | 20.7413 |
| Horizontal with machined surface (as-built) | 578 | 1.54183 | 6 | 1329.18 | 20.8978 |
| Horizontal with machined surface (as-built) | 579 | 1.540308 | 6 | 1329.84 | 21.0551 |
| Horizontal with machined surface (as-built) | 580 | 1.538778 | 6 | 1330.5 | 21.2132 |
| Horizontal with machined surface (as-built) | 581 | 1.537239 | 6 | 1331.16 | 21.372 |
| Horizontal with machined surface (as-built) | 582 | 1.535691 | 6 | 1331.82 | 21.5315 |
| Horizontal with machined surface (as-built) | 583 | 1.534134 | 6 | 1332.48 | 21.6918 |
| Horizontal with machined surface (as-built) | 584 | 1.53257 | 6 | 1333.14 | 21.8527 |
| Horizontal with machined surface (as-built) | 585 | 1.530999 | 6 | 1333.8 | 22.0144 |
| Horizontal with machined surface (as-built) | 586 | 1.529422 | 6 | 1334.46 | 22.1767 |
| Horizontal with machined surface (as-built) | 587 | 1.527839 | 6 | 1335.12 | 22.3396 |
| Horizontal with machined surface (as-built) | 588 | 1.526253 | 6 | 1335.8 | 22.5031 |
| Horizontal with machined surface (as-built) | 589 | 1.524662 | 6 | 1336.46 | 22.6672 |
| Horizontal with machined surface (as-built) | 590 | 1.523067 | 6 | 1337.14 | 22.8319 |
| Horizontal with machined surface (as-built) | 591 | 1.521467 | 6 | 1337.82 | 22.997 |
| Horizontal with machined surface (as-built) | 592 | 1.519864 | 6 | 1338.5 | 23.1627 |
| Horizontal with machined surface (as-built) | 593 | 1.518257 | 6 | 1339.16 | 23.3287 |
| Horizontal with machined surface (as-built) | 594 | 1.516646 | 6 | 1339.84 | 23.4952 |
| Horizontal with machined surface (as-built) | 595 | 1.515031 | 6 | 1340.52 | 23.6621 |
| Horizontal with machined surface (as-built) | 596 | 1.513414 | 6 | 1341.2 | 23.8294 |
| Horizontal with machined surface (as-built) | 597 | 1.511795 | 6 | 1341.88 | 23.997 |
| Horizontal with machined surface (as-built) | 598 | 1.510174 | 6 | 1342.58 | 24.165 |
| Horizontal with machined surface (as-built) | 599 | 1.508551 | 6 | 1343.26 | 24.3334 |
| Horizontal with machined surface (as-built) | 600 | 1.506926 | 6 | 1343.94 | 24.5021 |
| Horizontal with machined surface (as-built) | 601 | 1.505301 | 6 | 1344.64 | 24.6712 |
| Horizontal with machined surface (as-built) | 602 | 1.503674 | 6 | 1345.32 | 24.8407 |
| Horizontal with machined surface (as-built) | 603 | 1.502047 | 6 | 1346.02 | 25.0107 |
| Horizontal with machined surface (as-built) | 604 | 1.500421 | 6 | 1346.72 | 25.1811 |
| Horizontal with machined surface (as-built) | 605 | 1.498794 | 6 | 1347.44 | 25.3521 |
| Horizontal with machined surface (as-built) | 606 | 1.497169 | 6 | 1348.16 | 25.5237 |
| Horizontal with machined surface (as-built) | 607 | 1.495544 | 6 | 1348.88 | 25.6959 |
| Horizontal with machined surface (as-built) | 608 | 1.493919 | 6 | 1349.6 | 25.8687 |
| Horizontal with machined surface (as-built) | 609 | 1.492295 | 6 | 1350.34 | 26.0423 |
| Horizontal with machined surface (as-built) | 610 | 1.490671 | 6 | 1351.1 | 26.2165 |
| Horizontal with machined surface (as-built) | 611 | 1.489046 | 6 | 1351.84 | 26.3915 |
| Horizontal with machined surface (as-built) | 612 | 1.487421 | 6 | 1352.6 | 26.5672 |
| Horizontal with machined surface (as-built) | 613 | 1.485795 | 6 | 1353.38 | 26.7437 |
| Horizontal with machined surface (as-built) | 614 | 1.484168 | 6 | 1354.16 | 26.921 |
| Horizontal with machined surface (as-built) | 615 | 1.482539 | 6 | 1354.94 | 27.099 |
| Horizontal with machined surface (as-built) | 616 | 1.480906 | 6 | 1355.72 | 27.2777 |
| Horizontal with machined surface (as-built) | 617 | 1.479269 | 6 | 1356.52 | 27.4571 |
| Horizontal with machined surface (as-built) | 618 | 1.477628 | 6 | 1357.3 | 27.6372 |
| Horizontal with machined surface (as-built) | 619 | 1.47598 | 6 | 1358.1 | 27.818 |
| Horizontal with machined surface (as-built) | 620 | 1.474327 | 6 | 1358.9 | 27.9994 |
| Horizontal with machined surface (as-built) | 621 | 1.472668 | 6 | 1359.7 | 28.1814 |
| Horizontal with machined surface (as-built) | 622 | 1.471004 | 6 | 1360.5 | 28.364 |
| Horizontal with machined surface (as-built) | 623 | 1.469334 | 6 | 1361.3 | 28.5472 |
| Horizontal with machined surface (as-built) | 624 | 1.467659 | 6 | 1362.1 | 28.7309 |
| Horizontal with machined surface (as-built) | 625 | 1.465979 | 6 | 1362.9 | 28.9151 |
| Horizontal with machined surface (as-built) | 626 | 1.464294 | 6 | 1363.7 | 29.0998 |
| Horizontal with machined surface (as-built) | 627 | 1.462605 | 6 | 1364.5 | 29.285 |
| Horizontal with machined surface (as-built) | 628 | 1.46091 | 6 | 1365.3 | 29.4706 |
| Horizontal with machined surface (as-built) | 629 | 1.459209 | 6 | 1366.1 | 29.6567 |
| Horizontal with machined surface (as-built) | 630 | 1.457504 | 6 | 1366.9 | 29.8431 |
| Horizontal with machined surface (as-built) | 631 | 1.455793 | 6 | 1367.68 | 30.03 |
| Horizontal with machined surface (as-built) | 632 | 1.454076 | 6 | 1368.48 | 30.2172 |
| Horizontal with machined surface (as-built) | 633 | 1.452354 | 6 | 1369.28 | 30.4048 |
| Horizontal with machined surface (as-built) | 634 | 1.450627 | 6 | 1370.06 | 30.5927 |
| Horizontal with machined surface (as-built) | 635 | 1.448893 | 6 | 1370.84 | 30.7811 |
| Horizontal with machined surface (as-built) | 636 | 1.447154 | 6 | 1371.62 | 30.9697 |
| Horizontal with machined surface (as-built) | 637 | 1.44541 | 6 | 1372.42 | 31.1588 |
| Horizontal with machined surface (as-built) | 638 | 1.443659 | 6 | 1373.18 | 31.3482 |
| Horizontal with machined surface (as-built) | 639 | 1.441902 | 6 | 1373.96 | 31.538 |
| Horizontal with machined surface (as-built) | 640 | 1.440138 | 6 | 1374.74 | 31.7281 |
| Horizontal with machined surface (as-built) | 641 | 1.438368 | 6 | 1375.5 | 31.9186 |
| Horizontal with machined surface (as-built) | 642 | 1.436589 | 6 | 1376.26 | 32.1094 |
| Horizontal with machined surface (as-built) | 643 | 1.434803 | 6 | 1377.02 | 32.3006 |
| Horizontal with machined surface (as-built) | 644 | 1.43301 | 6 | 1377.78 | 32.4921 |
| Horizontal with machined surface (as-built) | 645 | 1.431209 | 6 | 1378.52 | 32.6839 |
| Horizontal with machined surface (as-built) | 646 | 1.429402 | 6 | 1379.26 | 32.8761 |
| Horizontal with machined surface (as-built) | 647 | 1.427588 | 6 | 1380.02 | 33.0687 |
| Horizontal with machined surface (as-built) | 648 | 1.425767 | 6 | 1380.74 | 33.2617 |
| Horizontal with machined surface (as-built) | 649 | 1.42394 | 6 | 1381.48 | 33.455 |
| Horizontal with machined surface (as-built) | 650 | 1.422107 | 6 | 1382.22 | 33.6488 |
| Horizontal with machined surface (as-built) | 651 | 1.420266 | 6 | 1382.94 | 33.8432 |
| Horizontal with machined surface (as-built) | 652 | 1.418417 | 6 | 1383.68 | 34.0381 |
| Horizontal with machined surface (as-built) | 653 | 1.416562 | 6 | 1384.4 | 34.2336 |
| Horizontal with machined surface (as-built) | 654 | 1.414698 | 6 | 1385.12 | 34.4298 |
| Horizontal with machined surface (as-built) | 655 | 1.412826 | 6 | 1385.84 | 34.6267 |
| Horizontal with machined surface (as-built) | 656 | 1.410948 | 6 | 1386.58 | 34.8244 |
| Horizontal with machined surface (as-built) | 657 | 1.409062 | 6 | 1387.3 | 35.023 |
| Horizontal with machined surface (as-built) | 658 | 1.40717 | 6 | 1388.04 | 35.2225 |
| Horizontal with machined surface (as-built) | 659 | 1.405271 | 6 | 1388.76 | 35.4229 |
| Horizontal with machined surface (as-built) | 660 | 1.403367 | 6 | 1389.5 | 35.6243 |
| Horizontal with machined surface (as-built) | 661 | 1.401458 | 6 | 1390.26 | 35.8267 |
| Horizontal with machined surface (as-built) | 662 | 1.399545 | 6 | 1391 | 36.0302 |
| Horizontal with machined surface (as-built) | 663 | 1.397628 | 6 | 1391.78 | 36.2347 |
| Horizontal with machined surface (as-built) | 664 | 1.395709 | 6 | 1392.54 | 36.4402 |
| Horizontal with machined surface (as-built) | 665 | 1.393788 | 6 | 1393.32 | 36.6468 |
| Horizontal with machined surface (as-built) | 666 | 1.391868 | 6 | 1394.12 | 36.8545 |
| Horizontal with machined surface (as-built) | 667 | 1.389949 | 6 | 1394.92 | 37.0632 |
| Horizontal with machined surface (as-built) | 668 | 1.388031 | 6 | 1395.74 | 37.273 |
| Horizontal with machined surface (as-built) | 669 | 1.386117 | 6 | 1396.58 | 37.4837 |
| Horizontal with machined surface (as-built) | 670 | 1.384205 | 6 | 1397.42 | 37.6954 |
| Horizontal with machined surface (as-built) | 671 | 1.382296 | 6 | 1398.28 | 37.9081 |
| Horizontal with machined surface (as-built) | 672 | 1.380389 | 6 | 1399.16 | 38.1217 |
| Horizontal with machined surface (as-built) | 673 | 1.378483 | 6 | 1400.04 | 38.3362 |
| Horizontal with machined surface (as-built) | 674 | 1.376578 | 6 | 1400.94 | 38.5517 |
| Horizontal with machined surface (as-built) | 675 | 1.374673 | 6 | 1401.84 | 38.768 |
| Horizontal with machined surface (as-built) | 676 | 1.372767 | 6 | 1402.76 | 38.9853 |
| Horizontal with machined surface (as-built) | 677 | 1.370859 | 6 | 1403.68 | 39.2033 |
| Horizontal with machined surface (as-built) | 678 | 1.368949 | 6 | 1404.6 | 39.4223 |
| Horizontal with machined surface (as-built) | 679 | 1.367038 | 6 | 1405.54 | 39.6421 |
| Horizontal with machined surface (as-built) | 680 | 1.365124 | 6 | 1406.48 | 39.8627 |
| Horizontal with machined surface (as-built) | 681 | 1.363209 | 6 | 1407.42 | 40.0843 |
| Horizontal with machined surface (as-built) | 682 | 1.361291 | 6 | 1408.38 | 40.3067 |
| Horizontal with machined surface (as-built) | 683 | 1.359371 | 6 | 1409.34 | 40.53 |
| Horizontal with machined surface (as-built) | 684 | 1.357448 | 6 | 1410.32 | 40.7543 |
| Horizontal with machined surface (as-built) | 685 | 1.355523 | 6 | 1411.3 | 40.9796 |
| Horizontal with machined surface (as-built) | 686 | 1.353595 | 6 | 1412.28 | 41.2058 |
| Horizontal with machined surface (as-built) | 687 | 1.351665 | 6 | 1413.28 | 41.433 |
| Horizontal with machined surface (as-built) | 688 | 1.349732 | 6 | 1414.28 | 41.6613 |
| Horizontal with machined surface (as-built) | 689 | 1.347798 | 6 | 1415.3 | 41.8906 |
| Horizontal with machined surface (as-built) | 690 | 1.345862 | 6 | 1416.32 | 42.1211 |
| Horizontal with machined surface (as-built) | 691 | 1.343926 | 6 | 1417.36 | 42.3527 |
| Horizontal with machined surface (as-built) | 692 | 1.34199 | 6 | 1418.4 | 42.5854 |
| Horizontal with machined surface (as-built) | 693 | 1.340054 | 6 | 1419.48 | 42.8194 |
| Horizontal with machined surface (as-built) | 694 | 1.338117 | 6 | 1420.54 | 43.0547 |
| Horizontal with machined surface (as-built) | 695 | 1.336177 | 6 | 1421.64 | 43.2913 |
| Horizontal with machined surface (as-built) | 696 | 1.334233 | 6 | 1422.74 | 43.5292 |
| Horizontal with machined surface (as-built) | 697 | 1.332286 | 6 | 1423.86 | 43.7686 |
| Horizontal with machined surface (as-built) | 698 | 1.330334 | 6 | 1424.98 | 44.0094 |
| Horizontal with machined surface (as-built) | 699 | 1.328377 | 6 | 1426.12 | 44.2515 |
| Horizontal with machined surface (as-built) | 700 | 1.326415 | 6 | 1427.28 | 44.4951 |
| Horizontal with machined surface (as-built) | 701 | 1.324447 | 6 | 1428.44 | 44.7401 |
| Horizontal with machined surface (as-built) | 702 | 1.322472 | 6 | 1429.6 | 44.9865 |
| Horizontal with machined surface (as-built) | 703 | 1.320491 | 6 | 1430.78 | 45.2343 |
| Horizontal with machined surface (as-built) | 704 | 1.318503 | 6 | 1431.96 | 45.4834 |
| Horizontal with machined surface (as-built) | 705 | 1.316508 | 6 | 1433.16 | 45.7338 |
| Horizontal with machined surface (as-built) | 706 | 1.314507 | 6 | 1434.34 | 45.9855 |
| Horizontal with machined surface (as-built) | 707 | 1.312498 | 6 | 1435.56 | 46.2383 |
| Horizontal with machined surface (as-built) | 708 | 1.310482 | 6 | 1436.76 | 46.4923 |
| Horizontal with machined surface (as-built) | 709 | 1.308458 | 6 | 1437.98 | 46.7474 |
| Horizontal with machined surface (as-built) | 710 | 1.306426 | 6 | 1439.18 | 47.0035 |
| Horizontal with machined surface (as-built) | 711 | 1.304386 | 6 | 1440.4 | 47.2606 |
| Horizontal with machined surface (as-built) | 712 | 1.302338 | 6 | 1441.62 | 47.5186 |
| Horizontal with machined surface (as-built) | 713 | 1.300281 | 6 | 1442.84 | 47.7774 |
| Horizontal with machined surface (as-built) | 714 | 1.298216 | 6 | 1444.04 | 48.0371 |
| Horizontal with machined surface (as-built) | 715 | 1.296144 | 6 | 1445.26 | 48.2977 |
| Horizontal with machined surface (as-built) | 716 | 1.294064 | 6 | 1446.48 | 48.559 |
| Horizontal with machined surface (as-built) | 717 | 1.291978 | 6 | 1447.68 | 48.821 |
| Horizontal with machined surface (as-built) | 718 | 1.289885 | 6 | 1448.9 | 49.0838 |
| Horizontal with machined surface (as-built) | 719 | 1.287786 | 6 | 1450.12 | 49.3474 |
| Horizontal with machined surface (as-built) | 720 | 1.28568 | 6 | 1451.32 | 49.6117 |
| Horizontal with machined surface (as-built) | 721 | 1.283567 | 6 | 1452.54 | 49.8769 |
| Horizontal with machined surface (as-built) | 722 | 1.281447 | 6 | 1453.76 | 50.1429 |
| Horizontal with machined surface (as-built) | 723 | 1.279318 | 6 | 1454.96 | 50.4097 |
| Horizontal with machined surface (as-built) | 724 | 1.277181 | 6 | 1456.18 | 50.6774 |
| Horizontal with machined surface (as-built) | 725 | 1.275037 | 6 | 1457.38 | 50.946 |
| Horizontal with machined surface (as-built) | 726 | 1.272886 | 6 | 1458.6 | 51.2156 |
| Horizontal with machined surface (as-built) | 727 | 1.270728 | 6 | 1459.82 | 51.4862 |
| Horizontal with machined surface (as-built) | 728 | 1.268562 | 6 | 1461.04 | 51.7579 |
| Horizontal with machined surface (as-built) | 729 | 1.26639 | 6 | 1462.26 | 52.0306 |
| Horizontal with machined surface (as-built) | 730 | 1.264211 | 6 | 1463.48 | 52.3045 |
| Horizontal with machined surface (as-built) | 731 | 1.262026 | 6 | 1464.7 | 52.5795 |
| Horizontal with machined surface (as-built) | 732 | 1.259835 | 6 | 1465.94 | 52.8558 |
| Horizontal with machined surface (as-built) | 733 | 1.257638 | 6 | 1467.18 | 53.1332 |
| Horizontal with machined surface (as-built) | 734 | 1.255435 | 6 | 1468.44 | 53.412 |
| Horizontal with machined surface (as-built) | 735 | 1.253227 | 6 | 1469.7 | 53.692 |
| Horizontal with machined surface (as-built) | 736 | 1.251012 | 6 | 1470.96 | 53.9733 |
| Horizontal with machined surface (as-built) | 737 | 1.248792 | 6 | 1472.22 | 54.2558 |
| Horizontal with machined surface (as-built) | 738 | 1.246565 | 6 | 1473.5 | 54.5396 |
| Horizontal with machined surface (as-built) | 739 | 1.244333 | 6 | 1474.78 | 54.8245 |
| Horizontal with machined surface (as-built) | 740 | 1.242095 | 6 | 1476.08 | 55.1107 |
| Horizontal with machined surface (as-built) | 741 | 1.239851 | 6 | 1477.36 | 55.3981 |
| Horizontal with machined surface (as-built) | 742 | 1.237602 | 6 | 1478.66 | 55.6866 |
| Horizontal with machined surface (as-built) | 743 | 1.235348 | 6 | 1479.96 | 55.9763 |
| Horizontal with machined surface (as-built) | 744 | 1.233088 | 6 | 1481.28 | 56.2671 |
| Horizontal with machined surface (as-built) | 745 | 1.230821 | 6 | 1482.58 | 56.5591 |
| Horizontal with machined surface (as-built) | 746 | 1.228547 | 6 | 1483.9 | 56.8523 |
| Horizontal with machined surface (as-built) | 747 | 1.226265 | 6 | 1485.22 | 57.1465 |
| Horizontal with machined surface (as-built) | 748 | 1.223975 | 6 | 1486.52 | 57.4419 |
| Horizontal with machined surface (as-built) | 749 | 1.221675 | 6 | 1487.84 | 57.7384 |
| Horizontal with machined surface (as-built) | 750 | 1.219366 | 6 | 1489.16 | 58.036 |
| Horizontal with machined surface (as-built) | 751 | 1.217048 | 6 | 1490.46 | 58.3347 |
| Horizontal with machined surface (as-built) | 752 | 1.21472 | 6 | 1491.78 | 58.6344 |
| Horizontal with machined surface (as-built) | 753 | 1.212382 | 6 | 1493.08 | 58.9352 |
| Horizontal with machined surface (as-built) | 754 | 1.210034 | 6 | 1494.38 | 59.237 |
| Horizontal with machined surface (as-built) | 755 | 1.207675 | 6 | 1495.68 | 59.5397 |
| Horizontal with machined surface (as-built) | 756 | 1.205304 | 6 | 1496.96 | 59.8434 |
| Horizontal with machined surface (as-built) | 757 | 1.202923 | 6 | 1498.26 | 60.148 |
| Horizontal with machined surface (as-built) | 758 | 1.20053 | 6 | 1499.52 | 60.4535 |
| Horizontal with machined surface (as-built) | 759 | 1.198126 | 6 | 1500.8 | 60.7599 |
| Horizontal with machined surface (as-built) | 760 | 1.195711 | 6 | 1502.06 | 61.0672 |
| Horizontal with machined surface (as-built) | 761 | 1.193286 | 6 | 1503.32 | 61.3754 |
| Horizontal with machined surface (as-built) | 762 | 1.190852 | 6 | 1504.58 | 61.6846 |
| Horizontal with machined surface (as-built) | 763 | 1.188411 | 6 | 1505.82 | 61.9947 |
| Horizontal with machined surface (as-built) | 764 | 1.185962 | 6 | 1507.08 | 62.3058 |
| Horizontal with machined surface (as-built) | 765 | 1.183506 | 6 | 1508.32 | 62.6179 |
| Horizontal with machined surface (as-built) | 766 | 1.181045 | 6 | 1509.58 | 62.931 |
| Horizontal with machined surface (as-built) | 767 | 1.178579 | 6 | 1510.82 | 63.2451 |
| Horizontal with machined surface (as-built) | 768 | 1.176107 | 6 | 1512.08 | 63.5604 |
| Horizontal with machined surface (as-built) | 769 | 1.173629 | 6 | 1513.34 | 63.8769 |
| Horizontal with machined surface (as-built) | 770 | 1.171145 | 6 | 1514.6 | 64.1945 |
| Horizontal with machined surface (as-built) | 771 | 1.168655 | 6 | 1515.88 | 64.5134 |
| Horizontal with machined surface (as-built) | 772 | 1.166159 | 6 | 1517.14 | 64.8337 |
| Horizontal with machined surface (as-built) | 773 | 1.163657 | 6 | 1518.42 | 65.1554 |
| Horizontal with machined surface (as-built) | 774 | 1.161149 | 6 | 1519.72 | 65.4787 |
| Horizontal with machined surface (as-built) | 775 | 1.158636 | 6 | 1521 | 65.8035 |
| Horizontal with machined surface (as-built) | 776 | 1.156118 | 6 | 1522.32 | 66.1299 |
| Horizontal with machined surface (as-built) | 777 | 1.153594 | 6 | 1523.62 | 66.4579 |
| Horizontal with machined surface (as-built) | 778 | 1.151065 | 6 | 1524.96 | 66.7877 |
| Horizontal with machined surface (as-built) | 779 | 1.14853 | 6 | 1526.3 | 67.1191 |
| Horizontal with machined surface (as-built) | 780 | 1.145989 | 6 | 1527.64 | 67.4523 |
| Horizontal with machined surface (as-built) | 781 | 1.143442 | 6 | 1529 | 67.7872 |
| Horizontal with machined surface (as-built) | 782 | 1.140889 | 6 | 1530.36 | 68.1239 |
| Horizontal with machined surface (as-built) | 783 | 1.138328 | 6 | 1531.74 | 68.4623 |
| Horizontal with machined surface (as-built) | 784 | 1.135761 | 6 | 1533.12 | 68.8025 |
| Horizontal with machined surface (as-built) | 785 | 1.133187 | 6 | 1534.52 | 69.1444 |
| Horizontal with machined surface (as-built) | 786 | 1.130609 | 6 | 1535.92 | 69.4882 |
| Horizontal with machined surface (as-built) | 787 | 1.128025 | 6 | 1537.34 | 69.8338 |
| Horizontal with machined surface (as-built) | 788 | 1.125436 | 6 | 1538.78 | 70.1813 |
| Horizontal with machined surface (as-built) | 789 | 1.122842 | 6 | 1540.22 | 70.5306 |
| Horizontal with machined surface (as-built) | 790 | 1.120242 | 6 | 1541.68 | 70.8819 |
| Horizontal with machined surface (as-built) | 791 | 1.117635 | 6 | 1543.14 | 71.2352 |
| Horizontal with machined surface (as-built) | 792 | 1.115021 | 6 | 1544.62 | 71.5904 |
| Horizontal with machined surface (as-built) | 793 | 1.1124 | 6 | 1546.12 | 71.9478 |
| Horizontal with machined surface (as-built) | 794 | 1.109772 | 6 | 1547.62 | 72.3072 |
| Horizontal with machined surface (as-built) | 795 | 1.107135 | 6 | 1549.14 | 72.6689 |
| Horizontal with machined surface (as-built) | 796 | 1.10449 | 6 | 1550.68 | 73.0327 |
| Horizontal with machined surface (as-built) | 797 | 1.101836 | 6 | 1552.22 | 73.3987 |
| Horizontal with machined surface (as-built) | 798 | 1.099174 | 6 | 1553.78 | 73.767 |
| Horizontal with machined surface (as-built) | 799 | 1.096503 | 6 | 1555.34 | 74.1376 |
| Horizontal with machined surface (as-built) | 800 | 1.093824 | 6 | 1556.92 | 74.5105 |
| Horizontal with machined surface (as-built) | 801 | 1.091136 | 6 | 1558.5 | 74.8856 |
| Horizontal with machined surface (as-built) | 802 | 1.08844 | 6 | 1560.12 | 75.2631 |
| Horizontal with machined surface (as-built) | 803 | 1.085735 | 6 | 1561.72 | 75.6428 |
| Horizontal with machined surface (as-built) | 804 | 1.083022 | 6 | 1563.36 | 76.0249 |
| Horizontal with machined surface (as-built) | 805 | 1.080299 | 6 | 1564.98 | 76.4093 |
| Horizontal with machined surface (as-built) | 806 | 1.077566 | 6 | 1566.64 | 76.796 |
| Horizontal with machined surface (as-built) | 807 | 1.074823 | 6 | 1568.28 | 77.1851 |
| Horizontal with machined surface (as-built) | 808 | 1.07207 | 6 | 1569.94 | 77.5765 |
| Horizontal with machined surface (as-built) | 809 | 1.069306 | 6 | 1571.62 | 77.9704 |
| Horizontal with machined surface (as-built) | 810 | 1.06653 | 6 | 1573.3 | 78.3666 |
| Horizontal with machined surface (as-built) | 811 | 1.063742 | 6 | 1574.98 | 78.7652 |
| Horizontal with machined surface (as-built) | 812 | 1.060941 | 6 | 1576.66 | 79.1662 |
| Horizontal with machined surface (as-built) | 813 | 1.058126 | 6 | 1578.34 | 79.5695 |
| Horizontal with machined surface (as-built) | 814 | 1.055295 | 6 | 1580.02 | 79.9752 |
| Horizontal with machined surface (as-built) | 815 | 1.052449 | 6 | 1581.68 | 80.3833 |
| Horizontal with machined surface (as-built) | 816 | 1.049586 | 6 | 1583.36 | 80.7937 |
| Horizontal with machined surface (as-built) | 817 | 1.046706 | 6 | 1585.02 | 81.2064 |
| Horizontal with machined surface (as-built) | 818 | 1.043808 | 6 | 1586.66 | 81.6214 |
| Horizontal with machined surface (as-built) | 819 | 1.04089 | 6 | 1588.3 | 82.0385 |
| Horizontal with machined surface (as-built) | 820 | 1.037951 | 6 | 1589.9 | 82.4579 |
| Horizontal with machined surface (as-built) | 821 | 1.034988 | 6 | 1591.5 | 82.8794 |
| Horizontal with machined surface (as-built) | 822 | 1.032001 | 6 | 1593.06 | 83.3031 |
| Horizontal with machined surface (as-built) | 823 | 1.028986 | 6 | 1594.6 | 83.7289 |
| Horizontal with machined surface (as-built) | 824 | 1.025943 | 6 | 1596.1 | 84.1569 |
| Horizontal with machined surface (as-built) | 825 | 1.022869 | 6 | 1597.56 | 84.587 |
| Horizontal with machined surface (as-built) | 826 | 1.019763 | 6 | 1598.98 | 85.0192 |
| Horizontal with machined surface (as-built) | 827 | 1.016622 | 6 | 1600.36 | 85.4535 |
| Horizontal with machined surface (as-built) | 828 | 1.013444 | 6 | 1601.68 | 85.89 |
| Horizontal with machined surface (as-built) | 829 | 1.010227 | 6 | 1602.94 | 86.3285 |
| Horizontal with machined surface (as-built) | 830 | 1.006968 | 6 | 1604.16 | 86.7692 |
| Horizontal with machined surface (as-built) | 831 | 1.003664 | 6 | 1605.3 | 87.2121 |
| Horizontal with machined surface (as-built) | 832 | 1.000313 | 6 | 1606.38 | 87.6572 |
| Horizontal with machined surface (as-built) | 833 | 0.996913 | 6 | 1607.38 | 88.1045 |
| Horizontal with machined surface (as-built) | 834 | 0.993461 | 6 | 1608.32 | 88.5543 |
| Horizontal with machined surface (as-built) | 835 | 0.989954 | 6 | 1609.16 | 89.0066 |
| Horizontal with machined surface (as-built) | 836 | 0.986391 | 6 | 1609.94 | 89.4614 |
| Horizontal with machined surface (as-built) | 837 | 0.98277 | 6 | 1610.62 | 89.919 |
| Horizontal with machined surface (as-built) | 838 | 0.979088 | 6 | 1611.22 | 90.3792 |
| Horizontal with machined surface (as-built) | 839 | 0.975343 | 6 | 1611.74 | 90.8422 |
| Horizontal with machined surface (as-built) | 840 | 0.971533 | 6 | 1612.16 | 91.3079 |
| Horizontal with machined surface (as-built) | 841 | 0.967659 | 6 | 1612.5 | 91.7763 |
| Horizontal with machined surface (as-built) | 842 | 0.963719 | 6 | 1612.74 | 92.2474 |
| Horizontal with machined surface (as-built) | 843 | 0.959713 | 6 | 1612.86 | 92.7209 |
| Vertical with raw surface (heat-treated) | 1 | 0 | 6 | 0 | 0 |
| Vertical with raw surface (heat-treated) | 2 | 0.013942 | 6 | 9.86 | 0.0031 |
| Vertical with raw surface (heat-treated) | 3 | 0.027916 | 6 | 19.74 | 0.0062 |
| Vertical with raw surface (heat-treated) | 4 | 0.04197 | 6 | 29.68 | 0.0093 |
| Vertical with raw surface (heat-treated) | 5 | 0.056167 | 6 | 39.72 | 0.0124 |
| Vertical with raw surface (heat-treated) | 6 | 0.070575 | 6 | 49.9 | 0.0154 |
| Vertical with raw surface (heat-treated) | 7 | 0.085263 | 6 | 60.28 | 0.0185 |
| Vertical with raw surface (heat-treated) | 8 | 0.100305 | 6 | 70.92 | 0.0217 |
| Vertical with raw surface (heat-treated) | 9 | 0.115773 | 6 | 81.86 | 0.0249 |
| Vertical with raw surface (heat-treated) | 10 | 0.131737 | 6 | 93.16 | 0.0282 |
| Vertical with raw surface (heat-treated) | 11 | 0.148261 | 6 | 104.84 | 0.0317 |
| Vertical with raw surface (heat-treated) | 12 | 0.165391 | 6 | 116.96 | 0.0353 |
| Vertical with raw surface (heat-treated) | 13 | 0.183161 | 6 | 129.54 | 0.0391 |
| Vertical with raw surface (heat-treated) | 14 | 0.201581 | 6 | 142.56 | 0.043 |
| Vertical with raw surface (heat-treated) | 15 | 0.220647 | 6 | 156.06 | 0.0471 |
| Vertical with raw surface (heat-treated) | 16 | 0.240335 | 6 | 170 | 0.0513 |
| Vertical with raw surface (heat-treated) | 17 | 0.260613 | 6 | 184.34 | 0.0557 |
| Vertical with raw surface (heat-treated) | 18 | 0.281436 | 6 | 199.1 | 0.0603 |
| Vertical with raw surface (heat-treated) | 19 | 0.302755 | 6 | 214.2 | 0.0652 |
| Vertical with raw surface (heat-treated) | 20 | 0.324516 | 6 | 229.6 | 0.0704 |
| Vertical with raw surface (heat-treated) | 21 | 0.346663 | 6 | 245.3 | 0.076 |
| Vertical with raw surface (heat-treated) | 22 | 0.36914 | 6 | 261.22 | 0.082 |
| Vertical with raw surface (heat-treated) | 23 | 0.391894 | 6 | 277.36 | 0.0884 |
| Vertical with raw surface (heat-treated) | 24 | 0.414876 | 6 | 293.64 | 0.0953 |
| Vertical with raw surface (heat-treated) | 25 | 0.43804 | 6 | 310.06 | 0.1026 |
| Vertical with raw surface (heat-treated) | 26 | 0.461346 | 6 | 326.6 | 0.1102 |
| Vertical with raw surface (heat-treated) | 27 | 0.484752 | 6 | 343.2 | 0.1183 |
| Vertical with raw surface (heat-treated) | 28 | 0.508223 | 6 | 359.84 | 0.1267 |
| Vertical with raw surface (heat-treated) | 29 | 0.531725 | 6 | 376.52 | 0.1354 |
| Vertical with raw surface (heat-treated) | 30 | 0.555233 | 6 | 393.2 | 0.1445 |
| Vertical with raw surface (heat-treated) | 31 | 0.578725 | 6 | 409.86 | 0.1537 |
| Vertical with raw surface (heat-treated) | 32 | 0.602186 | 6 | 426.52 | 0.163 |
| Vertical with raw surface (heat-treated) | 33 | 0.625607 | 6 | 443.14 | 0.1724 |
| Vertical with raw surface (heat-treated) | 34 | 0.648985 | 6 | 459.74 | 0.1817 |
| Vertical with raw surface (heat-treated) | 35 | 0.672321 | 6 | 476.3 | 0.1909 |
| Vertical with raw surface (heat-treated) | 36 | 0.695618 | 6 | 492.84 | 0.2002 |
| Vertical with raw surface (heat-treated) | 37 | 0.718882 | 6 | 509.36 | 0.2094 |
| Vertical with raw surface (heat-treated) | 38 | 0.74212 | 6 | 525.86 | 0.2187 |
| Vertical with raw surface (heat-treated) | 39 | 0.765337 | 6 | 542.36 | 0.2279 |
| Vertical with raw surface (heat-treated) | 40 | 0.788535 | 6 | 558.84 | 0.2372 |
| Vertical with raw surface (heat-treated) | 41 | 0.811719 | 6 | 575.3 | 0.2465 |
| Vertical with raw surface (heat-treated) | 42 | 0.834892 | 6 | 591.76 | 0.2558 |
| Vertical with raw surface (heat-treated) | 43 | 0.858055 | 6 | 608.22 | 0.2652 |
| Vertical with raw surface (heat-treated) | 44 | 0.881207 | 6 | 624.68 | 0.2746 |
| Vertical with raw surface (heat-treated) | 45 | 0.904349 | 6 | 641.14 | 0.2842 |
| Vertical with raw surface (heat-treated) | 46 | 0.927476 | 6 | 657.58 | 0.2937 |
| Vertical with raw surface (heat-treated) | 47 | 0.950584 | 6 | 674.02 | 0.3032 |
| Vertical with raw surface (heat-treated) | 48 | 0.97367 | 6 | 690.46 | 0.3126 |
| Vertical with raw surface (heat-treated) | 49 | 0.996732 | 6 | 706.86 | 0.3219 |
| Vertical with raw surface (heat-treated) | 50 | 1.019768 | 6 | 723.28 | 0.3311 |
| Vertical with raw surface (heat-treated) | 51 | 1.042778 | 6 | 739.66 | 0.3402 |
| Vertical with raw surface (heat-treated) | 52 | 1.06576 | 6 | 756.04 | 0.349 |
| Vertical with raw surface (heat-treated) | 53 | 1.08871 | 6 | 772.38 | 0.3577 |
| Vertical with raw surface (heat-treated) | 54 | 1.111622 | 6 | 788.72 | 0.3662 |
| Vertical with raw surface (heat-treated) | 55 | 1.134491 | 6 | 805.02 | 0.3745 |
| Vertical with raw surface (heat-treated) | 56 | 1.157318 | 6 | 821.3 | 0.3826 |
| Vertical with raw surface (heat-treated) | 57 | 1.180105 | 6 | 837.56 | 0.3908 |
| Vertical with raw surface (heat-treated) | 58 | 1.20285 | 6 | 853.78 | 0.3989 |
| Vertical with raw surface (heat-treated) | 59 | 1.225554 | 6 | 870 | 0.4071 |
| Vertical with raw surface (heat-treated) | 60 | 1.248215 | 6 | 886.18 | 0.4155 |
| Vertical with raw surface (heat-treated) | 61 | 1.270829 | 6 | 902.32 | 0.424 |
| Vertical with raw surface (heat-treated) | 62 | 1.293394 | 6 | 918.44 | 0.4327 |
| Vertical with raw surface (heat-treated) | 63 | 1.315904 | 6 | 934.54 | 0.4415 |
| Vertical with raw surface (heat-treated) | 64 | 1.338353 | 6 | 950.58 | 0.4505 |
| Vertical with raw surface (heat-treated) | 65 | 1.360738 | 6 | 966.58 | 0.4597 |
| Vertical with raw surface (heat-treated) | 66 | 1.383057 | 6 | 982.54 | 0.469 |
| Vertical with raw surface (heat-treated) | 67 | 1.405309 | 6 | 998.46 | 0.4787 |
| Vertical with raw surface (heat-treated) | 68 | 1.427495 | 6 | 1014.34 | 0.4885 |
| Vertical with raw surface (heat-treated) | 69 | 1.449614 | 6 | 1030.18 | 0.4984 |
| Vertical with raw surface (heat-treated) | 70 | 1.471668 | 6 | 1045.96 | 0.5086 |
| Vertical with raw surface (heat-treated) | 71 | 1.493654 | 6 | 1061.72 | 0.5187 |
| Vertical with raw surface (heat-treated) | 72 | 1.515573 | 6 | 1077.42 | 0.529 |
| Vertical with raw surface (heat-treated) | 73 | 1.537423 | 6 | 1093.08 | 0.5393 |
| Vertical with raw surface (heat-treated) | 74 | 1.5592 | 6 | 1108.68 | 0.5497 |
| Vertical with raw surface (heat-treated) | 75 | 1.5809 | 6 | 1124.24 | 0.5603 |
| Vertical with raw surface (heat-treated) | 76 | 1.602516 | 6 | 1139.74 | 0.571 |
| Vertical with raw surface (heat-treated) | 77 | 1.624039 | 6 | 1155.2 | 0.5818 |
| Vertical with raw surface (heat-treated) | 78 | 1.645459 | 6 | 1170.56 | 0.5926 |
| Vertical with raw surface (heat-treated) | 79 | 1.666762 | 6 | 1185.86 | 0.6034 |
| Vertical with raw surface (heat-treated) | 80 | 1.687931 | 6 | 1201.06 | 0.6142 |
| Vertical with raw surface (heat-treated) | 81 | 1.708946 | 6 | 1216.16 | 0.625 |
| Vertical with raw surface (heat-treated) | 82 | 1.729786 | 6 | 1231.12 | 0.6358 |
| Vertical with raw surface (heat-treated) | 83 | 1.750429 | 6 | 1245.96 | 0.6466 |
| Vertical with raw surface (heat-treated) | 84 | 1.770851 | 6 | 1260.64 | 0.6573 |
| Vertical with raw surface (heat-treated) | 85 | 1.791028 | 6 | 1275.14 | 0.6678 |
| Vertical with raw surface (heat-treated) | 86 | 1.810933 | 6 | 1289.46 | 0.6782 |
| Vertical with raw surface (heat-treated) | 87 | 1.830538 | 6 | 1303.56 | 0.6883 |
| Vertical with raw surface (heat-treated) | 88 | 1.849816 | 6 | 1317.42 | 0.6982 |
| Vertical with raw surface (heat-treated) | 89 | 1.868739 | 6 | 1331.02 | 0.7077 |
| Vertical with raw surface (heat-treated) | 90 | 1.887281 | 6 | 1344.36 | 0.7171 |
| Vertical with raw surface (heat-treated) | 91 | 1.905416 | 6 | 1357.4 | 0.7263 |
| Vertical with raw surface (heat-treated) | 92 | 1.923122 | 6 | 1370.14 | 0.7354 |
| Vertical with raw surface (heat-treated) | 93 | 1.940375 | 6 | 1382.56 | 0.7444 |
| Vertical with raw surface (heat-treated) | 94 | 1.957152 | 6 | 1394.64 | 0.7532 |
| Vertical with raw surface (heat-treated) | 95 | 1.973434 | 6 | 1406.36 | 0.7619 |
| Vertical with raw surface (heat-treated) | 96 | 1.9892 | 6 | 1417.72 | 0.7705 |
| Vertical with raw surface (heat-treated) | 97 | 2.004434 | 6 | 1428.7 | 0.7789 |
| Vertical with raw surface (heat-treated) | 98 | 2.01912 | 6 | 1439.3 | 0.7874 |
| Vertical with raw surface (heat-treated) | 99 | 2.033246 | 6 | 1449.5 | 0.7959 |
| Vertical with raw surface (heat-treated) | 100 | 2.046804 | 6 | 1459.3 | 0.8046 |
| Vertical with raw surface (heat-treated) | 101 | 2.059791 | 6 | 1468.68 | 0.8136 |
| Vertical with raw surface (heat-treated) | 102 | 2.072204 | 6 | 1477.68 | 0.8229 |
| Vertical with raw surface (heat-treated) | 103 | 2.084048 | 6 | 1486.28 | 0.8326 |
| Vertical with raw surface (heat-treated) | 104 | 2.095328 | 6 | 1494.46 | 0.8425 |
| Vertical with raw surface (heat-treated) | 105 | 2.106055 | 6 | 1502.28 | 0.8527 |
| Vertical with raw surface (heat-treated) | 106 | 2.11624 | 6 | 1509.7 | 0.8631 |
| Vertical with raw surface (heat-treated) | 107 | 2.125897 | 6 | 1516.74 | 0.8736 |
| Vertical with raw surface (heat-treated) | 108 | 2.135041 | 6 | 1523.42 | 0.8841 |
| Vertical with raw surface (heat-treated) | 109 | 2.143688 | 6 | 1529.76 | 0.8946 |
| Vertical with raw surface (heat-treated) | 110 | 2.151858 | 6 | 1535.76 | 0.9052 |
| Vertical with raw surface (heat-treated) | 111 | 2.159573 | 6 | 1541.42 | 0.9158 |
| Vertical with raw surface (heat-treated) | 112 | 2.166853 | 6 | 1546.78 | 0.9267 |
| Vertical with raw surface (heat-treated) | 113 | 2.173719 | 6 | 1551.86 | 0.9378 |
| Vertical with raw surface (heat-treated) | 114 | 2.180191 | 6 | 1556.66 | 0.9494 |
| Vertical with raw surface (heat-treated) | 115 | 2.186285 | 6 | 1561.2 | 0.9614 |
| Vertical with raw surface (heat-treated) | 116 | 2.192023 | 6 | 1565.48 | 0.9739 |
| Vertical with raw surface (heat-treated) | 117 | 2.197425 | 6 | 1569.54 | 0.9868 |
| Vertical with raw surface (heat-treated) | 118 | 2.202512 | 6 | 1573.38 | 1.0003 |
| Vertical with raw surface (heat-treated) | 119 | 2.207304 | 6 | 1577.02 | 1.0141 |
| Vertical with raw surface (heat-treated) | 120 | 1 | 6 | 1580.48 | 1.0281 |
| Vertical with raw surface (heat-treated) | 121 | 2.216074 | 6 | 1583.74 | 1.0423 |
| Vertical with raw surface (heat-treated) | 122 | 2.22008 | 6 | 1586.82 | 1.0566 |
| Vertical with raw surface (heat-treated) | 123 | 2.223849 | 6 | 1589.74 | 1.0709 |
| Vertical with raw surface (heat-treated) | 124 | 2.227393 | 6 | 1592.48 | 1.0851 |
| Vertical with raw surface (heat-treated) | 125 | 2.230725 | 6 | 1595.1 | 1.0992 |
| Vertical with raw surface (heat-treated) | 126 | 2.233857 | 6 | 1597.54 | 1.1132 |
| Vertical with raw surface (heat-treated) | 127 | 2.236806 | 6 | 1599.88 | 1.127 |
| Vertical with raw surface (heat-treated) | 128 | 2.239584 | 6 | 1602.08 | 1.1408 |
| Vertical with raw surface (heat-treated) | 129 | 2.242204 | 6 | 1604.18 | 1.1548 |
| Vertical with raw surface (heat-treated) | 130 | 2.24468 | 6 | 1606.16 | 1.169 |
| Vertical with raw surface (heat-treated) | 131 | 2.24702 | 6 | 1608.06 | 1.1835 |
| Vertical with raw surface (heat-treated) | 132 | 2.249233 | 6 | 1609.9 | 1.1985 |
| Vertical with raw surface (heat-treated) | 133 | 2.251329 | 6 | 1611.64 | 1.214 |
| Vertical with raw surface (heat-treated) | 134 | 2.253315 | 6 | 1613.32 | 1.2301 |
| Vertical with raw surface (heat-treated) | 135 | 2.255202 | 6 | 1614.92 | 1.2468 |
| Vertical with raw surface (heat-treated) | 136 | 2.256996 | 6 | 1616.48 | 1.264 |
| Vertical with raw surface (heat-treated) | 137 | 2.258704 | 6 | 1617.98 | 1.2816 |
| Vertical with raw surface (heat-treated) | 138 | 2.260333 | 6 | 1619.44 | 1.2996 |
| Vertical with raw surface (heat-treated) | 139 | 2.261891 | 6 | 1620.84 | 1.3178 |
| Vertical with raw surface (heat-treated) | 140 | 2.263384 | 6 | 1622.22 | 1.3362 |
| Vertical with raw surface (heat-treated) | 141 | 2.26482 | 6 | 1623.54 | 1.3547 |
| Vertical with raw surface (heat-treated) | 142 | 2.266203 | 6 | 1624.82 | 1.3733 |
| Vertical with raw surface (heat-treated) | 143 | 2.267541 | 6 | 1626.08 | 1.3919 |
| Vertical with raw surface (heat-treated) | 144 | 2.268839 | 6 | 1627.3 | 1.4104 |
| Vertical with raw surface (heat-treated) | 145 | 2.270106 | 6 | 1628.5 | 1.4289 |
| Vertical with raw surface (heat-treated) | 146 | 2.271344 | 6 | 1629.7 | 1.4474 |
| Vertical with raw surface (heat-treated) | 147 | 2.272557 | 6 | 1630.86 | 1.4659 |
| Vertical with raw surface (heat-treated) | 148 | 2.273742 | 6 | 1632 | 1.4845 |
| Vertical with raw surface (heat-treated) | 149 | 2.274896 | 6 | 1633.14 | 1.5033 |
| Vertical with raw surface (heat-treated) | 150 | 2.276017 | 6 | 1634.24 | 1.5224 |
| Vertical with raw surface (heat-treated) | 151 | 2.277104 | 6 | 1635.34 | 1.542 |
| Vertical with raw surface (heat-treated) | 152 | 2.278159 | 6 | 1636.42 | 1.5621 |
| Vertical with raw surface (heat-treated) | 153 | 2.279184 | 6 | 1637.48 | 1.5828 |
| Vertical with raw surface (heat-treated) | 154 | 2.280186 | 6 | 1638.54 | 1.6039 |
| Vertical with raw surface (heat-treated) | 155 | 2.281171 | 6 | 1639.6 | 1.6254 |
| Vertical with raw surface (heat-treated) | 156 | 2.282146 | 6 | 1640.66 | 1.6473 |
| Vertical with raw surface (heat-treated) | 157 | 2.283119 | 6 | 1641.72 | 1.6695 |
| Vertical with raw surface (heat-treated) | 158 | 2.284092 | 6 | 1642.78 | 1.6919 |
| Vertical with raw surface (heat-treated) | 159 | 2.285072 | 6 | 1643.84 | 1.7146 |
| Vertical with raw surface (heat-treated) | 160 | 2.28606 | 6 | 1644.92 | 1.7376 |
| Vertical with raw surface (heat-treated) | 161 | 2.287061 | 6 | 1646.02 | 1.761 |
| Vertical with raw surface (heat-treated) | 162 | 2.288079 | 6 | 1647.14 | 1.785 |
| Vertical with raw surface (heat-treated) | 163 | 2.289118 | 6 | 1648.3 | 1.8096 |
| Vertical with raw surface (heat-treated) | 164 | 2.290184 | 6 | 1649.48 | 1.8352 |
| Vertical with raw surface (heat-treated) | 165 | 2.291281 | 6 | 1650.7 | 1.8618 |
| Vertical with raw surface (heat-treated) | 166 | 2.292411 | 6 | 1651.98 | 1.8897 |
| Vertical with raw surface (heat-treated) | 167 | 2.293576 | 6 | 1653.28 | 1.9192 |
| Vertical with raw surface (heat-treated) | 168 | 2.294773 | 6 | 1654.66 | 1.9505 |
| Vertical with raw surface (heat-treated) | 169 | 2.295998 | 6 | 1656.08 | 1.9836 |
| Vertical with raw surface (heat-treated) | 170 | 2.29724 | 6 | 1657.56 | 2.0189 |
| Vertical with raw surface (heat-treated) | 171 | 2.298489 | 6 | 1659.06 | 2.0563 |
| Vertical with raw surface (heat-treated) | 172 | 2.299726 | 6 | 1660.62 | 2.0959 |
| Vertical with raw surface (heat-treated) | 173 | 2.300936 | 6 | 1662.16 | 2.1377 |
| Vertical with raw surface (heat-treated) | 174 | 2.302103 | 6 | 1663.72 | 2.1817 |
| Vertical with raw surface (heat-treated) | 175 | 2.303214 | 6 | 1665.28 | 2.2277 |
| Vertical with raw surface (heat-treated) | 176 | 2.30426 | 6 | 1666.82 | 2.2759 |
| Vertical with raw surface (heat-treated) | 177 | 2.305238 | 6 | 1668.36 | 2.3262 |
| Vertical with raw surface (heat-treated) | 178 | 2.306146 | 6 | 1669.88 | 2.3785 |
| Vertical with raw surface (heat-treated) | 179 | 2.306984 | 6 | 1671.38 | 2.4329 |
| Vertical with raw surface (heat-treated) | 180 | 2.307751 | 6 | 1672.86 | 2.4893 |
| Vertical with raw surface (heat-treated) | 181 | 2.30845 | 6 | 1674.32 | 2.5475 |
| Vertical with raw surface (heat-treated) | 182 | 2.309078 | 6 | 1675.76 | 2.6076 |
| Vertical with raw surface (heat-treated) | 183 | 2.309638 | 6 | 1677.18 | 2.6694 |
| Vertical with raw surface (heat-treated) | 184 | 2.310128 | 6 | 1678.58 | 2.7328 |
| Vertical with raw surface (heat-treated) | 185 | 2.310549 | 6 | 1679.96 | 2.7977 |
| Vertical with raw surface (heat-treated) | 186 | 2.310904 | 6 | 1681.3 | 2.8638 |
| Vertical with raw surface (heat-treated) | 187 | 2.311195 | 6 | 1682.62 | 2.931 |
| Vertical with raw surface (heat-treated) | 188 | 2.311424 | 6 | 1683.9 | 2.9991 |
| Vertical with raw surface (heat-treated) | 189 | 2.311597 | 6 | 1685.18 | 3.068 |
| Vertical with raw surface (heat-treated) | 190 | 2.31172 | 6 | 1686.4 | 3.1375 |
| Vertical with raw surface (heat-treated) | 191 | 2.3118 | 6 | 1687.62 | 3.2074 |
| Vertical with raw surface (heat-treated) | 192 | 2.311845 | 6 | 1688.82 | 3.2775 |
| Vertical with raw surface (heat-treated) | 193 | 2.311861 | 6 | 1690 | 3.3478 |
| Vertical with raw surface (heat-treated) | 194 | 2.311854 | 6 | 1691.16 | 3.4182 |
| Vertical with raw surface (heat-treated) | 195 | 2.311827 | 6 | 1692.3 | 3.4885 |
| Vertical with raw surface (heat-treated) | 196 | 2.311784 | 6 | 1693.44 | 3.5587 |
| Vertical with raw surface (heat-treated) | 197 | 2.311726 | 6 | 1694.56 | 3.6287 |
| Vertical with raw surface (heat-treated) | 198 | 2.311653 | 6 | 1695.66 | 3.6987 |
| Vertical with raw surface (heat-treated) | 199 | 2.311568 | 6 | 1696.76 | 3.7685 |
| Vertical with raw surface (heat-treated) | 200 | 2.311472 | 6 | 1697.86 | 3.8383 |
| Vertical with raw surface (heat-treated) | 201 | 2.311368 | 6 | 1698.94 | 3.9081 |
| Vertical with raw surface (heat-treated) | 202 | 2.311255 | 6 | 1700.02 | 3.9781 |
| Vertical with raw surface (heat-treated) | 203 | 2.311133 | 6 | 1701.1 | 4.0482 |
| Vertical with raw surface (heat-treated) | 204 | 2.311004 | 6 | 1702.18 | 4.1186 |
| Vertical with raw surface (heat-treated) | 205 | 2.310866 | 6 | 1703.26 | 4.1896 |
| Vertical with raw surface (heat-treated) | 206 | 2.310723 | 6 | 1704.36 | 4.2612 |
| Vertical with raw surface (heat-treated) | 207 | 2.31057 | 6 | 1705.46 | 4.3335 |
| Vertical with raw surface (heat-treated) | 208 | 2.310405 | 6 | 1706.56 | 4.4068 |
| Vertical with raw surface (heat-treated) | 209 | 2.310222 | 6 | 1707.66 | 4.4809 |
| Vertical with raw surface (heat-treated) | 210 | 2.310018 | 6 | 1708.78 | 4.5559 |
| Vertical with raw surface (heat-treated) | 211 | 2.30979 | 6 | 1709.88 | 4.6319 |
| Vertical with raw surface (heat-treated) | 212 | 2.309538 | 6 | 1710.98 | 4.7087 |
| Vertical with raw surface (heat-treated) | 213 | 2.309263 | 6 | 1712.1 | 4.7865 |
| Vertical with raw surface (heat-treated) | 214 | 2.308969 | 6 | 1713.2 | 4.8652 |
| Vertical with raw surface (heat-treated) | 215 | 2.308661 | 6 | 1714.3 | 4.9447 |
| Vertical with raw surface (heat-treated) | 216 | 2.308343 | 6 | 1715.42 | 5.0251 |
| Vertical with raw surface (heat-treated) | 217 | 2.308019 | 6 | 1716.56 | 5.1061 |
| Vertical with raw surface (heat-treated) | 218 | 2.307691 | 6 | 1717.68 | 5.1877 |
| Vertical with raw surface (heat-treated) | 219 | 2.307358 | 6 | 1718.82 | 5.2698 |
| Vertical with raw surface (heat-treated) | 220 | 2.307018 | 6 | 1719.96 | 5.3521 |
| Vertical with raw surface (heat-treated) | 221 | 2.306668 | 6 | 1721.1 | 5.4347 |
| Vertical with raw surface (heat-treated) | 222 | 2.306306 | 6 | 1722.22 | 5.5174 |
| Vertical with raw surface (heat-treated) | 223 | 2.305928 | 6 | 1723.34 | 5.6002 |
| Vertical with raw surface (heat-treated) | 224 | 2.305532 | 6 | 1724.46 | 5.6829 |
| Vertical with raw surface (heat-treated) | 225 | 2.305118 | 6 | 1725.54 | 5.7657 |
| Vertical with raw surface (heat-treated) | 226 | 2.304685 | 6 | 1726.62 | 5.8484 |
| Vertical with raw surface (heat-treated) | 227 | 2.304235 | 6 | 1727.7 | 5.931 |
| Vertical with raw surface (heat-treated) | 228 | 2.303767 | 6 | 1728.74 | 6.0137 |
| Vertical with raw surface (heat-treated) | 229 | 2.303283 | 6 | 1729.78 | 6.0965 |
| Vertical with raw surface (heat-treated) | 230 | 2.302783 | 6 | 1730.82 | 6.1795 |
| Vertical with raw surface (heat-treated) | 231 | 2.302265 | 6 | 1731.86 | 6.2629 |
| Vertical with raw surface (heat-treated) | 232 | 2.301731 | 6 | 1732.88 | 6.3466 |
| Vertical with raw surface (heat-treated) | 233 | 2.301182 | 6 | 1733.9 | 6.4308 |
| Vertical with raw surface (heat-treated) | 234 | 2.300617 | 6 | 1734.92 | 6.5156 |
| Vertical with raw surface (heat-treated) | 235 | 2.300038 | 6 | 1735.94 | 6.6012 |
| Vertical with raw surface (heat-treated) | 236 | 2.299441 | 6 | 1736.96 | 6.6875 |
| Vertical with raw surface (heat-treated) | 237 | 2.298828 | 6 | 1737.98 | 6.7747 |
| Vertical with raw surface (heat-treated) | 238 | 2.2982 | 6 | 1739.02 | 6.8628 |
| Vertical with raw surface (heat-treated) | 239 | 2.297559 | 6 | 1740.06 | 6.9519 |
| Vertical with raw surface (heat-treated) | 240 | 2.296904 | 6 | 1741.1 | 7.0422 |
| Vertical with raw surface (heat-treated) | 241 | 2.296235 | 6 | 1742.16 | 7.1336 |
| Vertical with raw surface (heat-treated) | 242 | 2.295551 | 6 | 1743.22 | 7.2263 |
| Vertical with raw surface (heat-treated) | 243 | 2.294848 | 6 | 1744.3 | 7.3202 |
| Vertical with raw surface (heat-treated) | 244 | 2.294124 | 6 | 1745.38 | 7.4152 |
| Vertical with raw surface (heat-treated) | 245 | 2.293378 | 6 | 1746.46 | 7.5116 |
| Vertical with raw surface (heat-treated) | 246 | 2.292609 | 6 | 1747.54 | 7.6091 |
| Vertical with raw surface (heat-treated) | 247 | 2.291817 | 6 | 1748.64 | 7.7079 |
| Vertical with raw surface (heat-treated) | 248 | 2.291004 | 6 | 1749.72 | 7.8078 |
| Vertical with raw surface (heat-treated) | 249 | 2.290171 | 6 | 1750.82 | 7.9086 |
| Vertical with raw surface (heat-treated) | 250 | 2.289319 | 6 | 1751.92 | 8.0103 |
| Vertical with raw surface (heat-treated) | 251 | 2.288448 | 6 | 1753.02 | 8.1128 |
| Vertical with raw surface (heat-treated) | 252 | 2.28756 | 6 | 1754.12 | 8.2159 |
| Vertical with raw surface (heat-treated) | 253 | 2.286654 | 6 | 1755.2 | 8.3196 |
| Vertical with raw surface (heat-treated) | 254 | 2.285733 | 6 | 1756.3 | 8.4237 |
| Vertical with raw surface (heat-treated) | 255 | 2.284797 | 6 | 1757.38 | 8.5282 |
| Vertical with raw surface (heat-treated) | 256 | 2.283852 | 6 | 1758.46 | 8.6329 |
| Vertical with raw surface (heat-treated) | 257 | 2.2829 | 6 | 1759.54 | 8.7377 |
| Vertical with raw surface (heat-treated) | 258 | 2.281948 | 6 | 1760.62 | 8.8427 |
| Vertical with raw surface (heat-treated) | 259 | 2.280999 | 6 | 1761.7 | 8.9478 |
| Vertical with raw surface (heat-treated) | 260 | 2.280055 | 6 | 1762.8 | 9.0532 |
| Vertical with raw surface (heat-treated) | 261 | 2.279117 | 6 | 1763.9 | 9.1587 |
| Vertical with raw surface (heat-treated) | 262 | 2.278181 | 6 | 1765 | 9.2645 |
| Vertical with raw surface (heat-treated) | 263 | 2.277243 | 6 | 1766.12 | 9.3706 |
| Vertical with raw surface (heat-treated) | 264 | 2.2763 | 6 | 1767.24 | 9.4771 |
| Vertical with raw surface (heat-treated) | 265 | 2.27535 | 6 | 1768.34 | 9.584 |
| Vertical with raw surface (heat-treated) | 266 | 2.27439 | 6 | 1769.46 | 9.6915 |
| Vertical with raw surface (heat-treated) | 267 | 2.273421 | 6 | 1770.6 | 9.7995 |
| Vertical with raw surface (heat-treated) | 268 | 2.272442 | 6 | 1771.72 | 9.9082 |
| Vertical with raw surface (heat-treated) | 269 | 2.271448 | 6 | 1772.84 | 10.0174 |
| Vertical with raw surface (heat-treated) | 270 | 2.270435 | 6 | 1773.96 | 10.1272 |
| Vertical with raw surface (heat-treated) | 271 | 2.269397 | 6 | 1775.08 | 10.2378 |
| Vertical with raw surface (heat-treated) | 272 | 2.268329 | 6 | 1776.18 | 10.3493 |
| Vertical with raw surface (heat-treated) | 273 | 2.267231 | 6 | 1777.28 | 10.4618 |
| Vertical with raw surface (heat-treated) | 274 | 2.266101 | 6 | 1778.38 | 10.5754 |
| Vertical with raw surface (heat-treated) | 275 | 2.26494 | 6 | 1779.46 | 10.69 |
| Vertical with raw surface (heat-treated) | 276 | 2.263752 | 6 | 1780.56 | 10.8058 |
| Vertical with raw surface (heat-treated) | 277 | 2.262538 | 6 | 1781.64 | 10.9228 |
| Vertical with raw surface (heat-treated) | 278 | 2.261302 | 6 | 1782.74 | 11.0408 |
| Vertical with raw surface (heat-treated) | 279 | 2.260045 | 6 | 1783.84 | 11.16 |
| Vertical with raw surface (heat-treated) | 280 | 2.258773 | 6 | 1784.94 | 11.2801 |
| Vertical with raw surface (heat-treated) | 281 | 2.257486 | 6 | 1786.04 | 11.4012 |
| Vertical with raw surface (heat-treated) | 282 | 2.256184 | 6 | 1787.14 | 11.523 |
| Vertical with raw surface (heat-treated) | 283 | 2.254865 | 6 | 1788.24 | 11.6454 |
| Vertical with raw surface (heat-treated) | 284 | 2.253525 | 6 | 1789.34 | 11.7685 |
| Vertical with raw surface (heat-treated) | 285 | 2.252163 | 6 | 1790.44 | 11.8922 |
| Vertical with raw surface (heat-treated) | 286 | 2.250778 | 6 | 1791.52 | 12.0165 |
| Vertical with raw surface (heat-treated) | 287 | 2.249369 | 6 | 1792.58 | 12.1413 |
| Vertical with raw surface (heat-treated) | 288 | 2.247939 | 6 | 1793.66 | 12.2666 |
| Vertical with raw surface (heat-treated) | 289 | 2.246489 | 6 | 1794.72 | 12.3925 |
| Vertical with raw surface (heat-treated) | 290 | 2.245023 | 6 | 1795.76 | 12.5189 |
| Vertical with raw surface (heat-treated) | 291 | 2.243541 | 6 | 1796.82 | 12.646 |
| Vertical with raw surface (heat-treated) | 292 | 2.242046 | 6 | 1797.88 | 12.7738 |
| Vertical with raw surface (heat-treated) | 293 | 2.240536 | 6 | 1798.94 | 12.9024 |
| Vertical with raw surface (heat-treated) | 294 | 2.239012 | 6 | 1800 | 13.0318 |
| Vertical with raw surface (heat-treated) | 295 | 2.237474 | 6 | 1801.08 | 13.1622 |
| Vertical with raw surface (heat-treated) | 296 | 2.23592 | 6 | 1802.14 | 13.2936 |
| Vertical with raw surface (heat-treated) | 297 | 2.23435 | 6 | 1803.22 | 13.426 |
| Vertical with raw surface (heat-treated) | 298 | 2.232766 | 6 | 1804.32 | 13.5595 |
| Vertical with raw surface (heat-treated) | 299 | 2.231171 | 6 | 1805.4 | 13.6939 |
| Vertical with raw surface (heat-treated) | 300 | 2.229568 | 6 | 1806.5 | 13.8291 |
| Vertical with raw surface (heat-treated) | 301 | 2.227959 | 6 | 1807.62 | 13.9652 |
| Vertical with raw surface (heat-treated) | 302 | 2.226347 | 6 | 1808.74 | 14.102 |
| Vertical with raw surface (heat-treated) | 303 | 2.224729 | 6 | 1809.86 | 14.2393 |
| Vertical with raw surface (heat-treated) | 304 | 2.223103 | 6 | 1810.98 | 14.3772 |
| Vertical with raw surface (heat-treated) | 305 | 2.221465 | 6 | 1812.1 | 14.5153 |
| Vertical with raw surface (heat-treated) | 306 | 2.21981 | 6 | 1813.22 | 14.6535 |
| Vertical with raw surface (heat-treated) | 307 | 2.218135 | 6 | 1814.3 | 14.7917 |
| Vertical with raw surface (heat-treated) | 308 | 2.216437 | 6 | 1815.38 | 14.9297 |
| Vertical with raw surface (heat-treated) | 309 | 2.214716 | 6 | 1816.42 | 15.0673 |
| Vertical with raw surface (heat-treated) | 310 | 2.212974 | 6 | 1817.44 | 15.2046 |
| Vertical with raw surface (heat-treated) | 311 | 2.211213 | 6 | 1818.42 | 15.3416 |
| Vertical with raw surface (heat-treated) | 312 | 2.209436 | 6 | 1819.4 | 15.4782 |
| Vertical with raw surface (heat-treated) | 313 | 2.207646 | 6 | 1820.36 | 15.6145 |
| Vertical with raw surface (heat-treated) | 314 | 2.205842 | 6 | 1821.3 | 15.7506 |
| Vertical with raw surface (heat-treated) | 315 | 2.204023 | 6 | 1822.24 | 15.8867 |
| Vertical with raw surface (heat-treated) | 316 | 2.202187 | 6 | 1823.14 | 16.0227 |
| Vertical with raw surface (heat-treated) | 317 | 2.200333 | 6 | 1824.04 | 16.1588 |
| Vertical with raw surface (heat-treated) | 318 | 2.198455 | 6 | 1824.92 | 16.295 |
| Vertical with raw surface (heat-treated) | 319 | 2.19655 | 6 | 1825.78 | 16.4314 |
| Vertical with raw surface (heat-treated) | 320 | 2.194616 | 6 | 1826.62 | 16.5682 |
| Vertical with raw surface (heat-treated) | 321 | 2.192651 | 6 | 1827.44 | 16.7054 |
| Vertical with raw surface (heat-treated) | 322 | 2.190658 | 6 | 1828.26 | 16.8432 |
| Vertical with raw surface (heat-treated) | 323 | 2.188639 | 6 | 1829.04 | 16.9816 |
| Vertical with raw surface (heat-treated) | 324 | 2.186597 | 6 | 1829.84 | 17.1207 |
| Vertical with raw surface (heat-treated) | 325 | 2.184537 | 6 | 1830.62 | 17.2606 |
| Vertical with raw surface (heat-treated) | 326 | 2.182465 | 6 | 1831.42 | 17.4013 |
| Vertical with raw surface (heat-treated) | 327 | 2.180387 | 6 | 1832.22 | 17.5429 |
| Vertical with raw surface (heat-treated) | 328 | 2.178303 | 6 | 1833.02 | 17.6854 |
| Vertical with raw surface (heat-treated) | 329 | 2.176215 | 6 | 1833.84 | 17.829 |
| Vertical with raw surface (heat-treated) | 330 | 2.17412 | 6 | 1834.68 | 17.9735 |
| Vertical with raw surface (heat-treated) | 331 | 2.172018 | 6 | 1835.52 | 18.1191 |
| Vertical with raw surface (heat-treated) | 332 | 2.169908 | 6 | 1836.38 | 18.2658 |
| Vertical with raw surface (heat-treated) | 333 | 2.167792 | 6 | 1837.26 | 18.4135 |
| Vertical with raw surface (heat-treated) | 334 | 2.16567 | 6 | 1838.14 | 18.5623 |
| Vertical with raw surface (heat-treated) | 335 | 2.163544 | 6 | 1839.04 | 18.7121 |
| Vertical with raw surface (heat-treated) | 336 | 2.161416 | 6 | 1839.94 | 18.863 |
| Vertical with raw surface (heat-treated) | 337 | 2.159286 | 6 | 1840.88 | 19.0147 |
| Vertical with raw surface (heat-treated) | 338 | 2.157156 | 6 | 1841.82 | 19.1672 |
| Vertical with raw surface (heat-treated) | 339 | 2.155025 | 6 | 1842.76 | 19.3207 |
| Vertical with raw surface (heat-treated) | 340 | 2.152892 | 6 | 1843.74 | 19.475 |
| Vertical with raw surface (heat-treated) | 341 | 2.150757 | 6 | 1844.7 | 19.63 |
| Vertical with raw surface (heat-treated) | 342 | 2.148618 | 6 | 1845.68 | 19.7856 |
| Vertical with raw surface (heat-treated) | 343 | 2.146473 | 6 | 1846.68 | 19.9418 |
| Vertical with raw surface (heat-treated) | 344 | 2.144318 | 6 | 1847.66 | 20.0986 |
| Vertical with raw surface (heat-treated) | 345 | 2.142152 | 6 | 1848.64 | 20.256 |
| Vertical with raw surface (heat-treated) | 346 | 2.13997 | 6 | 1849.62 | 20.4139 |
| Vertical with raw surface (heat-treated) | 347 | 2.137773 | 6 | 1850.6 | 20.5723 |
| Vertical with raw surface (heat-treated) | 348 | 2.135557 | 6 | 1851.56 | 20.7312 |
| Vertical with raw surface (heat-treated) | 349 | 2.133322 | 6 | 1852.52 | 20.8906 |
| Vertical with raw surface (heat-treated) | 350 | 2.131068 | 6 | 1853.46 | 21.0507 |
| Vertical with raw surface (heat-treated) | 351 | 2.128796 | 6 | 1854.4 | 21.2115 |
| Vertical with raw surface (heat-treated) | 352 | 2.126505 | 6 | 1855.34 | 21.373 |
| Vertical with raw surface (heat-treated) | 353 | 2.124196 | 6 | 1856.28 | 21.5355 |
| Vertical with raw surface (heat-treated) | 354 | 2.121865 | 6 | 1857.22 | 21.6989 |
| Vertical with raw surface (heat-treated) | 355 | 2.119511 | 6 | 1858.14 | 21.8633 |
| Vertical with raw surface (heat-treated) | 356 | 2.11713 | 6 | 1859.08 | 22.0287 |
| Vertical with raw surface (heat-treated) | 357 | 2.114719 | 6 | 1859.98 | 22.1952 |
| Vertical with raw surface (heat-treated) | 358 | 2.112277 | 6 | 1860.9 | 22.3628 |
| Vertical with raw surface (heat-treated) | 359 | 2.109806 | 6 | 1861.78 | 22.5315 |
| Vertical with raw surface (heat-treated) | 360 | 2.107311 | 6 | 1862.68 | 22.7012 |
| Vertical with raw surface (heat-treated) | 361 | 2.104796 | 6 | 1863.58 | 22.872 |
| Vertical with raw surface (heat-treated) | 362 | 2.102265 | 6 | 1864.48 | 23.0439 |
| Vertical with raw surface (heat-treated) | 363 | 2.099722 | 6 | 1865.38 | 23.2168 |
| Vertical with raw surface (heat-treated) | 364 | 2.097168 | 6 | 1866.28 | 23.3909 |
| Vertical with raw surface (heat-treated) | 365 | 2.0946 | 6 | 1867.2 | 23.5658 |
| Vertical with raw surface (heat-treated) | 366 | 2.092015 | 6 | 1868.12 | 23.7416 |
| Vertical with raw surface (heat-treated) | 367 | 2.089413 | 6 | 1869.02 | 23.9181 |
| Vertical with raw surface (heat-treated) | 368 | 2.086791 | 6 | 1869.92 | 24.0952 |
| Vertical with raw surface (heat-treated) | 369 | 2.084148 | 6 | 1870.8 | 24.2729 |
| Vertical with raw surface (heat-treated) | 370 | 2.081487 | 6 | 1871.68 | 24.451 |
| Vertical with raw surface (heat-treated) | 371 | 2.078814 | 6 | 1872.54 | 24.6296 |
| Vertical with raw surface (heat-treated) | 372 | 2.07613 | 6 | 1873.42 | 24.8085 |
| Vertical with raw surface (heat-treated) | 373 | 2.07344 | 6 | 1874.28 | 24.9877 |
| Vertical with raw surface (heat-treated) | 374 | 2.070745 | 6 | 1875.14 | 25.1671 |
| Vertical with raw surface (heat-treated) | 375 | 2.068047 | 6 | 1876 | 25.3467 |
| Vertical with raw surface (heat-treated) | 376 | 2.065346 | 6 | 1876.84 | 25.5266 |
| Vertical with raw surface (heat-treated) | 377 | 2.062645 | 6 | 1877.7 | 25.7069 |
| Vertical with raw surface (heat-treated) | 378 | 2.059947 | 6 | 1878.58 | 25.8873 |
| Vertical with raw surface (heat-treated) | 379 | 2.057257 | 6 | 1879.44 | 26.0681 |
| Vertical with raw surface (heat-treated) | 380 | 2.054573 | 6 | 1880.34 | 26.2492 |
| Vertical with raw surface (heat-treated) | 381 | 2.051895 | 6 | 1881.22 | 26.4308 |
| Vertical with raw surface (heat-treated) | 382 | 2.049219 | 6 | 1882.12 | 26.6128 |
| Vertical with raw surface (heat-treated) | 383 | 2.046541 | 6 | 1883.04 | 26.7954 |
| Vertical with raw surface (heat-treated) | 384 | 2.043859 | 6 | 1883.94 | 26.9786 |
| Vertical with raw surface (heat-treated) | 385 | 2.041171 | 6 | 1884.86 | 27.1625 |
| Vertical with raw surface (heat-treated) | 386 | 2.038474 | 6 | 1885.78 | 27.3472 |
| Vertical with raw surface (heat-treated) | 387 | 2.03577 | 6 | 1886.7 | 27.533 |
| Vertical with raw surface (heat-treated) | 388 | 2.033057 | 6 | 1887.64 | 27.7199 |
| Vertical with raw surface (heat-treated) | 389 | 2.030333 | 6 | 1888.58 | 27.9081 |
| Vertical with raw surface (heat-treated) | 390 | 2.027596 | 6 | 1889.54 | 28.0977 |
| Vertical with raw surface (heat-treated) | 391 | 2.024846 | 6 | 1890.52 | 28.2887 |
| Vertical with raw surface (heat-treated) | 392 | 2.022081 | 6 | 1891.5 | 28.4811 |
| Vertical with raw surface (heat-treated) | 393 | 2.019303 | 6 | 1892.48 | 28.6751 |
| Vertical with raw surface (heat-treated) | 394 | 2.016511 | 6 | 1893.48 | 28.8706 |
| Vertical with raw surface (heat-treated) | 395 | 2.013709 | 6 | 1894.52 | 29.0677 |
| Vertical with raw surface (heat-treated) | 396 | 2.010896 | 6 | 1895.54 | 29.2664 |
| Vertical with raw surface (heat-treated) | 397 | 2.008075 | 6 | 1896.6 | 29.4668 |
| Vertical with raw surface (heat-treated) | 398 | 2.005247 | 6 | 1897.68 | 29.669 |
| Vertical with raw surface (heat-treated) | 399 | 2.002409 | 6 | 1898.8 | 29.8732 |
| Vertical with raw surface (heat-treated) | 400 | 1.999562 | 6 | 1899.92 | 30.0796 |
| Vertical with raw surface (heat-treated) | 401 | 1.996703 | 6 | 1901.08 | 30.2882 |
| Vertical with raw surface (heat-treated) | 402 | 1.99383 | 6 | 1902.28 | 30.4993 |
| Vertical with raw surface (heat-treated) | 403 | 1.990942 | 6 | 1903.5 | 30.7127 |
| Vertical with raw surface (heat-treated) | 404 | 1.98804 | 6 | 1904.74 | 30.9285 |
| Vertical with raw surface (heat-treated) | 405 | 1.985122 | 6 | 1906.02 | 31.1467 |
| Vertical with raw surface (heat-treated) | 406 | 1.982193 | 6 | 1907.32 | 31.3673 |
| Vertical with raw surface (heat-treated) | 407 | 1.979253 | 6 | 1908.64 | 31.5902 |
| Vertical with raw surface (heat-treated) | 408 | 1.976304 | 6 | 1910 | 31.8152 |
| Vertical with raw surface (heat-treated) | 409 | 1.973348 | 6 | 1911.38 | 32.0424 |
| Vertical with raw surface (heat-treated) | 410 | 1.970384 | 6 | 1912.8 | 32.2716 |
| Vertical with raw surface (heat-treated) | 411 | 1.96741 | 6 | 1914.24 | 32.5026 |
| Vertical with raw surface (heat-treated) | 412 | 1.964422 | 6 | 1915.68 | 32.7354 |
| Vertical with raw surface (heat-treated) | 413 | 1.961413 | 6 | 1917.14 | 32.9699 |
| Vertical with raw surface (heat-treated) | 414 | 1.958382 | 6 | 1918.6 | 33.2058 |
| Vertical with raw surface (heat-treated) | 415 | 1.955329 | 6 | 1920.06 | 33.4431 |
| Vertical with raw surface (heat-treated) | 416 | 1.952257 | 6 | 1921.54 | 33.6817 |
| Vertical with raw surface (heat-treated) | 417 | 1.949165 | 6 | 1923 | 33.9215 |
| Vertical with raw surface (heat-treated) | 418 | 1.946056 | 6 | 1924.46 | 34.1624 |
| Vertical with raw surface (heat-treated) | 419 | 1.942929 | 6 | 1925.92 | 34.4043 |
| Vertical with raw surface (heat-treated) | 420 | 1.939786 | 6 | 1927.4 | 34.6471 |
| Vertical with raw surface (heat-treated) | 421 | 1.93663 | 6 | 1928.86 | 34.8908 |
| Vertical with raw surface (heat-treated) | 422 | 1.933467 | 6 | 1930.34 | 35.1353 |
| Vertical with raw surface (heat-treated) | 423 | 1.930303 | 6 | 1931.82 | 35.3807 |
| Vertical with raw surface (heat-treated) | 424 | 1.927144 | 6 | 1933.32 | 35.6268 |
| Vertical with raw surface (heat-treated) | 425 | 1.923991 | 6 | 1934.84 | 35.8734 |
| Vertical with raw surface (heat-treated) | 426 | 1.920845 | 6 | 1936.38 | 36.1206 |
| Vertical with raw surface (heat-treated) | 427 | 1.917704 | 6 | 1937.92 | 36.3681 |
| Vertical with raw surface (heat-treated) | 428 | 1.914565 | 6 | 1939.48 | 36.6159 |
| Vertical with raw surface (heat-treated) | 429 | 1.911427 | 6 | 1941.04 | 36.8639 |
| Vertical with raw surface (heat-treated) | 430 | 1.908289 | 6 | 1942.6 | 37.1119 |
| Vertical with machined surface (heat-treated) | 1 | 0 | 6 | 0 | 0 |
| Vertical with machined surface (heat-treated) | 2 | 0.007611 | 6 | 5.38 | 0.0036 |
| Vertical with machined surface (heat-treated) | 3 | 0.015258 | 6 | 10.78 | 0.0072 |
| Vertical with machined surface (heat-treated) | 4 | 0.022998 | 6 | 16.26 | 0.0107 |
| Vertical with machined surface (heat-treated) | 5 | 0.030909 | 6 | 21.86 | 0.0142 |
| Vertical with machined surface (heat-treated) | 6 | 0.039084 | 6 | 27.64 | 0.0177 |
| Vertical with machined surface (heat-treated) | 7 | 0.047627 | 6 | 33.68 | 0.0211 |
| Vertical with machined surface (heat-treated) | 8 | 0.056652 | 6 | 40.06 | 0.0244 |
| Vertical with machined surface (heat-treated) | 9 | 0.06627 | 6 | 46.86 | 0.0278 |
| Vertical with machined surface (heat-treated) | 10 | 0.076599 | 6 | 54.18 | 0.0312 |
| Vertical with machined surface (heat-treated) | 11 | 0.08775 | 6 | 62.06 | 0.0347 |
| Vertical with machined surface (heat-treated) | 12 | 0.099831 | 6 | 70.62 | 0.0384 |
| Vertical with machined surface (heat-treated) | 13 | 0.112947 | 6 | 79.9 | 0.0423 |
| Vertical with machined surface (heat-treated) | 14 | 0.127196 | 6 | 89.98 | 0.0466 |
| Vertical with machined surface (heat-treated) | 15 | 0.142656 | 6 | 100.92 | 0.0513 |
| Vertical with machined surface (heat-treated) | 16 | 0.159374 | 6 | 112.76 | 0.0563 |
| Vertical with machined surface (heat-treated) | 17 | 0.177362 | 6 | 125.5 | 0.0618 |
| Vertical with machined surface (heat-treated) | 18 | 0.196605 | 6 | 139.14 | 0.0676 |
| Vertical with machined surface (heat-treated) | 19 | 0.217063 | 6 | 153.62 | 0.0738 |
| Vertical with machined surface (heat-treated) | 20 | 0.23867 | 6 | 168.94 | 0.0803 |
| Vertical with machined surface (heat-treated) | 21 | 0.261342 | 6 | 185 | 0.0872 |
| Vertical with machined surface (heat-treated) | 22 | 0.28498 | 6 | 201.76 | 0.0944 |
| Vertical with machined surface (heat-treated) | 23 | 0.309481 | 6 | 219.12 | 0.1018 |
| Vertical with machined surface (heat-treated) | 24 | 0.33474 | 6 | 237.04 | 0.1095 |
| Vertical with machined surface (heat-treated) | 25 | 0.360652 | 6 | 255.4 | 0.1173 |
| Vertical with machined surface (heat-treated) | 26 | 0.387117 | 6 | 274.18 | 0.1254 |
| Vertical with machined surface (heat-treated) | 27 | 0.414036 | 6 | 293.28 | 0.1337 |
| Vertical with machined surface (heat-treated) | 28 | 0.441322 | 6 | 312.64 | 0.1422 |
| Vertical with machined surface (heat-treated) | 29 | 0.468892 | 6 | 332.2 | 0.151 |
| Vertical with machined surface (heat-treated) | 30 | 0.496672 | 6 | 351.92 | 0.1601 |
| Vertical with machined surface (heat-treated) | 31 | 0.52459 | 6 | 371.74 | 0.1694 |
| Vertical with machined surface (heat-treated) | 32 | 0.552583 | 6 | 391.62 | 0.179 |
| Vertical with machined surface (heat-treated) | 33 | 0.580594 | 6 | 411.5 | 0.1887 |
| Vertical with machined surface (heat-treated) | 34 | 0.608571 | 6 | 431.38 | 0.1986 |
| Vertical with machined surface (heat-treated) | 35 | 0.636469 | 6 | 451.2 | 0.2085 |
| Vertical with machined surface (heat-treated) | 36 | 0.664248 | 6 | 470.94 | 0.2183 |
| Vertical with machined surface (heat-treated) | 37 | 0.691878 | 6 | 490.58 | 0.228 |
| Vertical with machined surface (heat-treated) | 38 | 0.719333 | 6 | 510.1 | 0.2375 |
| Vertical with machined surface (heat-treated) | 39 | 0.746601 | 6 | 529.48 | 0.2469 |
| Vertical with machined surface (heat-treated) | 40 | 0.773672 | 6 | 548.72 | 0.256 |
| Vertical with machined surface (heat-treated) | 41 | 0.800546 | 6 | 567.84 | 0.2648 |
| Vertical with machined surface (heat-treated) | 42 | 0.82722 | 6 | 586.8 | 0.2732 |
| Vertical with machined surface (heat-treated) | 43 | 0.853697 | 6 | 605.64 | 0.2812 |
| Vertical with machined surface (heat-treated) | 44 | 0.879982 | 6 | 624.32 | 0.2886 |
| Vertical with machined surface (heat-treated) | 45 | 0.906079 | 6 | 642.88 | 0.2955 |
| Vertical with machined surface (heat-treated) | 46 | 0.931993 | 6 | 661.3 | 0.3018 |
| Vertical with machined surface (heat-treated) | 47 | 0.957727 | 6 | 679.6 | 0.3074 |
| Vertical with machined surface (heat-treated) | 48 | 0.983284 | 6 | 697.78 | 0.3123 |
| Vertical with machined surface (heat-treated) | 49 | 1.008666 | 6 | 715.82 | 0.3167 |
| Vertical with machined surface (heat-treated) | 50 | 1.033874 | 6 | 733.74 | 0.3206 |
| Vertical with machined surface (heat-treated) | 51 | 1.058911 | 6 | 751.54 | 0.3241 |
| Vertical with machined surface (heat-treated) | 52 | 1.08378 | 6 | 769.2 | 0.3273 |
| Vertical with machined surface (heat-treated) | 53 | 1.108487 | 6 | 786.76 | 0.3303 |
| Vertical with machined surface (heat-treated) | 54 | 1.133043 | 6 | 804.22 | 0.3332 |
| Vertical with machined surface (heat-treated) | 55 | 1.157458 | 6 | 821.58 | 0.336 |
| Vertical with machined surface (heat-treated) | 56 | 1.181744 | 6 | 838.84 | 0.3388 |
| Vertical with machined surface (heat-treated) | 57 | 1.205912 | 6 | 856.02 | 0.3418 |
| Vertical with machined surface (heat-treated) | 58 | 1.229973 | 6 | 873.14 | 0.3449 |
| Vertical with machined surface (heat-treated) | 59 | 1.253936 | 6 | 890.18 | 0.3484 |
| Vertical with machined surface (heat-treated) | 60 | 1.27781 | 6 | 907.18 | 0.3522 |
| Vertical with machined surface (heat-treated) | 61 | 1.301601 | 6 | 924.12 | 0.3565 |
| Vertical with machined surface (heat-treated) | 62 | 1.325314 | 6 | 941 | 0.3612 |
| Vertical with machined surface (heat-treated) | 63 | 1.348957 | 6 | 957.86 | 0.3665 |
| Vertical with machined surface (heat-treated) | 64 | 1.372535 | 6 | 974.66 | 0.3724 |
| Vertical with machined surface (heat-treated) | 65 | 1.396054 | 6 | 991.44 | 0.3789 |
| Vertical with machined surface (heat-treated) | 66 | 1.419514 | 6 | 1008.18 | 0.3861 |
| Vertical with machined surface (heat-treated) | 67 | 1.442918 | 6 | 1024.9 | 0.394 |
| Vertical with machined surface (heat-treated) | 68 | 1.466263 | 6 | 1041.58 | 0.4026 |
| Vertical with machined surface (heat-treated) | 69 | 1.489545 | 6 | 1058.22 | 0.4119 |
| Vertical with machined surface (heat-treated) | 70 | 1.512756 | 6 | 1074.84 | 0.4219 |
| Vertical with machined surface (heat-treated) | 71 | 1.535888 | 6 | 1091.38 | 0.4324 |
| Vertical with machined surface (heat-treated) | 72 | 1.558924 | 6 | 1107.88 | 0.4435 |
| Vertical with machined surface (heat-treated) | 73 | 1.58185 | 6 | 1124.32 | 0.455 |
| Vertical with machined surface (heat-treated) | 74 | 1.604646 | 6 | 1140.66 | 0.4669 |
| Vertical with machined surface (heat-treated) | 75 | 1.627291 | 6 | 1156.9 | 0.4791 |
| Vertical with machined surface (heat-treated) | 76 | 1.649768 | 6 | 1173.02 | 0.4914 |
| Vertical with machined surface (heat-treated) | 77 | 1.67206 | 6 | 1189.02 | 0.5037 |
| Vertical with machined surface (heat-treated) | 78 | 1.694149 | 6 | 1204.88 | 0.516 |
| Vertical with machined surface (heat-treated) | 79 | 1.716017 | 6 | 1220.56 | 0.5281 |
| Vertical with machined surface (heat-treated) | 80 | 1.737645 | 6 | 1236.1 | 0.5399 |
| Vertical with machined surface (heat-treated) | 81 | 1.75901 | 6 | 1251.44 | 0.5513 |
| Vertical with machined surface (heat-treated) | 82 | 1.780093 | 6 | 1266.56 | 0.5622 |
| Vertical with machined surface (heat-treated) | 83 | 1.800872 | 6 | 1281.48 | 0.5728 |
| Vertical with machined surface (heat-treated) | 84 | 1.821326 | 6 | 1296.16 | 0.5829 |
| Vertical with machined surface (heat-treated) | 85 | 1.841433 | 6 | 1310.6 | 0.5927 |
| Vertical with machined surface (heat-treated) | 86 | 1.861176 | 6 | 1324.76 | 0.6023 |
| Vertical with machined surface (heat-treated) | 87 | 1.880538 | 6 | 1338.66 | 0.6116 |
| Vertical with machined surface (heat-treated) | 88 | 1.899503 | 6 | 1352.28 | 0.6206 |
| Vertical with machined surface (heat-treated) | 89 | 1.918057 | 6 | 1365.62 | 0.6295 |
| Vertical with machined surface (heat-treated) | 90 | 1.936183 | 6 | 1378.62 | 0.6381 |
| Vertical with machined surface (heat-treated) | 91 | 1.953869 | 6 | 1391.34 | 0.6466 |
| Vertical with machined surface (heat-treated) | 92 | 1.9711 | 6 | 1403.72 | 0.6549 |
| Vertical with machined surface (heat-treated) | 93 | 1.987862 | 6 | 1415.76 | 0.663 |
| Vertical with machined surface (heat-treated) | 94 | 2.004144 | 6 | 1427.46 | 0.6707 |
| Vertical with machined surface (heat-treated) | 95 | 2.019937 | 6 | 1438.82 | 0.6781 |
| Vertical with machined surface (heat-treated) | 96 | 2.035231 | 6 | 1449.8 | 0.6852 |
| Vertical with machined surface (heat-treated) | 97 | 2.050022 | 6 | 1460.44 | 0.692 |
| Vertical with machined surface (heat-treated) | 98 | 2.064309 | 6 | 1470.72 | 0.6986 |
| Vertical with machined surface (heat-treated) | 99 | 2.078095 | 6 | 1480.64 | 0.7052 |
| Vertical with machined surface (heat-treated) | 100 | 2.091387 | 6 | 1490.2 | 0.7117 |
| Vertical with machined surface (heat-treated) | 101 | 2.104194 | 6 | 1499.44 | 0.7184 |
| Vertical with machined surface (heat-treated) | 102 | 2.116528 | 6 | 1508.34 | 0.7253 |
| Vertical with machined surface (heat-treated) | 103 | 2.128402 | 6 | 1516.92 | 0.7328 |
| Vertical with machined surface (heat-treated) | 104 | 2.139831 | 6 | 1525.2 | 0.7408 |
| Vertical with machined surface (heat-treated) | 105 | 2.150828 | 6 | 1533.16 | 0.7496 |
| Vertical with machined surface (heat-treated) | 106 | 2.161403 | 6 | 1540.86 | 0.7591 |
| Vertical with machined surface (heat-treated) | 107 | 2.171564 | 6 | 1548.28 | 0.7695 |
| Vertical with machined surface (heat-treated) | 108 | 2.181317 | 6 | 1555.42 | 0.7808 |
| Vertical with machined surface (heat-treated) | 109 | 2.190666 | 6 | 1562.28 | 0.7931 |
| Vertical with machined surface (heat-treated) | 110 | 2.199613 | 6 | 1568.86 | 0.8063 |
| Vertical with machined surface (heat-treated) | 111 | 2.20816 | 6 | 1575.18 | 0.8204 |
| Vertical with machined surface (heat-treated) | 112 | 2.216313 | 6 | 1581.24 | 0.8352 |
| Vertical with machined surface (heat-treated) | 113 | 2.224081 | 6 | 1587.04 | 0.8508 |
| Vertical with machined surface (heat-treated) | 114 | 2.231475 | 6 | 1592.58 | 0.8671 |
| Vertical with machined surface (heat-treated) | 115 | 2.238506 | 6 | 1597.86 | 0.8839 |
| Vertical with machined surface (heat-treated) | 116 | 2.245189 | 6 | 1602.9 | 0.9011 |
| Vertical with machined surface (heat-treated) | 117 | 2.251534 | 6 | 1607.72 | 0.9188 |
| Vertical with machined surface (heat-treated) | 118 | 2.257554 | 6 | 1612.3 | 0.9368 |
| Vertical with machined surface (heat-treated) | 119 | 2.263262 | 6 | 1616.66 | 0.9549 |
| Vertical with machined surface (heat-treated) | 120 | **2.268669** | 6 | **1620.82** | **0.9732** |
| Vertical with machined surface (heat-treated) | 121 | 2.273786 | 6 | 1624.76 | 0.9915 |
| Vertical with machined surface (heat-treated) | 122 | 2.278627 | 6 | 1628.52 | 1.0099 |
| Vertical with machined surface (heat-treated) | 123 | 2.283204 | 6 | 1632.08 | 1.0281 |
| Vertical with machined surface (heat-treated) | 124 | 2.287533 | 6 | 1635.46 | 1.0463 |
| Vertical with machined surface (heat-treated) | 125 | 2.291629 | 6 | 1638.68 | 1.0642 |
| Vertical with machined surface (heat-treated) | 126 | 2.295504 | 6 | 1641.72 | 1.0819 |
| Vertical with machined surface (heat-treated) | 127 | 2.299174 | 6 | 1644.64 | 1.0993 |
| Vertical with machined surface (heat-treated) | 128 | 2.302652 | 6 | 1647.4 | 1.1164 |
| Vertical with machined surface (heat-treated) | 129 | 2.305949 | 6 | 1650.02 | 1.133 |
| Vertical with machined surface (heat-treated) | 130 | 2.309077 | 6 | 1652.52 | 1.1494 |
| Vertical with machined surface (heat-treated) | 131 | 2.312045 | 6 | 1654.9 | 1.1654 |
| Vertical with machined surface (heat-treated) | 132 | 2.314863 | 6 | 1657.18 | 1.1811 |
| Vertical with machined surface (heat-treated) | 133 | 2.31754 | 6 | 1659.34 | 1.1966 |
| Vertical with machined surface (heat-treated) | 134 | 2.320087 | 6 | 1661.42 | 1.2119 |
| Vertical with machined surface (heat-treated) | 135 | 2.322508 | 6 | 1663.4 | 1.2272 |
| Vertical with machined surface (heat-treated) | 136 | 2.324808 | 6 | 1665.3 | 1.2425 |
| Vertical with machined surface (heat-treated) | 137 | 2.326988 | 6 | 1667.1 | 1.2579 |
| Vertical with machined surface (heat-treated) | 138 | 2.329054 | 6 | 1668.84 | 1.2734 |
| Vertical with machined surface (heat-treated) | 139 | 2.331011 | 6 | 1670.5 | 1.2891 |
| Vertical with machined surface (heat-treated) | 140 | 2.332865 | 6 | 1672.08 | 1.3049 |
| Vertical with machined surface (heat-treated) | 141 | 2.334623 | 6 | 1673.62 | 1.3209 |
| Vertical with machined surface (heat-treated) | 142 | 2.336293 | 6 | 1675.08 | 1.3371 |
| Vertical with machined surface (heat-treated) | 143 | 2.337881 | 6 | 1676.48 | 1.3535 |
| Vertical with machined surface (heat-treated) | 144 | 2.33939 | 6 | 1677.84 | 1.3703 |
| Vertical with machined surface (heat-treated) | 145 | 2.340823 | 6 | 1679.16 | 1.3875 |
| Vertical with machined surface (heat-treated) | 146 | 2.342182 | 6 | 1680.42 | 1.405 |
| Vertical with machined surface (heat-treated) | 147 | 2.343471 | 6 | 1681.64 | 1.4231 |
| Vertical with machined surface (heat-treated) | 148 | 2.344696 | 6 | 1682.84 | 1.4418 |
| Vertical with machined surface (heat-treated) | 149 | 2.345862 | 6 | 1684 | 1.4611 |
| Vertical with machined surface (heat-treated) | 150 | 2.346974 | 6 | 1685.12 | 1.4812 |
| Vertical with machined surface (heat-treated) | 151 | 2.348036 | 6 | 1686.24 | 1.502 |
| Vertical with machined surface (heat-treated) | 152 | 2.349053 | 6 | 1687.32 | 1.5236 |
| Vertical with machined surface (heat-treated) | 153 | 2.350031 | 6 | 1688.4 | 1.5458 |
| Vertical with machined surface (heat-treated) | 154 | 2.350973 | 6 | 1689.46 | 1.5689 |
| Vertical with machined surface (heat-treated) | 155 | 2.351884 | 6 | 1690.5 | 1.5927 |
| Vertical with machined surface (heat-treated) | 156 | 2.352766 | 6 | 1691.54 | 1.6172 |
| Vertical with machined surface (heat-treated) | 157 | 2.353624 | 6 | 1692.58 | 1.6426 |
| Vertical with machined surface (heat-treated) | 158 | 2.354459 | 6 | 1693.62 | 1.6686 |
| Vertical with machined surface (heat-treated) | 159 | 2.355276 | 6 | 1694.64 | 1.6951 |
| Vertical with machined surface (heat-treated) | 160 | 2.35608 | 6 | 1695.68 | 1.7222 |
| Vertical with machined surface (heat-treated) | 161 | 2.356879 | 6 | 1696.72 | 1.7496 |
| Vertical with machined surface (heat-treated) | 162 | 2.357678 | 6 | 1697.76 | 1.7774 |
| Vertical with machined surface (heat-treated) | 163 | 2.358484 | 6 | 1698.8 | 1.8055 |
| Vertical with machined surface (heat-treated) | 164 | 2.359298 | 6 | 1699.86 | 1.8339 |
| Vertical with machined surface (heat-treated) | 165 | 2.360124 | 6 | 1700.94 | 1.8625 |
| Vertical with machined surface (heat-treated) | 166 | 2.360964 | 6 | 1702.02 | 1.8912 |
| Vertical with machined surface (heat-treated) | 167 | 2.361822 | 6 | 1703.14 | 1.9202 |
| Vertical with machined surface (heat-treated) | 168 | 2.362696 | 6 | 1704.26 | 1.9495 |
| Vertical with machined surface (heat-treated) | 169 | 2.363588 | 6 | 1705.4 | 1.9791 |
| Vertical with machined surface (heat-treated) | 170 | 2.364497 | 6 | 1706.56 | 2.0092 |
| Vertical with machined surface (heat-treated) | 171 | 2.36542 | 6 | 1707.74 | 2.04 |
| Vertical with machined surface (heat-treated) | 172 | 2.366355 | 6 | 1708.94 | 2.0716 |
| Vertical with machined surface (heat-treated) | 173 | 2.367296 | 6 | 1710.18 | 2.1042 |
| Vertical with machined surface (heat-treated) | 174 | 2.368239 | 6 | 1711.42 | 2.1378 |
| Vertical with machined surface (heat-treated) | 175 | 2.369175 | 6 | 1712.68 | 2.1726 |
| Vertical with machined surface (heat-treated) | 176 | 2.370093 | 6 | 1713.94 | 2.2084 |
| Vertical with machined surface (heat-treated) | 177 | 2.370978 | 6 | 1715.22 | 2.2455 |
| Vertical with machined surface (heat-treated) | 178 | 2.37182 | 6 | 1716.46 | 2.2837 |
| Vertical with machined surface (heat-treated) | 179 | 2.372609 | 6 | 1717.7 | 2.3231 |
| Vertical with machined surface (heat-treated) | 180 | 2.373337 | 6 | 1718.9 | 2.3635 |
| Vertical with machined surface (heat-treated) | 181 | 2.373997 | 6 | 1720.08 | 2.4051 |
| Vertical with machined surface (heat-treated) | 182 | 2.374588 | 6 | 1721.24 | 2.4477 |
| Vertical with machined surface (heat-treated) | 183 | 2.375109 | 6 | 1722.34 | 2.4914 |
| Vertical with machined surface (heat-treated) | 184 | 2.375563 | 6 | 1723.44 | 2.5362 |
| Vertical with machined surface (heat-treated) | 185 | 2.375952 | 6 | 1724.5 | 2.5821 |
| Vertical with machined surface (heat-treated) | 186 | 2.376284 | 6 | 1725.52 | 2.6291 |
| Vertical with machined surface (heat-treated) | 187 | 2.376566 | 6 | 1726.54 | 2.6772 |
| Vertical with machined surface (heat-treated) | 188 | 2.376805 | 6 | 1727.56 | 2.7265 |
| Vertical with machined surface (heat-treated) | 189 | 2.377008 | 6 | 1728.56 | 2.7768 |
| Vertical with machined surface (heat-treated) | 190 | 2.377173 | 6 | 1729.54 | 2.8281 |
| Vertical with machined surface (heat-treated) | 191 | 2.3773 | 6 | 1730.52 | 2.8805 |
| Vertical with machined surface (heat-treated) | 192 | 2.377389 | 6 | 1731.5 | 2.9338 |
| Vertical with machined surface (heat-treated) | 193 | 2.377447 | 6 | 1732.46 | 2.9879 |
| Vertical with machined surface (heat-treated) | 194 | 2.37748 | 6 | 1733.42 | 3.0429 |
| Vertical with machined surface (heat-treated) | 195 | 2.377495 | 6 | 1734.38 | 3.0988 |
| Vertical with machined surface (heat-treated) | 196 | **2.377496** | 6 | **1735.34** | **3.1554** |
| Vertical with machined surface (heat-treated) | 197 | 2.377486 | 6 | 1736.3 | 3.2128 |
| Vertical with machined surface (heat-treated) | 198 | 2.377465 | 6 | 1737.28 | 3.271 |
| Vertical with machined surface (heat-treated) | 199 | 2.377434 | 6 | 1738.26 | 3.3298 |
| Vertical with machined surface (heat-treated) | 200 | 2.377396 | 6 | 1739.24 | 3.3893 |
| Vertical with machined surface (heat-treated) | 201 | 2.37735 | 6 | 1740.24 | 3.4492 |
| Vertical with machined surface (heat-treated) | 202 | 2.377296 | 6 | 1741.22 | 3.5097 |
| Vertical with machined surface (heat-treated) | 203 | 2.377234 | 6 | 1742.22 | 3.5706 |
| Vertical with machined surface (heat-treated) | 204 | 2.377162 | 6 | 1743.22 | 3.632 |
| Vertical with machined surface (heat-treated) | 205 | 2.377078 | 6 | 1744.22 | 3.6937 |
| Vertical with machined surface (heat-treated) | 206 | 2.376978 | 6 | 1745.2 | 3.7558 |
| Vertical with machined surface (heat-treated) | 207 | 2.376861 | 6 | 1746.18 | 3.8184 |
| Vertical with machined surface (heat-treated) | 208 | 2.376721 | 6 | 1747.16 | 3.8813 |
| Vertical with machined surface (heat-treated) | 209 | 2.376559 | 6 | 1748.12 | 3.9445 |
| Vertical with machined surface (heat-treated) | 210 | 2.376375 | 6 | 1749.08 | 4.0081 |
| Vertical with machined surface (heat-treated) | 211 | 2.376173 | 6 | 1750.04 | 4.0719 |
| Vertical with machined surface (heat-treated) | 212 | 2.375953 | 6 | 1750.98 | 4.1361 |
| Vertical with machined surface (heat-treated) | 213 | 2.375718 | 6 | 1751.92 | 4.2007 |
| Vertical with machined surface (heat-treated) | 214 | 2.375468 | 6 | 1752.84 | 4.2657 |
| Vertical with machined surface (heat-treated) | 215 | 2.375205 | 6 | 1753.78 | 4.3312 |
| Vertical with machined surface (heat-treated) | 216 | 2.374929 | 6 | 1754.72 | 4.3973 |
| Vertical with machined surface (heat-treated) | 217 | 2.374642 | 6 | 1755.66 | 4.4641 |
| Vertical with machined surface (heat-treated) | 218 | 2.374345 | 6 | 1756.62 | 4.5316 |
| Vertical with machined surface (heat-treated) | 219 | 2.37404 | 6 | 1757.56 | 4.5997 |
| Vertical with machined surface (heat-treated) | 220 | 2.373729 | 6 | 1758.52 | 4.6685 |
| Vertical with machined surface (heat-treated) | 221 | 2.373415 | 6 | 1759.5 | 4.7379 |
| Vertical with machined surface (heat-treated) | 222 | 2.373099 | 6 | 1760.46 | 4.8078 |
| Vertical with machined surface (heat-treated) | 223 | 2.372783 | 6 | 1761.46 | 4.8781 |
| Vertical with machined surface (heat-treated) | 224 | 2.372468 | 6 | 1762.44 | 4.9488 |
| Vertical with machined surface (heat-treated) | 225 | 2.372153 | 6 | 1763.44 | 5.0197 |
| Vertical with machined surface (heat-treated) | 226 | 2.371838 | 6 | 1764.44 | 5.0908 |
| Vertical with machined surface (heat-treated) | 227 | 2.37152 | 6 | 1765.44 | 5.162 |
| Vertical with machined surface (heat-treated) | 228 | 2.371195 | 6 | 1766.42 | 5.2333 |
| Vertical with machined surface (heat-treated) | 229 | 2.370859 | 6 | 1767.4 | 5.3044 |
| Vertical with machined surface (heat-treated) | 230 | 2.370509 | 6 | 1768.38 | 5.3755 |
| Vertical with machined surface (heat-treated) | 231 | 2.370141 | 6 | 1769.34 | 5.4465 |
| Vertical with machined surface (heat-treated) | 232 | 2.369753 | 6 | 1770.28 | 5.5176 |
| Vertical with machined surface (heat-treated) | 233 | 2.369342 | 6 | 1771.2 | 5.5888 |
| Vertical with machined surface (heat-treated) | 234 | 2.36891 | 6 | 1772.12 | 5.6603 |
| Vertical with machined surface (heat-treated) | 235 | 2.368457 | 6 | 1773.02 | 5.7322 |
| Vertical with machined surface (heat-treated) | 236 | 2.367988 | 6 | 1773.92 | 5.8047 |
| Vertical with machined surface (heat-treated) | 237 | 2.367505 | 6 | 1774.84 | 5.8781 |
| Vertical with machined surface (heat-treated) | 238 | 2.36701 | 6 | 1775.76 | 5.9525 |
| Vertical with machined surface (heat-treated) | 239 | 2.366501 | 6 | 1776.68 | 6.0282 |
| Vertical with machined surface (heat-treated) | 240 | 2.365978 | 6 | 1777.64 | 6.1054 |
| Vertical with machined surface (heat-treated) | 241 | 2.365437 | 6 | 1778.6 | 6.1842 |
| Vertical with machined surface (heat-treated) | 242 | 2.364878 | 6 | 1779.58 | 6.2649 |
| Vertical with machined surface (heat-treated) | 243 | 2.3643 | 6 | 1780.58 | 6.3474 |
| Vertical with machined surface (heat-treated) | 244 | 2.363705 | 6 | 1781.6 | 6.4318 |
| Vertical with machined surface (heat-treated) | 245 | 2.363095 | 6 | 1782.66 | 6.5183 |
| Vertical with machined surface (heat-treated) | 246 | 2.362477 | 6 | 1783.72 | 6.6069 |
| Vertical with machined surface (heat-treated) | 247 | 2.361856 | 6 | 1784.84 | 6.6973 |
| Vertical with machined surface (heat-treated) | 248 | 2.361236 | 6 | 1785.98 | 6.7894 |
| Vertical with machined surface (heat-treated) | 249 | 2.360609 | 6 | 1787.14 | 6.883 |
| Vertical with machined surface (heat-treated) | 250 | 2.359971 | 6 | 1788.32 | 6.9779 |
| Vertical with machined surface (heat-treated) | 251 | 2.359317 | 6 | 1789.52 | 7.0739 |
| Vertical with machined surface (heat-treated) | 252 | 2.358649 | 6 | 1790.7 | 7.1709 |
| Vertical with machined surface (heat-treated) | 253 | 2.357968 | 6 | 1791.9 | 7.2688 |
| Vertical with machined surface (heat-treated) | 254 | 2.357275 | 6 | 1793.1 | 7.3672 |
| Vertical with machined surface (heat-treated) | 255 | 2.356572 | 6 | 1794.32 | 7.4663 |
| Vertical with machined surface (heat-treated) | 256 | 2.355856 | 6 | 1795.52 | 7.5658 |
| Vertical with machined surface (heat-treated) | 257 | 2.355127 | 6 | 1796.72 | 7.6657 |
| Vertical with machined surface (heat-treated) | 258 | 2.354383 | 6 | 1797.92 | 7.766 |
| Vertical with machined surface (heat-treated) | 259 | 2.353623 | 6 | 1799.12 | 7.8665 |
| Vertical with machined surface (heat-treated) | 260 | 2.352846 | 6 | 1800.3 | 7.9673 |
| Vertical with machined surface (heat-treated) | 261 | 2.352049 | 6 | 1801.48 | 8.0685 |
| Vertical with machined surface (heat-treated) | 262 | 2.351233 | 6 | 1802.66 | 8.17 |
| Vertical with machined surface (heat-treated) | 263 | 2.350394 | 6 | 1803.82 | 8.272 |
| Vertical with machined surface (heat-treated) | 264 | 2.349532 | 6 | 1804.96 | 8.3745 |
| Vertical with machined surface (heat-treated) | 265 | 2.348644 | 6 | 1806.12 | 8.4777 |
| Vertical with machined surface (heat-treated) | 266 | 2.347728 | 6 | 1807.26 | 8.5816 |
| Vertical with machined surface (heat-treated) | 267 | 2.346782 | 6 | 1808.38 | 8.6862 |
| Vertical with machined surface (heat-treated) | 268 | 2.345809 | 6 | 1809.5 | 8.7915 |
| Vertical with machined surface (heat-treated) | 269 | 2.344808 | 6 | 1810.62 | 8.8977 |
| Vertical with machined surface (heat-treated) | 270 | 2.343782 | 6 | 1811.74 | 9.0046 |
| Vertical with machined surface (heat-treated) | 271 | 2.342733 | 6 | 1812.84 | 9.1122 |
| Vertical with machined surface (heat-treated) | 272 | 2.341662 | 6 | 1813.94 | 9.2204 |
| Vertical with machined surface (heat-treated) | 273 | 2.340571 | 6 | 1815.04 | 9.3292 |
| Vertical with machined surface (heat-treated) | 274 | 2.339462 | 6 | 1816.14 | 9.4387 |
| Vertical with machined surface (heat-treated) | 275 | 2.338337 | 6 | 1817.22 | 9.5489 |
| Vertical with machined surface (heat-treated) | 276 | 2.337199 | 6 | 1818.32 | 9.6598 |
| Vertical with machined surface (heat-treated) | 277 | 2.33605 | 6 | 1819.42 | 9.7714 |
| Vertical with machined surface (heat-treated) | 278 | 2.334894 | 6 | 1820.54 | 9.8837 |
| Vertical with machined surface (heat-treated) | 279 | 2.333736 | 6 | 1821.66 | 9.9969 |
| Vertical with machined surface (heat-treated) | 280 | 2.33258 | 6 | 1822.8 | 10.111 |
| Vertical with machined surface (heat-treated) | 281 | 2.331436 | 6 | 1823.96 | 10.226 |
| Vertical with machined surface (heat-treated) | 282 | 2.330314 | 6 | 1825.18 | 10.3422 |
| Vertical with machined surface (heat-treated) | 283 | 2.329211 | 6 | 1826.42 | 10.4595 |
| Vertical with machined surface (heat-treated) | 284 | 2.328121 | 6 | 1827.7 | 10.578 |
| Vertical with machined surface (heat-treated) | 285 | 2.327034 | 6 | 1829 | 10.6978 |
| Vertical with machined surface (heat-treated) | 286 | 2.32594 | 6 | 1830.32 | 10.8189 |
| Vertical with machined surface (heat-treated) | 287 | 2.324831 | 6 | 1831.64 | 10.9412 |
| Vertical with machined surface (heat-treated) | 288 | 2.323701 | 6 | 1832.98 | 11.0647 |
| Vertical with machined surface (heat-treated) | 289 | 2.32255 | 6 | 1834.32 | 11.1893 |
| Vertical with machined surface (heat-treated) | 290 | 2.321375 | 6 | 1835.66 | 11.3151 |
| Vertical with machined surface (heat-treated) | 291 | 2.320174 | 6 | 1837 | 11.4419 |
| Vertical with machined surface (heat-treated) | 292 | 2.318947 | 6 | 1838.32 | 11.5697 |
| Vertical with machined surface (heat-treated) | 293 | 2.317691 | 6 | 1839.66 | 11.6984 |
| Vertical with machined surface (heat-treated) | 294 | 2.316405 | 6 | 1840.98 | 11.8281 |
| Vertical with machined surface (heat-treated) | 295 | 2.315091 | 6 | 1842.3 | 11.9585 |
| Vertical with machined surface (heat-treated) | 296 | 2.313752 | 6 | 1843.6 | 12.0896 |
| Vertical with machined surface (heat-treated) | 297 | 2.312388 | 6 | 1844.9 | 12.2214 |
| Vertical with machined surface (heat-treated) | 298 | 2.311001 | 6 | 1846.2 | 12.3537 |
| Vertical with machined surface (heat-treated) | 299 | 2.30959 | 6 | 1847.48 | 12.4866 |
| Vertical with machined surface (heat-treated) | 300 | 2.308152 | 6 | 1848.76 | 12.6199 |
| Vertical with machined surface (heat-treated) | 301 | 2.306686 | 6 | 1850 | 12.7536 |
| Vertical with machined surface (heat-treated) | 302 | 2.305191 | 6 | 1851.24 | 12.8876 |
| Vertical with machined surface (heat-treated) | 303 | 2.303669 | 6 | 1852.48 | 13.0221 |
| Vertical with machined surface (heat-treated) | 304 | 2.302123 | 6 | 1853.68 | 13.1569 |
| Vertical with machined surface (heat-treated) | 305 | 2.300552 | 6 | 1854.88 | 13.2922 |
| Vertical with machined surface (heat-treated) | 306 | 2.298959 | 6 | 1856.08 | 13.4279 |
| Vertical with machined surface (heat-treated) | 307 | 2.297342 | 6 | 1857.26 | 13.5641 |
| Vertical with machined surface (heat-treated) | 308 | 2.295702 | 6 | 1858.42 | 13.701 |
| Vertical with machined surface (heat-treated) | 309 | 2.294039 | 6 | 1859.6 | 13.8384 |
| Vertical with machined surface (heat-treated) | 310 | 2.292351 | 6 | 1860.76 | 13.9766 |
| Vertical with machined surface (heat-treated) | 311 | 2.290639 | 6 | 1861.9 | 14.1155 |
| Vertical with machined surface (heat-treated) | 312 | 2.288905 | 6 | 1863.06 | 14.2552 |
| Vertical with machined surface (heat-treated) | 313 | 2.287152 | 6 | 1864.2 | 14.3957 |
| Vertical with machined surface (heat-treated) | 314 | 2.285386 | 6 | 1865.36 | 14.537 |
| Vertical with machined surface (heat-treated) | 315 | 2.28361 | 6 | 1866.52 | 14.6791 |
| Vertical with machined surface (heat-treated) | 316 | 2.281829 | 6 | 1867.68 | 14.8221 |
| Vertical with machined surface (heat-treated) | 317 | 2.280044 | 6 | 1868.86 | 14.966 |
| Vertical with machined surface (heat-treated) | 318 | 2.278257 | 6 | 1870.06 | 15.1108 |
| Vertical with machined surface (heat-treated) | 319 | 2.276468 | 6 | 1871.28 | 15.2565 |
| Vertical with machined surface (heat-treated) | 320 | 2.274678 | 6 | 1872.5 | 15.403 |
| Vertical with machined surface (heat-treated) | 321 | 2.272887 | 6 | 1873.74 | 15.5503 |
| Vertical with machined surface (heat-treated) | 322 | 2.271096 | 6 | 1875 | 15.6984 |
| Vertical with machined surface (heat-treated) | 323 | 2.269304 | 6 | 1876.26 | 15.8473 |
| Vertical with machined surface (heat-treated) | 324 | 2.267506 | 6 | 1877.54 | 15.9968 |
| Vertical with machined surface (heat-treated) | 325 | 2.2657 | 6 | 1878.82 | 16.147 |
| Vertical with machined surface (heat-treated) | 326 | 2.26388 | 6 | 1880.1 | 16.2979 |
| Vertical with machined surface (heat-treated) | 327 | 2.262043 | 6 | 1881.38 | 16.4495 |
| Vertical with machined surface (heat-treated) | 328 | 2.260186 | 6 | 1882.66 | 16.602 |
| Vertical with machined surface (heat-treated) | 329 | 2.258309 | 6 | 1883.94 | 16.7552 |
| Vertical with machined surface (heat-treated) | 330 | 2.256416 | 6 | 1885.2 | 16.909 |
| Vertical with machined surface (heat-treated) | 331 | 2.254507 | 6 | 1886.48 | 17.0636 |
| Vertical with machined surface (heat-treated) | 332 | 2.252587 | 6 | 1887.76 | 17.2188 |
| Vertical with machined surface (heat-treated) | 333 | 2.250657 | 6 | 1889.04 | 17.3745 |
| Vertical with machined surface (heat-treated) | 334 | 2.248717 | 6 | 1890.3 | 17.5308 |
| Vertical with machined surface (heat-treated) | 335 | 2.246765 | 6 | 1891.58 | 17.6874 |
| Vertical with machined surface (heat-treated) | 336 | 2.244801 | 6 | 1892.84 | 17.8444 |
| Vertical with machined surface (heat-treated) | 337 | 2.242823 | 6 | 1894.1 | 18.0017 |
| Vertical with machined surface (heat-treated) | 338 | 2.240826 | 6 | 1895.36 | 18.1592 |
| Vertical with machined surface (heat-treated) | 339 | 2.23881 | 6 | 1896.58 | 18.317 |
| Vertical with machined surface (heat-treated) | 340 | 2.236772 | 6 | 1897.8 | 18.475 |
| Vertical with machined surface (heat-treated) | 341 | 2.234713 | 6 | 1899 | 18.6332 |
| Vertical with machined surface (heat-treated) | 342 | 2.232634 | 6 | 1900.2 | 18.7916 |
| Vertical with machined surface (heat-treated) | 343 | 2.230537 | 6 | 1901.38 | 18.9502 |
| Vertical with machined surface (heat-treated) | 344 | 2.228423 | 6 | 1902.54 | 19.1089 |
| Vertical with machined surface (heat-treated) | 345 | 2.226292 | 6 | 1903.68 | 19.2678 |
| Vertical with machined surface (heat-treated) | 346 | 2.224144 | 6 | 1904.82 | 19.4271 |
| Vertical with machined surface (heat-treated) | 347 | 2.22198 | 6 | 1905.96 | 19.5867 |
| Vertical with machined surface (heat-treated) | 348 | 2.219798 | 6 | 1907.08 | 19.7467 |
| Vertical with machined surface (heat-treated) | 349 | 2.217601 | 6 | 1908.2 | 19.9072 |
| Vertical with machined surface (heat-treated) | 350 | 2.21539 | 6 | 1909.32 | 20.0682 |
| Vertical with machined surface (heat-treated) | 351 | 2.213164 | 6 | 1910.42 | 20.2297 |
| Vertical with machined surface (heat-treated) | 352 | 2.210924 | 6 | 1911.52 | 20.3916 |
| Vertical with machined surface (heat-treated) | 353 | 2.208668 | 6 | 1912.62 | 20.554 |
| Vertical with machined surface (heat-treated) | 354 | 2.206397 | 6 | 1913.72 | 20.7169 |
| Vertical with machined surface (heat-treated) | 355 | 2.204112 | 6 | 1914.8 | 20.8805 |
| Vertical with machined surface (heat-treated) | 356 | 2.201817 | 6 | 1915.9 | 21.0448 |
| Vertical with machined surface (heat-treated) | 357 | 2.199514 | 6 | 1917 | 21.2101 |
| Vertical with machined surface (heat-treated) | 358 | 2.197202 | 6 | 1918.12 | 21.3763 |
| Vertical with machined surface (heat-treated) | 359 | 2.194882 | 6 | 1919.24 | 21.5435 |
| Vertical with machined surface (heat-treated) | 360 | 2.192552 | 6 | 1920.38 | 21.7118 |
| Vertical with machined surface (heat-treated) | 361 | 2.190208 | 6 | 1921.52 | 21.881 |
| Vertical with machined surface (heat-treated) | 362 | 2.187851 | 6 | 1922.66 | 22.0512 |
| Vertical with machined surface (heat-treated) | 363 | 2.185478 | 6 | 1923.8 | 22.2224 |
| Vertical with machined surface (heat-treated) | 364 | 2.183091 | 6 | 1924.96 | 22.3946 |
| Vertical with machined surface (heat-treated) | 365 | 2.180691 | 6 | 1926.1 | 22.5677 |
| Vertical with machined surface (heat-treated) | 366 | 2.178278 | 6 | 1927.26 | 22.7415 |
| Vertical with machined surface (heat-treated) | 367 | 2.175853 | 6 | 1928.42 | 22.9161 |
| Vertical with machined surface (heat-treated) | 368 | 2.173415 | 6 | 1929.56 | 23.0914 |
| Vertical with machined surface (heat-treated) | 369 | 2.170961 | 6 | 1930.72 | 23.2672 |
| Vertical with machined surface (heat-treated) | 370 | 2.168489 | 6 | 1931.86 | 23.4434 |
| Vertical with machined surface (heat-treated) | 371 | 2.165997 | 6 | 1932.98 | 23.6201 |
| Vertical with machined surface (heat-treated) | 372 | 2.163491 | 6 | 1934.1 | 23.7971 |
| Vertical with machined surface (heat-treated) | 373 | 2.160973 | 6 | 1935.22 | 23.9745 |
| Vertical with machined surface (heat-treated) | 374 | 2.158447 | 6 | 1936.34 | 24.1522 |
| Vertical with machined surface (heat-treated) | 375 | 2.155915 | 6 | 1937.44 | 24.3304 |
| Vertical with machined surface (heat-treated) | 376 | 2.153379 | 6 | 1938.56 | 24.509 |
| Vertical with machined surface (heat-treated) | 377 | 2.150835 | 6 | 1939.68 | 24.688 |
| Vertical with machined surface (heat-treated) | 378 | 2.148283 | 6 | 1940.8 | 24.8676 |
| Vertical with machined surface (heat-treated) | 379 | 2.145722 | 6 | 1941.9 | 25.0477 |
| Vertical with machined surface (heat-treated) | 380 | 2.14315 | 6 | 1943.02 | 25.2282 |
| Vertical with machined surface (heat-treated) | 381 | 2.140565 | 6 | 1944.12 | 25.4092 |
| Vertical with machined surface (heat-treated) | 382 | 2.137969 | 6 | 1945.24 | 25.5906 |
| Vertical with machined surface (heat-treated) | 383 | 2.135361 | 6 | 1946.32 | 25.7722 |
| Vertical with machined surface (heat-treated) | 384 | 2.132739 | 6 | 1947.4 | 25.954 |
| Vertical with machined surface (heat-treated) | 385 | 2.130105 | 6 | 1948.48 | 26.1358 |
| Vertical with machined surface (heat-treated) | 386 | 2.127457 | 6 | 1949.52 | 26.3176 |
| Vertical with machined surface (heat-treated) | 387 | 2.124796 | 6 | 1950.56 | 26.4995 |
| Vertical with machined surface (heat-treated) | 388 | 2.122124 | 6 | 1951.6 | 26.6815 |
| Vertical with machined surface (heat-treated) | 389 | 2.119444 | 6 | 1952.62 | 26.8637 |
| Vertical with machined surface (heat-treated) | 390 | 2.116759 | 6 | 1953.64 | 27.0462 |
| Vertical with machined surface (heat-treated) | 391 | 2.114069 | 6 | 1954.66 | 27.2293 |
| Vertical with machined surface (heat-treated) | 392 | 2.111379 | 6 | 1955.7 | 27.4128 |
| Vertical with machined surface (heat-treated) | 393 | 2.108689 | 6 | 1956.74 | 27.5971 |
| Vertical with machined surface (heat-treated) | 394 | 2.106 | 6 | 1957.8 | 27.7822 |
| Vertical with machined surface (heat-treated) | 395 | 2.103314 | 6 | 1958.88 | 27.9681 |
| Vertical with machined surface (heat-treated) | 396 | 2.10063 | 6 | 1959.96 | 28.1548 |
| Vertical with machined surface (heat-treated) | 397 | 2.097947 | 6 | 1961.06 | 28.3425 |
| Vertical with machined surface (heat-treated) | 398 | 2.095265 | 6 | 1962.18 | 28.5311 |
| Vertical with machined surface (heat-treated) | 399 | 2.092582 | 6 | 1963.32 | 28.7205 |
| Vertical with machined surface (heat-treated) | 400 | 2.089898 | 6 | 1964.46 | 28.9107 |
| Vertical with machined surface (heat-treated) | 401 | 2.087214 | 6 | 1965.62 | 29.1019 |
| Vertical with machined surface (heat-treated) | 402 | 2.08453 | 6 | 1966.8 | 29.2939 |
| Vertical with machined surface (heat-treated) | 403 | 2.081843 | 6 | 1967.98 | 29.4868 |
| Vertical with machined surface (heat-treated) | 404 | 2.079148 | 6 | 1969.18 | 29.6805 |
| Vertical with machined surface (heat-treated) | 405 | 2.076438 | 6 | 1970.36 | 29.875 |
| Vertical with machined surface (heat-treated) | 406 | 2.073706 | 6 | 1971.54 | 30.0704 |
| Vertical with machined surface (heat-treated) | 407 | 2.070947 | 6 | 1972.72 | 30.2667 |
| Vertical with machined surface (heat-treated) | 408 | 2.068154 | 6 | 1973.86 | 30.4638 |
| Vertical with machined surface (heat-treated) | 409 | 2.065321 | 6 | 1975 | 30.662 |
| Vertical with machined surface (heat-treated) | 410 | 2.062448 | 6 | 1976.1 | 30.8611 |
| Vertical with machined surface (heat-treated) | 411 | 2.059533 | 6 | 1977.18 | 31.0611 |
| Vertical with machined surface (heat-treated) | 412 | 2.056576 | 6 | 1978.24 | 31.262 |
| Vertical with machined surface (heat-treated) | 413 | 2.053581 | 6 | 1979.28 | 31.4637 |
| Vertical with machined surface (heat-treated) | 414 | 2.050552 | 6 | 1980.3 | 31.666 |
| Vertical with machined surface (heat-treated) | 415 | 2.047497 | 6 | 1981.3 | 31.8688 |
| Vertical with machined surface (heat-treated) | 416 | 2.044423 | 6 | 1982.28 | 32.0721 |
| Vertical with machined surface (heat-treated) | 417 | 2.041336 | 6 | 1983.26 | 32.2756 |
| Vertical with machined surface (heat-treated) | 418 | 2.038242 | 6 | 1984.24 | 32.4792 |
| Vertical with machined surface (heat-treated) | 419 | 2.035146 | 6 | 1985.2 | 32.683 |
| Vertical with machined surface (heat-treated) | 420 | 2.032049 | 6 | 1986.18 | 32.8867 |
| Horizontal with machined surface (heat-treated) | 1 | 0 | 6 | 0 | 0 |
| Horizontal with machined surface (heat-treated) | 2 | 0.021538 | 6 | 15.26 | 0.0046 |
| Horizontal with machined surface (heat-treated) | 3 | 0.043112 | 6 | 30.54 | 0.0092 |
| Horizontal with machined surface (heat-treated) | 4 | 0.064775 | 6 | 45.88 | 0.0137 |
| Horizontal with machined surface (heat-treated) | 5 | 0.086603 | 6 | 61.34 | 0.0183 |
| Horizontal with machined surface (heat-treated) | 6 | 0.108681 | 6 | 76.98 | 0.0229 |
| Horizontal with machined surface (heat-treated) | 7 | 0.131103 | 6 | 92.86 | 0.0275 |
| Horizontal with machined surface (heat-treated) | 8 | 0.153964 | 6 | 109.06 | 0.0322 |
| Horizontal with machined surface (heat-treated) | 9 | 0.177366 | 6 | 125.64 | 0.037 |
| Horizontal with machined surface (heat-treated) | 10 | 0.201409 | 6 | 142.68 | 0.0418 |
| Horizontal with machined surface (heat-treated) | 11 | 0.226189 | 6 | 160.24 | 0.0468 |
| Horizontal with machined surface (heat-treated) | 12 | 0.251791 | 6 | 178.38 | 0.0521 |
| Horizontal with machined surface (heat-treated) | 13 | 0.278289 | 6 | 197.16 | 0.0576 |
| Horizontal with machined surface (heat-treated) | 14 | 0.30574 | 6 | 216.62 | 0.0635 |
| Horizontal with machined surface (heat-treated) | 15 | 0.334183 | 6 | 236.78 | 0.0698 |
| Horizontal with machined surface (heat-treated) | 16 | 0.363637 | 6 | 257.66 | 0.0767 |
| Horizontal with machined surface (heat-treated) | 17 | 0.394098 | 6 | 279.26 | 0.084 |
| Horizontal with machined surface (heat-treated) | 18 | 0.425546 | 6 | 301.56 | 0.092 |
| Horizontal with machined surface (heat-treated) | 19 | 0.457936 | 6 | 324.54 | 0.1004 |
| Horizontal with machined surface (heat-treated) | 20 | 0.491206 | 6 | 348.14 | 0.1095 |
| Horizontal with machined surface (heat-treated) | 21 | 0.525274 | 6 | 372.32 | 0.1191 |
| Horizontal with machined surface (heat-treated) | 22 | 0.560041 | 6 | 397 | 0.1292 |
| Horizontal with machined surface (heat-treated) | 23 | 0.595394 | 6 | 422.1 | 0.1398 |
| Horizontal with machined surface (heat-treated) | 24 | 0.631211 | 6 | 447.54 | 0.1509 |
| Horizontal with machined surface (heat-treated) | 25 | 0.667366 | 6 | 473.2 | 0.1625 |
| Horizontal with machined surface (heat-treated) | 26 | 0.703737 | 6 | 499.04 | 0.1745 |
| Horizontal with machined surface (heat-treated) | 27 | 0.740203 | 6 | 524.96 | 0.1869 |
| Horizontal with machined surface (heat-treated) | 28 | 0.77665 | 6 | 550.86 | 0.1996 |
| Horizontal with machined surface (heat-treated) | 29 | 0.81297 | 6 | 576.68 | 0.2125 |
| Horizontal with machined surface (heat-treated) | 30 | 0.84906 | 6 | 602.32 | 0.2257 |
| Horizontal with machined surface (heat-treated) | 31 | 0.884824 | 6 | 627.76 | 0.2389 |
| Horizontal with machined surface (heat-treated) | 32 | 0.920174 | 6 | 652.9 | 0.252 |
| Horizontal with machined surface (heat-treated) | 33 | 0.955029 | 6 | 677.7 | 0.2651 |
| Horizontal with machined surface (heat-treated) | 34 | 0.98932 | 6 | 702.08 | 0.278 |
| Horizontal with machined surface (heat-treated) | 35 | 1.022991 | 6 | 726.04 | 0.2906 |
| Horizontal with machined surface (heat-treated) | 36 | 1.056001 | 6 | 749.54 | 0.3029 |
| Horizontal with machined surface (heat-treated) | 37 | 1.088324 | 6 | 772.54 | 0.3147 |
| Horizontal with machined surface (heat-treated) | 38 | 1.119949 | 6 | 795.06 | 0.3262 |
| Horizontal with machined surface (heat-treated) | 39 | 1.15088 | 6 | 817.08 | 0.3373 |
| Horizontal with machined surface (heat-treated) | 40 | 1.181134 | 6 | 838.62 | 0.3479 |
| Horizontal with machined surface (heat-treated) | 41 | 1.210741 | 6 | 859.7 | 0.358 |
| Horizontal with machined surface (heat-treated) | 42 | 1.239733 | 6 | 880.34 | 0.3677 |
| Horizontal with machined surface (heat-treated) | 43 | 1.268143 | 6 | 900.58 | 0.377 |
| Horizontal with machined surface (heat-treated) | 44 | 1.296 | 6 | 920.42 | 0.3858 |
| Horizontal with machined surface (heat-treated) | 45 | 1.323324 | 6 | 939.88 | 0.3943 |
| Horizontal with machined surface (heat-treated) | 46 | 1.350129 | 6 | 958.98 | 0.4023 |
| Horizontal with machined surface (heat-treated) | 47 | 1.376414 | 6 | 977.72 | 0.41 |
| Horizontal with machined surface (heat-treated) | 48 | 1.402168 | 6 | 996.06 | 0.4173 |
| Horizontal with machined surface (heat-treated) | 49 | 1.427368 | 6 | 1014.02 | 0.4243 |
| Horizontal with machined surface (heat-treated) | 50 | 1.451993 | 6 | 1031.58 | 0.431 |
| Horizontal with machined surface (heat-treated) | 51 | 1.476043 | 6 | 1048.72 | 0.4375 |
| Horizontal with machined surface (heat-treated) | 52 | 1.499547 | 6 | 1065.48 | 0.4437 |
| Horizontal with machined surface (heat-treated) | 53 | 1.52254 | 6 | 1081.86 | 0.4497 |
| Horizontal with machined surface (heat-treated) | 54 | 1.545055 | 6 | 1097.92 | 0.4556 |
| Horizontal with machined surface (heat-treated) | 55 | 1.567123 | 6 | 1113.66 | 0.4613 |
| Horizontal with machined surface (heat-treated) | 56 | 1.588772 | 6 | 1129.12 | 0.4668 |
| Horizontal with machined surface (heat-treated) | 57 | 1.610027 | 6 | 1144.28 | 0.4723 |
| Horizontal with machined surface (heat-treated) | 58 | 1.630914 | 6 | 1159.18 | 0.4778 |
| Horizontal with machined surface (heat-treated) | 59 | 1.651453 | 6 | 1173.86 | 0.4833 |
| Horizontal with machined surface (heat-treated) | 60 | 1.671663 | 6 | 1188.28 | 0.4889 |
| Horizontal with machined surface (heat-treated) | 61 | 1.691563 | 6 | 1202.5 | 0.4946 |
| Horizontal with machined surface (heat-treated) | 62 | 1.711171 | 6 | 1216.52 | 0.5006 |
| Horizontal with machined surface (heat-treated) | 63 | 1.730501 | 6 | 1230.34 | 0.5068 |
| Horizontal with machined surface (heat-treated) | 64 | 1.749566 | 6 | 1243.98 | 0.5133 |
| Horizontal with machined surface (heat-treated) | 65 | 1.768376 | 6 | 1257.44 | 0.52 |
| Horizontal with machined surface (heat-treated) | 66 | 1.786937 | 6 | 1270.74 | 0.5272 |
| Horizontal with machined surface (heat-treated) | 67 | 1.805254 | 6 | 1283.88 | 0.5347 |
| Horizontal with machined surface (heat-treated) | 68 | 1.823329 | 6 | 1296.84 | 0.5427 |
| Horizontal with machined surface (heat-treated) | 69 | 1.841161 | 6 | 1309.64 | 0.5511 |
| Horizontal with machined surface (heat-treated) | 70 | 1.858747 | 6 | 1322.26 | 0.56 |
| Horizontal with machined surface (heat-treated) | 71 | 1.876083 | 6 | 1334.74 | 0.5694 |
| Horizontal with machined surface (heat-treated) | 72 | 1.893167 | 6 | 1347.02 | 0.5794 |
| Horizontal with machined surface (heat-treated) | 73 | 1.909992 | 6 | 1359.14 | 0.5898 |
| Horizontal with machined surface (heat-treated) | 74 | 1.926553 | 6 | 1371.08 | 0.6008 |
| Horizontal with machined surface (heat-treated) | 75 | 1.942843 | 6 | 1382.84 | 0.6123 |
| Horizontal with machined surface (heat-treated) | 76 | 1.958854 | 6 | 1394.4 | 0.6244 |
| Horizontal with machined surface (heat-treated) | 77 | 1.974576 | 6 | 1405.76 | 0.637 |
| Horizontal with machined surface (heat-treated) | 78 | 1.99 | 6 | 1416.92 | 0.6502 |
| Horizontal with machined surface (heat-treated) | 79 | 2.005111 | 6 | 1427.88 | 0.6638 |
| Horizontal with machined surface (heat-treated) | 80 | 2.019896 | 6 | 1438.6 | 0.678 |
| Horizontal with machined surface (heat-treated) | 81 | 2.034341 | 6 | 1449.1 | 0.6926 |
| Horizontal with machined surface (heat-treated) | 82 | 2.048436 | 6 | 1459.36 | 0.7077 |
| Horizontal with machined surface (heat-treated) | 83 | 2.062168 | 6 | 1469.36 | 0.7231 |
| Horizontal with machined surface (heat-treated) | 84 | 2.075528 | 6 | 1479.1 | 0.739 |
| Horizontal with machined surface (heat-treated) | 85 | 2.088504 | 6 | 1488.58 | 0.7551 |
| Horizontal with machined surface (heat-treated) | 86 | 2.101086 | 6 | 1497.78 | 0.7716 |
| Horizontal with machined surface (heat-treated) | 87 | 2.113262 | 6 | 1506.7 | 0.7883 |
| Horizontal with machined surface (heat-treated) | 88 | 2.125024 | 6 | 1515.32 | 0.8053 |
| Horizontal with machined surface (heat-treated) | 89 | 2.136365 | 6 | 1523.66 | 0.8224 |
| Horizontal with machined surface (heat-treated) | 90 | 2.147278 | 6 | 1531.7 | 0.8397 |
| Horizontal with machined surface (heat-treated) | 91 | 2.15776 | 6 | 1539.42 | 0.8571 |
| Horizontal with machined surface (heat-treated) | 92 | 2.167809 | 6 | 1546.84 | 0.8745 |
| Horizontal with machined surface (heat-treated) | 93 | **2.177424** | 6 | **1553.96** | **0.892** |
| Horizontal with machined surface (heat-treated) | 94 | 2.186606 | 6 | 1560.78 | 0.9095 |
| Horizontal with machined surface (heat-treated) | 95 | 2.19536 | 6 | 1567.28 | 0.927 |
| Horizontal with machined surface (heat-treated) | 96 | 2.203691 | 6 | 1573.48 | 0.9447 |
| Horizontal with machined surface (heat-treated) | 97 | 2.211607 | 6 | 1579.4 | 0.9624 |
| Horizontal with machined surface (heat-treated) | 98 | 2.219117 | 6 | 1585.02 | 0.9803 |
| Horizontal with machined surface (heat-treated) | 99 | 2.226231 | 6 | 1590.38 | 0.9984 |
| Horizontal with machined surface (heat-treated) | 100 | 2.23296 | 6 | 1595.46 | 1.0167 |
| Horizontal with machined surface (heat-treated) | 101 | 2.239315 | 6 | 1600.28 | 1.0354 |
| Horizontal with machined surface (heat-treated) | 102 | 2.245308 | 6 | 1604.86 | 1.0545 |
| Horizontal with machined surface (heat-treated) | 103 | 2.250953 | 6 | 1609.2 | 1.0741 |
| Horizontal with machined surface (heat-treated) | 104 | 2.256263 | 6 | 1613.3 | 1.0941 |
| Horizontal with machined surface (heat-treated) | 105 | 2.261256 | 6 | 1617.18 | 1.1147 |
| Horizontal with machined surface (heat-treated) | 106 | 2.265946 | 6 | 1620.86 | 1.1359 |
| Horizontal with machined surface (heat-treated) | 107 | 2.27035 | 6 | 1624.34 | 1.1578 |
| Horizontal with machined surface (heat-treated) | 108 | 2.274484 | 6 | 1627.66 | 1.1804 |
| Horizontal with machined surface (heat-treated) | 109 | 2.278365 | 6 | 1630.8 | 1.2038 |
| Horizontal with machined surface (heat-treated) | 110 | 2.282007 | 6 | 1633.78 | 1.2281 |
| Horizontal with machined surface (heat-treated) | 111 | 2.285426 | 6 | 1636.62 | 1.2531 |
| Horizontal with machined surface (heat-treated) | 112 | 2.288637 | 6 | 1639.32 | 1.279 |
| Horizontal with machined surface (heat-treated) | 113 | 2.291655 | 6 | 1641.9 | 1.3057 |
| Horizontal with machined surface (heat-treated) | 114 | 2.294494 | 6 | 1644.38 | 1.3331 |
| Horizontal with machined surface (heat-treated) | 115 | 2.297169 | 6 | 1646.74 | 1.3613 |
| Horizontal with machined surface (heat-treated) | 116 | 2.299693 | 6 | 1649 | 1.3901 |
| Horizontal with machined surface (heat-treated) | 117 | 2.302079 | 6 | 1651.18 | 1.4196 |
| Horizontal with machined surface (heat-treated) | 118 | 2.30434 | 6 | 1653.28 | 1.4497 |
| Horizontal with machined surface (heat-treated) | 119 | 2.306485 | 6 | 1655.3 | 1.4803 |
| Horizontal with machined surface (heat-treated) | 120 | 2.308524 | 6 | 1657.26 | 1.5114 |
| Horizontal with machined surface (heat-treated) | 121 | 2.310468 | 6 | 1659.16 | 1.543 |
| Horizontal with machined surface (heat-treated) | 122 | 2.312323 | 6 | 1661 | 1.5749 |
| Horizontal with machined surface (heat-treated) | 123 | 2.314097 | 6 | 1662.78 | 1.6072 |
| Horizontal with machined surface (heat-treated) | 124 | 2.315797 | 6 | 1664.52 | 1.6397 |
| Horizontal with machined surface (heat-treated) | 125 | 2.317428 | 6 | 1666.22 | 1.6725 |
| Horizontal with machined surface (heat-treated) | 126 | 2.318996 | 6 | 1667.88 | 1.7054 |
| Horizontal with machined surface (heat-treated) | 127 | 2.320501 | 6 | 1669.48 | 1.7384 |
| Horizontal with machined surface (heat-treated) | 128 | 2.321948 | 6 | 1671.06 | 1.7714 |
| Horizontal with machined surface (heat-treated) | 129 | 2.323339 | 6 | 1672.58 | 1.8044 |
| Horizontal with machined surface (heat-treated) | 130 | 2.324676 | 6 | 1674.08 | 1.8372 |
| Horizontal with machined surface (heat-treated) | 131 | 2.325961 | 6 | 1675.52 | 1.8699 |
| Horizontal with machined surface (heat-treated) | 132 | 2.327197 | 6 | 1676.94 | 1.9025 |
| Horizontal with machined surface (heat-treated) | 133 | 2.328388 | 6 | 1678.32 | 1.9348 |
| Horizontal with machined surface (heat-treated) | 134 | 2.329539 | 6 | 1679.66 | 1.967 |
| Horizontal with machined surface (heat-treated) | 135 | 2.330652 | 6 | 1680.98 | 1.9989 |
| Horizontal with machined surface (heat-treated) | 136 | 2.331731 | 6 | 1682.26 | 2.0307 |
| Horizontal with machined surface (heat-treated) | 137 | 2.33278 | 6 | 1683.54 | 2.0624 |
| Horizontal with machined surface (heat-treated) | 138 | 2.333798 | 6 | 1684.78 | 2.0939 |
| Horizontal with machined surface (heat-treated) | 139 | 2.334787 | 6 | 1686 | 2.1254 |
| Horizontal with machined surface (heat-treated) | 140 | 2.335749 | 6 | 1687.2 | 2.1568 |
| Horizontal with machined surface (heat-treated) | 141 | 2.336689 | 6 | 1688.38 | 2.1882 |
| Horizontal with machined surface (heat-treated) | 142 | 2.33761 | 6 | 1689.56 | 2.2196 |
| Horizontal with machined surface (heat-treated) | 143 | 2.338517 | 6 | 1690.72 | 2.251 |
| Horizontal with machined surface (heat-treated) | 144 | 2.339412 | 6 | 1691.88 | 2.2823 |
| Horizontal with machined surface (heat-treated) | 145 | 2.340297 | 6 | 1693.02 | 2.3137 |
| Horizontal with machined surface (heat-treated) | 146 | 2.341173 | 6 | 1694.18 | 2.345 |
| Horizontal with machined surface (heat-treated) | 147 | 2.342039 | 6 | 1695.3 | 2.3765 |
| Horizontal with machined surface (heat-treated) | 148 | 2.342895 | 6 | 1696.44 | 2.408 |
| Horizontal with machined surface (heat-treated) | 149 | 2.343738 | 6 | 1697.56 | 2.4396 |
| Horizontal with machined surface (heat-treated) | 150 | 2.344567 | 6 | 1698.68 | 2.4713 |
| Horizontal with machined surface (heat-treated) | 151 | 2.345381 | 6 | 1699.78 | 2.5031 |
| Horizontal with machined surface (heat-treated) | 152 | 2.346179 | 6 | 1700.88 | 2.5351 |
| Horizontal with machined surface (heat-treated) | 153 | 2.34696 | 6 | 1701.98 | 2.5672 |
| Horizontal with machined surface (heat-treated) | 154 | 2.347725 | 6 | 1703.06 | 2.5993 |
| Horizontal with machined surface (heat-treated) | 155 | 2.348472 | 6 | 1704.12 | 2.6314 |
| Horizontal with machined surface (heat-treated) | 156 | 2.349202 | 6 | 1705.16 | 2.6634 |
| Horizontal with machined surface (heat-treated) | 157 | 2.349918 | 6 | 1706.2 | 2.6953 |
| Horizontal with machined surface (heat-treated) | 158 | 2.350622 | 6 | 1707.24 | 2.7271 |
| Horizontal with machined surface (heat-treated) | 159 | 2.351316 | 6 | 1708.26 | 2.7587 |
| Horizontal with machined surface (heat-treated) | 160 | 2.352 | 6 | 1709.26 | 2.7902 |
| Horizontal with machined surface (heat-treated) | 161 | 2.352675 | 6 | 1710.28 | 2.8215 |
| Horizontal with machined surface (heat-treated) | 162 | 2.35334 | 6 | 1711.26 | 2.8526 |
| Horizontal with machined surface (heat-treated) | 163 | 2.353996 | 6 | 1712.24 | 2.8835 |
| Horizontal with machined surface (heat-treated) | 164 | 2.35464 | 6 | 1713.22 | 2.9142 |
| Horizontal with machined surface (heat-treated) | 165 | 2.355274 | 6 | 1714.18 | 2.9448 |
| Horizontal with machined surface (heat-treated) | 166 | 2.355897 | 6 | 1715.14 | 2.9753 |
| Horizontal with machined surface (heat-treated) | 167 | 2.356512 | 6 | 1716.08 | 3.0056 |
| Horizontal with machined surface (heat-treated) | 168 | 2.357117 | 6 | 1717.02 | 3.0359 |
| Horizontal with machined surface (heat-treated) | 169 | 2.357711 | 6 | 1717.94 | 3.0661 |
| Horizontal with machined surface (heat-treated) | 170 | 2.358292 | 6 | 1718.86 | 3.0964 |
| Horizontal with machined surface (heat-treated) | 171 | 2.358858 | 6 | 1719.78 | 3.1266 |
| Horizontal with machined surface (heat-treated) | 172 | 2.35941 | 6 | 1720.68 | 3.1568 |
| Horizontal with machined surface (heat-treated) | 173 | 2.359947 | 6 | 1721.56 | 3.1871 |
| Horizontal with machined surface (heat-treated) | 174 | 2.36047 | 6 | 1722.44 | 3.2173 |
| Horizontal with machined surface (heat-treated) | 175 | 2.36098 | 6 | 1723.32 | 3.2475 |
| Horizontal with machined surface (heat-treated) | 176 | 2.361476 | 6 | 1724.18 | 3.2778 |
| Horizontal with machined surface (heat-treated) | 177 | 2.361958 | 6 | 1725.02 | 3.3081 |
| Horizontal with machined surface (heat-treated) | 178 | 2.362427 | 6 | 1725.86 | 3.3384 |
| Horizontal with machined surface (heat-treated) | 179 | 2.362881 | 6 | 1726.7 | 3.3688 |
| Horizontal with machined surface (heat-treated) | 180 | 2.36332 | 6 | 1727.52 | 3.3993 |
| Horizontal with machined surface (heat-treated) | 181 | 2.363745 | 6 | 1728.34 | 3.4299 |
| Horizontal with machined surface (heat-treated) | 182 | 2.364157 | 6 | 1729.16 | 3.4606 |
| Horizontal with machined surface (heat-treated) | 183 | 2.364558 | 6 | 1729.96 | 3.4914 |
| Horizontal with machined surface (heat-treated) | 184 | 2.364948 | 6 | 1730.76 | 3.5223 |
| Horizontal with machined surface (heat-treated) | 185 | 2.365327 | 6 | 1731.54 | 3.5532 |
| Horizontal with machined surface (heat-treated) | 186 | 2.365694 | 6 | 1732.32 | 3.5841 |
| Horizontal with machined surface (heat-treated) | 187 | 2.36605 | 6 | 1733.1 | 3.6152 |
| Horizontal with machined surface (heat-treated) | 188 | 2.366393 | 6 | 1733.86 | 3.6463 |
| Horizontal with machined surface (heat-treated) | 189 | 2.366725 | 6 | 1734.62 | 3.6774 |
| Horizontal with machined surface (heat-treated) | 190 | 2.367045 | 6 | 1735.38 | 3.7086 |
| Horizontal with machined surface (heat-treated) | 191 | 2.367355 | 6 | 1736.12 | 3.7399 |
| Horizontal with machined surface (heat-treated) | 192 | 2.367656 | 6 | 1736.86 | 3.7712 |
| Horizontal with machined surface (heat-treated) | 193 | 2.36795 | 6 | 1737.6 | 3.8027 |
| Horizontal with machined surface (heat-treated) | 194 | 2.368238 | 6 | 1738.34 | 3.8342 |
| Horizontal with machined surface (heat-treated) | 195 | 2.368521 | 6 | 1739.08 | 3.8659 |
| Horizontal with machined surface (heat-treated) | 196 | 2.368799 | 6 | 1739.8 | 3.8977 |
| Horizontal with machined surface (heat-treated) | 197 | 2.369074 | 6 | 1740.54 | 3.9297 |
| Horizontal with machined surface (heat-treated) | 198 | 2.369345 | 6 | 1741.28 | 3.962 |
| Horizontal with machined surface (heat-treated) | 199 | 2.369612 | 6 | 1742.02 | 3.9945 |
| Horizontal with machined surface (heat-treated) | 200 | 2.369873 | 6 | 1742.76 | 4.0272 |
| Horizontal with machined surface (heat-treated) | 201 | 2.37013 | 6 | 1743.48 | 4.0602 |
| Horizontal with machined surface (heat-treated) | 202 | 2.370381 | 6 | 1744.22 | 4.0933 |
| Horizontal with machined surface (heat-treated) | 203 | 2.370628 | 6 | 1744.96 | 4.1266 |
| Horizontal with machined surface (heat-treated) | 204 | 2.370869 | 6 | 1745.7 | 4.1599 |
| Horizontal with machined surface (heat-treated) | 205 | 2.371106 | 6 | 1746.42 | 4.1934 |
| Horizontal with machined surface (heat-treated) | 206 | 2.371337 | 6 | 1747.16 | 4.2269 |
| Horizontal with machined surface (heat-treated) | 207 | 2.371562 | 6 | 1747.88 | 4.2605 |
| Horizontal with machined surface (heat-treated) | 208 | 2.37178 | 6 | 1748.6 | 4.294 |
| Horizontal with machined surface (heat-treated) | 209 | 2.371988 | 6 | 1749.32 | 4.3276 |
| Horizontal with machined surface (heat-treated) | 210 | 2.372189 | 6 | 1750.04 | 4.3611 |
| Horizontal with machined surface (heat-treated) | 211 | 2.37238 | 6 | 1750.74 | 4.3946 |
| Horizontal with machined surface (heat-treated) | 212 | 2.372562 | 6 | 1751.44 | 4.4281 |
| Horizontal with machined surface (heat-treated) | 213 | 2.372735 | 6 | 1752.12 | 4.4616 |
| Horizontal with machined surface (heat-treated) | 214 | 2.372899 | 6 | 1752.8 | 4.4951 |
| Horizontal with machined surface (heat-treated) | 215 | 2.373055 | 6 | 1753.48 | 4.5287 |
| Horizontal with machined surface (heat-treated) | 216 | 2.373204 | 6 | 1754.16 | 4.5623 |
| Horizontal with machined surface (heat-treated) | 217 | 2.373347 | 6 | 1754.82 | 4.596 |
| Horizontal with machined surface (heat-treated) | 218 | 2.373484 | 6 | 1755.5 | 4.6298 |
| Horizontal with machined surface (heat-treated) | 219 | 2.373617 | 6 | 1756.16 | 4.6638 |
| Horizontal with machined surface (heat-treated) | 220 | 2.373745 | 6 | 1756.84 | 4.6981 |
| Horizontal with machined surface (heat-treated) | 221 | 2.373868 | 6 | 1757.5 | 4.7325 |
| Horizontal with machined surface (heat-treated) | 222 | 2.373986 | 6 | 1758.18 | 4.7673 |
| Horizontal with machined surface (heat-treated) | 223 | 2.374098 | 6 | 1758.86 | 4.8023 |
| Horizontal with machined surface (heat-treated) | 224 | 2.374205 | 6 | 1759.52 | 4.8375 |
| Horizontal with machined surface (heat-treated) | 225 | 2.374307 | 6 | 1760.2 | 4.873 |
| Horizontal with machined surface (heat-treated) | 226 | 2.374403 | 6 | 1760.88 | 4.9088 |
| Horizontal with machined surface (heat-treated) | 227 | 2.374494 | 6 | 1761.54 | 4.9448 |
| Horizontal with machined surface (heat-treated) | 228 | 2.37458 | 6 | 1762.22 | 4.981 |
| Horizontal with machined surface (heat-treated) | 229 | 2.37466 | 6 | 1762.9 | 5.0174 |
| Horizontal with machined surface (heat-treated) | 230 | 2.374734 | 6 | 1763.56 | 5.0541 |
| Horizontal with machined surface (heat-treated) | 231 | 2.374802 | 6 | 1764.24 | 5.091 |
| Horizontal with machined surface (heat-treated) | 232 | 2.374864 | 6 | 1764.92 | 5.1281 |
| Horizontal with machined surface (heat-treated) | 233 | 2.374919 | 6 | 1765.58 | 5.1655 |
| Horizontal with machined surface (heat-treated) | 234 | 2.374967 | 6 | 1766.26 | 5.2032 |
| Horizontal with machined surface (heat-treated) | 235 | 2.375008 | 6 | 1766.92 | 5.2412 |
| Horizontal with machined surface (heat-treated) | 236 | 2.375042 | 6 | 1767.6 | 5.2794 |
| Horizontal with machined surface (heat-treated) | 237 | 2.375071 | 6 | 1768.28 | 5.3181 |
| Horizontal with machined surface (heat-treated) | 238 | 2.375096 | 6 | 1768.96 | 5.3571 |
| Horizontal with machined surface (heat-treated) | 239 | 2.375117 | 6 | 1769.64 | 5.3966 |
| Horizontal with machined surface (heat-treated) | 240 | 2.375135 | 6 | 1770.32 | 5.4365 |
| Horizontal with machined surface (heat-treated) | 241 | 2.37515 | 6 | 1771.02 | 5.4769 |
| Horizontal with machined surface (heat-treated) | 242 | 2.375161 | 6 | 1771.72 | 5.5178 |
| Horizontal with machined surface (heat-treated) | 243 | 2.37517 | 6 | 1772.44 | 5.5592 |
| Horizontal with machined surface (heat-treated) | 244 | 2.375178 | 6 | 1773.16 | 5.6011 |
| Horizontal with machined surface (heat-treated) | 245 | 2.375184 | 6 | 1773.88 | 5.6435 |
| Horizontal with machined surface (heat-treated) | 246 | 2.375188 | 6 | 1774.6 | 5.6864 |
| Horizontal with machined surface (heat-treated) | 247 | **2.375189** | 6 | **1775.34** | **5.7298** |
| Horizontal with machined surface (heat-treated) | 248 | 2.375186 | 6 | 1776.1 | 5.7739 |
| Horizontal with machined surface (heat-treated) | 249 | 2.375177 | 6 | 1776.84 | 5.8185 |
| Horizontal with machined surface (heat-treated) | 250 | 2.375162 | 6 | 1777.6 | 5.8638 |
| Horizontal with machined surface (heat-treated) | 251 | 2.375139 | 6 | 1778.36 | 5.9096 |
| Horizontal with machined surface (heat-treated) | 252 | 2.375108 | 6 | 1779.14 | 5.9562 |
| Horizontal with machined surface (heat-treated) | 253 | 2.375071 | 6 | 1779.92 | 6.0033 |
| Horizontal with machined surface (heat-treated) | 254 | 2.375027 | 6 | 1780.7 | 6.0511 |
| Horizontal with machined surface (heat-treated) | 255 | 2.374977 | 6 | 1781.48 | 6.0994 |
| Horizontal with machined surface (heat-treated) | 256 | 2.374923 | 6 | 1782.28 | 6.1484 |
| Horizontal with machined surface (heat-treated) | 257 | 2.374861 | 6 | 1783.08 | 6.1979 |
| Horizontal with machined surface (heat-treated) | 258 | 2.374791 | 6 | 1783.88 | 6.2479 |
| Horizontal with machined surface (heat-treated) | 259 | 2.374709 | 6 | 1784.68 | 6.2985 |
| Horizontal with machined surface (heat-treated) | 260 | 2.374614 | 6 | 1785.48 | 6.3495 |
| Horizontal with machined surface (heat-treated) | 261 | 2.374504 | 6 | 1786.28 | 6.4009 |
| Horizontal with machined surface (heat-treated) | 262 | 2.374382 | 6 | 1787.06 | 6.4528 |
| Horizontal with machined surface (heat-treated) | 263 | 2.374248 | 6 | 1787.86 | 6.5051 |
| Horizontal with machined surface (heat-treated) | 264 | 2.374104 | 6 | 1788.66 | 6.5579 |
| Horizontal with machined surface (heat-treated) | 265 | 2.373951 | 6 | 1789.44 | 6.6111 |
| Horizontal with machined surface (heat-treated) | 266 | 2.373789 | 6 | 1790.24 | 6.6648 |
| Horizontal with machined surface (heat-treated) | 267 | 2.373615 | 6 | 1791.04 | 6.7191 |
| Horizontal with machined surface (heat-treated) | 268 | 2.373428 | 6 | 1791.84 | 6.774 |
| Horizontal with machined surface (heat-treated) | 269 | 2.373225 | 6 | 1792.64 | 6.8296 |
| Horizontal with machined surface (heat-treated) | 270 | 2.373004 | 6 | 1793.44 | 6.8861 |
| Horizontal with machined surface (heat-treated) | 271 | 2.372762 | 6 | 1794.24 | 6.9434 |
| Horizontal with machined surface (heat-treated) | 272 | 2.372497 | 6 | 1795.06 | 7.0017 |
| Horizontal with machined surface (heat-treated) | 273 | 2.372209 | 6 | 1795.86 | 7.061 |
| Horizontal with machined surface (heat-treated) | 274 | 2.371897 | 6 | 1796.66 | 7.1213 |
| Horizontal with machined surface (heat-treated) | 275 | 2.371561 | 6 | 1797.46 | 7.1828 |
| Horizontal with machined surface (heat-treated) | 276 | 2.371203 | 6 | 1798.26 | 7.2454 |
| Horizontal with machined surface (heat-treated) | 277 | 2.370823 | 6 | 1799.06 | 7.3091 |
| Horizontal with machined surface (heat-treated) | 278 | 2.370424 | 6 | 1799.88 | 7.374 |
| Horizontal with machined surface (heat-treated) | 279 | 2.37001 | 6 | 1800.7 | 7.4401 |
| Horizontal with machined surface (heat-treated) | 280 | 2.369583 | 6 | 1801.54 | 7.5072 |
| Horizontal with machined surface (heat-treated) | 281 | 2.369148 | 6 | 1802.38 | 7.5754 |
| Horizontal with machined surface (heat-treated) | 282 | 2.368706 | 6 | 1803.24 | 7.6446 |
| Horizontal with machined surface (heat-treated) | 283 | 2.36826 | 6 | 1804.12 | 7.7148 |
| Horizontal with machined surface (heat-treated) | 284 | 2.367813 | 6 | 1805 | 7.786 |
| Horizontal with machined surface (heat-treated) | 285 | 2.367368 | 6 | 1805.92 | 7.8581 |
| Horizontal with machined surface (heat-treated) | 286 | 2.366929 | 6 | 1806.84 | 7.9311 |
| Horizontal with machined surface (heat-treated) | 287 | 2.366496 | 6 | 1807.78 | 8.0049 |
| Horizontal with machined surface (heat-treated) | 288 | 2.366066 | 6 | 1808.74 | 8.0796 |
| Horizontal with machined surface (heat-treated) | 289 | 2.365638 | 6 | 1809.72 | 8.155 |
| Horizontal with machined surface (heat-treated) | 290 | 2.365207 | 6 | 1810.72 | 8.2312 |
| Horizontal with machined surface (heat-treated) | 291 | 2.364769 | 6 | 1811.72 | 8.3081 |
| Horizontal with machined surface (heat-treated) | 292 | 2.364323 | 6 | 1812.72 | 8.3857 |
| Horizontal with machined surface (heat-treated) | 293 | 2.363865 | 6 | 1813.72 | 8.4639 |
| Horizontal with machined surface (heat-treated) | 294 | 2.363396 | 6 | 1814.74 | 8.5426 |
| Horizontal with machined surface (heat-treated) | 295 | 2.362916 | 6 | 1815.74 | 8.6218 |
| Horizontal with machined surface (heat-treated) | 296 | 2.362424 | 6 | 1816.74 | 8.7015 |
| Horizontal with machined surface (heat-treated) | 297 | 2.361919 | 6 | 1817.74 | 8.7815 |
| Horizontal with machined surface (heat-treated) | 298 | 2.361402 | 6 | 1818.74 | 8.8619 |
| Horizontal with machined surface (heat-treated) | 299 | 2.360869 | 6 | 1819.74 | 8.9426 |
| Horizontal with machined surface (heat-treated) | 300 | 2.36032 | 6 | 1820.72 | 9.0236 |
| Horizontal with machined surface (heat-treated) | 301 | 2.359753 | 6 | 1821.7 | 9.1048 |
| Horizontal with machined surface (heat-treated) | 302 | 2.359167 | 6 | 1822.68 | 9.1864 |
| Horizontal with machined surface (heat-treated) | 303 | 2.358562 | 6 | 1823.64 | 9.2683 |
| Horizontal with machined surface (heat-treated) | 304 | 2.357938 | 6 | 1824.58 | 9.3506 |
| Horizontal with machined surface (heat-treated) | 305 | 2.357295 | 6 | 1825.54 | 9.4333 |
| Horizontal with machined surface (heat-treated) | 306 | 2.356633 | 6 | 1826.48 | 9.5165 |
| Horizontal with machined surface (heat-treated) | 307 | 2.35595 | 6 | 1827.4 | 9.6002 |
| Horizontal with machined surface (heat-treated) | 308 | 2.355247 | 6 | 1828.34 | 9.6846 |
| Horizontal with machined surface (heat-treated) | 309 | 2.35452 | 6 | 1829.26 | 9.7697 |
| Horizontal with machined surface (heat-treated) | 310 | 2.35377 | 6 | 1830.18 | 9.8555 |
| Horizontal with machined surface (heat-treated) | 311 | 2.352996 | 6 | 1831.1 | 9.9421 |
| Horizontal with machined surface (heat-treated) | 312 | 2.352196 | 6 | 1832 | 10.0295 |
| Horizontal with machined surface (heat-treated) | 313 | 2.351373 | 6 | 1832.9 | 10.1177 |
| Horizontal with machined surface (heat-treated) | 314 | 2.350525 | 6 | 1833.8 | 10.2067 |
| Horizontal with machined surface (heat-treated) | 315 | 2.349653 | 6 | 1834.7 | 10.2966 |
| Horizontal with machined surface (heat-treated) | 316 | 2.348754 | 6 | 1835.58 | 10.3873 |
| Horizontal with machined surface (heat-treated) | 317 | 2.347829 | 6 | 1836.46 | 10.4788 |
| Horizontal with machined surface (heat-treated) | 318 | 2.346876 | 6 | 1837.34 | 10.5711 |
| Horizontal with machined surface (heat-treated) | 319 | 2.345895 | 6 | 1838.2 | 10.6641 |
| Horizontal with machined surface (heat-treated) | 320 | 2.344885 | 6 | 1839.06 | 10.7579 |
| Horizontal with machined surface (heat-treated) | 321 | 2.343847 | 6 | 1839.9 | 10.8524 |
| Horizontal with machined surface (heat-treated) | 322 | 2.342784 | 6 | 1840.76 | 10.9477 |
| Horizontal with machined surface (heat-treated) | 323 | 2.341697 | 6 | 1841.58 | 11.0438 |
| Horizontal with machined surface (heat-treated) | 324 | 2.340586 | 6 | 1842.42 | 11.1407 |
| Horizontal with machined surface (heat-treated) | 325 | 2.339455 | 6 | 1843.26 | 11.2385 |
| Horizontal with machined surface (heat-treated) | 326 | 2.338303 | 6 | 1844.08 | 11.3373 |
| Horizontal with machined surface (heat-treated) | 327 | 2.337132 | 6 | 1844.92 | 11.437 |
| Horizontal with machined surface (heat-treated) | 328 | 2.33594 | 6 | 1845.76 | 11.5378 |
| Horizontal with machined surface (heat-treated) | 329 | 2.33473 | 6 | 1846.6 | 11.6396 |
| Horizontal with machined surface (heat-treated) | 330 | 2.3335 | 6 | 1847.44 | 11.7425 |
| Horizontal with machined surface (heat-treated) | 331 | 2.332252 | 6 | 1848.28 | 11.8463 |
| Horizontal with machined surface (heat-treated) | 332 | 2.330988 | 6 | 1849.14 | 11.9512 |
| Horizontal with machined surface (heat-treated) | 333 | 2.32971 | 6 | 1849.98 | 12.0571 |
| Horizontal with machined surface (heat-treated) | 334 | 2.328417 | 6 | 1850.86 | 12.1639 |
| Horizontal with machined surface (heat-treated) | 335 | 2.327114 | 6 | 1851.72 | 12.2717 |
| Horizontal with machined surface (heat-treated) | 336 | 2.325799 | 6 | 1852.6 | 12.3804 |
| Horizontal with machined surface (heat-treated) | 337 | 2.324476 | 6 | 1853.48 | 12.4901 |
| Horizontal with machined surface (heat-treated) | 338 | 2.323143 | 6 | 1854.38 | 12.6008 |
| Horizontal with machined surface (heat-treated) | 339 | 2.321803 | 6 | 1855.3 | 12.7123 |
| Horizontal with machined surface (heat-treated) | 340 | 2.320454 | 6 | 1856.22 | 12.8248 |
| Horizontal with machined surface (heat-treated) | 341 | 2.319096 | 6 | 1857.14 | 12.9382 |
| Horizontal with machined surface (heat-treated) | 342 | 2.317728 | 6 | 1858.08 | 13.0525 |
| Horizontal with machined surface (heat-treated) | 343 | 2.316352 | 6 | 1859.02 | 13.1675 |
| Horizontal with machined surface (heat-treated) | 344 | 2.314965 | 6 | 1859.96 | 13.2834 |
| Horizontal with machined surface (heat-treated) | 345 | 2.313569 | 6 | 1860.92 | 13.4 |
| Horizontal with machined surface (heat-treated) | 346 | 2.312165 | 6 | 1861.86 | 13.5174 |
| Horizontal with machined surface (heat-treated) | 347 | 2.310754 | 6 | 1862.84 | 13.6354 |
| Horizontal with machined surface (heat-treated) | 348 | 2.309337 | 6 | 1863.8 | 13.7542 |
| Horizontal with machined surface (heat-treated) | 349 | 2.307915 | 6 | 1864.78 | 13.8736 |
| Horizontal with machined surface (heat-treated) | 350 | 2.306486 | 6 | 1865.78 | 13.9937 |
| Horizontal with machined surface (heat-treated) | 351 | 2.30505 | 6 | 1866.76 | 14.1146 |
| Horizontal with machined surface (heat-treated) | 352 | 2.303603 | 6 | 1867.76 | 14.2362 |
| Horizontal with machined surface (heat-treated) | 353 | 2.302145 | 6 | 1868.76 | 14.3586 |
| Horizontal with machined surface (heat-treated) | 354 | 2.30067 | 6 | 1869.76 | 14.4817 |
| Horizontal with machined surface (heat-treated) | 355 | 2.299178 | 6 | 1870.76 | 14.6057 |
| Horizontal with machined surface (heat-treated) | 356 | 2.297666 | 6 | 1871.76 | 14.7304 |
| Horizontal with machined surface (heat-treated) | 357 | 2.296134 | 6 | 1872.76 | 14.8559 |
| Horizontal with machined surface (heat-treated) | 358 | 2.294584 | 6 | 1873.76 | 14.9823 |
| Horizontal with machined surface (heat-treated) | 359 | 2.293018 | 6 | 1874.76 | 15.1095 |
| Horizontal with machined surface (heat-treated) | 360 | 2.291438 | 6 | 1875.76 | 15.2376 |
| Horizontal with machined surface (heat-treated) | 361 | 2.289847 | 6 | 1876.76 | 15.3665 |
| Horizontal with machined surface (heat-treated) | 362 | 2.288244 | 6 | 1877.78 | 15.4962 |
| Horizontal with machined surface (heat-treated) | 363 | 2.286631 | 6 | 1878.8 | 15.6267 |
| Horizontal with machined surface (heat-treated) | 364 | 2.285007 | 6 | 1879.82 | 15.758 |
| Horizontal with machined surface (heat-treated) | 365 | 2.283372 | 6 | 1880.84 | 15.89 |
| Horizontal with machined surface (heat-treated) | 366 | 2.281726 | 6 | 1881.88 | 16.0228 |
| Horizontal with machined surface (heat-treated) | 367 | 2.280067 | 6 | 1882.9 | 16.1563 |
| Horizontal with machined surface (heat-treated) | 368 | 2.278396 | 6 | 1883.94 | 16.2904 |
| Horizontal with machined surface (heat-treated) | 369 | 2.276709 | 6 | 1884.96 | 16.4252 |
| Horizontal with machined surface (heat-treated) | 370 | 2.275006 | 6 | 1886 | 16.5605 |
| Horizontal with machined surface (heat-treated) | 371 | 2.273285 | 6 | 1887.02 | 16.6964 |
| Horizontal with machined surface (heat-treated) | 372 | 2.271547 | 6 | 1888.02 | 16.8327 |
| Horizontal with machined surface (heat-treated) | 373 | 2.269789 | 6 | 1889.02 | 16.9693 |
| Horizontal with machined surface (heat-treated) | 374 | 2.268013 | 6 | 1890.02 | 17.1063 |
| Horizontal with machined surface (heat-treated) | 375 | 2.266217 | 6 | 1890.98 | 17.2434 |
| Horizontal with machined surface (heat-treated) | 376 | 2.2644 | 6 | 1891.94 | 17.3807 |
| Horizontal with machined surface (heat-treated) | 377 | 2.262562 | 6 | 1892.9 | 17.518 |
| Horizontal with machined surface (heat-treated) | 378 | 2.2607 | 6 | 1893.82 | 17.6552 |
| Horizontal with machined surface (heat-treated) | 379 | 2.258814 | 6 | 1894.7 | 17.7923 |
| Horizontal with machined surface (heat-treated) | 380 | 2.256905 | 6 | 1895.58 | 17.9293 |
| Horizontal with machined surface (heat-treated) | 381 | 2.254975 | 6 | 1896.44 | 18.0659 |
| Horizontal with machined surface (heat-treated) | 382 | 2.253026 | 6 | 1897.26 | 18.2023 |
| Horizontal with machined surface (heat-treated) | 383 | 2.25106 | 6 | 1898.06 | 18.3384 |
| Horizontal with machined surface (heat-treated) | 384 | 2.24908 | 6 | 1898.86 | 18.4742 |
| Horizontal with machined surface (heat-treated) | 385 | 2.247088 | 6 | 1899.62 | 18.6098 |
| Horizontal with machined surface (heat-treated) | 386 | 2.245083 | 6 | 1900.38 | 18.7451 |
| Horizontal with machined surface (heat-treated) | 387 | 2.243067 | 6 | 1901.12 | 18.8802 |
| Horizontal with machined surface (heat-treated) | 388 | 2.241039 | 6 | 1901.84 | 19.0151 |
| Horizontal with machined surface (heat-treated) | 389 | 2.238999 | 6 | 1902.56 | 19.1501 |
| Horizontal with machined surface (heat-treated) | 390 | 2.236945 | 6 | 1903.28 | 19.2851 |
| Horizontal with machined surface (heat-treated) | 391 | 2.234878 | 6 | 1903.96 | 19.4203 |
| Horizontal with machined surface (heat-treated) | 392 | 2.232795 | 6 | 1904.66 | 19.5558 |
| Horizontal with machined surface (heat-treated) | 393 | 2.230699 | 6 | 1905.32 | 19.6917 |
| Horizontal with machined surface (heat-treated) | 394 | 2.228588 | 6 | 1906 | 19.8281 |
| Horizontal with machined surface (heat-treated) | 395 | 2.226464 | 6 | 1906.68 | 19.9652 |
| Horizontal with machined surface (heat-treated) | 396 | 2.224326 | 6 | 1907.36 | 20.103 |
| Horizontal with machined surface (heat-treated) | 397 | 2.222175 | 6 | 1908.04 | 20.2416 |
| Horizontal with machined surface (heat-treated) | 398 | 2.220013 | 6 | 1908.72 | 20.3812 |
| Horizontal with machined surface (heat-treated) | 399 | 2.217839 | 6 | 1909.4 | 20.5218 |
| Horizontal with machined surface (heat-treated) | 400 | 2.215654 | 6 | 1910.1 | 20.6635 |
| Horizontal with machined surface (heat-treated) | 401 | 2.213458 | 6 | 1910.82 | 20.8062 |
| Horizontal with machined surface (heat-treated) | 402 | 2.211251 | 6 | 1911.52 | 20.9501 |
| Horizontal with machined surface (heat-treated) | 403 | 2.20903 | 6 | 1912.26 | 21.0952 |
| Horizontal with machined surface (heat-treated) | 404 | 2.206793 | 6 | 1912.98 | 21.2414 |
| Horizontal with machined surface (heat-treated) | 405 | 2.204539 | 6 | 1913.72 | 21.3888 |
| Horizontal with machined surface (heat-treated) | 406 | 2.202267 | 6 | 1914.46 | 21.5372 |
| Horizontal with machined surface (heat-treated) | 407 | 2.199978 | 6 | 1915.2 | 21.6866 |
| Horizontal with machined surface (heat-treated) | 408 | 2.197677 | 6 | 1915.94 | 21.8371 |
| Horizontal with machined surface (heat-treated) | 409 | 2.195367 | 6 | 1916.68 | 21.9884 |
| Horizontal with machined surface (heat-treated) | 410 | 2.193047 | 6 | 1917.44 | 22.1406 |
| Horizontal with machined surface (heat-treated) | 411 | 2.190718 | 6 | 1918.2 | 22.2936 |
| Horizontal with machined surface (heat-treated) | 412 | 2.188379 | 6 | 1918.96 | 22.4474 |
| Horizontal with machined surface (heat-treated) | 413 | 2.186028 | 6 | 1919.74 | 22.6018 |
| Horizontal with machined surface (heat-treated) | 414 | 2.183668 | 6 | 1920.5 | 22.757 |
| Horizontal with machined surface (heat-treated) | 415 | 2.1813 | 6 | 1921.26 | 22.9128 |
| Horizontal with machined surface (heat-treated) | 416 | 2.178924 | 6 | 1922.04 | 23.0691 |
| Horizontal with machined surface (heat-treated) | 417 | 2.176541 | 6 | 1922.8 | 23.2261 |
| Horizontal with machined surface (heat-treated) | 418 | 2.174152 | 6 | 1923.58 | 23.3837 |
| Horizontal with machined surface (heat-treated) | 419 | 2.171756 | 6 | 1924.36 | 23.5418 |
| Horizontal with machined surface (heat-treated) | 420 | 2.169351 | 6 | 1925.14 | 23.7005 |
| Horizontal with machined surface (heat-treated) | 421 | 2.166938 | 6 | 1925.92 | 23.8597 |
| Horizontal with machined surface (heat-treated) | 422 | 2.164514 | 6 | 1926.7 | 24.0194 |
| Horizontal with machined surface (heat-treated) | 423 | 2.162081 | 6 | 1927.46 | 24.1796 |
| Horizontal with machined surface (heat-treated) | 424 | 2.159636 | 6 | 1928.24 | 24.3403 |
| Horizontal with machined surface (heat-treated) | 425 | 2.157181 | 6 | 1929 | 24.5014 |
| Horizontal with machined surface (heat-treated) | 426 | 2.154713 | 6 | 1929.76 | 24.663 |
| Horizontal with machined surface (heat-treated) | 427 | 2.152232 | 6 | 1930.52 | 24.825 |
| Horizontal with machined surface (heat-treated) | 428 | 2.149736 | 6 | 1931.26 | 24.9875 |
| Horizontal with machined surface (heat-treated) | 429 | 2.147223 | 6 | 1932 | 25.1505 |
| Horizontal with machined surface (heat-treated) | 430 | 2.144693 | 6 | 1932.74 | 25.3141 |
| Horizontal with machined surface (heat-treated) | 431 | 2.142146 | 6 | 1933.46 | 25.4782 |
| Horizontal with machined surface (heat-treated) | 432 | 2.139582 | 6 | 1934.18 | 25.643 |
| Horizontal with machined surface (heat-treated) | 433 | 2.137 | 6 | 1934.88 | 25.8084 |
| Horizontal with machined surface (heat-treated) | 434 | 2.1344 | 6 | 1935.58 | 25.9746 |
| Horizontal with machined surface (heat-treated) | 435 | 2.131781 | 6 | 1936.28 | 26.1416 |
| Horizontal with machined surface (heat-treated) | 436 | 2.129142 | 6 | 1936.98 | 26.3094 |
| Horizontal with machined surface (heat-treated) | 437 | 2.126483 | 6 | 1937.68 | 26.4782 |
| Horizontal with machined surface (heat-treated) | 438 | 2.123804 | 6 | 1938.36 | 26.6481 |
| Horizontal with machined surface (heat-treated) | 439 | 2.121107 | 6 | 1939.06 | 26.8191 |
| Horizontal with machined surface (heat-treated) | 440 | 2.118394 | 6 | 1939.74 | 26.9913 |
| Horizontal with machined surface (heat-treated) | 441 | 2.115666 | 6 | 1940.44 | 27.1647 |
| Horizontal with machined surface (heat-treated) | 442 | 2.112926 | 6 | 1941.16 | 27.3393 |
| Horizontal with machined surface (heat-treated) | 443 | 2.110176 | 6 | 1941.88 | 27.5152 |
| Horizontal with machined surface (heat-treated) | 444 | 2.107418 | 6 | 1942.6 | 27.6923 |
| Horizontal with machined surface (heat-treated) | 445 | 2.104653 | 6 | 1943.36 | 27.8705 |
| Horizontal with machined surface (heat-treated) | 446 | 2.101881 | 6 | 1944.1 | 28.0499 |
| Horizontal with machined surface (heat-treated) | 447 | 2.099103 | 6 | 1944.88 | 28.2303 |
| Horizontal with machined surface (heat-treated) | 448 | 2.096318 | 6 | 1945.64 | 28.4117 |
| Horizontal with machined surface (heat-treated) | 449 | 2.093525 | 6 | 1946.42 | 28.594 |
| Horizontal with machined surface (heat-treated) | 450 | 2.090725 | 6 | 1947.2 | 28.777 |
| Horizontal with machined surface (heat-treated) | 451 | 2.087917 | 6 | 1948 | 28.9607 |
| Horizontal with machined surface (heat-treated) | 452 | 2.0851 | 6 | 1948.78 | 29.1449 |
| Horizontal with machined surface (heat-treated) | 453 | 2.082276 | 6 | 1949.56 | 29.3297 |
| Horizontal with machined surface (heat-treated) | 454 | 2.079446 | 6 | 1950.34 | 29.515 |
| Horizontal with machined surface (heat-treated) | 455 | 2.076611 | 6 | 1951.12 | 29.7005 |
| Horizontal with machined surface (heat-treated) | 456 | 2.073773 | 6 | 1951.9 | 29.8864 |
| Horizontal with machined surface (heat-treated) | 457 | 2.070934 | 6 | 1952.68 | 30.0725 |
| Horizontal with machined surface (heat-treated) | 458 | 2.068096 | 6 | 1953.46 | 30.2587 |
| Horizontal with machined surface (heat-treated) | 459 | 2.06526 | 6 | 1954.26 | 30.4451 |
| Horizontal with machined surface (heat-treated) | 460 | 2.062427 | 6 | 1955.04 | 30.6316 |
| Horizontal with machined surface (heat-treated) | 461 | 2.059597 | 6 | 1955.82 | 30.8183 |
| Horizontal with machined surface (heat-treated) | 462 | 2.05677 | 6 | 1956.6 | 31.0051 |
| Horizontal with machined surface (heat-treated) | 463 | 2.053945 | 6 | 1957.4 | 31.1921 |
| Horizontal with machined surface (heat-treated) | 464 | 2.05112 | 6 | 1958.18 | 31.3793 |
| Horizontal with machined surface (heat-treated) | 465 | 2.048296 | 6 | 1958.98 | 31.5669 |
| Horizontal with machined surface (heat-treated) | 466 | 2.045471 | 6 | 1959.78 | 31.7548 |
| Horizontal with machined surface (heat-treated) | 467 | 2.042646 | 6 | 1960.58 | 31.9431 |
| Horizontal with machined surface (heat-treated) | 468 | 2.03982 | 6 | 1961.38 | 32.1318 |
| Horizontal with machined surface (heat-treated) | 469 | 2.03699 | 6 | 1962.18 | 32.3212 |
| Horizontal with machined surface (heat-treated) | 470 | 2.034153 | 6 | 1962.98 | 32.5111 |
| Horizontal with machined surface (heat-treated) | 471 | 2.031307 | 6 | 1963.78 | 32.7018 |
| Horizontal with machined surface (heat-treated) | 472 | 2.028449 | 6 | 1964.6 | 32.8931 |
| Horizontal with machined surface (heat-treated) | 473 | 2.025575 | 6 | 1965.38 | 33.0852 |
| Horizontal with machined surface (heat-treated) | 474 | 2.022684 | 6 | 1966.18 | 33.278 |
| Horizontal with machined surface (heat-treated) | 475 | 2.019775 | 6 | 1966.96 | 33.4714 |
| Horizontal with machined surface (heat-treated) | 476 | 2.016849 | 6 | 1967.72 | 33.6655 |
| Horizontal with machined surface (heat-treated) | 477 | 2.013905 | 6 | 1968.48 | 33.8602 |
| Horizontal with machined surface (heat-treated) | 478 | 2.010944 | 6 | 1969.24 | 34.0556 |
| Horizontal with machined surface (heat-treated) | 479 | 2.007968 | 6 | 1969.98 | 34.2516 |
| Horizontal with machined surface (heat-treated) | 480 | 2.004975 | 6 | 1970.72 | 34.4485 |
| Horizontal with machined surface (heat-treated) | 481 | 2.001965 | 6 | 1971.46 | 34.6461 |
| Horizontal with machined surface (heat-treated) | 482 | 1.998937 | 6 | 1972.18 | 34.8447 |
| Horizontal with machined surface (heat-treated) | 483 | 1.995891 | 6 | 1972.92 | 35.0443 |
| Horizontal with machined surface (heat-treated) | 484 | 1.992826 | 6 | 1973.64 | 35.2451 |
| Horizontal with machined surface (heat-treated) | 485 | 1.989743 | 6 | 1974.36 | 35.4471 |
| Horizontal with machined surface (heat-treated) | 486 | 1.986643 | 6 | 1975.1 | 35.6506 |
| Horizontal with machined surface (heat-treated) | 487 | 1.983524 | 6 | 1975.82 | 35.8557 |
| Horizontal with machined surface (heat-treated) | 488 | 1.980388 | 6 | 1976.58 | 36.0625 |
| Horizontal with machined surface (heat-treated) | 489 | 1.977236 | 6 | 1977.34 | 36.2713 |
| Horizontal with machined surface (heat-treated) | 490 | 1.974068 | 6 | 1978.12 | 36.482 |
| Horizontal with machined surface (heat-treated) | 491 | 1.970886 | 6 | 1978.92 | 36.6949 |
| Horizontal with machined surface (heat-treated) | 492 | 1.967689 | 6 | 1979.74 | 36.91 |
| Horizontal with machined surface (heat-treated) | 493 | 1.964479 | 6 | 1980.6 | 37.1272 |
| Horizontal with machined surface (heat-treated) | 494 | 1.961255 | 6 | 1981.46 | 37.3467 |
| Horizontal with machined surface (heat-treated) | 495 | 1.958019 | 6 | 1982.36 | 37.5684 |
| Horizontal with machined surface (heat-treated) | 496 | 1.954771 | 6 | 1983.28 | 37.7922 |
| Horizontal with machined surface (heat-treated) | 497 | 1.951511 | 6 | 1984.22 | 38.0181 |
| Horizontal with machined surface (heat-treated) | 498 | 1.948237 | 6 | 1985.16 | 38.2459 |
| Horizontal with machined surface (heat-treated) | 499 | 1.94495 | 6 | 1986.14 | 38.4755 |
| Horizontal with machined surface (heat-treated) | 500 | 1.941649 | 6 | 1987.12 | 38.7068 |
| Horizontal with machined surface (heat-treated) | 501 | 1.938333 | 6 | 1988.12 | 38.9398 |
| Horizontal with machined surface (heat-treated) | 502 | 1.935003 | 6 | 1989.12 | 39.1742 |
| Horizontal with machined surface (heat-treated) | 503 | 1.931661 | 6 | 1990.12 | 39.41 |
| Horizontal with machined surface (heat-treated) | 504 | 1.928307 | 6 | 1991.14 | 39.6471 |
| Horizontal with machined surface (heat-treated) | 505 | 1.924942 | 6 | 1992.16 | 39.8854 |
| Horizontal with machined surface (heat-treated) | 506 | 1.921566 | 6 | 1993.18 | 40.1247 |
| Horizontal with machined surface (heat-treated) | 507 | 1.91818 | 6 | 1994.2 | 40.365 |
| Horizontal with machined surface (heat-treated) | 508 | 1.914783 | 6 | 1995.22 | 40.6062 |
| Horizontal with machined surface (heat-treated) | 509 | 1.911374 | 6 | 1996.24 | 40.8482 |
| Horizontal with machined surface (heat-treated) | 510 | 1.907953 | 6 | 1997.24 | 41.091 |
| Horizontal with machined surface (heat-treated) | 511 | 1.90452 | 6 | 1998.26 | 41.3345 |
| Horizontal with machined surface (heat-treated) | 512 | 1.901074 | 6 | 1999.26 | 41.5787 |
| Horizontal with machined surface (heat-treated) | 513 | 1.897616 | 6 | 2000.24 | 41.8235 |
| Horizontal with machined surface (heat-treated) | 514 | 1.89415 | 6 | 2001.24 | 42.0688 |
| Horizontal with machined surface (heat-treated) | 515 | 1.890676 | 6 | 2002.2 | 42.3146 |
| Horizontal with machined surface (heat-treated) | 516 | 1.887195 | 6 | 2003.18 | 42.561 |
| Horizontal with machined surface (heat-treated) | 517 | 1.883709 | 6 | 2004.16 | 42.8078 |
| Horizontal with machined surface (heat-treated) | 518 | 1.880218 | 6 | 2005.12 | 43.0552 |
| Horizontal with machined surface (heat-treated) | 519 | 1.876723 | 6 | 2006.1 | 43.3033 |
| Horizontal with machined surface (heat-treated) | 520 | 1.873225 | 6 | 2007.08 | 43.5521 |
| Horizontal with machined surface (heat-treated) | 521 | 1.869724 | 6 | 2008.06 | 43.8017 |
| Horizontal with machined surface (heat-treated) | 522 | 1.866217 | 6 | 2009.04 | 44.0523 |
| Horizontal with machined surface (heat-treated) | 523 | 1.862706 | 6 | 2010.04 | 44.3041 |
| Horizontal with machined surface (heat-treated) | 524 | 1.859189 | 6 | 2011.04 | 44.557 |
| Horizontal with machined surface (heat-treated) | 525 | 1.855666 | 6 | 2012.06 | 44.8112 |
| Horizontal with machined surface (heat-treated) | 526 | 1.852138 | 6 | 2013.08 | 45.0667 |
| Horizontal with machined surface (heat-treated) | 527 | 1.848606 | 6 | 2014.12 | 45.3237 |
| Horizontal with machined surface (heat-treated) | 528 | 1.845068 | 6 | 2015.18 | 45.5822 |
| Horizontal with machined surface (heat-treated) | 529 | 1.841524 | 6 | 2016.26 | 45.8423 |
| Horizontal with machined surface (heat-treated) | 530 | 1.837971 | 6 | 2017.34 | 46.104 |
| Horizontal with machined surface (heat-treated) | 531 | 1.834407 | 6 | 2018.44 | 46.3673 |
| Horizontal with machined surface (heat-treated) | 532 | 1.830829 | 6 | 2019.56 | 46.6321 |
| Horizontal with machined surface (heat-treated) | 533 | 1.827236 | 6 | 2020.68 | 46.8986 |
| Horizontal with machined surface (heat-treated) | 534 | 1.823628 | 6 | 2021.8 | 47.1666 |
| Horizontal with machined surface (heat-treated) | 535 | 1.820005 | 6 | 2022.92 | 47.436 |
| Horizontal with machined surface (heat-treated) | 536 | 1.816369 | 6 | 2024.06 | 47.7067 |
| Horizontal with machined surface (heat-treated) | 537 | 1.81272 | 6 | 2025.2 | 47.9788 |
| Horizontal with machined surface (heat-treated) | 538 | 1.809062 | 6 | 2026.34 | 48.252 |
| Horizontal with machined surface (heat-treated) | 539 | 1.805393 | 6 | 2027.5 | 48.5264 |
| Horizontal with machined surface (heat-treated) | 540 | 1.801715 | 6 | 2028.66 | 48.8019 |
| Horizontal with machined surface (heat-treated) | 541 | 1.798027 | 6 | 2029.82 | 49.0783 |
| Horizontal with machined surface (heat-treated) | 542 | 1.794331 | 6 | 2030.98 | 49.3556 |
| Horizontal with machined surface (heat-treated) | 543 | 1.790628 | 6 | 2032.16 | 49.6337 |
| Horizontal with machined surface (heat-treated) | 544 | 1.786918 | 6 | 2033.32 | 49.9124 |
| Horizontal with machined surface (heat-treated) | 545 | 1.783202 | 6 | 2034.5 | 50.1916 |
| Horizontal with machined surface (heat-treated) | 546 | 1.779483 | 6 | 2035.68 | 50.4711 |
| Horizontal with machined surface (heat-treated) | 547 | 1.775761 | 6 | 2036.84 | 50.7509 |
| Horizontal with machined surface (heat-treated) | 548 | 1.772036 | 6 | 2038.02 | 51.0308 |
| Horizontal with machined surface (heat-treated) | 549 | 1.768309 | 6 | 2039.2 | 51.3107 |
| Horizontal with machined surface (heat-treated) | 550 | 1.764582 | 6 | 2040.36 | 51.5907 |
